# Transcriptome analysis identifies differentially expressed genes in the progenies of a cross between two low phytic acid soybean mutants

**DOI:** 10.1038/s41598-021-88055-4

**Published:** 2021-04-22

**Authors:** Hangxia Jin, Xiaomin Yu, Qinghua Yang, Xujun Fu, Fengjie Yuan

**Affiliations:** Institute of Crop Science and Nuclear Technology Utilization, Zhejiang Academy of Agricultural Sciences, Hangzhou, Zhejiang China

**Keywords:** Genetics, Plant sciences

## Abstract

Phytic acid (PA) is a major antinutrient that cannot be digested by monogastric animals, but it can decrease the bioavailability of micronutrients (e.g., Zn and Fe). Lowering the PA content of crop seeds will lead to enhanced nutritional traits. Low-PA mutant crop lines carrying more than one mutated gene (*lpa*) have lower PA contents than mutants with a single *lpa* mutant gene. However, little is known about the link between PA pathway intermediates and downstream regulatory activities following the mutation of these genes in soybean. Consequently, we performed a comparative transcriptome analysis using an advanced generation recombinant inbred line with low PA levels [*2mlpa* (*mips1*/*ipk1*)] and a sibling line with homozygous non-mutant alleles and normal PA contents [2MWT (*MIPS1*/*IPK1*)]. An RNA sequencing analysis of five seed developmental stages revealed 7945 differentially expressed genes (DEGs) between the *2mlpa* and 2MWT seeds. Moreover, 3316 DEGs were associated with 128 metabolic and signal transduction pathways and 4980 DEGs were annotated with 345 Gene Ontology terms related to biological processes. Genes associated with PA metabolism, photosynthesis, starch and sucrose metabolism, and defense mechanisms were among the DEGs in *2mlpa*. Of these genes, 36 contributed to PA metabolism, including 22 genes possibly mediating the low-PA phenotype of *2mlpa*. The expression of most of the genes associated with photosynthesis (81 of 117) was down-regulated in *2mlpa* at the late seed developmental stage. In contrast, the expression of three genes involved in sucrose metabolism was up-regulated at the late seed developmental stage, which might explain the high sucrose content of *2mlpa* soybeans. Furthermore, 604 genes related to defense mechanisms were differentially expressed between *2mlpa* and 2MWT. In this study, we detected a low PA content as well as changes to multiple metabolites in the *2mlpa* mutant. These results may help elucidate the regulation of metabolic events in *2mlpa*. Many genes involved in PA metabolism may contribute to the substantial decrease in the PA content and the moderate accumulation of InsP3–InsP5 in the *2mlpa* mutant. The other regulated genes related to photosynthesis, starch and sucrose metabolism, and defense mechanisms may provide additional insights into the nutritional and agronomic performance of *2mlpa* seeds.

## Introduction

Phosphorus (P) is an essential element required for optimal plant growth^1^. Approximately 75% of the P in grains is stored as phytic acid (PA; *myo*-inositol 1,2,3,4,5,6-hexakisphosphate)^2^. Phytic acid is poorly digested by animals and humans, leading to low mineral bioavailability and phosphate pollution of soil and water. These problems may be addressed by the development and cultivation of crops with low PA contents^2,3^.

To minimize the adverse effects of PA, many *low phytic acid* (*lpa*) mutants have been generated via mutagenesis and biotechnological methods in different crops, including soybean (*Glycine max* L. Merr.), rice (*Oryza sativa* L.), barley (*Hordeum vulgare* L.), maize (*Zea mays* L.), and wheat (*Triticum aestivum* L.)^4,5^. Decreases in the PA contents are related to the various types of mutated genes. Previous research revealed that cross and selective breeding of different soybean mutants and pyramiding different gene mutations is an effective strategy for obtaining progeny soybean *lpa* lines that produce seeds with stable and substantial decreases in PA levels. For example, decreases in PA contents ranging from 79 to 88% were observed in double *lpa* soybean mutants with two *IPK1* mutations on chromosomes 6 and 14^6^. More recently, a 63% lowering of the PA content was detected in the progeny resulting from a cross between a rice mutant with a mutation to the *myo*-inositol kinase gene (*OsMIK*) and a rice mutant with a mutated multidrug resistance-associated protein ABC transporter 5 gene (*OsMRP5*)^7^.

The *lpa* soybean lines *Gm-lpa*-TW-1, which has a non-lethal mutation in *MIPS1* (2-bp deletion in the third exon)^8,9^, and *Gm-lpa*-ZC-2, which has a G-A point mutation in *IPK1*, were selected to generate a double *lpa* soybean mutant with mutated *MIPS1* and *IPK1*. An earlier investigation proved *myo*-inositol phosphate synthase (MIPS; EC 5.5.1.4), which is the first and rate-limiting enzyme in the inositol and PA biosynthesis pathways, catalyzes the conversion of glucose 6-phosphate to *myo*-inositol 3-phosphate^10^. The *IPK1* gene encodes an enzyme (EC 2.7.1.158) that converts inositol 1,3,4,5,6-pentakisphosphate (InsP_5_) to PA. An advanced generation recombinant inbred line, *2mlpa* (*mips1/ipk1*), and a sibling line designated 2MWT (*MIPS1/IPK1*) with homozygous non-mutant alleles and a normal PA content were generated. The decrease in the PA content (up to 87%) in the double *lpa* line *2mlpa* was significantly greater than that expected for the single mutants (approximately 50% decrease in phytate contents). The *2mlpa* line accumulated less inositol phosphate isomers (InsP4 and InsP5) than the mutant with only the *IPK1* mutation^11^.

The PA biosynthesis pathway is vital for plant growth^4,5^. Moreover, loss-of-function mutations in *MIPS1* and *IPK1* disrupt the PA biosynthesis pathway, resulting in dramatic decreases in the PA concentration. However, the mechanisms regulating PA levels and downstream gene expression in these *lpa* soybean lines are poorly characterized. Thus, the complete regulatory network associated with PA metabolism must be explored. In this study, mRNA-sequencing (RNA-seq) was used to study the effects of mutations in *MIPS1* and *IPK1* on global changes in the gene expression profiles of developing soybean seeds. The biological processes and pathways revealed by the functional enrichment analyses of differentially expressed genes (DEGs) identified in this study will further clarify the regulation of PA contents in soybean.

## Results and discussion

### Library sequencing and assembly

An advanced generation recombinant inbred line (*2mlpa*) with a low PA content and a sibling line (2MWT) with homozygous non-mutant alleles and a normal PA level were analyzed during five soybean seed developmental stages [7, 12, 17, 22, and 27 days after flowering (DAF)]. To comprehensively characterize the transcriptome changes in *lpa* soybeans, 30 cDNA libraries (three replicates) were sequenced. A total of 195.7 Gb of 150-bp paired-end clean reads were obtained. Each sample yielded up to 6.3 Gb of clean data with a Q20 value exceeding 98%. We mapped 85.71% of the clean reads to the annotated *Glycine_max*_v2.0 soybean reference genome. The number of mapped reads ranged from 34,971,326 to 39,249,412 (Table 1). These results suggest that the data were adequate for subsequent analyses. All raw data were deposited in the NCBI database (accession number PRJNA522338; http://www.ncbi.nlm.nih.gov/bioproject/).

**Table 1:** Summary of the library read analysis.

Sample ID	Total clean reads	Total base pairs	Total mapped reads	Perfect match	Total unmapped reads
2mlpa-1-I	47,376,510	7,106,476,500	39,217,355	29,254,240	8,159,155
2mlpa-1-II	46,147,142	6,922,071,300	38,174,762	28,505,756	7,972,380
2mlpa-1-III	45,752,784	6,862,917,600	37,927,606	28,179,059	7,825,178
2mlpa-2-I	47,766,190	7,164,928,500	38,332,152	28,840,283	9,434,038
2mlpa-2-II	46,019,862	6,902,979,300	36,858,472	27,854,925	9,161,390
2mlpa-2-III	47,627,596	7,144,139,400	37,613,372	28,285,902	10,014,224
2mlpa-3-I	45,237,408	6,785,611,200	36,559,719	27,658,430	8,677,689
2mlpa-3-II	46,939,696	7,040,954,400	38,163,913	28,687,412	8,775,783
2mlpa-3-III	47,094,904	7,064,235,600	39,249,412	29,157,151	7,845,492
2mlpa-4-I	46,624,724	6,993,708,600	38,733,400	28,405,148	7,891,324
2mlpa-4-II	45,328,594	6,799,289,100	37,799,964	27,797,006	7,528,630
2mlpa-4-III	46,884,742	7,032,711,300	37,231,517	28,195,905	9,653,225
2mlpa-5-I	46,770,398	7,015,559,700	38,807,109	29,708,501	7,963,289
2mlpa-5-II	45,679,520	6,851,928,000	36,180,381	26,871,446	9,499,139
2mlpa-5-III	45,845,714	6,876,857,100	37,756,564	28,344,742	8,089,150
2MWT-1-I	47,105,886	7,065,882,900	35,450,056	25,998,470	11,655,830
2MWT-1-II	46,892,294	7,033,844,100	35,547,215	25,864,330	11,345,079
2MWT-1-III	47,828,524	7,174,278,600	35,999,905	26,378,660	11,828,619
2MWT-2-I	47,286,614	7,092,992,100	36,019,630	26,110,062	11,266,984
2MWT-2-II	46,048,510	6,907,276,500	35,423,782	26,086,498	10,624,728
2MWT-2-III	46,622,194	6,993,329,100	34,971,326	25,850,610	11,650,868
2MWT-3-I	47,317,516	7,097,627,400	37,157,999	27,267,786	10,159,517
2MWT-3-II	46,063,542	6,909,531,300	36,372,452	26,833,737	9,691,090
2MWT-3-III	47,549,838	7,132,475,700	37,382,695	27,523,661	10,167,143
2MWT-4-I	45,889,736	6,883,460,400	36,197,074	26,470,608	9,692,662
2MWT-4-II	46,699,880	7,004,982,000	37,777,280	27,945,411	8,922,600
2MWT-4-III	47,302,502	7,095,375,300	37,508,685	27,875,751	9,793,817
2MWT-5-I	47,662,516	7,149,377,400	38,108,064	28,405,856	9,554,452
2MWT-5-II	46,925,520	7,038,828,000	38,110,466	28,209,436	8,815,054
2MWT-5-III	46,929,246	7,039,386,900	36,868,656	27,610,343	10,060,590

### Sequence annotation and DEG analysis

**Figure 1:**
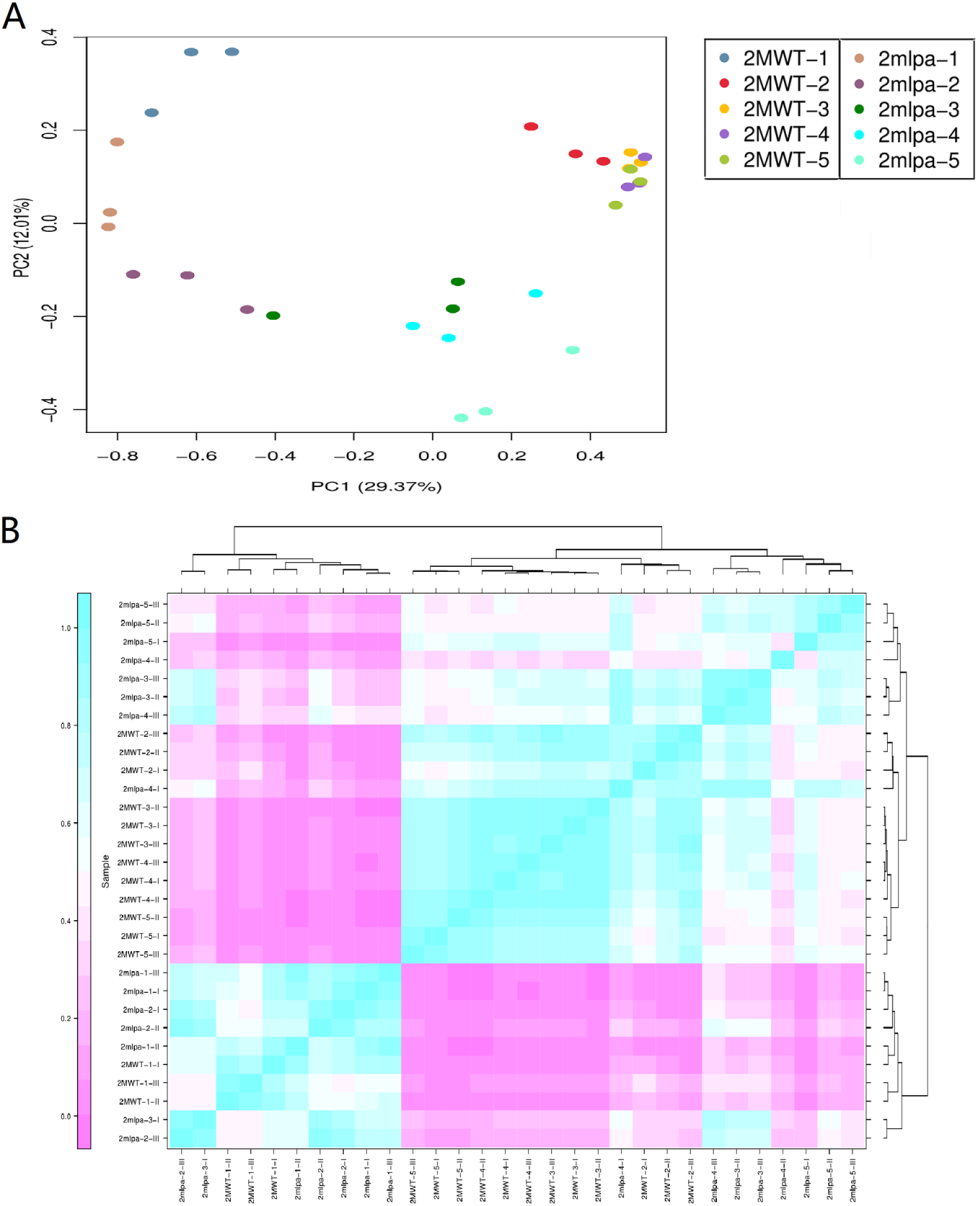
Biological sample variability. (**A**) Principal component analysis plots for 30 *2mlpa* and 2MWT samples. (**B**) Sample clustering heatmap presenting sample-to-sample distances.

**Figure 2:**
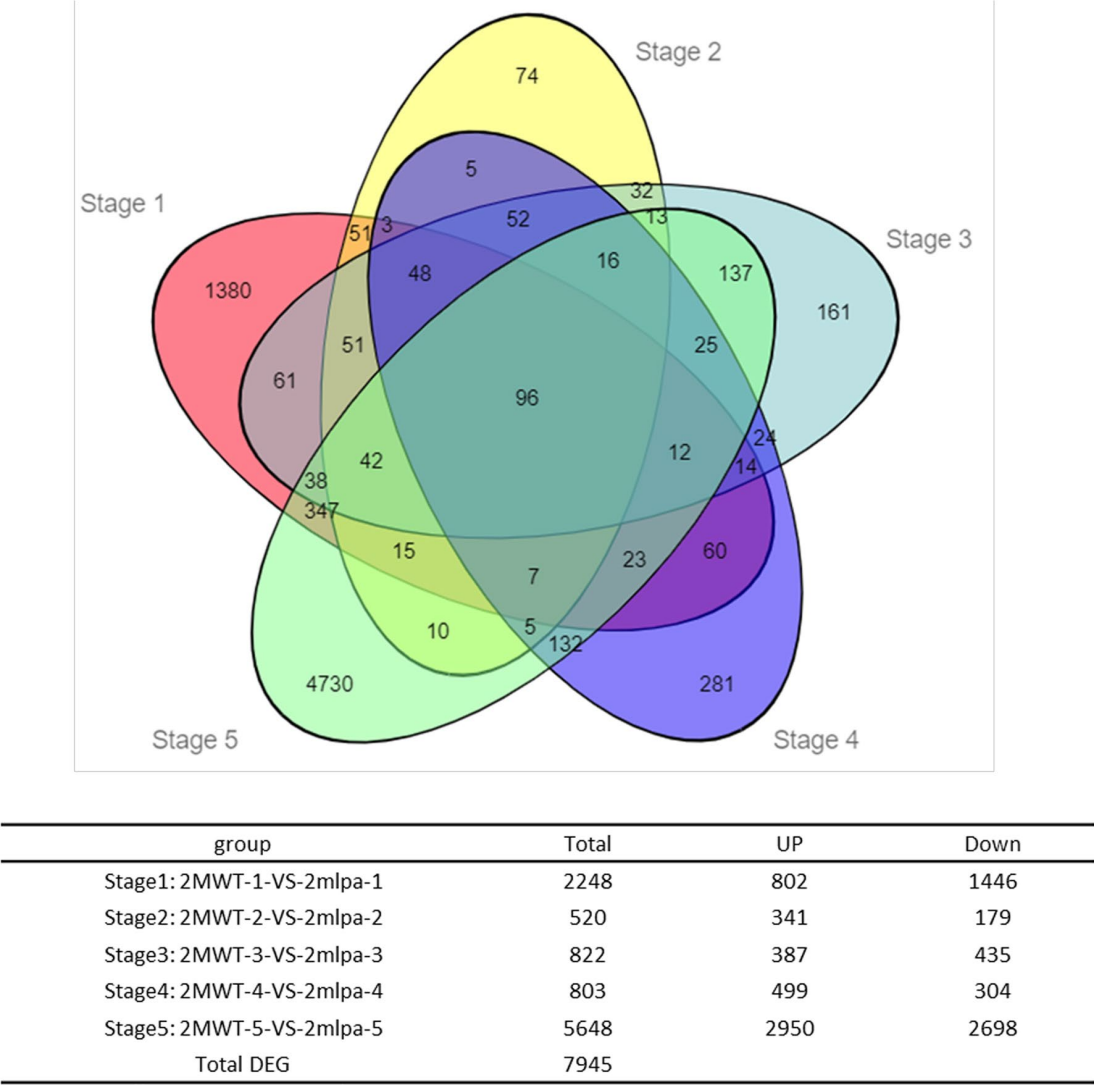
Venn diagram of the co-expressed and uniquely expressed DEGs between *2mlpa* and 2MWT in five seed developmental stages. The number of DEGs between *2mlpa* and 2MWT in each stage is also presented.

The reads per kilobase per million fragments (RPKM) values were used to estimate gene expression levels and conduct DEG analyses. For each mapped soybean gene, its RPKM value was calculated for all 30 libraries. The results of a principal component analysis identified the seed developmental stage and genotype as the major contributors to the data variations (Fig. 1A). Additionally, the biological replicates of each soybean sample clustered together, suggesting there was little variance between replicates. Similarities and differences between individual sample libraries were visualized using a heatmap presenting sample-to-sample distances (Fig. 1B). The *2mlpa*-1, *2mlpa*-2, and 2MWT-1 samples were relatively similar in the early seed developmental stages, whereas *2mlpa*-4, *2mlpa*-5, 2MWT-4, and 2MWT-5 were relatively similar during the late stages. However, there were also differences in gene expression among the examined samples. More specifically, 7,945 genes were significantly differentially expressed between *2mlpa* and 2MWT during the five seed developmental stages (Fig. 2). A Venn diagram analysis was performed using the online OmicShare tools (http://www.omicshare.com/tools). Most of the DEGs were expressed during the early (stage 1) and late (stage 5) seed developmental stages. Moreover, 1380 DEGs were only differentially expressed in stage 1, whereas 4730 DEGs were only differentially expressed in stage 5. Of the identified DEGs, 96 were differentially expressed in all five seed developmental stages.

### Validation of DEGs through reverse-transcription quantitative PCR (RT-qPCR)

**Figure 3:**
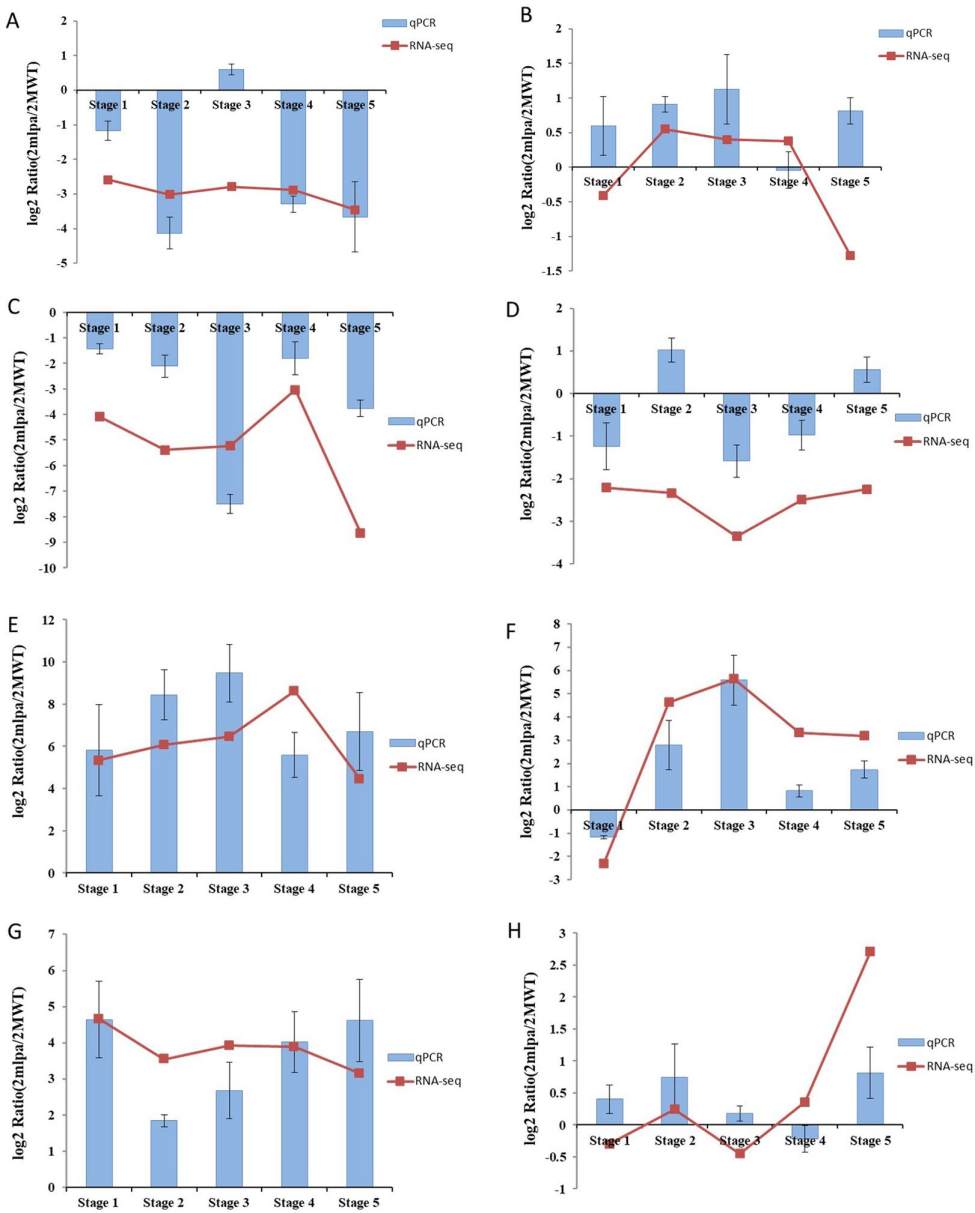
Verification of the RNA-seq results by RT-qPCR. The y-axis represents the log_2_(ratio) (*2mlpa*/2MWT) for (**A**) Glyma.11G238800, (**B**) Glyma.14G072200, (**C**) Glyma.16G065700, (**D**) Glyma.12G210600, (**E**) Glyma.08G109300, (**F**) Glyma.12G232500, (**G**) Glyma.16G214900, and (**H**) Glyma.09G073600 in five seed developmental stages.

Eight randomly selected DEGs associated with biological processes were analyzed by RT-qPCR (Fig. 3). In most cases, the gene expression trends revealed by RT-qPCR were similar to those detected by the RNA-seq analysis, although the fold-changes varied. The RNA-seq and RT-qPCR expression profiles were similar for Glyma.16G065700 (Fig. 3C), Glyma.08G109300 (Fig. 3E), Glyma.12G232500 (Fig. 3F), and Glyma.16G214900 (Fig. 3G). However, in contrast to the corresponding RT-qPCR expression profiles, the RNA-seq analysis revealed the down-regulated expression of the following genes during specific seed developmental stages: Glyma.11G238800 (Fig. 3A) in stage 3, Glyma.14G072200 (Fig. 3B) in stage 5, Glyma.12G210600 (Fig. 3D) in stage 2, and Glyma.09G073600 (Fig. 3H) in stage 3. These results suggest the RT-qPCR data were relatively consistent with the RNA-seq data for 8 of the DEGs.

### Functional enrichment analyses of DEGs

We annotated 4,980 genes that were differentially expressed between *2mlpa* and 2MWT (62.67% of all DEGs) during the five seed developmental stages with at least one Gene Ontology (GO) term. The GO terms included 345 associated with biological processes (Supplementary Table S1). Some terms were included in more than one stage, whereas others were exclusive to one stage. For example, establishment of localization (23% of DEGs in stage 1), single-organism metabolic process (13.39% of DEGs in stage 1), and localization (8.05% of DEGs in stage 1) were specific to early seed developmental stages (stages 1 and 2). In contrast, organic substance metabolic process (28.95% of DEGs in stage 5), macromolecule metabolic process (21.23% of DEGs in stage 5), and response to abiotic stimulus (4.44% of DEGs in stage 5) were exclusive to the late seed developmental stage (stage 5).

The Kyoto Encyclopedia of Genes and Genomes (KEGG) pathway enrichment analysis is useful for elucidating the significantly enriched metabolic and signal transduction pathways among DEGs as well as the associated biological functions^12^. A total of 3316 DEGs (41.74% of all DEGs) between the 2*mlpa* and 2MWT lines in five seed developmental stages were involved in 128 metabolic and signal transduction pathways (Supplementary Table S2). The highly represented pathways included metabolic pathways, plant–pathogen interaction, plant hormone signal transduction, photosynthesis, flavonoid biosynthesis, phenylpropanoid biosynthesis, and starch and sucrose metabolism (Table 2). The DEGs assigned to KEGG pathways varied among the seed developmental stages. For example, none of the DEGs in stages 2 and 3 were associated with KEGG pathway (ko00196), whereas 22 DEGs in stage 5 were.

**Table 2:** Number of DEGs in enriched KEGG pathways in five seed developmental stages.

Pathway	Pathway ID	Stage 1	*P* value	Stage 2	*P* value	Stage 3	*P* value	Stage 4	*P* value	Stage 5	*P* value
Metabolic pathways	ko01100	361	1.1 E−12	108	4.2E−11	153	3.9 E−09	138	7.4 E−06	794	1.8 E−02
Biosynthesis of secondary metabolites	ko01110	210	1.9 E−08	42	8.1 E−03	66	8.6 E−03	65	9.3 E−02	374	9.9 E−03
Plant–pathogen interaction	ko04626	109	3.9 E−02	26	4.2 E−02	54	2.0 E−02	39	6.1 E−02	303	5.6 E−02
Plant hormone signal transduction	ko04075	67	9.9 E−03	12	9.9 E−01	27	9.0 E−03	38	2.1 E−01	252	4.6 E−66
Ribosome	ko03010	27	6.0 E−03	18	1.1 E−04	22	1.3 E−03	15	1.1 E−01	252	4.9 E−02
Protein processing in endoplasmic reticulum	ko04141	24	9.0 E−03	2	9.9 E−03	14	2.8 E−02	5	9.9 E−03	113	2.3 E−03
Purine metabolism	ko00230	37	3.3 E−02	11	5.2 E−02	19	8.8 E−03	12	3.3 E−02	104	1.1 E−03
Pyrimidine metabolism	ko00240	39	8.7 E−03	9	1.6 E−02	19	6.3 E−03	12	2.9 E−01	100	1.8 E−03
Starch and sucrose metabolism	ko00500	30	4.8 E−02	5	8.2 E−03	14	2.4 E−02	14	2.3 E−01	75	8.8 E−02
Phenylpropanoid biosynthesis	ko00940	42	2.5 E−03	9	1.7 E−02	14	1.5 E−01	14	1.4 E−02	74	6.3 E−02
Photosynthesis	ko00195	24	1.2 E−08	33	2.2 E−35	32	7.3 E−27	28	4.1 E−22	69	3.5 E−25
Flavonoid biosynthesis	ko00941	45	3.3 E−08	18	3.4 E−07	14	1.2 E−02	12	4.8 E−02	67	9.4 E−07
Oxidative phosphorylation	ko00190	25	1.3 E−03	30	3.0 E−21	29	2.6 E−14	7	2.2 E−01	67	2.3 E−02
Photosynthesis-antenna proteins	ko00196	1	8.0 E−03	0		0		5	3.0 E−04	22	1.8 E−11

The GO and KEGG pathway functional enrichment analyses indicated that the enriched DEGs were mainly involved in PA metabolism, photosynthesis, starch and sucrose metabolism, and defense mechanisms. These findings were somewhat consistent with those of earlier investigations on other plants, including *Arabidopsis thaliana* (Arabidopsis)^13,14^, rice^15^, poplar^16^, and sweet potato^17^. Moreover, the experimental design and results of a study by Redekar et al.^18^ were similar to those of this study. However, Redekar et al. analyzed a triple *lpa* mutant (*mips1/mrp-l/mrp-n*), but our investigation was completed with *2mlpa* (*mips1/ipk1*). Thus, the *mips1* mutation was common to both studies, whereas the other mutations differed (*mrp-l/mrp-n* vs *ipk1*). Both IPK1 and MRP function at very similar stages: the last step of the PA biosynthesis pathway (IPK1) as well as immediately after PA is synthesized, when PA is transported to the vacuole (MRP). Many of the DEGs revealed by the two studies are involved in similar pathways (e.g., photosynthesis and defense), which might be related to the common mutation (*mips1*) in the analyzed plants. Some of the DEGs are involved in different pathways, including glucan metabolism and apoptosis in the study by Redekar et al., which might be associated with the different mutations in the examined plants. The two studies are further compared in the following sections.

Low phytate contents often have undesirable effects on seed development and germination potential^13,14,16^. We observed that the *2mlpa* plants grew and developed more poorly (lower emergence, thinner stems, and greater susceptibility to soybean mosaic virus) than the 2MWT plants under field conditions. This is consistent with the results of previous studies^13,14,16^ and is likely related to the biological processes discussed in the following section.

### DEGs involved in PA metabolism

**Figure 4:**
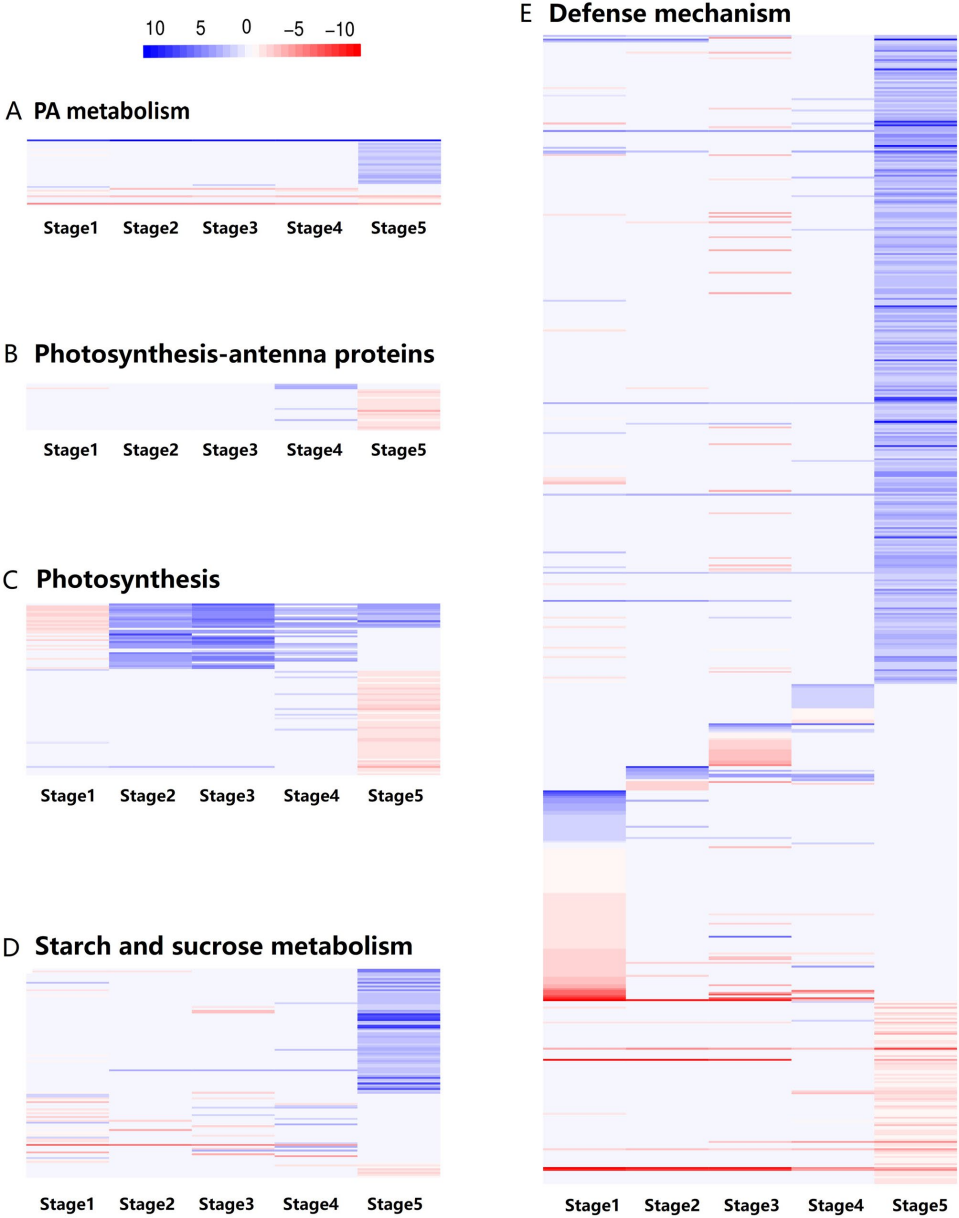
Fold-change (*2mlpa* over 2MWT) ratios in five seed developmental stages for the DEGs associated with (**A**) PA metabolism, (**B**) photosynthesis-antenna proteins, (**C**) photosynthesis, (**D**) starch and sucrose metabolism, and (**E**) defense mechanisms. Rows represent DEGs between the *2mlpa* and 2MWT soybean lines, whereas columns represent the five seed developmental stages. Blue and red indicate up-regulated and down-regulated expression (*2mlpa* over 2MWT), respectively.

The PA biosynthesis pathway is critical for plant growth. Plants possess two parallel PA biosynthesis pathways, with both starting with the production of inositol 3-phosphate (InsP3)^19^. A total of 36 genes involved in PA metabolism and inositol phosphate metabolism (ko00562) were differentially expressed between *2mlpa* and 2MWT by more than twofold in the late seed developmental stage (stage 5) (Supplementary Table S3, Fig. 4A). One of the mutated genes in *2mlpa* was *MIPS1* (Glyma.11G238800), and its expression was down-regulated in *2mlpa* throughout the seed developmental period (Table 3). The *2mlpa* plants were derived from a cross between *Gm-lpa*-TW-1 (mutated *MIPS1*) and *Gm-lpa*-ZC-2 (mutated *IPK1*). We previously determined that *MIPS1* is expressed at low levels in developing *Gm-lpa*-TW-1 seeds^20^. Accordingly, cross-breeding with *Gm-lpa*-ZC-2 did not alter *MIPS1* expression. Soybean includes three *MIPS1* orthologs (Glyma.18G018600, Glyma.05G180600, and Glyma.08G138200)^21,22^. Interestingly, Glyma.18G018600 and Glyma.05G180600 expression levels were up-regulated in *2mlpa* in the late seed developmental stage (Table 3). In a previous study, we observed that *MIPS1* expression increased as seed organs developed in *Gm-lpa*-ZC-2 and the wild-type parents of *Gm-lpa*-TW-1 and *Gm-lpa*-ZC-2, peaking at 22 DAF^20^. It seems that the up-regulated expression of Glyma.18G018600 and Glyma.05G180600 in *2mlpa* compensates for a mutated *MIPS1* (Glyma.11G238800) to enhance *MIPS* expression in the late seed developmental stage. We hypothesized that these DEGs encoding MIPS are functionally redundant.

**Table 3:** Differential expression of *MIPS1* and *IPK1* orthologs in five seed developmental stages.

Gene ID	log2Ratio(2mlpa-1/2MWT-1)	log2Ratio(2mlpa-2/2MWT-2)	log2Ratio(2mlpa-3/2MWT-3)	log2Ratio(2mlpa-4/2MWT-4)	log2Ratio(2mlpa-5/2MWT-5)
Glyma.14G078800	–	–	–	–	–
Glyma.04G032400	–	–	–	–	–
Glyma.06G033100	–	–	–	–	–
Glyma.11G238800	−2.59117	− 3.01574	− 2.78554	− 2.88699	− 3.45853
P-value	2.28E−23	4.24E−13	2.93E−18	2.17E−14	2.19E−18
Glyma.18G018600	− 1.30207	–	–	–	1.765128
P-value	2.09E−10	–	–	–	0.00743
Glyma.05G180600	–	–	–	–	2.68211
P-value	–	–	–	–	6.06E–07
Glyma.08G138200	–	–	–	–	–

Several studies confirmed that MIPS regulates pathways influencing seed development and plant growth^9,23,24^. For example, in Arabidopsis, the disruption of MIPS severely decreases the inositol and PA levels, resulting in smaller plants, programmed cell death, or hypersensitivity to high-intensity light stress^25–27^. In an earlier study, the *Gm-lpa*-TW-1 mutant exhibited acceptable agronomic and nutritional traits, with the exception of poor field emergence. In this study, the field emergence of *2mlpa* was relatively low, but it was substantially better than that of *Gm-lpa*-TW-1. Hence, the *2mlpa* seed traits may improve following a hybridization with *Gm-lpa*-ZC-2, which exhibits normal field emergence^8^.

The other mutated gene was *IPK1* (Glyma.14g07880), which encodes the enzyme (InsP5 2-kinase) that catalyzes the final step of the PA biosynthesis pathway^15^. The expression of this gene was unaffected in *2mlpa*, which is consistent with the RT-qPCR results for *Gm-lpa*-ZC-2. There are reportedly no obvious differences in the relative expression levels of *IPK1* (Glyma.14g07880) between *Gm-lpa*-ZC-2 and its wild-type parent^28^. We determined that the single G-A point mutation in *Gm-lpa*-ZC-2 results in a deletion of 37 amino acids that disrupts an important motif. Thus, the mutation does not affect *IPK1* expression, but it alters the functionality of the encoded enzyme^28^. This may explain the lack of significant changes in *IPK1* expression in *Gm-lpa*-ZC-2 and *2mlpa*. Moreover, *IPK1* expression in *2mlpa* might not be affected by cross-breeding. The expression of *IPK1* orthologs (Glyma.04G032400 and Glyma.06G033100) was also not significantly affected in *2mlpa* seeds (Table 3), in contrast to the significant up-regulated expression of Glyma.06G033100 in *Gm-lpa*-ZC-2 seeds at 15 DAF^28^. This difference may be related to a series of chain reactions induced by the *MIPS1* mutation. Alternatively, it may be due to the low *IPK1* ortholog expression levels revealed by the transcriptome analysis.

In rice, RNAi-mediated seed-specific silencing of *IPK1* significantly decreases phytate levels and increases inorganic phosphate contents^15^. Although the constitutive suppression of the enzymes involved in phytate biosynthesis may be detrimental to plant growth and development^23,29–32^, we did not find any defects induced by the *IPK1* mutation in *Gm-lpa-*ZC-2. Furthermore, some of the traits of *Gm-lpa*-TW-1 with a single mutation to *MIPS1* were improved by the hybridization with *Gm-lpa*-ZC2.

We also observed that the expression levels of some inositol polyphosphate kinase and phosphatase genes involved in PA metabolism were up-regulated only at the late seed developmental stage (stage 5). These genes included two *ITPK1* genes (inositol-1,3,4-trisphosphate 5/6-kinase), two *PI4KA* genes (phosphatidylinositol 4-kinase A), and five *INPP5B/F* genes (inositol polyphosphate 5-phosphatase). These results imply that the mutations in *2mlpa* mainly affect PA biosynthesis in the late seed developmental stage and induce complex chain reactions related to PA metabolism. An earlier investigation indicated that *Gm*-*lpa*-ZC-2 (one of the parents of *2mlpa*) and *2mlpa* mutants have significantly more inositol triphosphate (InsP3) and inositol tetraphosphate (InsP4) than the wild-type control^33^. Both InsP3 and InsP4 are intermediary metabolites in PA metabolism. The up-regulated expression of the DEGs encoding inositol polyphosphate kinases and phosphatases is consistent with the increase in InsP3 and InsP4 in *lpa* lines. The effects of a single mutated gene on the expression of these PA metabolism-related genes remain to be characterized.

### DEGs involved in photosynthesis in the late seed developmental stage

Photosynthesis and photosynthesis-antenna protein (ko00195 and ko00196) pathways were enriched across the five *2mlpa* seed developmental stages, but especially in stage 5 (Supplementary Table S4). A total of 117 DEGs were associated with photosynthesis and photosynthesis-antenna proteins. The expression levels of most of the photosynthesis-related DEGs (81 of 94 DEGs) in stage 5 were lower in *2mlpa* than in 2MWT (Fig. 4B,C). These DEGs encode different subunits of photosystems I and II, the cytochrome b6/f complex, the photosynthetic electron transport system, F-type ATPase, and the light-harvesting chlorophyll protein complex (LHC). The down-regulated expression of genes encoding the photosystem complex subunits may lead to decreased photosynthetic activities in *2mlpa* mutant plants, similar to what has been observed in other *lpa* soybean mutants^18,34^. The initial reaction of the *myo*-inositol biosynthesis pathway is catalyzed by MIPS. The subsequent dephosphorylation of L-*myo*-inositol 1-phosphate results in the production of *myo*-inositol. This compound helps maintain the photosynthesis in *Mesembryanthemum crystallinum*, which is consistent with the observed changes in *myo*-inositol contents and the down-regulated expression of photosynthesis-related DEGs in *2mlpa*^33,34^*.*

The photosynthesis-antenna protein pathway modulates plant defense responses induced by abiotic stimuli (e.g., light and temperature)^15,35,36^. The LHC affects the photosynthesis-antenna protein pathway. The chief function of the LHC is to collect and transfer light energy to photosynthetic reaction centers^36^. In this study, LHC-associated DEGs were significantly enriched in the late soybean seed developmental stage (stage 5). The expression levels of 16 DEGs encoding chlorophyll a/b-binding proteins related to 9 of 11 LHC subunits were down-regulated only in the late seed developmental stage (stage 5) in *2mlpa* (Fig. 4B). Such diminished expression may inhibit light harvesting and energy production. These results suggest the mutations in *2mlpa* modulate the photosynthesis-antenna protein pathway during the late seed developmental stage. Furthermore, light provides energy for photosynthesis, while also serving as a signal that influences plant growth and development. Arabidopsis *mips1* mutants undergo light intensity-dependent cell death^26,37^. We observed that most of the photosynthesis-related DEGs had down-regulated expression levels in *2mlpa* in stage 5, in accordance with the results of previous research. In a study by Redekar et al. (2015), photosynthesis (GO:0015979) was an enriched GO term in the late seed developmental period (stages 3–5), and the expression of most of the photosynthesis-related DEGs in these stages was down-regulated in the *3mlpa* (*mips1/mrp-l/mrp-n*) mutant^18^. These findings imply that a mutation in *MIPS1* or the lack of PA may decrease photosynthesis during the late seed developmental stages. The down-regulated expression of the genes involved in photosynthesis might contribute to some of the unfavorable traits in *lpa* mutants (e.g., decreased field emergence).

### DEGs involved in starch and sucrose metabolism

In higher plants, sucrose is the main carbohydrate that is transported to provide the carbon and energy needed for growth and the synthesis of storage reserves^38^. Furthermore, sucrose is a signaling molecule affecting plant developmental processes, including growth, defense, and the regulation of flowering and the maturation of storage organs^3,38,39^. The *2mlpa* soybean mutant exhibits a high-sucrose phenotype. In the current study, 67 DEGs related to starch and sucrose metabolism (ko00500) were more highly expressed in *2mlpa* than in 2MWT almost exclusively in the late seed developmental stage (Fig. 4D, Supplementary Table S5). These DEGs mainly encode key enzymes involved in starch biosynthesis and degradation, sucrose metabolism, sugar and starch interconversion, and transport. The expression of a series of genes associated with starch and sucrose metabolism was altered in *2mlpa*. Additionally, *myo*-inositol is an important intermediate in pathways mediating PA biosynthesis and sucrose metabolism. It also helps facilitate the reversible addition of galactose units from galactinol to sucrose during raffinosaccharide biosynthesis^40^. Variations in *myo*-inositol levels may inhibit raffinosaccharide biosynthesis, thereby increasing sucrose levels^39,40^. A mutation in *MIPS1* decreases the PA level, induces a series of changes in the intermediate metabolites of the PA biosynthesis pathway (e.g., *myo*-inositol), and concomitantly decreases the raffinosaccharide content, which may increase the sucrose level in *Gm*-*lpa*-TW-1^39^. The mutation in *MIPS1* in *2mlpa* might be responsible for the observed increased sucrose content and the up-regulated expression of 67 starch and sucrose metabolism (ko00500)-related DEGs. However, none of the DEGs in *2mlpa* were involved in the raffinose family oligosaccharide pathway. The *Gm-lpa*-TW75-1 mutant, which is one of the parents of *2mlpa*, has lower raffinose contents (74.2% to 84.3% lower) than the wild-type cultivar Taiwan 75^39^. The *mips* mutation reportedly down-regulates the expression of genes in the raffinose family oligosaccharide pathway^25,37,41,42^. Redekar et al.^18^ observed the same phenomenon in *3mlpa* (*mips1/mrp-l/mrp-n*) soybean seeds. Thus, we speculated that mutations in *IPK1* and *MIPS1* gene have a complementary role affect in the raffinose family oligosaccharide pathway while the mutation in *MIPS1* gene down-regulates the raffinose family oligosaccharide pathway.

Cell wall synthesis is modulated in soybean *lpa* mutants^18^. β-Glucosidase is a cell wall-associated enzyme^43,44^. In Arabidopsis leaves, β-glucosidase may play a vital role in the breakdown of cell wall polysaccharides, which provides the soluble sugars for remobilization and the completion of energy-dependent senescence^45^. In this study, the expression levels of 13 DEGs encoding β-glucosidase were higher in *2mlpa* than in 2MWT in the late seed developmental stage. Interestingly, decreased photosynthesis is accompanied by a significant increase in the activity of cell wall-bound β-glucosidase in Arabidopsis^45^. This is consistent with the down-regulated expression of DEGs related to photosynthesis and the up-regulated expression of DEGs encoding β-glucosidase in *2mlpa* soybean seeds. A precise characterization of the β-glucosidase function in the *2mlpa* mutant is beyond the scope of this study; however, the mutations in *2mlpa* may enhance the degradation of cell wall polysaccharides.

The DEGs directly associated with sucrose biosynthesis encoded the following two enzymes: sucrose-phosphate synthase (SPS; EC 2.4.1.14), which catalyzes the biosynthesis of sucrose 6-phosphate from UDP-glucose and fructose 6-phosphate, and sucrose synthase (SuSy; EC 2.4.1.13)^46–49^. We identified one up-regulated DEG (Glyma.13G161600) encoding SPS and two up-regulated DEGs (Glyma.09G073600 and Glyma.15G182600) encoding SuSy in *2mlpa* in the late seed developmental stage (stage 5). These up-regulated DEGs may contribute to the high sucrose content of *2mlpa* soybean seeds. Another *lpa* soybean line, V99-5089 (mutation in *MIPS1*), also produces seeds with low PA and high sucrose contents^50^. A mutation in *MIPS1* or low PA levels may increase the sucrose content of soybean seeds, which is consistent with the high-sucrose phenotype of *lpa* mutant seeds^39^.

### DEGs involved in defense mechanisms

The DEGs associated with the plant–pathogen interaction (ko04626) pathway and annotated with the response to stress (GO:0006950) GO term were enriched in *2mlpa* in the five seed developmental stages, but especially in stages 1 and 5. A total of 604 DEGs were related to defense mechanisms, and the expression levels of most of these genes were exclusively regulated in one stage (Fig. 4E, Supplementary Table S6). For example, most of the DEGs in the early seed developmental stages (105 of 146 DEGs) were not differentially expressed in the late seed developmental stage (stage 5). Moreover, most of the DEGs in stage 5 (366 of 447 DEGs) were differentially expressed exclusively in this stage. These results suggest that the genes affected by the *lpa* mutations differ depending on the seed developmental stage.

Phytic acid is a natural antioxidant^4,51^. In plants, PA is also a key signaling molecule generated in response to the drought stress-related hormone abscisic acid, which stimulates Ca^2+^ release in guard cells^4,5,52^. Furthermore, PA is important for maintaining the basal resistance to plant pathogens^4,5,53^. Earlier research confirmed that InsP3 and InsP4 are intermediate metabolites in PA metabolism that act as second messengers during the regulation of cytosolic Ca^2+^ levels^54^. Hence, the decrease in the PA level and the increase in InsP3 and InsP4 contents in *lpa* soybean may induce a series of complex chain reactions influencing defense mechanisms^33,39^. In the current study, 604 DEGs were related to defense mechanisms, with about a third of these genes encoding plant disease resistance (R) proteins, including leucine-rich repeat (LRR) receptor-like serine/threonine-protein kinase, calmodulin, transcription factors, peroxidases, and mitogen-activated proteins kinases (MAPKs).

Plant R proteins are important components underlying the stress resistance mechanism, and several *R* genes confer resistance to diverse plant pathogens^55^. In this study, 64 *R* genes were more highly expressed in *2mlpa* than in 2MWT in the late seed developmental stage. The most prevalent domain among the characterized R proteins is the LRR, which is the major pathogen recognition determinant. The LRR receptor-like R protein comprises an extracellular LRR domain and a transmembrane motif^56^. On the basis of the data generated in this study, the expression of 21 DEGs encoding the LRR receptor-like serine/threonine protein kinase FLS2 was up-regulated in the late seed developmental stage. In Arabidopsis, FLS2 is a transmembrane receptor kinase that activates antimicrobial defense responses^57^. Brassinosteroid insensitive 1-associated receptor kinase 1 (BAK1) is also an LRR receptor-like protein kinase and is involved in the signaling by FLS2^58^. Plants in which *BAK1* is mutated exhibit inhibited responses to various microbial elicitors and cold shock proteins^58,59^. In this study, the expression levels of 28 DEGs encoding BAK1 were up-regulated in *2mlpa* in stage 5. The role of LRR-encoding genes during *2mlpa* soybean seed development remains unknown, and additional studies are required to confirm the effects of the up-regulated expression of LRR genes on the *2mlpa* soybean line. Similar results were reported by Redekar et al.^18^. Defense-related DEGs were enriched in the *3mlpa* (*mips1/mrp-l/mrp-n*) soybean seeds, and some of the DEGs encoded LRRs or LRR-containing disease resistance proteins. However, Redekar et al. reported that apoptosis (GO:0006915) is an enriched GO term in the early seed developmental stages, which was in contrast to our results for *2mlpa*. These findings are apparently associated with the *IPK1* mutation in *2mlpa* or the *MRP* mutations in *3mlpa*^18^.

Some genes encoding NAC, MYC2, and WRKY transcription factors that regulate defense-related genes were also differentially expressed at seed developmental stage 5 in the *2mlpa* mutant, including two WRKY70-encoding DEGs, which were up-regulated. In Arabidopsis, WRKY70 negatively modulates cell wall-associated defenses against pathogens and leaf senescence^60–62^. The MAPKs are key enzymes mediating adaptive responses to various abiotic and biotic stresses^63^. In the current study, 10 DEGs encoding MAPKs were differentially regulated between *2mlpa* and 2MWT in the five examined seed stages. These defense-related DEGs may be affected by changes in the abundance of PA or its intermediary metabolites. Interactions between plants and pathogens involve bidirectional recognition. In response to a pathogen infection, plant defense mechanisms modulate the expression of many genes^64^. Our functional characterization revealed 418 DEGs involved in plant–pathogen interactions in *2mlpa* soybean seeds, suggesting that *MIPS1*- and *IPK1*-induced processes share some common features with the defense responses to pathogens in plants. Overall, our results verify the correlation between PA and defense mechanisms.

## Conclusions

In this study, an analysis of the *2mlpa* mutant line revealed low PA contents as well as changes in multiple primary and secondary metabolites. We used a transcriptomics approach to compare gene expression profiles in five soybean seed developmental stages in a *lpa* soybean line (*2mlpa*) carrying two mutations (*mips1* and *ipk1*) and in a non-mutant line (2MWT). A total of 7,945 genes were identified as differentially expressed between *2mlpa* and 2MWT across the five seed developmental stages, with DEGs mainly related to 128 KEGG pathways (e.g., metabolic pathways, biosynthesis of secondary metabolites, starch and sucrose metabolism, photosynthesis, flavonoid biosynthesis, and photosynthesis-antenna proteins). The results presented herein have clarified the regulation of these metabolic events in *2mlpa*. We also identified multiple DEGs involved in defense mechanisms, suggesting that *MIPS1*- and *IPK1*-induced processes and defense responses to abiotic and biotic stresses share common features in plants. On the basis of a comparison between the results of this study and those of an earlier investigation by Redekar et al.^18^, we conclude that the mutation in *IPK1* compensates for the mutation in *MIPS1* in the raffinose family oligosaccharide pathway. Furthermore, the mutation in *MIPS1* is a major factor adversely affecting photosynthesis. The findings of the current study further clarify the metabolic regulatory network of the *2mlpa* mutant during seed development.

## Methods

### Genetic materials and backgrounds

**Figure 5:**
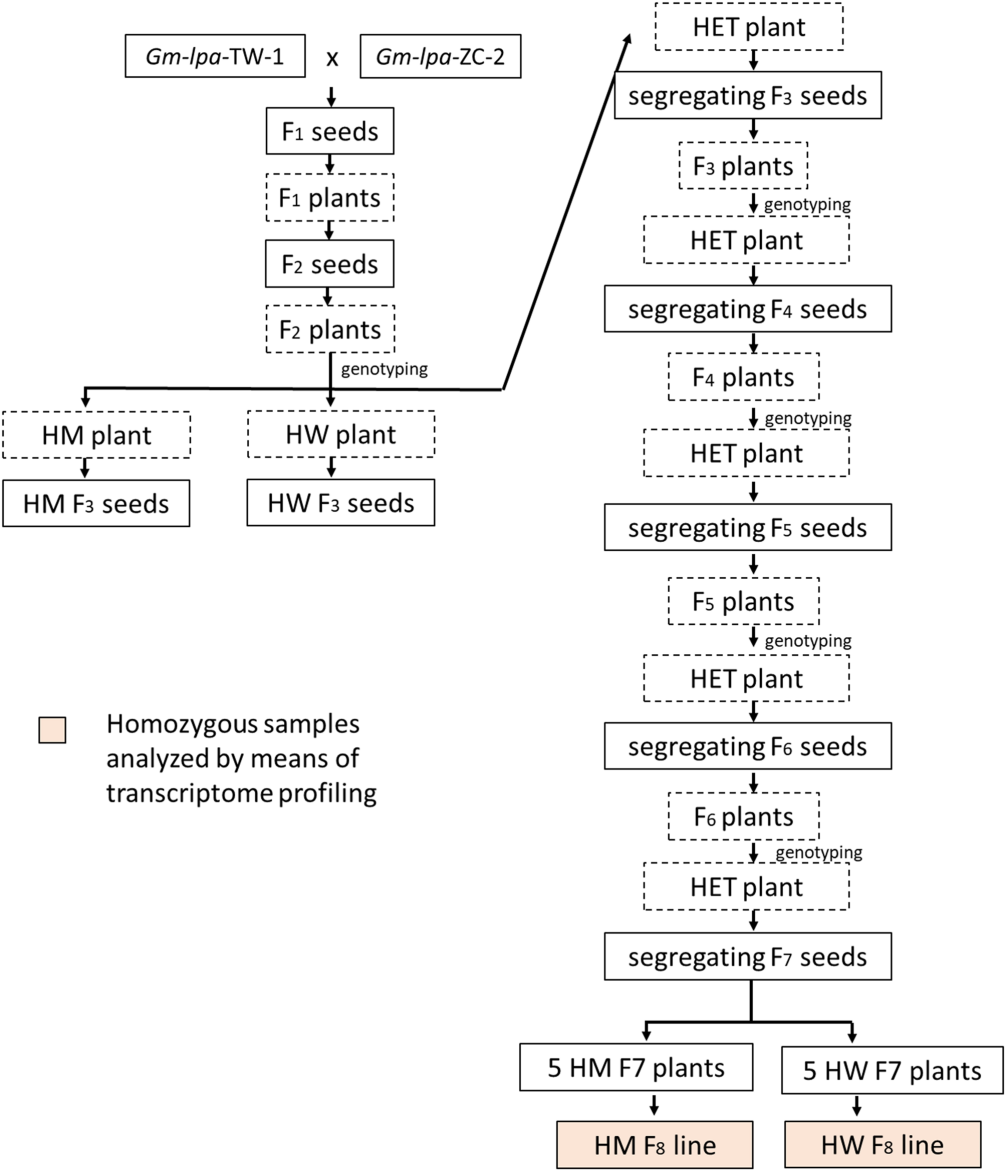
Flowchart of soybean material production via the cross-breeding of Gm-*lpa*-TW-1 and Gm-*lpa*-ZC-2. *HM* homozygous *lpa*-mutant, *HW* homozygous wild-type, *HET* heterozygous.

A homozygous double-mutant soybean line with low PA contents, designated *2mlpa* (*mips1/ipk1*), and a sibling line with homozygous non-mutant alleles and normal PA levels, designated 2MWT (MIPS1/IPK1), were produced in the present study. These lines were developed by crossing *Gm-lpa-*TW-1 with *Gm-lpa-*ZC-2. *Gm-lpa-*TW-1 was developed earlier via the gamma irradiation of Taiwan 75 (a soybean cultivar widely grown in Zhejiang province, China), resulting in a 2-bp deletion in *GmMIPS1*^8^. A comparison with the commercial wild-type cultivar Zhechun No. 3 detected a G-A point mutation in *GmIPK1* in the *Gm-lpa-*ZC-2 soybean line^28^. The F_1_ plants generated by the cross were grown and harvested individually. The F_2_ plants (as well as the original parents) were grown and genotyped on the basis of a high inorganic P test and a high-resolution melting curve analysis^8,28^. Additionally, the F_2_ plants were classified as homozygous wild-type (HWT), homozygous *lpa* mutant (HM), or heterozygous plants. The F_3_ seeds from five heterozygous F_2_ individuals were sown and the resulting plants were genotyped to identify heterozygous F_3_ plants. The F_4_ seeds from five heterozygous F_3_ plants were sown to produce the F_4_ population, which was examined to detect homozygous wild-type and *lpa* mutant plants (Fig. 5). The method used to select F_5_–F_6_ plants was similar to that used for identifying F_3_–F_4_ plants. The F_7_ population was developed from five heterozygous F_6_ plants and segregated, after which their genotypes were determined. The HWT and HM seeds from 10 F_7_ plants were used to produce five HWT and five HM F_8_ lines, which were subsequently analyzed. The genotypes of the F_8_ plants were determined following a high-resolution melting curve analysis, after which the *lpa* and non-*lpa* lines were selected^65^. The seed PA and inositol phosphate isomer contents of the F_8_ lines were measured, which revealed the PA contents of the *2mlpa* and 2MWT lines were 2.32 mg/g and 18.2 mg/g, respectively^11^.

### Plant growth and RNA extraction

For each of the experimental F_8_ lines (*2mlpa* and 2MWT), 50 plants were grown in a greenhouse at 25 °C with a 14-h light/10-h dark photoperiod. Seed length was used as the criterion for defining the following five soybean seed developmental stages: stage 1, 7 DAF; stage 2, 12 DAF; stage 3, 17 DAF; stage 4, 22 DAF; and stage 5, 27 DAF^28,39^. For each stage, seeds were collected from 50 plants and mixed to produce one sample. Three parallel samples collected in each stage served as biological replicates for the RNA extraction. The collected seeds were immediately ground to a fine powder, from which total RNA was extracted using the E.Z.N.A. Plant RNA kit (Omega Bio-tek, Inc., USA). Genomic DNA contaminants were eliminated using RQ1 RNase-Free DNase (Promega, USA).

### Library construction and sequencing

The quality of the extracted total RNA was assessed using the 2100 Bioanalyzer (Agilent, Santa Clara, CA, USA) as well as by agarose gel electrophoresis. The concentrations of all RNA samples were determined and then adjusted to 500 ng/μl. High-quality RNA samples were used to construct sequencing libraries. A total of 30 cDNA libraries were generated using the SMART™ cDNA Library Construction Kit (Takara Bio-tek, Inc., Japan) and tested using the Agilent 2100 Bioanalyzer and the ABI StepOnePlus Real-Time PCR System (Thermo Fisher, USA). The libraries were then sequenced using the Illumina HiSeq 4000 platform (Illumina, USA) at iGENE Biological Technology (Hangzhou, China).

### Sequence assembly

The raw sequencing reads were filtered by removing low-quality sequences and rRNA reads. The clean reads were assembled using SOAPaligner/SOAP2^66^. The TopHat^67^ program (http://ccb.jhu.edu/software/tophat/index.shtml) was used to align the clean reads for each sample with the reference genome sequence (*Glycine_max_*v2.0). Mismatches of no more than five bases were allowed for the alignment.

### Gene annotation and analysis of DEGs

All genes were analyzed by aligning them with sequences in the NR (NCBI non-redundant protein), Swiss-Prot, GO, COG (Clusters of Orthologous Groups), and KEGG databases using BLAST2GO (https://www.blast2go.com/).

Gene expression levels were calculated and normalized as RPKM values. The RPKM method can eliminate the influence of gene length and sequence quantity on gene expression data. The calculated gene expression levels can be used directly to reveal differences in gene expression between samples. Differentially expressed genes were analyzed using the DESeq function of the Bioconductor software (http://www.bioconductor.org). The false discovery rate (FDR) was determined by correcting the *P* value according to the Benjamini–Hochberg method^68^. The criteria used for identifying significant DEGs were as follows: FDR ≤ 0.05 and |log_2_(ratio)| ≥ 1.

The TopGO function of the Bioconductor software was used for the GO enrichment analysis of the DEGs, with a *P* value threshold of 0.01^69^. Additionally, hypergeometric tests were used for the KEGG pathway enrichment analysis of the DEGs, with 0.05 FDR corrections^70^.

### RT-qPCR

The RNA-seq data were validated by analyzing the expression of eight DEGs in a RT-qPCR assay. First-strand cDNA was synthesized from the high-quality total RNA using the RevertAid First Strand cDNA Synthesis Kit (Thermo Fisher Scientific Inc., Rockford, Illinois, USA). Gene-specific primers were designed using the Primer-Blast tool available on the NCBI website (https://www.ncbi.nlm.nih.gov/tools/primer-blast/). The housekeeping gene *ACT11* was used to normalize gene expression levels. All primers are listed in Supplementary Table S7. The RT-qPCR analysis of the eight selected genes was conducted in triplicate using the SYBR Green Real-time PCR Master Mix (Toyobo, Osaka, Japan) and the LightCycler 480 Real-Time PCR System (Roche Diagnostics GmbH, Mannheim, Germany). Relative gene expression levels were calculated according to the corrected relative 2^−ΔΔCT^ method.

## Supplementary Information

Supplementary Information.

## Data Availability

All raw data were deposited in the NCBI database (http://www.ncbi.nlm.nih.gov/bioproject/) under accession number PRJNA522338. Other study data are available upon request.
