# Supplementary material for: Transcriptome analysis identifies differentially expressed genes in the progenies of a cross between two low phytic acid soybean mutants
Source: Sci Rep. 2021 Apr 22;11:8740. doi: 10.1038/s41598-021-88055-4 (PMC8062490; doi:10.1038/s41598-021-88055-4)
Supplement: Supplementary file 1 — Supplementary Information. [file 41598_2021_88055_MOESM1_ESM.pdf]

Supplementary Tables

Transcriptome analysis identifies differentially expressed genes in the progenies of a cross between two low phytic acid soybean mutants

Hangxia Jin, Xiaomin Yu, Qinghua Yang, Xujun Fu, Fengjie Yuan *

Institute of Crop Science and Nuclear Technology Utilization, Zhejiang Academy of Agricultural Sciences, Hangzhou, Zhejiang, China

***Correspondence:**

Fengjie Yuan

fjyuanhz@126.com

Supplemental table legend

Table S1: Enriched GO terms associated with biological processes among DEGs between the *2mlpa* and 2MWT lines at five seed developmental stages

Table S2: Enriched KEGG pathways between the *2mlpa* and 2MWT lines at five seed developmental stages

Table S3: DEGs involved in PA metabolism

Table S4: DEGs involved in photosynthesis

Table S5: DEGs involved in starch and sucrose metabolism

Table S6: DEGs involved in defense mechanisms

Table S7: Primers of qPCR

Supplementary Table S1: Enriched GO terms associated with biological processes among DEGs between the *2mlpa* and 2MWT lines at five seed developmental stages

		Gene Number In Term				
GO_ID	GOTerm	Stage 1	Stage 2	Stage 3	Stage 4	Stage 5
GO:0001738	morphogenesis of a polarized epithelium	-	-	-	-	3
GO:0002009	morphogenesis of an epithelium	-	-	-	-	3
GO:0015691	cadmium ion transport	-	-	-	-	3
GO:0046108	uridine metabolic process	-	-	-	-	3
GO:0048729	tissue morphogenesis	-	-	-	-	3
GO:1901658	glycosyl compound catabolic process	-	-	-	-	3
GO:0002239	response to oomycetes	-	-	-	-	4
GO:0009403	toxin biosynthetic process	-	-	2	-	4
GO:0009700	indole phytoalexin biosynthetic process	-	-	2	-	4
GO:0015977	carbon fixation	-	-	-	-	4
GO:0035434	copper ion transmembrane transport	-	-	-	-	4
GO:0042435	indole-containing compound biosynthetic process	-	-	2	-	4
GO:0042548	regulation of photosynthesis, light reaction	-	-	-	-	4
GO:0043467	regulation of generation of precursor metabolites and energy	-	-	-	-	4
GO:0045682	regulation of epidermis development	-	-	2	-	4
GO:0046217	indole phytoalexin metabolic process	-	-	2	-	4
GO:0052314	phytoalexin metabolic process	-	-	2	-	4
GO:0052315	phytoalexin biosynthetic process	-	-	2	-	4
GO:0006658	phosphatidylserine metabolic process	2	-	-	-	4
GO:0015985	energy coupled proton transport, down electrochemical gradient	5	5	5	-	4
GO:0015986	ATP synthesis coupled proton transport	5	5	5	-	4
GO:0006536	glutamate metabolic process	-	-	-	-	5
GO:0006829	zinc II ion transport	-	-	-	-	5
GO:0010109	regulation of photosynthesis	-	-	-	-	5
GO:0032958	inositol phosphate biosynthetic process	-	-	-	-	5
GO:0046173	polyol biosynthetic process	-	-	-	-	5
GO:0072350	tricarboxylic acid metabolic process	-	-	-	-	5

20 × 20

GO:0009250	glucan biosynthetic process	-	-	-	-	7
GO:0009608	response to symbiont	-	-	-	-	7
GO:0009627	systemic acquired resistance	-	-	-	-	7
GO:0072506	trivalent inorganic anion homeostasis	-	-	2	-	7
GO:0006241	CTP biosynthetic process	-	-	-	-	8
GO:0009926	auxin polar transport	-	-	2	-	8
GO:0046036	CTP metabolic process	-	-	-	-	8
GO:1990066	energy quenching	-	-	-	-	8
GO:0015850	organic hydroxy compound transport	4	-	-	-	8
GO:0001934	positive regulation of protein phosphorylation	-	-	-	-	9
GO:0010562	positive regulation of phosphorus metabolic process	-	-	-	-	9
GO:0042327	positive regulation of phosphorylation	-	-	-	-	9
GO:0045937	positive regulation of phosphate metabolic process	-	-	-	-	9
GO:0055081	anion homeostasis	-	-	3	-	9
GO:0006220	pyrimidine nucleotide metabolic process	-	-	-	-	10
GO:0006221	pyrimidine nucleotide biosynthetic process	-	-	-	-	10
GO:0009218	pyrimidine ribonucleotide metabolic process	-	-	-	-	10
GO:0009220	pyrimidine ribonucleotide biosynthetic process	-	-	-	-	10
GO:0046132	pyrimidine ribonucleoside biosynthetic process	-	-	-	-	10
GO:0046134	pyrimidine nucleoside biosynthetic process	-	-	-	-	10
GO:0072528	pyrimidine-containing compound biosynthetic process	-	-	-	-	10
GO:0006825	copper ion transport	-	-	-	-	11
GO:0009147	pyrimidine nucleoside triphosphate metabolic process	-	-	-	-	11
GO:0009148	pyrimidine nucleoside triphosphate biosynthetic process	-	-	-	-	11
GO:0009208	pyrimidine ribonucleoside triphosphate metabolic process	-	-	-	-	11
GO:0009209	pyrimidine ribonucleoside triphosphate biosynthetic process	-	-	-	-	11
GO:0009630	gravitropism	-	-	-	-	11
GO:0009814	defense response, incompatible interaction	-	-	-	-	11
GO:0031347	regulation of defense response	-	-	-	-	11
GO:0031401	positive regulation of protein modification process	-	-	-	-	11
GO:0032270	positive regulation of cellular protein metabolic process	-	-	-	-	11
GO:0051247	positive regulation of protein metabolic process	-	-	-	-	11

GO:0002252	immune effector process	-	-	-	-	12
GO:0006074	(1->3)-beta-D-glucan metabolic process	-	-	-	-	12
GO:0009629	response to gravity	-	-	-	-	12
GO:0015914	phospholipid transport	-	-	-	-	12
GO:0010466	negative regulation of peptidase activity	8	-	-	-	12
GO:0030162	regulation of proteolysis	8	-	-	-	12
GO:0045861	negative regulation of proteolysis	8	-	-	-	12
GO:0051346	negative regulation of hydrolase activity	8	-	-	-	12
GO:0052547	regulation of peptidase activity	8	-	-	-	12
GO:0006213	pyrimidine nucleoside metabolic process	-	-	-	-	13
GO:0009409	response to cold	-	-	-	-	13
GO:0010604	positive regulation of macromolecule metabolic process	-	-	-	-	13
GO:0031325	positive regulation of cellular metabolic process	-	-	-	-	13
GO:0046131	pyrimidine ribonucleoside metabolic process	-	-	-	-	13
GO:0048437	floral organ development	-	-	-	-	13
GO:0080134	regulation of response to stress	-	-	-	-	13
GO:0043086	negative regulation of catalytic activity	8	-	-	-	13
GO:0019220	regulation of phosphate metabolic process	-	-	-	-	14
GO:0043648	dicarboxylic acid metabolic process	-	-	-	-	14
GO:0051174	regulation of phosphorus metabolic process	-	-	-	-	14
GO:0051239	regulation of multicellular organismal process	-	-	4	-	14
GO:0031399	regulation of protein modification process	-	-	-	-	15
GO:0006869	lipid transport	-	-	-	-	16
GO:0009893	positive regulation of metabolic process	-	-	-	-	16
GO:0006972	hyperosmotic response	-	-	-	-	18
GO:0009167	purine ribonucleoside monophosphate metabolic process	9	10	10	-	18
GO:0009168	purine ribonucleoside monophosphate biosynthetic process	9	10	10	-	18
GO:0009411	response to UV	-	-	-	-	19
GO:0098655	cation transmembrane transport	8	9	9	-	19
GO:0098660	inorganic ion transmembrane transport	8	9	9	-	19
GO:0098662	inorganic cation transmembrane transport	8	9	9	-	19
GO:1901698	response to nitrogen compound	-	-	5	-	20

20 × 20

GO:0009126	purine nucleoside monophosphate metabolic process	9	10	10	-	21
GO:0009127	purine nucleoside monophosphate biosynthetic process	9	10	10	-	21
GO:0009812	flavonoid metabolic process	12	4	-	-	21
GO:0009266	response to temperature stimulus	-	-	-	-	22
GO:0010876	lipid localization	-	-	-	-	22
GO:0098771	inorganic ion homeostasis	-	-	5	-	22
GO:0006633	fatty acid biosynthetic process	9	-	-	-	22
GO:0009156	ribonucleoside monophosphate biosynthetic process	9	10	11	-	22
GO:0009161	ribonucleoside monophosphate metabolic process	9	10	11	-	22
GO:0072330	monocarboxylic acid biosynthetic process	9	-	-	-	23
GO:0009914	hormone transport	-	-	-	-	25
GO:0050801	ion homeostasis	-	-	7	-	25
GO:0051273	beta-glucan metabolic process	12	-	-	-	25
GO:0009064	glutamine family amino acid metabolic process	10	-	-	-	25
GO:0009123	nucleoside monophosphate metabolic process	10	10	11	-	25
GO:0009124	nucleoside monophosphate biosynthetic process	10	10	11	-	25
GO:0060918	auxin transport	-	3	-	-	25
GO:0000302	response to reactive oxygen species	13	-	6	-	27
GO:0006955	immune response	-	-	-	-	28
GO:0045087	innate immune response	-	-	-	-	28
GO:0000041	transition metal ion transport	-	-	-	-	29
GO:0003002	regionalization	-	-	-	-	29
GO:0007389	pattern specification process	-	-	-	-	29
GO:0032268	regulation of cellular protein metabolic process	-	-	-	-	29
GO:0051246	regulation of protein metabolic process	-	-	-	-	29
GO:0009141	nucleoside triphosphate metabolic process	11	11	12	-	29
GO:0009639	response to red or far red light	-	-	-	-	30
GO:0048878	chemical homeostasis	-	-	8	-	30
GO:0034728	nucleosome organization	-	-	-	-	31
GO:0071824	protein-DNA complex subunit organization	-	-	-	-	31
GO:0015992	proton transport	12	13	13	-	31
GO:0010817	regulation of hormone levels	-	-	-	-	33

GO:0009908	flower development	-	-	-	-	34
GO:0006164	purine nucleotide biosynthetic process	13	12	13	-	36
GO:0009152	purine ribonucleotide biosynthetic process	13	12	13	-	36
GO:0002376	immune system process	-	-	-	-	38
GO:0009620	response to fungus	-	-	10	-	38
GO:0006818	hydrogen transport	16	15	16	-	38
GO:0072522	purine-containing compound biosynthetic process	14	13	14	-	38
GO:0015711	organic anion transport	19	-	-	-	39
GO:0015672	monovalent inorganic cation transport	15	13	14	-	39
GO:1901657	glycosyl compound metabolic process	13	13	15	-	41
GO:0006073	cellular glucan metabolic process	16	-	-	-	42
GO:0044264	cellular polysaccharide metabolic process	17	-	-	-	43
GO:0080090	regulation of primary metabolic process	-	-	-	-	46
GO:0009260	ribonucleotide biosynthetic process	15	13	15	-	46
GO:0046390	ribose phosphate biosynthetic process	15	13	15	-	46
GO:0032446	protein modification by small protein conjugation	-	-	-	-	48
GO:0090567	reproductive shoot system development	-	-	-	-	48
GO:0009165	nucleotide biosynthetic process	-	13	15	-	51
GO:1901293	nucleoside phosphate biosynthetic process	-	13	15	-	51
GO:0006325	chromatin organization	-	-	-	-	52
GO:0044042	glucan metabolic process	24	-	-	-	52
GO:0031323	regulation of cellular metabolic process	-	-	-	-	53
GO:0006952	defense response	-	-	11	-	54
GO:0009617	response to bacterium	-	-	-	-	55
GO:0071822	protein complex subunit organization	-	-	-	-	58
GO:0048608	reproductive structure development	-	-	-	-	59
GO:0061458	reproductive system development	-	-	-	-	59
GO:0090407	organophosphate biosynthetic process	19	14	16	-	60
GO:0048367	shoot system development	-	-	-	-	62
GO:1901137	carbohydrate derivative biosynthetic process	-	13	17	-	62
GO:0044262	cellular carbohydrate metabolic process	-	-	-	-	67
GO:0044702	single organism reproductive process	-	-	-	-	67

GO:0005976	polysaccharide metabolic process	34	-	-	-	75
GO:0009416	response to light stimulus	-	-	-	-	82
GO:0009314	response to radiation	-	-	-	-	94
GO:0009791	post-embryonic development	-	-	-	-	94
GO:0006970	response to osmotic stress	-	-	-	-	100
GO:0065008	regulation of biological quality	-	-	17	-	105
GO:0006812	cation transport	-	15	21	-	105
GO:0009725	response to hormone	-	-	-	-	111
GO:0001101	response to acid chemical	-	-	-	-	112
GO:0003006	developmental process involved in reproduction	-	-	-	-	124
GO:0048731	system development	-	-	-	-	125
GO:0009719	response to endogenous stimulus	-	-	22	-	126
GO:0043207	response to external biotic stimulus	-	-	24	-	134
GO:0051707	response to other organism	-	-	24	-	134
GO:0009607	response to biotic stimulus	-	-	24	-	138
GO:0005975	carbohydrate metabolic process	-	-	-	-	140
GO:0044711	single-organism biosynthetic process	55	19	24	-	147
GO:0010033	response to organic substance	-	-	30	-	149
GO:0051704	multi-organism process	-	-	28	-	161
GO:0006811	ion transport	71	20	31	-	172
GO:0009605	response to external stimulus	-	-	30	-	178
GO:0007275	multicellular organismal development	-	-	-	-	204
GO:0044707	single-multicellular organism process	-	-	-	-	225
GO:0032501	multicellular organismal process	-	-	-	-	231
GO:0009628	response to abiotic stimulus	-	-	-	-	251
GO:0044765	single-organism transport	111	33	46	-	269
GO:1902578	single-organism localization	112	33	47	-	272
GO:0042221	response to chemical	-	-	59	-	334
GO:0006950	response to stress	-	-	64	-	406
GO:0010467	gene expression	-	-	-	-	651
GO:0050896	response to stimulus	-	-	114	-	795
GO:0043170	macromolecule metabolic process	-	-	-	-	1199

20 × 20

GO:0071704	organic substance metabolic process	-	-	-	-	1635
GO:0009967	positive regulation of signal transduction	-	-	2	-	-
GO:0010039	response to iron ion	-	-	3	-	-
GO:0010647	positive regulation of cell communication	-	-	2	-	-
GO:0018130	heterocycle biosynthetic process	-	-	50	-	-
GO:0018871	1-aminocyclopropane-1-carboxylate metabolic process	-	-	2	-	-
GO:0019438	aromatic compound biosynthetic process	-	-	51	-	-
GO:0023056	positive regulation of signaling	-	-	2	-	-
GO:0034654	nucleobase-containing compound biosynthetic process	-	-	48	-	-
GO:0080135	regulation of cellular response to stress	-	-	2	-	-
GO:0098542	defense response to other organism	-	-	6	-	-
GO:1901362	organic cyclic compound biosynthetic process	-	-	51	-	-
GO:1901700	response to oxygen-containing compound	-	-	14	-	-
GO:1990267	response to transition metal nanoparticle	-	-	3	-	-
GO:2000026	regulation of multicellular organismal development	-	-	4	-	-
GO:0044699	single-organism process	517	-	-	-	-
GO:0006820	anion transport	32	-	-	-	-
GO:0065009	regulation of molecular function	29	-	-	-	-
GO:0044092	negative regulation of molecular function	27	-	-	-	-
GO:0033554	cellular response to stress	24	-	-	-	-
GO:0071554	cell wall organization or biogenesis	24	-	-	-	-
GO:0019748	secondary metabolic process	20	-	-	-	-
GO:0009698	phenylpropanoid metabolic process	19	-	-	-	-
GO:0044550	secondary metabolite biosynthetic process	17	-	-	-	-
GO:0009699	phenylpropanoid biosynthetic process	16	-	-	-	-
GO:0015849	organic acid transport	15	-	-	-	-
GO:0046942	carboxylic acid transport	15	-	-	-	-
GO:0006979	response to oxidative stress	13	-	-	-	-
GO:0015698	inorganic anion transport	13	-	-	-	-
GO:0048523	negative regulation of cellular process	10	-	-	-	-
GO:0030243	cellulose metabolic process	9	-	-	-	-
GO:0031324	negative regulation of cellular metabolic process	8	-	-	-	-

GO:0032269	negative regulation of cellular protein metabolic process	8	-	-	-	-
GO:0051248	negative regulation of protein metabolic process	8	-	-	-	-
GO:0009414	response to water deprivation	7	-	-	-	-
GO:0009415	response to water	7	-	-	-	-
GO:0055065	metal ion homeostasis	7	-	-	-	-
GO:0008272	sulfate transport	6	-	3	-	-
GO:0009808	lignin metabolic process	6	-	-	-	-
GO:0055076	transition metal ion homeostasis	6	-	3	-	-
GO:0072348	sulfur compound transport	6	-	3	-	-
GO:0006875	cellular metal ion homeostasis	5	-	-	-	-
GO:0009813	flavonoid biosynthetic process	5	-	-	-	-
GO:0030003	cellular cation homeostasis	5	-	3	-	-
GO:0034599	cellular response to oxidative stress	5	-	-	-	-
GO:0034614	cellular response to reactive oxygen species	5	-	-	-	-
GO:0046916	cellular transition metal ion homeostasis	5	-	-	-	-
GO:0006558	L-phenylalanine metabolic process	4	-	-	-	-
GO:0009685	gibberellin metabolic process	4	-	-	-	-
GO:0070542	response to fatty acid	4	-	2	-	-
GO:1902221	erythrose 4-phosphate/phosphoenolpyruvate family amino acid metabolic process	4	-	-	-	-
GO:0000304	response to singlet oxygen	3	-	-	-	-
GO:0006476	protein deacetylation	3	-	-	-	-
GO:0016575	histone deacetylation	3	-	-	-	-
GO:0035601	protein deacylation	3	-	-	-	-
GO:0051552	flavone metabolic process	3	-	-	-	-
GO:0051553	flavone biosynthetic process	3	-	-	-	-
GO:0098732	macromolecule deacylation	3	-	-	-	-
GO:0006817	phosphate ion transport	2	-	-	-	-
GO:0007062	sister chromatid cohesion	2	-	-	-	-
GO:0010192	mucilage biosynthetic process	2	-	-	-	-
GO:0015750	pentose transport	2	-	-	-	-
GO:0071452	cellular response to singlet oxygen	2	-	-	-	-
GO:0044710	single-organism metabolic process	301	91	-	-	-

GO:0051179	localization	181	46	-	-	-
GO:0051234	establishment of localization	179	46	-	-	-
GO:0055086	nucleobase-containing small molecule metabolic process	-	20	-	-	-
GO:1901135	carbohydrate derivative metabolic process	-	20	-	-	-
GO:1901566	organonitrogen compound biosynthetic process	-	20	23	-	-
GO:0006753	nucleoside phosphate metabolic process	-	19	-	-	-
GO:0009117	nucleotide metabolic process	-	19	-	-	-
GO:0009259	ribonucleotide metabolic process	-	19	-	-	-
GO:0019693	ribose phosphate metabolic process	-	19	-	-	-
GO:0072521	purine-containing compound metabolic process	-	19	-	-	-
GO:0006163	purine nucleotide metabolic process	-	18	-	-	-
GO:0009150	purine ribonucleotide metabolic process	-	18	-	-	-
GO:0034220	ion transmembrane transport	-	10	10	-	-
GO:0055085	transmembrane transport	-	10	12	-	-
GO:1902600	hydrogen ion transmembrane transport	8	8	9	-	-
GO:0015980	energy derivation by oxidation of organic compounds	-	7	6	-	-
GO:0045333	cellular respiration	9	6	5	-	-
GO:0006461	protein complex assembly	-	5	-	-	-
GO:0015988	energy coupled proton transmembrane transport, against electrochemical gradient	-	5	6	-	-
GO:0022904	respiratory electron transport chain	-	5	3	-	-
GO:0070271	protein complex biogenesis	-	5	-	-	-
GO:0006835	dicarboxylic acid transport	-	3	-	-	-
GO:0015740	C4-dicarboxylate transport	-	3	-	-	-
GO:0006308	DNA catabolic process	-	2	-	-	-
GO:0009692	ethylene metabolic process	-	2	2	-	-
GO:0015936	coenzyme A metabolic process	-	2	2	-	-
GO:0015937	coenzyme A biosynthetic process	-	2	2	-	-
GO:0033865	nucleoside bisphosphate metabolic process	-	2	2	-	-
GO:0033866	nucleoside bisphosphate biosynthetic process	-	2	2	-	-
GO:0033875	ribonucleoside bisphosphate metabolic process	-	2	2	-	-
GO:0034030	ribonucleoside bisphosphate biosynthetic process	-	2	2	-	-
GO:0034032	purine nucleoside bisphosphate metabolic process	-	2	2	-	-

GO:0034033	purine nucleoside bisphosphate biosynthetic process	-	2	2	-	-
GO:0043449	cellular alkene metabolic process	-	2	2	-	-
GO:0051258	protein polymerization	-	2	2	-	-
GO:0051259	protein oligomerization	-	2	-	-	-
GO:1900673	olefin metabolic process	-	2	-	-	-
GO:0019752	carboxylic acid metabolic process	-	-	-	28	-
GO:0006508	proteolysis	68	-	-	27	-
GO:0006091	generation of precursor metabolites and energy	30	31	26	26	97
GO:0016485	protein processing	52	-	-	24	-
GO:0051604	protein maturation	52	-	-	24	-
GO:0010035	response to inorganic substance	40	-	18	19	123
GO:0010038	response to metal ion	-	-	-	17	108
GO:0055114	oxidation-reduction process	21	21	20	15	39
GO:0032787	monocarboxylic acid metabolic process	31	-	-	14	-
GO:0022900	electron transport chain	12	19	16	12	-
GO:0034660	ncRNA metabolic process	-	-	-	11	-
GO:0006631	fatty acid metabolic process	23	-	-	11	-
GO:0015979	photosynthesis	-	12	12	9	18
GO:0006399	tRNA metabolic process	-	-	-	9	-
GO:0019684	photosynthesis, light reaction	-	11	11	8	16
GO:0090305	nucleic acid phosphodiester bond hydrolysis	-	-	-	8	-
GO:0090501	RNA phosphodiester bond hydrolysis	8	-	-	8	-
GO:0031668	cellular response to extracellular stimulus	10	-	-	7	24
GO:0031669	cellular response to nutrient levels	10	-	-	7	24
GO:0071496	cellular response to external stimulus	10	-	-	7	24
GO:0009991	response to extracellular stimulus	-	-	-	7	25
GO:0031667	response to nutrient levels	-	-	-	7	25
GO:0034470	ncRNA processing	-	-	-	7	-
GO:0009267	cellular response to starvation	10	-	-	6	20
GO:0042594	response to starvation	10	-	-	6	20
GO:0008033	tRNA processing	-	-	-	6	-
GO:0048507	meristem development	-	-	-	6	-

20 × 20

GO:0009163	nucleoside biosynthetic process	12	13	14	5	28
GO:0042455	ribonucleoside biosynthetic process	12	13	14	5	28
GO:0009116	nucleoside metabolic process	12	13	14	5	32
GO:0009119	ribonucleoside metabolic process	12	13	14	5	32
GO:1901659	glycosyl compound biosynthetic process	13	13	15	5	34
GO:0015893	drug transport	10	-	-	5	-
GO:0042493	response to drug	10	-	-	5	-
GO:0009767	photosynthetic electron transport chain	-	7	6	5	-
GO:0019751	polyol metabolic process	-	3	-	4	14
GO:0010243	response to organonitrogen compound	-	-	5	4	15
GO:0042451	purine nucleoside biosynthetic process	10	12	12	4	18
GO:0046129	purine ribonucleoside biosynthetic process	10	12	12	4	18
GO:0046128	purine ribonucleoside metabolic process	10	12	12	4	19
GO:0042278	purine nucleoside metabolic process	10	12	12	4	20
GO:0009142	nucleoside triphosphate biosynthetic process	11	11	12	4	28
GO:0009199	ribonucleoside triphosphate metabolic process	11	11	12	4	28
GO:0009201	ribonucleoside triphosphate biosynthetic process	11	11	12	4	28
GO:0006457	protein folding	-	-	-	4	-
GO:0006754	ATP biosynthetic process	9	10	10	3	17
GO:0009144	purine nucleoside triphosphate metabolic process	9	10	10	3	17
GO:0009145	purine nucleoside triphosphate biosynthetic process	9	10	10	3	17
GO:0009205	purine ribonucleoside triphosphate metabolic process	9	10	10	3	17
GO:0009206	purine ribonucleoside triphosphate biosynthetic process	9	10	10	3	17
GO:0046034	ATP metabolic process	9	10	10	3	17
GO:0010051	xylem and phloem pattern formation	-	-	-	3	-
GO:0043647	inositol phosphate metabolic process	5	-	-	3	-
GO:0009404	toxin metabolic process	-	-	2	2	5
GO:0006570	tyrosine metabolic process	-	-	-	2	-
GO:0009637	response to blue light	-	-	-	2	-
GO:0006020	inositol metabolic process	-	2	2	2	-

Supplementary Table S2: Enriched KEGG pathways between the *2mlpa* and 2MWT lines at five seed developmental stages

	Gene Number In Pathway					
#Pathway	Pathway ID	Stage 1	Stage 2	Stage 3	Stage 4	Stage 5
Glycolysis / Gluconeogenesis	ko00010	18	3	3	9	39
Citrate cycle (TCA cycle)	ko00020	1			1	12
Pentose phosphate pathway	ko00030	6	2	3	2	19
Pentose and glucuronate interconversions	ko00040	15	3	2	6	31
Fructose and mannose metabolism	ko00051	4		1	2	17
Galactose metabolism	ko00052	15		1	3	26
Ascorbate and aldarate metabolism	ko00053	11	3	5	7	26
Fatty acid biosynthesis	ko00061	10	2	1		11
Fatty acid elongation	ko00062	4	1	3	3	6
Fatty acid metabolism	ko00071	9	3	3	5	19
Synthesis and degradation of ketone bodies	ko00072	1	1	1	1	2
Cutin, suberine and wax biosynthesis	ko00073	17	2	1	4	16
Steroid biosynthesis	ko00100	2			1	11
Ubiquinone and other terpenoid-quinone biosynthesis	ko00130	12		1		19
Oxidative phosphorylation	ko00190	25	30	29	7	67
Photosynthesis	ko00195	24	33	32	28	69
Photosynthesis - antenna proteins	ko00196	1			5	22
Purine metabolism	ko00230	37	11	19	12	104
Caffeine metabolism	ko00232					1
Pyrimidine metabolism	ko00240	39	9	19	12	100
Alanine, aspartate and glutamate metabolism	ko00250	6	2	3	6	31
Glycine, serine and threonine metabolism	ko00260	16	1	8	2	18
Cysteine and methionine metabolism	ko00270	11	3	9	7	24
Valine, leucine and isoleucine degradation	ko00280	4	2	2	2	15

20 × 20

Valine, leucine and isoleucine biosynthesis	ko00290	8		6		10
Lysine biosynthesis	ko00300					2
Lysine degradation	ko00310	6	3	2	3	21
Arginine and proline metabolism	ko00330	13	2	1	6	27
Histidine metabolism	ko00340	3	1		1	5
Tyrosine metabolism	ko00350	6	2	2	5	15
Phenylalanine metabolism	ko00360	22	2	4	5	39
Tryptophan metabolism	ko00380	10	2	3	5	12
Phenylalanine, tyrosine and tryptophan biosynthesis	ko00400	5	1		4	8
Benzoxazinoid biosynthesis	ko00402	6	2	3		5
beta-Alanine metabolism	ko00410	5	2		1	16
Taurine and hypotaurine metabolism	ko00430	7		1	2	7
Selenocompound metabolism	ko00450	2	3	2	3	7
Cyanoamino acid metabolism	ko00460	9	2	6	5	21
Glutathione metabolism	ko00480	11	4	5	1	25
Starch and sucrose metabolism	ko00500	30	5	14	14	75
N-Glycan biosynthesis	ko00510	3		2	1	9
Other glycan degradation	ko00511	21	1	2	3	21
Other types of O-glycan biosynthesis	ko00514				1	2
Amino sugar and nucleotide sugar metabolism	ko00520	9	1	6	3	38
Glycosaminoglycan degradation	ko00531	3				11
Glycerolipid metabolism	ko00561	10	1	2	1	16
Inositol phosphate metabolism	ko00562	8	4	5	5	31
Glycosylphosphatidylinositol(GPI)-anchor biosynthesis	ko00563	12		5	3	29
Glycerophospholipid metabolism	ko00564	7		2		23
Ether lipid metabolism	ko00565	1				5
Arachidonic acid metabolism	ko00590	3		1		2
Linoleic acid metabolism	ko00591	9		2	2	16

alpha-Linolenic acid metabolism	ko00592	8		3	5	23
Sphingolipid metabolism	ko00600	6				15
Glycosphingolipid biosynthesis - globo series	ko00603	3				2
Glycosphingolipid biosynthesis - ganglio series	ko00604	3				7
Pyruvate metabolism	ko00620	17	5	4	6	36
Glyoxylate and dicarboxylate metabolism	ko00630	9	1	2	1	17
Propanoate metabolism	ko00640	14	4	2	3	23
Butanoate metabolism	ko00650	3	1	1	1	12
C5-Branched dibasic acid metabolism	ko00660					2
One carbon pool by folate	ko00670	2		1		5
Carbon fixation in photosynthetic organisms	ko00710	9	2	1	4	31
Thiamine metabolism	ko00730	1			1	2
Riboflavin metabolism	ko00740	2				8
Vitamin B6 metabolism	ko00750	3	2	1	2	9
Nicotinate and nicotinamide metabolism	ko00760					2
Pantothenate and CoA biosynthesis	ko00770	1		1	1	5
Biotin metabolism	ko00780					1
Lipoic acid metabolism	ko00785	1			1	2
Folate biosynthesis	ko00790	1				4
Porphyrin and chlorophyll metabolism	ko00860	7		3	1	19
Terpenoid backbone biosynthesis	ko00900	4	2	2	2	12
Indole alkaloid biosynthesis	ko00901	4		2		1
Monoterpenoid biosynthesis	ko00902					3
Limonene and pinene degradation	ko00903	19	3	4	8	29
Diterpenoid biosynthesis	ko00904	12	3	3	5	21
Brassinosteroid biosynthesis	ko00905	2		1		4
Carotenoid biosynthesis	ko00906	24		5	5	16
Zeatin biosynthesis	ko00908	6	3	5	4	13

Sesquiterpenoid and triterpenoid biosynthesis	ko00909	4	2	1		2
Nitrogen metabolism	ko00910	7	3	4	4	25
Sulfur metabolism	ko00920	2	2	2	1	9
Phenylpropanoid biosynthesis	ko00940	42	9	14	14	74
Flavonoid biosynthesis	ko00941	45	18	14	12	67
Anthocyanin biosynthesis	ko00942	2				1
Isoflavonoid biosynthesis	ko00943	9		3	2	8
Flavone and flavonol biosynthesis	ko00944	22	7	9	7	26
Stilbenoid, diarylheptanoid and gingerol biosynthesis	ko00945	28	6	10	9	43
Isoquinoline alkaloid biosynthesis	ko00950	3	1		2	9
Tropane, piperidine and pyridine alkaloid biosynthesis	ko00960	4	1		2	10
Betalain biosynthesis	ko00965					1
Glucosinolate biosynthesis	ko00966	5		2	1	4
Aminoacyl-tRNA biosynthesis	ko00970	3	1	1	2	10
Biosynthesis of unsaturated fatty acids	ko01040	6	1	1	2	12
Metabolic pathways	ko01100	361	108	153	138	794
Biosynthesis of secondary metabolites	ko01110	210	42	66	65	374
ABC transporters	ko02010	21	1	0	3	46
Ribosome biogenesis in eukaryotes	ko03008	14	2	3	15	45
Ribosome	ko03010	27	18	22	15	252
RNA transport	ko03013	26	2	9	16	61
mRNA surveillance pathway	ko03015	10	2	2	4	28
RNA degradation	ko03018	12	4	6	7	31
RNA polymerase	ko03020	28	6	15	6	58
Basal transcription factors	ko03022	2	2			4
DNA replication	ko03030	3	1	2	3	20
Spliceosome	ko03040	17	1	5	11	66
Proteasome	ko03050	1			1	6

Protein export	ko03060	6	1	3	3	30
Base excision repair	ko03410	7			2	16
Nucleotide excision repair	ko03420	3	1	1	2	20
Mismatch repair	ko03430	3	1	3	1	12
Homologous recombination	ko03440	9	3	6	1	22
Non-homologous end-joining	ko03450					2
Phosphatidylinositol signaling system	ko04070	7	2	5	3	37
Plant hormone signal transduction	ko04075	67	12	27	38	252
Ubiquitin mediated proteolysis	ko04120	13	1	8	9	64
Sulfur relay system	ko04122					1
SNARE interactions in vesicular transport	ko04130	2		1		22
Regulation of autophagy	ko04140	5		2		29
Protein processing in endoplasmic reticulum	ko04141	24	2	14	5	113
Endocytosis	ko04144	4	1	3	3	27
Phagosome	ko04145	12	2	3	1	47
Peroxisome	ko04146	12	1			33
Plant-pathogen interaction	ko04626	109	26	54	39	303
Natural killer cell mediated cytotoxicity	ko04650			2		7
Circadian rhythm - mammal	ko04710	2	1	1		9
Circadian rhythm - plant	ko04712	12	4	6	7	36

Supplementary Table S3: DEGs involved in PA metabolism

GeneID	log2Ratio(2mlpa-1/2MWT-1)	P-value	log2Ratio(2mlpa-2/2MWT-2)	P-value	log2Ratio(2mlpa-3/2MWT-3)	P-value	log2Ratio(2mlpa-4/2MWT-4)	P-value	log2Ratio(2mlpa-5/2MWT-5)	P-value	Length	Pathway	GO Component	GO Function	GO Process	Blast nr
Glyma.04G251700	-	-	-	-	1.4419	2.38E-05	-	-	-	-	1819	ko00562//Inositol phosphate metabolism;ko03008//Ribosome biogenesis in eukaryotes;ko04070//Phosphatidylinositol signaling system;ko01100//Metabolic pathways;ko04144//Endocytosis;ko04712//Circadian rhythm - plant	-	-	-	gi|356509438|ref|XP_003523456.1|/0/PREDICTED: phosphatidylinositol 4-phosphate 5-kinase 4-like [Glycine max]
Glyma.05G070400	1.1479	0.00074	-	-	-	-	-	-	-	-	4016	ko00562//Inositol phosphate metabolism;ko04070//Phosphatidylinositol signaling system;ko01100//Metabolic pathways	-	-	GO:0046488//phosphatidylinositol metabolic process;GO:0043647//inositol phosphate metabolic process;GO:0044707;GO:0044723;GO:0001101//response to acid chemical	gi|947109262|gb|KRH57588.1|;gi|947109263|gb|KRH57589.1|;gi|571454088|ref|XP_006579677.1|;gi|947109261|gb|KRH57587.1|/0;0;0;0/hypothetical protein GLYMA_05G070400 [Glycine max];hypothetical protein GLYMA_05G070400 [Glycine max];PREDICTED: type I inositol polyphosphate 5-phosphatase 2-like [Glycine max];hypothetical protein GLYMA_05G070400 [Glycine max]
Glyma.07G013900	-	-	-3.24	4.48E-06	-3.276	5.75E-06	-2.599	3.98E-05	-	-	1625	ko00562//Inositol phosphate metabolism;ko00053//Ascorbate and aldarate metabolism	GO:0044424	GO:0046914//transition metal ion binding;GO:0016701//oxidoreductase activity, acting on single donors with incorporation of molecular oxygen	GO:0006020//inositol metabolic process	gi|947098691|gb|KRH47183.1|;gi|947098690|gb|KRH47182.1|;gi|947098692|gb|KRH47184.1|/4.8486e-172;0;0/hypothetical protein GLYMA_07G013900 [Glycine max];hypothetical protein GLYMA_07G013900 [Glycine max];hypothetical protein GLYMA_07G013900 [Glycine max]
Glyma.17G153000	-1.484	0.00028	-	-	-	-	-1.409	0.0012368	-	-	2721	ko00562//Inositol phosphate metabolism;ko04070//Phosphatidylinositol signaling system;ko01100//Metabolic pathways	-	-	GO:0046488//phosphatidylinositol metabolic process;GO:0043647//inositol phosphate metabolic process	gi|947054852|gb|KRH04305.1|;gi|356563443|ref|XP_003549972.1|/0;0/hypothetical protein GLYMA_17G153000 [Glycine max];PREDICTED: type I inositol polyphosphate 5-phosphatase 2-like [Glycine max]
Glyma.18G027200	-1.085	0.00021	-	-	-	-	-	-	-	-	2057	ko00562//Inositol phosphate metabolism;ko04070//Phosphatidylinositol signaling system;ko01100//Metabolic pathways	-	GO:0060089;GO:0046872//metal ion binding;GO:0004629//phospholipase C activity	GO:0007165//signal transduction;GO:0044238//primary metabolic process	gi|356568316|ref|XP_003552358.1|;gi|571542533|ref|XP_006601952.1|/0;0/PREDICTED: phosphoinositide phospholipase C 6 isoform X2 [Glycine max];PREDICTED: phosphoinositide phospholipase C 6 isoform X1 [Glycine max]
Glyma.01G016700	8.6673	1.87E-42	11.304	2.50E-38	9.9449	6.11E-24	10.415	4.45E-26	9.9027	1.85E-15	1074	ko00562//Inositol phosphate metabolism;ko04070//Phosphatidylinositol signaling system;ko01100//Metabolic pathways	GO:0044464	GO:0046872//metal ion binding;GO:0051766//inositol trisphosphate kinase activity;GO:0051765//inositol tetrakisphosphate kinase activity;GO:0032550	GO:0043647//inositol phosphate metabolic process	gi|947126543|gb|KRH74397.1|/0/hypothetical protein GLYMA_01G016700 [Glycine max]
Glyma.05G224500	-	-	-	-	-	-	-	-	3.3182	0.002618	1497	ko00562//Inositol phosphate metabolism;ko00053//Ascorbate and aldarate metabolism	GO:0044424	GO:0046914//transition metal ion binding;GO:0016701//oxidoreductase activity, acting on single donors with incorporation of molecular oxygen	GO:0048646//anatomical structure formation involved in morphogenesis;GO:0006020//inositol metabolic process	gi|947111847|gb|KRH60173.1|;gi|947111848|gb|KRH60174.1|;gi|947111846|gb|KRH60172.1|;gi|734310448|gb|KHM99828.1|/2.07501e-180;0;0;0/hypothetical protein GLYMA_05G224500 [Glycine max];hypothetical protein GLYMA_05G224500 [Glycine max];hypothetical protein GLYMA_05G224500 [Glycine max];Inositol oxygenase 4 [Glycine soja]

Glyma.03G186300	-	-	-	-	-	-	-	-	2.9732	0.0057441	3274	ko00562//Inositol phosphate metabolism;ko04070//Phosphatidylinositol signaling system;ko01100//Metabolic pathways;ko04144//Endocytosis	-	GO:0016307//phosphatidylinositol phosphate kinase activity;GO:0032550	GO:0046488//phosphatidylinositol metabolic process	gi|571446255|ref|XP_006577040.1|/0/PREDICTED: phosphatidylinositol 4-phosphate 5-kinase 9-like [Glycine max]
Glyma.07G266200	-	-	-	-	-	-	-	-	2.8717	0.0014403	1525	ko00562//Inositol phosphate metabolism;ko00053//Ascorbate and aldarate metabolism;ko04070//Phosphatidylinositol signaling system;ko01100//Metabolic pathways;ko01110//Biosynthesis of secondary metabolites	GO:0044444;GO:0016020//membrane	GO:0070456	GO:0019852//L-ascorbic acid metabolic process;GO:0046488//phosphatidylinositol metabolic process;GO:0009628//response to abiotic stimulus;GO:0006950//response to stress	gi|734423661|gb|KHN42287.1|;gi|947102679|gb|KRH51171.1|/0;0/Inositol monophosphatase 3 [Glycine soja] ;hypothetical protein GLYMA_07G266200 [Glycine max]
Glyma.05G180600	-	-	-	-	-	-	-	-	2.6821	6.06E-07	1864	ko00562//Inositol phosphate metabolism;ko01100//Metabolic pathways;ko01110//Biosynthesis of secondary metabolites	GO:0044424	GO:0016872//intramolecular lyase activity	GO:0009642//response to light intensity;GO:0009620//response to fungus;GO:0000302//response to reactive oxygen species;GO:0006020//inositol metabolic process;GO:0009617//response to bacterium;GO:0006658//phosphatidylserine metabolic process;GO:0002252;GO:0007275//multicellular organismal development;GO:0003006//developmental process involved in reproduction;GO:0032958//inositol phosphate biosynthetic process;GO:0006644//phospholipid metabolic process	gi|947111062|gb|KRH59388.1|;gi|947111064|gb|KRH59390.1|;gi|947111063|gb|KRH59389.1|;gi|356512722|ref|XP_003525065.1|/0;0;0;0/hypothetical protein GLYMA_05G180600 [Glycine max];hypothetical protein GLYMA_05G180600 [Glycine max];hypothetical protein GLYMA_05G180600 [Glycine max];PREDICTED: inositol-3-phosphate synthase [Glycine max]
Glyma.17G259400	-	-	-	-	-	-	-	-	2.5304	0.001405	984	ko00562//Inositol phosphate metabolism;ko04070//Phosphatidylinositol signaling system;ko04145//Phagosome	-	GO:0043169//cation binding	-	gi|734351221|gb|KHN12631.1|/0/WD repeat and FYVE domain-containing protein 3 [Glycine soja]
Glyma.10G218400	-	-	-	-	-	-	-	-	2.4082	5.02E-06	5471	ko00562//Inositol phosphate metabolism;ko04070//Phosphatidylinositol signaling system;ko04145//Phagosome	-	GO:0016301//kinase activity;GO:0032550	GO:0046488//phosphatidylinositol metabolic process;GO:0019538//protein metabolic process	gi|571484133|ref|XP_006589464.1|/0/PREDICTED: putative 1-phosphatidylinositol-3-phosphate 5-kinase FAB1D isoform X1 [Glycine max]
Glyma.06G096300	-	-	-	-	-	-	-	-	2.0648	7.46E-05	3011	ko00562//Inositol phosphate metabolism;ko04140//Regulation of autophagy;ko04070//Phosphatidylinositol signaling system;ko01100//Metabolic pathways;ko04145//Phagosome	-	GO:0032550;GO:0052742	GO:0016192//vesicle-mediated transport;GO:0048017//inositol lipid-mediated signaling;GO:0006661//phosphatidylinositol biosynthetic process;GO:0006970//response to osmotic stress;GO:0009555//pollen development	gi|734339581|gb|KHN08996.1|/0/Phosphatidylinositol 3-kinase, nodule isoform [Glycine soja]
Glyma.19G158700	-	-	-	-	-	-	-	-	2.0161	0.004807	2946	ko00562//Inositol phosphate metabolism;ko04070//Phosphatidylinositol signaling system;ko01100//Metabolic pathways	-	-	GO:0046488//phosphatidylinositol metabolic process	gi|734317079|gb|KHN02561.1|;gi|955389963|ref|XP_014627585.1|;gi|947045932|gb|KRG95561.1|;gi|947045936|gb|KRG95565.1|;gi|947045933|gb|KRG95562.1|/0;0;0;0;0/Type I inositol-1,4,5-trisphosphate 5-phosphatase CVP2 [Glycine soja] ;PREDICTED: type I inositol polyphosphate 5-phosphatase 4-like isoform X1 [Glycine max];hypothetical protein GLYMA_19G158700 [Glycine max];hypothetical protein GLYMA_19G158700 [Glycine max];hypothetical protein GLYMA_19G158700 [Glycine max]
Glyma.06G013800	-	-	-	-	-	-	-	-	1.9627	2.11E-07	6537	ko00562//Inositol phosphate metabolism;ko04070//Phosphatidylinositol signaling system;ko01100//Metabolic pathways	GO:0005911//cell-cell junction;GO:0016020//memb	GO:0016772//transferase activity, transferring phosphorus-containing groups;GO:1902936;GO:0003779//actin binding	GO:0046488//phosphatidylinositol metabolic process;GO:0048017//inositol lipid-mediated signaling;GO:0006464//cellular protein modification process	gi|947103164|gb|KRH51547.1|;gi|947103165|gb|KRH51548.1|/0;0/hypothetical protein GLYMA_06G013800 [Glycine max];hypothetical protein GLYMA_06G013800 [Glycine max]

													rane			
Glyma.11G041600	-	-	-	-	-	-	-	-	1.9501	0.0017678	3762	ko00562//Inositol phosphate metabolism;ko04070//Phosphatidylinositol signaling system;ko01100//Metabolic pathways	-	-	GO:0046488//phosphatidylinositol metabolic process;GO:0043647//inositol phosphate metabolic process;GO:0044707;GO:0044723;GO:0001101//response to acid chemical	gi|571487238|ref|XP_006590604.1|;gi|734408756|gb|KHN34863.1|;gi|947079477|gb|KRH28266.1|/0;0;0/PREDICTED: type I inositol polyphosphate 5-phosphatase 2-like [Glycine max];Type I inositol-1,4,5-trisphosphate 5-phosphatase 2 [Glycine soja];hypothetical protein GLYMA_11G041600 [Glycine max]
Glyma.01G200500	-	-	-	-	-	-	-	-	1.8766	0.0005575	2276	ko00562//Inositol phosphate metabolism;ko04070//Phosphatidylinositol signaling system;ko01100//Metabolic pathways	-	-	-	gi|947129377|gb|KRH77231.1|/0/hypothetical protein GLYMA_01G200500 [Glycine max]
Glyma.20G173200	-	-	-	-	-	-	-	-	1.8656	0.0007583	5623	ko00562//Inositol phosphate metabolism;ko04070//Phosphatidylinositol signaling system;ko04145//Phagosome	-	GO:0016301//kinase activity;GO:0032550	GO:0046488//phosphatidylinositol metabolic process;GO:0019538//protein metabolic process	gi|571568246|ref|XP_006606196.1|/0/PREDICTED: putative 1-phosphatidylinositol-3-phosphate 5-kinase FAB1D isoform X1 [Glycine max]
Glyma.18G018600	-1.302	2.09E-10	-	-	-	-	-	-	1.7651	0.0074304	2095	ko00562//Inositol phosphate metabolism;ko01100//Metabolic pathways;ko01110//Biosynthesis of secondary metabolites	-	GO:0016872//intramolecular lyase activity	GO:0009642//response to light intensity;GO:0009620//response to fungus;GO:0000302//response to reactive oxygen species;GO:0006020//inositol metabolic process;GO:0009617//response to bacterium;GO:0006658//phosphatidylserine metabolic process;GO:0002252;GO:0007275//multicellular organismal development;GO:0003006//developmental process involved in reproduction;GO:0032958//inositol phosphate biosynthetic process;GO:0006644//phospholipid metabolic process	gi|947048073|gb|KRG97601.1|;gi|947048074|gb|KRG97602.1|;gi|84311235|gb|ABC55420.1|;gi|947048072|gb|KRG97600.1|/0;0;0;0/hypothetical protein GLYMA_18G018600 [Glycine max];hypothetical protein GLYMA_18G018600 [Glycine max];myo-inositol-1-phosphate synthase [Glycine max];hypothetical protein GLYMA_18G018600 [Glycine max]
Glyma.20G013500	-	-	-	-	-	-	-	-	1.6738	5.93E-06	6424	ko00562//Inositol phosphate metabolism;ko04070//Phosphatidylinositol signaling system;ko04145//Phagosome	-	GO:0043169//cation binding;GO:0016301//kinase activity;GO:0032550	GO:0046488//phosphatidylinositol metabolic process;GO:0019538//protein metabolic process	gi|955392583|ref|XP_014628145.1|/0/PREDICTED: putative 1-phosphatidylinositol-3-phosphate 5-kinase FAB1C [Glycine max]
Glyma.17G222600	-	-	-	-	-	-	-	-	1.591	0.0025168	4395	ko00562//Inositol phosphate metabolism;ko04070//Phosphatidylinositol signaling system	-	-	-	gi|947055910|gb|KRH05363.1|/0/hypothetical protein GLYMA_17G222600 [Glycine max]
Glyma.07G273800	-	-	-	-	-	-	-	-	1.5463	0.0085208	3844	ko00562//Inositol phosphate metabolism;ko04070//Phosphatidylinositol signaling system;ko01100//Metabolic pathways	-	GO:0046030//inositol trisphosphate phosphatase activity	GO:0009888//tissue development;GO:0001101//response to acid chemical;GO:0046488//phosphatidylinositol metabolic process;GO:0009416//response to light stimulus;GO:0031667//response to nutrient levels;GO:0009756//carbohydrate mediated signaling;GO:0048878//chemical homeostasis;GO:0044723;GO:0022622//root system development;GO:0006950//response to stress	gi|571468135|ref|XP_006584139.1|/0/PREDICTED: type I inositol polyphosphate 5-phosphatase 12-like isoform X2 [Glycine max]
Glyma.07G216600	-	-	-	-	-	-	-	-	1.5295	7.62E-05	6440	ko00562//Inositol phosphate metabolism;ko04070//Phosphatidylinositol signaling	-	GO:0043169//cation binding;GO:0016301//kinase	GO:0046488//phosphatidylinositol metabolic process;GO:0019538//protein	gi|955332007|ref|XP_014633654.1|;gi|947101860|gb|KRH50352.1|/0;0/PREDICTED: putative 1-phosphatidylinositol-3-phosphate 5-kinase FAB1C

												system;ko04145//Phagosome		activity;GO:0032550	metabolic process	[Glycine max];hypothetical protein GLYMA_07G216600 [Glycine max]
Glyma.17G102800	-1.159	7.45E-07	-	-	-	-	-	-	1.5245	0.0002029	4611	ko00562//Inositol phosphate metabolism;ko04070//Phosphatidylinositol signaling system	-	-	-	gi|955378570|ref|XP_014625002.1|/0/PREDICTED: formin-like protein 20 [Glycine max]
Glyma.06G089800	-	-	-	-	-	-	-	-	1.423	8.19E-05	2250	ko00562//Inositol phosphate metabolism;ko04070//Phosphatidylinositol signaling system;ko01100//Metabolic pathways	GO:0044464	GO:0046872//metal ion binding;GO:0051766//inositol trisphosphate kinase activity;GO:0051765//inositol tetrakisphosphate kinase activity;GO:0032550	GO:0043647//inositol phosphate metabolic process	gi|351721983|ref|NP_001237484.1|/0/inositol phosphate kinase [Glycine max]
Glyma.08G276400	-	-	-	-	-	-	-	-	1.3953	0.0002009	3700	ko00562//Inositol phosphate metabolism;ko00640//Propanoate metabolism;ko00410//beta-Alanine metabolism;ko01100//Metabolic pathways;ko00280//Valine, leucine and isoleucine degradation	-	GO:0016620//oxidoreductase activity, acting on the aldehyde or oxo group of donors, NAD or NADP as acceptor	GO:0008152//metabolic process	gi|955337351|ref|XP_014634773.1|;gi|955337353|ref|XP_014634774.1|/0;0/PREDICTED: putative succinate-semialdehyde dehydrogenase C1002.12c [NADP(+)] isoform X1 [Glycine max];PREDICTED: putative succinate-semialdehyde dehydrogenase C1002.12c [NADP(+)] isoform X2 [Glycine max]
Glyma.04G213400	-	-	-	-	-	-	-	-	1.3361	0.0004795	5026	ko00562//Inositol phosphate metabolism;ko04070//Phosphatidylinositol signaling system;ko01100//Metabolic pathways	-	GO:0052742	GO:0046488//phosphatidylinositol metabolic process;GO:0048017//inositol lipid-mediated signaling;GO:0046474//glycerophospholipid biosynthetic process	gi|955317369|ref|XP_014630360.1|;gi|947115746|gb|KRH64048.1|;gi|947115749|gb|KRH64051.1|;gi|571451616|ref|XP_006578790.1|/0;0;0;0/PREDICTED: phosphatidylinositol 4-kinase beta 1-like isoform X2 [Glycine max];hypothetical protein GLYMA_04G213400 [Glycine max];hypothetical protein GLYMA_04G213400 [Glycine max];PREDICTED: phosphatidylinositol 4-kinase beta 1-like isoform X1 [Glycine max]
Glyma.07G045600	-	-	-	-	-	-	-	-	1.2135	0.0018572	7013	ko00562//Inositol phosphate metabolism;ko04070//Phosphatidylinositol signaling system;ko04145//Phagosome	GO:0043231//intracellular membrane-bounded organelle	GO:0043169//cation binding;GO:0032550;GO:0016307//phosphatidylinositol phosphate kinase activity;GO:0016301//kinase activity	GO:0010118//stomatal movement;GO:0006996//organelle organization;GO:0046488//phosphatidylinositol metabolic process;GO:0048229//gametophyte development;GO:0019538//protein metabolic process	gi|571464853|ref|XP_006583187.1|;gi|947099221|gb|KRH47713.1|;gi|947099222|gb|KRH47714.1|/0;0;0/PREDICTED: 1-phosphatidylinositol-3-phosphate 5-kinase FAB1B-like [Glycine max];hypothetical protein GLYMA_07G045600 [Glycine max];hypothetical protein GLYMA_07G045600 [Glycine max]
Glyma.20G218600	-	-	-	-	-	-	-	-	1.0042	0.0043565	3874	ko00562//Inositol phosphate metabolism;ko04070//Phosphatidylinositol signaling system;ko01100//Metabolic pathways	-	-	GO:0046488//phosphatidylinositol metabolic process	gi|571569653|ref|XP_006606425.1|;gi|947042834|gb|KRG92558.1|/0;0/PREDICTED: type II inositol polyphosphate 5-phosphatase 15-like [Glycine max];hypothetical protein GLYMA_20G218600 [Glycine max]
Glyma.08G086800	-	-	-	-	-	-	-	-	-1.046	0.0026145	1665	ko00562//Inositol phosphate metabolism;ko00920//Sulfur metabolism;ko04070//Phosphatidylinositol signaling system;ko01100//Metabolic pathways	-	GO:0008252//nucleotidase activity	GO:0046488//phosphatidylinositol metabolic process	gi|356524978|ref|XP_003531104.1|/0/PREDICTED: SAL1 phosphatase [Glycine max]
Glyma.02G076500	-	-	-	-	-	-	-	-	-1.087	0.0011053	1436	ko00562//Inositol phosphate metabolism;ko01100//Metabolic pathways;ko01110//Biosynthesis of secondary metabolites	-	GO:0003824//catalytic activity	GO:0044710	gi|356499161|ref|XP_003518411.1|/0/PREDICTED: uncharacterized oxidoreductase At4g09670-like [Glycine max]
Glyma.08G127700	-	-	-	-	-	-	-	-	-1.25	0.0017466	3280	ko00562//Inositol phosphate metabolism;ko04070//Phosphatidylinositol signaling system;ko01100//Metabolic pathways;ko04144//Endocytosis	GO:0016020//membrane	GO:0016307//phosphatidylinositol phosphate kinase activity;GO:0032550	GO:0046488//phosphatidylinositol metabolic process	gi|356525381|ref|XP_003531303.1|;gi|571471155|ref|XP_006585224.1|/0;0/PREDICTED: phosphatidylinositol 4-phosphate 5-kinase 7 isoform X1 [Glycine max];PREDICTED: phosphatidylinositol 4-phosphate 5-kinase 7 isoform X2 [Glycine max]

Glyma.14G072200	-	-	-	-	-	-	-	-	-1.278	0.0005507	2575	ko00562//Inositol phosphate metabolism;ko04070//Phosphatidylinositol signaling system;ko01100//Metabolic pathways	-	GO:0032550;GO:0051765//inositol tetrakisphosphate kinase activity	GO:0072502;GO:0009620//response to fungus;GO:0009617//response to bacterium;GO:0002252;GO:0032958//inositol phosphate biosynthetic process	gi|351727917|ref|NP_001237433.1|/0/inositol pentakisphosphate 2-kinase [Glycine max]
Glyma.09G011100	-	-	-	-	-	-	-	-	-1.507	0.0012327	1825	ko00562//Inositol phosphate metabolism;ko00053//Ascorbate and aldarate metabolism;ko04070//Phosphatidylinositol signaling system;ko01100//Metabolic pathways;ko01110//Biosynthesis of secondary metabolites	GO:0044444;GO:0016020//membrane	GO:0070456	GO:0019852//L-ascorbic acid metabolic process;GO:0046488//phosphatidylinositol metabolic process;GO:0009628//response to abiotic stimulus;GO:0006950//response to stress	gi|734420434|gb|KHN40821.1|;gi|947087895|gb|KRH36560.1|/0;4.89277e-157/Inositol monophosphatase 3 [Glycine soja] ;hypothetical protein GLYMA_09G011100 [Glycine max]
Glyma.11G238800	-2.591	2.28E-23	-3.016	4.24E-13	-2.786	2.93E-18	-2.887	2.17E-14	-3.459	2.19E-18	1945	ko00562//Inositol phosphate metabolism;ko01100//Metabolic pathways;ko01110//Biosynthesis of secondary metabolites	-	GO:0016872//intramolecular lyase activity	GO:0009642//response to light intensity;GO:0009620//response to fungus;GO:0000302//response to reactive oxygen species;GO:0006020//inositol metabolic process;GO:0009617//response to bacterium;GO:0006658//phosphatidylserine metabolic process;GO:0002252;GO:0007275//multicellular organismal development;GO:0003006//developmental process involved in reproduction;GO:0032958//inositol phosphate biosynthetic process	gi|14764466|gb|AAK72098.1|/0/myo-inositol-1-phosphate synthase [Glycine max]
Glyma.06G213600	-5.258	#######	-5.849	1.29E-43	-5.948	1.71E-44	-4.886	1.05E-14	-4.317	6.58E-13	5148	ko00562//Inositol phosphate metabolism;ko03008//Ribosome biogenesis in eukaryotes;ko04070//Phosphatidylinositol signaling system;ko01100//Metabolic pathways;ko04144//Endocytosis;ko04712//Circadian rhythm - plant	-	-	-	gi|947106465|gb|KRH54848.1|/1.00594e-90/hypothetical protein GLYMA_06G213600 [Glycine max]

20 × 20

Supplementary Table S4: DEGs involved in photosynthesis

<table>
<tr><th>GeneID</th><th>log2Ratio (2mlpa-1/ 2MWT-1)</th><th>P-value</th><th>log2Ratio (2mlpa-2/ 2MWT-2)</th><th>P-value</th><th>log2Ratio (2mlpa-3/ 2MWT-3)</th><th>P-value</th><th>log2Ratio (2mlpa-4/ 2MWT-4)</th><th>P-value</th><th>log2Ratio (2mlpa-5/ 2MWT-5)</th><th>P-value</th><th>Length</th><th>Pathway</th><th>GO Component</th><th>GO Function</th><th>GO Process</th><th>Blast nr</th></tr>
<tr><td>Glyma.01 G050900</td><td>-2.3292</td><td>6.20E-06</td><td>-</td><td>-</td><td>-</td><td>-</td><td>-</td><td>-</td><td>-</td><td>-</td><td>936</td><td>ko00195//Photosynthesis;ko01100//Metabolic pathways</td><td>GO:0031976;GO:0009523//photosystem II</td><td>GO:0046872//metal ion binding;GO:0009055//electron carrier activity</td><td>GO:0022900//electron transport chain</td><td>gi|571434179|ref|XP_003518016.2|/6.70419e-155/PREDICTED: photosynthetic NDH subunit of lumenal location 2, chloroplastic [Glycine max]</td></tr>
<tr><td>Glyma.01 G095900</td><td>-1.5306</td><td>4.04E-05</td><td>3.87635</td><td>1.67E-18</td><td>3.59217</td><td>1.29E-08</td><td>2.51069</td><td>1.12E-05</td><td>-</td><td>-</td><td>293</td><td>ko00195//Photosynthesis;ko01100//Metabolic pathways</td><td>GO:0009523//photosystem II;GO:0009507//chloroplast</td><td>-</td><td>GO:0015979//photosynthesis</td><td>gi|947127748|gb|KRH75602.1|/3.04979e-23/hypothetical protein GLYMA_01G095900 [Glycine max]</td></tr>
<tr><td>Glyma.01 G153500</td><td>-1.5333</td><td>0.00125</td><td>3.85582</td><td>3.35E-13</td><td>4.15308</td><td>2.55E-09</td><td>2.95855</td><td>2.13E-05</td><td>-</td><td>-</td><td>750</td><td>ko00195//Photosynthesis;ko01100//Metabolic pathways</td><td>GO:0009521;GO:0031224//intrinsic component of membrane;GO:0009534//chloroplast thylakoid</td><td>GO:0046914//transition metal ion binding;GO:0009055//electron carrier activity;GO:0003824//catalytic activity</td><td>GO:0009767//photosynthetic electron transport chain</td><td>gi|947128606|gb|KRH76460.1|/0/hypothetical protein GLYMA_01G153500 [Glycine max]</td></tr>
<tr><td>Glyma.05 G073600</td><td>-</td><td>-</td><td>8.09189</td><td>2.08E-11</td><td>-</td><td>-</td><td>-</td><td>-</td><td>-</td><td>-</td><td>426</td><td>ko00195//Photosynthesis;ko01100//Metabolic pathways</td><td>GO:0043231//intracellular membrane-bounded organelle</td><td>GO:0009055//electron carrier activity</td><td>GO:0009767//photosynthetic electron transport chain</td><td>gi|947109303|gb|KRH57629.1|/2.58432e-50/hypothetical protein GLYMA_05G073600 [Glycine max]</td></tr>
<tr><td>Glyma.06 G217900</td><td>-2.0852</td><td>1.18E-07</td><td>3.69572</td><td>1.00E-14</td><td>4.21351</td><td>4.04E-15</td><td>3.21924</td><td>5.21E-08</td><td>-</td><td>-</td><td>507</td><td>ko00195//Photosynthesis;ko01100//Metabolic pathways</td><td>GO:0009521;GO:0031224//intrinsic component of membrane;GO:0009534//chloroplast thylakoid</td><td>GO:0046914//transition metal ion binding;GO:0009055//electron carrier activity;GO:0003824//catalytic activity</td><td>GO:0009767//photosynthetic electron transport chain</td><td>gi|947106520|gb|KRH54903.1|/3.69129e-118/hypothetical protein GLYMA_06G217900 [Glycine max]</td></tr>
<tr><td>Glyma.06 G224500</td><td>-</td><td>-</td><td>4.46157</td><td>1.14E-19</td><td>7.11534</td><td>1.38E-10</td><td>-</td><td>-</td><td>-</td><td>-</td><td>279</td><td>ko00195//Photosynthesis;ko01100//Metabolic pathways</td><td>GO:0009521;GO:0031224//intrinsic component of membrane;GO:0009534//chloroplast thylakoid</td><td>GO:0046906//tetrapyrrole binding;GO:0046872//metal ion binding;GO:0051536//iron-sulfur cluster binding;GO:0003824//catalytic activity</td><td>GO:0006464//cellular protein modification process;GO:0044710</td><td>gi|947106622|gb|KRH55005.1|/1.83252e-63/hypothetical protein GLYMA_06G224500, partial [Glycine max]</td></tr>
<tr><td>Glyma.06 G229000</td><td>-2.3822</td><td>0.00074</td><td>2.7897</td><td>6.89E-09</td><td>4.99319</td><td>2.86E-10</td><td>-</td><td>-</td><td>-</td><td>-</td><td>417</td><td>ko00195//Photosynthesis;ko00190//Oxidative phosphorylation; ko01100//Metabolic pathways</td><td>GO:0031224//intrinsic component of membrane;GO:0033177//proton-transporting two-sector ATPase complex, proton-transporting domain;GO:0009534//chloroplast thylakoid</td><td>GO:0015077//monovalent inorganic cation transmembrane transporter activity;GO:0005488</td><td>GO:0015988//energy coupled proton transmembrane transport, against electrochemical gradient;GO:0006754//ATP biosynthetic process</td><td>gi|947106696|gb|KRH55079.1|/4.411e-37/hypothetical protein GLYMA_06G229000 [Glycine max]</td></tr>
<tr><td>Glyma.07 G143800</td><td>-</td><td>-</td><td>5.05164</td><td>5.42E-27</td><td>6.50017</td><td>9.31E-13</td><td>-</td><td>-</td><td>-</td><td>-</td><td>348</td><td>ko00195//Photosynthesis;ko01100//Metabolic pathways</td><td>GO:0009521;GO:0031224//intrinsic component of membrane;GO:0009534//chloroplast thylakoid</td><td>GO:0046906//tetrapyrrole binding;GO:0046872//metal ion binding;GO:0051536//iron-sulfur cluster binding;GO:0003824//catalytic activity</td><td>GO:0006464//cellular protein modification process;GO:0044710</td><td>gi|734395058|gb|KHN28788.1|/1.56572e-70/Photosystem I P700 chlorophyll a apoprotein A1 [Glycine soja]</td></tr>
<tr><td>Glyma.08 G281300</td><td>-</td><td>-</td><td>3.99838</td><td>1.12E-19</td><td>5.44119</td><td>4.16E-20</td><td>2.99514</td><td>0.000186</td><td>-</td><td>-</td><td>324</td><td>ko00195//Photosynthesis;ko01100//Metabolic pathways</td><td>GO:0031224//intrinsic component of membrane;GO:0034357;GO:0009536//plastid</td><td>GO:0046906//tetrapyrrole binding</td><td>GO:0006464//cellular protein modification process;GO:002290</td><td>gi|947096990|gb|KRH45575.1|/1.40663e-74/hypothetical protein GLYMA_08G281300</td></tr>
</table>

															0//electron transport chain	[Glycine max]
Glyma.08G363500	-1.3542	0.00236	4.01677	3.47E-16	3.98983	2.10E-09	2.57728	0.000109	-	-	621	ko00195//Photosynthesis;ko01100//Metabolic pathways	GO:0034357	-	-	gi|947098318|gb|KRH46903.1|/9.44696e-19/hypothetical protein GLYMA_08G363500 [Glycine max]
Glyma.11G162200	-	-	3.7818	1.29E-19	3.51122	2.59E-09	1.71363	0.000303	-	-	699	ko00195//Photosynthesis;ko01100//Metabolic pathways	GO:0009521;GO:0031224//intrinsic component of membrane	GO:0043169//cation binding;GO:0009055//electron carrier activity	GO:0009767//photosynthetic electron transport chain	gi|947081354|gb|KRH30143.1|/9.45488e-155/hypothetical protein GLYMA_11G162200, partial [Glycine max]
Glyma.12G169400	-1.3582	0.00317	-	-	-	-	-	-	-	-	1122	ko00195//Photosynthesis	GO:0009536//plastid	GO:0005488	-	gi|947077519|gb|KRH26359.1|/3.82053e-90/hypothetical protein GLYMA_12G169400 [Glycine max]
Glyma.12G202500	-	-	-	-	1.34376	0.0005	1.30934	4.46E-05	-	-	1010	ko00195//Photosynthesis;ko01100//Metabolic pathways	GO:0009534//chloroplast thylakoid	-	GO:0019684//photosynthesis, light reaction	gi|356543813|ref|XP_003540354.1|/6.4337e-96/PREDICTED: photosystem II repair protein PSB27-H1, chloroplastic-like [Glycine max]
Glyma.12G232700	-	-	5.52159	6.31E-28	5.20226	1.29E-10	3.29685	1.83E-06	-	-	429	ko00195//Photosynthesis;ko01100//Metabolic pathways	GO:0031224//intrinsic component of membrane;GO:0009523//photosystem II;GO:0009534//chloroplast thylakoid	GO:0046914//transition metal ion binding;GO:0032550;GO:0016421//CoA carboxylase activity;GO:0046906//tetrapyrrole binding	GO:0015937//coenzyme A biosynthetic process;GO:0006351//transcription, DNA-templated;GO:0022900//electron transport chain;GO:0009765//photosynthesis, light harvesting;GO:0006631//fatty acid metabolic process	gi|947078548|gb|KRH27388.1|/1.04111e-99/hypothetical protein GLYMA_12G232700 [Glycine max]
Glyma.13G028200	-	-	3.83427	1.88E-21	2.99907	6.70E-06	1.79323	0.000164	-	-	816	ko00195//Photosynthesis;ko01100//Metabolic pathways	GO:0009521;GO:0031224//intrinsic component of membrane;GO:0009534//chloroplast thylakoid	GO:0046914//transition metal ion binding;GO:0009055//electron carrier activity;GO:0003824//catalytic activity	GO:0006950//response to stress;GO:0009767//photosynthetic electron transport chain	gi|947069046|gb|KRH17937.1|/0/hypothetical protein GLYMA_13G028200, partial [Glycine max]
Glyma.13G088500	-	-	3.65135	7.24E-09	5.60027	5.33E-11	-	-	-	-	240	ko00195//Photosynthesis;ko01100//Metabolic pathways	GO:0009521;GO:0009534//chloroplast thylakoid	GO:0043169//cation binding;GO:0051536//iron-sulfur cluster binding;GO:0050136//NADH dehydrogenase (quinone) activity	GO:0009767//photosynthetic electron transport chain;GO:0022904//respiratory electron transport chain	gi|947070006|gb|KRH18897.1|/6.89749e-54/hypothetical protein GLYMA_13G088500 [Glycine max]
Glyma.15G114600	-1.4907	0.00038	3.91924	1.25E-19	5.49734	2.01E-20	3.02382	2.57E-05	-	-	1190	ko00195//Photosynthesis;ko01100//Metabolic pathways	GO:0031224//intrinsic component of membrane;GO:0034357;GO:0009534//chloroplast thylakoid	GO:0009055//electron carrier activity;GO:0003824//catalytic activity	GO:0022900//electron transport chain	gi|947062266|gb|KRH11527.1|/2.61757e-86/hypothetical protein GLYMA_15G114600 [Glycine max]
Glyma.15G126100	-	-	4.07773	5.53E-20	4.93214	4.03E-10	3.73562	1.91E-07	-	-	363	ko00195//Photosynthesis;ko01100//Metabolic pathways	GO:0009521;GO:0031224//intrinsic component of membrane;GO:0009534//chloroplast thylakoid	-	GO:0044237//cellular metabolic process	gi|91214162|ref|YP_538784.1|/4.7294e-24/photosystem I subunit IX [Glycine max]
Glyma.15G208300	-	-	3.13165	5.67E-07	-	-	-	-	-	-	273	ko00195//Photosynthesis;ko01100//Metabolic pathways	GO:0009521;GO:0031224//intrinsic component of membrane;GO:0009534//chloroplast thylakoid	-	GO:0006091//generation of precursor metabolites and energy	gi|763745629|gb|KJB13068.1|/1.93912e-15/hypothetical protein B456_002G055100 [Gossypium raimondii]

20 × 20

<table>
<tr><td>Glyma.15G248600</td><td>-</td><td>-</td><td>3.14569</td><td>2.14E-09</td><td>2.81734</td><td>7.23E-05</td><td>-</td><td>-</td><td>-</td><td>-</td><td>342</td><td>ko00195//Photosynthesis;ko01100//Metabolic pathways</td><td>GO:0009536//plastid;GO:0009523//photosystem II</td><td>-</td><td>GO:0044237//cellular metabolic process</td><td>gi|947064317|gb|KRH13578.1|/5.67331e-75/hypothetical protein GLYMA_15G248600 [Glycine max]</td></tr>
<tr><td>Glyma.17G186400</td><td>-</td><td>-</td><td>3.90764</td><td>5.61E-08</td><td>5.2486</td><td>1.19E-08</td><td>-</td><td>-</td><td>-</td><td>-</td><td>207</td><td>ko00195//Photosynthesis;ko01100//Metabolic pathways</td><td>GO:0009521;GO:0031224//intrinsic component of membrane;GO:0009534//chloroplast thylakoid</td><td>GO:0043168//anion binding</td><td>GO:0044237//cellular metabolic process;GO:0031647//regulation of protein stability</td><td>gi|947055325|gb|KRH04778.1|/3.72484e-44/hypothetical protein GLYMA_17G186400 [Glycine max]</td></tr>
<tr><td>Glyma.19G054200</td><td>-2.0822</td><td>0.00014</td><td>2.22477</td><td>0.00014</td><td>4.32237</td><td>2.30E-06</td><td>-</td><td>-</td><td>-</td><td>-</td><td>318</td><td>ko00195//Photosynthesis;ko00190//Oxidative phosphorylation;ko01100//Metabolic pathways</td><td>GO:0031224//intrinsic component of membrane;GO:0033177//proton-transporting two-sector ATPase complex, proton-transporting domain;GO:0009534//chloroplast thylakoid</td><td>GO:0019829//cation-transporting ATPase activity;GO:0015077//monovalent inorganic cation transmembrane transporter activity</td><td>GO:0015986//ATP synthesis coupled proton transport</td><td>gi|947044351|gb|KRG93980.1|/2.03094e-59/hypothetical protein GLYMA_19G054200 [Glycine max]</td></tr>
<tr><td>Glyma.20G020600</td><td>1.57644</td><td>0.00398</td><td>-</td><td>-</td><td>-</td><td>-</td><td>-</td><td>-</td><td>-</td><td>-</td><td>925</td><td>ko00195//Photosynthesis</td><td>-</td><td>-</td><td>-</td><td>gi|571563659|ref|XP_006605511.1|;gi|955391318|ref|XP_014627811.1|/1.21678e-120;1.45013e-102/PREDICTED: , chloroplastic-like isoform X1 [Glycine max];PREDICTED: uncharacterized protein LOC100797820 isoform X2 [Glycine max]</td></tr>
<tr><td>Glyma.05G099200</td><td>-2.654</td><td>3.46E-07</td><td>2.68556</td><td>1.85E-09</td><td>4.99906</td><td>1.21E-11</td><td>-</td><td>-</td><td>5.82843</td><td>3.79E-06</td><td>271</td><td>ko00195//Photosynthesis;ko00190//Oxidative phosphorylation;ko01100//Metabolic pathways</td><td>GO:0031224//intrinsic component of membrane;GO:0033177//proton-transporting two-sector ATPase complex, proton-transporting domain;GO:0009534//chloroplast thylakoid;GO:0009526//plastid envelope</td><td>GO:0015077//monovalent inorganic cation transmembrane transporter activity;GO:0005488</td><td>GO:0019684//photosynthesis, light reaction;GO:0015988//energy coupled proton transmembrane transport, against electrochemical gradient;GO:0006351//transcription, DNA-templated;GO:0006754//ATP biosynthetic process</td><td>gi|947109669|gb|KRH57995.1|/7.86592e-52/hypothetical protein GLYMA_05G099200, partial [Glycine max]</td></tr>
<tr><td>Glyma.04G095000</td><td>-2.2576</td><td>1.16E-07</td><td>3.87018</td><td>1.64E-15</td><td>4.11517</td><td>3.22E-14</td><td>3.56279</td><td>9.79E-11</td><td>4.10345</td><td>1.81E-06</td><td>711</td><td>ko00195//Photosynthesis;ko01100//Metabolic pathways</td><td>GO:0009521;GO:0031224//intrinsic component of membrane;GO:0043231//intracellular membrane-bounded organelle</td><td>GO:0043169//cation binding;GO:0009055//electron carrier activity</td><td>GO:0009767//photosynthetic electron transport chain</td><td>gi|947113933|gb|KRH62235.1|/1.4233e-107/hypothetical protein GLYMA_04G095000 [Glycine max]</td></tr>
<tr><td>Glyma.12G231900</td><td>-2.3851</td><td>8.45E-10</td><td>3.23154</td><td>1.60E-14</td><td>4.88738</td><td>3.62E-18</td><td>2.43866</td><td>0.000212</td><td>3.81275</td><td>1.12E-08</td><td>384</td><td>ko00195//Photosynthesis;ko00190//Oxidative phosphorylation;ko01100//Metabolic pathways</td><td>GO:0031224//intrinsic component of membrane;GO:0033177//proton-transporting two-sector ATPase complex, proton-transporting domain;GO:0009534//chloroplast thylakoid</td><td>GO:0032550;GO:0019829//cation-transporting ATPase activity;GO:0015077//monovalent inorganic cation transmembrane transporter activity</td><td>GO:0006818//hydrogen transport;GO:0006754//ATP biosynthetic process</td><td>gi|947078540|gb|KRH27380.1|/1.10934e-66/hypothetical protein GLYMA_12G231900, partial [Glycine max]</td></tr>
<tr><td>Glyma.19G109600</td><td>-2.573</td><td>1.09E-07</td><td>2.83652</td><td>3.41E-16</td><td>4.74518</td><td>2.94E-15</td><td>-</td><td>-</td><td>3.76104</td><td>1.27E-07</td><td>423</td><td>ko00195//Photosynthesis;ko00190//Oxidative phosphorylation;ko01100//Metabolic pathways</td><td>GO:0031224//intrinsic component of membrane;GO:0033177//proton-transporting two-sector ATPase complex,</td><td>GO:0019829//cation-transporting ATPase activity;GO:0015077//monovalent inorganic cation</td><td>GO:0015988//energy coupled proton transmembrane transport, against electrochemical gradient;GO:001598</td><td>gi|947045161|gb|KRG94790.1|/9.26156e-42/hypothetical protein GLYMA_19G109600 [Glycine max]</td></tr>
</table>

20 × 20

													proton-transporting domain;GO:0009534//chloroplast thylakoid;GO:0009526//plastid envelope	transmembrane transporter activity;GO:0005488	6//ATP synthesis coupled proton transport	
Glyma.U016600	-	-	4.76174	6.26E-31	6.62145	1.61E-16	2.84707	0.000285	3.61362	0.00092	375	ko00195//Photosynthesis;ko01100//Metabolic pathways	GO:0009521;GO:0031224//intrinsic component of membrane;GO:0009534//chloroplast thylakoid	GO:0046906//tetrapyrrole binding;GO:0046872//metal ion binding;GO:0051536//iron-sulfur cluster binding;GO:0003824//catalytic activity	GO:0006464//cellular protein modification process;GO:0044710	gi|947039131|gb|KRG88966.1|/8.09939e-73/hypothetical protein GLYMA_U016600, partial [Glycine max]
Glyma.12G232900	-2.3642	7.63E-21	3.57447	1.91E-19	5.67099	5.36E-20	2.3048	0.000369	3.57215	7.80E-09	1912	ko00195//Photosynthesis;ko01100//Metabolic pathways	GO:0034357;GO:0009534//chloroplast thylakoid;GO:0016021//integral component of membrane	GO:0046906//tetrapyrrole binding;GO:0046914//transition metal ion binding	GO:0044237//cellular metabolic process;GO:0044710	gi|91214155|ref|YP_538777.1|/0/cytochrome f [Glycine max]
Glyma.12G232500	-2.3405	3.08E-08	4.62078	2.89E-23	5.63807	2.47E-16	3.32027	1.64E-07	3.17592	0.00038	1023	ko00195//Photosynthesis;ko01100//Metabolic pathways	GO:0034357;GO:0031224//intrinsic component of membrane;GO:0009532//plastid stroma;GO:0009534//chloroplast thylakoid	GO:0009055//electron carrier activity;GO:0043169//cation binding	GO:0019684//photosynthesis, light reaction;GO:0006351//transcription, DNA-templated;GO:0006412//translation;GO:0022900//electron transport chain;GO:0010038//response to metal ion;GO:0043623//cellular protein complex assembly;GO:0055076//transition metal ion homeostasis;GO:0009416//response to light stimulus	gi|11465978|ref|NP_054520.1|/6.06418e-18/cytochrome b6/f complex subunit V [Nicotiana tabacum]
Glyma.01G058600	-1.8677	1.42E-05	3.96422	1.97E-18	5.64774	4.80E-19	3.38744	2.16E-05	2.94009	0.00323	463	ko00195//Photosynthesis;ko01100//Metabolic pathways	GO:0031224//intrinsic component of membrane;GO:0034357;GO:0009534//chloroplast thylakoid	GO:0046906//tetrapyrrole binding;GO:0046914//transition metal ion binding;GO:0009055//electron carrier activity;GO:0003824//catalytic activity	GO:0022900//electron transport chain	gi|947127189|gb|KRH75043.1|/2.29292e-78/hypothetical protein GLYMA_01G058600 [Glycine max]
Glyma.11G114700	-1.6986	9.96E-06	3.78507	4.79E-17	4.29086	4.91E-13	2.74467	1.36E-07	2.5227	0.00244	408	ko00195//Photosynthesis;ko01100//Metabolic pathways	GO:0031224//intrinsic component of membrane;GO:0034357;GO:0009536//plastid	GO:0046906//tetrapyrrole binding	GO:0006464//cellular protein modification process;GO:0022900//electron transport chain	gi|947080624|gb|KRH29413.1|/2.22449e-94/hypothetical protein GLYMA_11G114700 [Glycine max]
Glyma.09G090100	-1.3311	0.00415	4.85917	5.25E-26	6.41069	2.84E-13	4.45229	9.97E-08	2.52239	0.00569	558	ko00195//Photosynthesis;ko01100//Metabolic pathways	GO:0009521;GO:0031224//intrinsic component of membrane;GO:0009536//plastid	-	GO:0044237//cellular metabolic process	gi|947089135|gb|KRH37800.1|/7.49414e-119/hypothetical protein GLYMA_09G090100, partial [Glycine max]
Glyma.12G096200	-2.6199	9.59E-15	2.01925	2.75E-08	4.32398	4.17E-12	-	-	2.46085	0.0001	318	ko00195//Photosynthesis;ko00190//Oxidative phosphorylation;ko01100//Metabolic pathways	GO:0031224//intrinsic component of membrane;GO:0033177//proton-transporting two-sector ATPase complex, proton-transporting	GO:0019829//cation-transporting ATPase activity;GO:0015077//monovalent inorganic cation transmembrane	GO:0015986//ATP synthesis coupled proton transport	gi|947076502|gb|KRH25342.1|/4.62789e-71/hypothetical protein GLYMA_12G096200, partial [Glycine max]

20 × 20

													domain;GO:0009534//chloroplast thylakoid	transporter activity		
Glyma.12G232000	-1.7611	9.52E-09	3.48479	3.66E-18	4.64109	5.21E-17	2.41548	3.46E-05	2.15131	2.10E-05	2784	ko00195//Photosynthesis;ko00190//Oxidative phosphorylation;ko01100//Metabolic pathways	GO:0033178//proton-transporting two-sector ATPase complex, catalytic domain;GO:0019866//organelle inner membrane;GO:0009534//chloroplast thylakoid	GO:0032550;GO:0019829//cation-transporting ATPase activity;GO:0015078//hydrogen ion transmembrane transporter activity	GO:0015988//energy coupled proton transmembrane transport, against electrochemical gradient;GO:0015986//ATP synthesis coupled proton transport	gi|947078541|gb|KRH27381.1|/0/hypothetical protein GLYMA_12G232000 [Glycine max]
Glyma.05G074900	-1.8033	1.74E-06	3.92446	4.56E-17	4.21531	2.83E-13	3.45592	1.25E-09	2.06823	0.00579	439	ko00195//Photosynthesis;ko01100//Metabolic pathways	GO:0016020//membrane;GO:0044424	-	GO:0044237//cellular metabolic process	gi|947109318|gb|KRH57644.1|/8.28695e-19/hypothetical protein GLYMA_05G074900 [Glycine max]
Glyma.04G066000	-	-	-	-	-	-	-	-	-1.0766	0.00597	1289	ko00195//Photosynthesis;ko00190//Oxidative phosphorylation;ko01100//Metabolic pathways	GO:0044434;GO:0044425;GO:0009579//thylakoid	-	GO:0044765	gi|351722297|ref|NP_001237239.1|/7.45658e-115/uncharacterized protein LOC100527009 [Glycine max]
Glyma.18G241700	-	-	-	-	-	-	1.09562	0.00037	-1.1212	0.00138	1010	ko00195//Photosynthesis;ko01100//Metabolic pathways	GO:0009526//plastid envelope;GO:0031976;GO:0009570//chloroplast stroma;GO:0009522//photosystem I	-	GO:0044237//cellular metabolic process	gi|351724071|ref|NP_001236277.1|/7.26819e-124/uncharacterized protein LOC100500317 [Glycine max]
Glyma.11G077600	-	-	-	-	-	-	-	-	-1.1864	0.00112	1442	ko00195//Photosynthesis;ko01100//Metabolic pathways	GO:0009526//plastid envelope;GO:0031976;GO:0044436;GO:0005576//extracellular region	GO:0009055//electron carrier activity;GO:0008187//poly-pyrimidine tract binding;GO:0003824//catalytic activity	GO:0009617//response to bacterium;GO:0022900//electron transport chain	gi|734422972|gb|KHN41893.1|/0/Ferredoxin--NADP reductase, leaf isozyme, chloroplastic [Glycine soja]
Glyma.16G127300	-	-	-	-	-	-	-	-	-1.1959	0.0074	1628	ko00195//Photosynthesis;ko01100//Metabolic pathways	GO:0009526//plastid envelope;GO:0031976;GO:0044436;GO:0005576//extracellular region	GO:0009055//electron carrier activity;GO:0008187//poly-pyrimidine tract binding;GO:0003824//catalytic activity;GO:0016731//oxidoreductase activity, acting on iron-sulfur proteins as donors, NAD or NADP as acceptor	GO:0009617//response to bacterium;GO:0022900//electron transport chain	gi|356559282|ref|XP_003547929.1|;gi|947058656|gb|KRH08062.1|/0;0/PREDICTED: ferredoxin--NADP reductase, leaf isozyme, chloroplastic [Glycine max];hypothetical protein GLYMA_16G127300 [Glycine max]
Glyma.12G089200	-	-	-	-	-	-	-	-	-1.2263	0.00141	784	ko00195//Photosynthesis	-	GO:0051536//iron-sulfur cluster binding;GO:0043169//cation binding	GO:0006091//generation of precursor metabolites and energy	gi|351721230|ref|NP_001238738.1|/2.55515e-98/uncharacterized protein LOC100499711 [Glycine max]
Glyma.01G238000	-	-	-	-	-	-	-	-	-1.2355	0.00761	818	ko00195//Photosynthesis	GO:0009536//plastid	GO:0051536//iron-sulfur cluster binding;GO:0043169//cation binding	GO:0006091//generation of precursor metabolites and energy	gi|351725193|ref|NP_001236060.1|/2.57228e-92/uncharacterized protein LOC100500300 [Glycine max]
Glyma.20G110800	-	-	-	-	-	-	-	-	-1.3455	0.00089	988	ko00195//Photosynthesis	-	GO:0051536//iron-sulfur cluster binding;GO:0043169//cation binding	GO:0006091//generation of precursor metabolites and energy	gi|351727909|ref|NP_001236409.1|/2.3486e-111/uncharacterized protein LOC100500583 [Glycine max]

20 × 20

Glyma.13 G302900	-	-	-	-	-	-	-	-	-1.4536	0.00261	1060	ko00195//Photosynthesis;ko01100//Metabolic pathways	GO:0031224//intrinsic component of membrane;GO:0009526//plastid envelope;GO:0031976;GO:0034357	GO:0043169//cation binding;GO:0015078//hydrogen ion transmembrane transporter activity;GO:0051536//iron-sulfur cluster binding;GO:0009055//electron carrier activity;GO:0052880	GO:1990066;GO:0044710;GO:0009617//response to bacterium;GO:0044237//cellular metabolic process;GO:0015992//proton transport	gi|947073587|gb|KRH22478.1|;gi|734413144|gb|KHN36589.1|/1.21441e-85;1.16363e-156/hypothetical protein GLYMA_13G302900 [Glycine max];Cytochrome b6-f complex iron-sulfur subunit, chloroplastic [Glycine soja]
Glyma.20 G098500	-	-	-	-	-	-	-	-	-1.4605	0.00043	1412	ko00195//Photosynthesis;ko01100//Metabolic pathways	GO:0009523//photosystem II	GO:0046872//metal ion binding	GO:0044237//cellular metabolic process	gi|947040828|gb|KRG90552.1|/0/hypothetical protein GLYMA_20G098500 [Glycine max]
Glyma.10 G042100	-	-	-	-	-	-	-	-	-1.4816	0.00025	1020	ko00195//Photosynthesis;ko01100//Metabolic pathways	GO:0044434;GO:0009522//photosystem I	-	-	gi|351727413|ref|NP_001236392.1|/1.41727e-61/uncharacterized protein LOC100499814 [Glycine max]
Glyma.08 G126800	-	-	-	-	-	-	-	-	-1.5198	2.97E-05	1086	ko00195//Photosynthesis	GO:0009536//plastid	GO:0043169//cation binding;GO:0051536//iron-sulfur cluster binding	GO:0022900//electron transport chain	gi|351720711|ref|NP_001235138.1|/1.61599e-102/uncharacterized protein LOC100305932 [Glycine max]
Glyma.04 G215800	-	-	-	-	-	-	-	-	-1.5303	0.00103	1219	ko00195//Photosynthesis;ko01100//Metabolic pathways	GO:0009522//photosystem I;GO:0009534//chloroplast thylakoid;GO:0034357;GO:0009507//chloroplast;GO:0009579//thylakoid	GO:0005515//protein binding	GO:0006007//glucose catabolic process;GO:0006470//protein dephosphorylation;GO:0000097//sulfur amino acid biosynthetic process;GO:0009767//photosynthetic electron transport chain	gi|947115793|gb|KRH64095.1|;gi|947115794|gb|KRH64096.1|;gi|947115791|gb|KRH64093.1|;gi|351722715|ref|NP_001235206.1|/1.61686e-78;4.64534e-50;3.80265e-116;6.1549e-101/hypothetical protein GLYMA_04G215800 [Glycine max];hypothetical protein GLYMA_04G215800 [Glycine max];hypothetical protein GLYMA_04G215800 [Glycine max];uncharacterized protein LOC100499729 [Glycine max]
Glyma.15 G016300	-	-	-	-	-	-	-	-	-1.5452	0.00413	1316	ko00195//Photosynthesis;ko01100//Metabolic pathways	GO:0031976;GO:0009570//chloroplast stroma;GO:0009522//photosystem I	-	GO:0044237//cellular metabolic process	gi|358248194|ref|NP_001240092.1|/1.2461e-69/uncharacterized protein LOC100776789 [Glycine max]
Glyma.06 G150300	-	-	-	-	-	-	-	-	-1.5564	7.67E-05	1140	ko00195//Photosynthesis;ko01100//Metabolic pathways	GO:0009521;GO:0009534//chloroplast thylakoid;GO:0034357	GO:0005515//protein binding	GO:0044237//cellular metabolic process	gi|947105477|gb|KRH53860.1|;gi|351725111|ref|NP_001236569.1|/2.80026e-87;1.3001e-109/hypothetical protein GLYMA_06G150300 [Glycine max];uncharacterized protein LOC100306036 [Glycine max]
Glyma.08 G173700	-	-	-	-	-	-	-	-	-1.5775	2.26E-05	874	ko00195//Photosynthesis;ko01100//Metabolic pathways	GO:0009523//photosystem II;GO:0009534//chloroplast thylakoid	-	GO:0010207//photosystem II assembly	gi|351722140|ref|NP_001235442.1|/2.65135e-85/uncharacterized protein LOC100499745 [Glycine max]

20 × 20

Glyma.01 G180800	-	-	-	-	-	-	-	-	-1.5797	4.72E-06	1339	ko00195//Photosynthesis;ko01100//Metabolic pathways	GO:0031224//intrinsic component of membrane;GO:0009523//photosystem II;GO:0019867//outer membrane	GO:0046872//metal ion binding	GO:0042548//regulation of photosynthesis, light reaction	gi|359806573|ref|NP_001241522.1|/0/uncharacterized protein LOC100820458 [Glycine max]
Glyma.05 G172300	-	-	-	-	-	-	-	-	-1.5846	7.39E-06	1447	ko00195//Photosynthesis;ko01100//Metabolic pathways	GO:0044425	-	-	gi|359806150|ref|NP_001240940.1|/3.50724e-96/uncharacterized protein LOC100779397 [Glycine max]
Glyma.09 G250800	-	-	-	-	-	-	-	-	-1.6018	2.64E-05	1307	ko00195//Photosynthesis;ko01100//Metabolic pathways	GO:0009526//plastid envelope;GO:0031976;GO:0009570//chloroplast stroma;GO:0009522//photosystem I	-	GO:0044237//cellular metabolic process	gi|351726365|ref|NP_001236868.1|/2.01874e-134/uncharacterized protein LOC100527240 [Glycine max]
Glyma.10 G032200	-	-	-	-	-	-	1.55529	2.58E-06	-1.6047	9.86E-06	1332	ko00195//Photosynthesis;ko01100//Metabolic pathways	GO:0016020//membrane;GO:0044424	-	-	gi|351721030|ref|NP_001236684.1|/2.08586e-35/uncharacterized protein LOC100305788 [Glycine max]
Glyma.15 G253700	-	-	-	-	-	-	-	-	-1.6737	7.91E-06	840	ko00195//Photosynthesis;ko01100//Metabolic pathways	GO:0009523//photosystem II;GO:0009534//chloroplast thylakoid	-	GO:0010207//photosystem II assembly	gi|351722402|ref|NP_001236219.1|/1.48811e-84/uncharacterized protein LOC100305752 [Glycine max]
Glyma.02 G047600	-	-	-	-	-	-	-	-	-1.6875	0.00759	1494	ko00195//Photosynthesis;ko01100//Metabolic pathways	GO:0009526//plastid envelope;GO:0031976;GO:0044436;GO:0005576//extracellular region	GO:0016731//oxidoreductase activity, acting on iron-sulfur proteins as donors, NAD or NADP as acceptor;GO:0009055//electron carrier activity;GO:0008187//poly-pyrimidine tract binding	GO:0009617//response to bacterium;GO:0022900//electron transport chain	gi|734312293|gb|KHN00471.1|/0/Ferredoxin--NADP reductase, leaf isozyme, chloroplastic [Glycine soja]
Glyma.04 G112800	-	-	-	-	-	-	-	-	-1.6901	3.70E-05	1366	ko00195//Photosynthesis;ko01100//Metabolic pathways	GO:0009522//photosystem I;GO:0009526//plastid envelope;GO:0031976	GO:0046906//tetrapyrrole binding	GO:0009767//photosynthetic electron transport chain;GO:0031647//regulation of protein stability;GO:0042548//regulation of photosynthesis, light reaction;GO:0006740//NADPH regeneration	gi|351727473|ref|NP_001236650.1|/6.49139e-99/uncharacterized protein LOC100305786 [Glycine max]
Glyma.11 G183300	-	-	-	-	-	-	-	-	-1.6983	0.0003	1620	ko00195//Photosynthesis	-	GO:0051536//iron-sulfur cluster binding;GO:0043169//cation binding	GO:0006091//generation of precursor metabolites and energy	gi|359807123|ref|NP_001241349.1|/2.88363e-100/uncharacterized protein LOC100785611 [Glycine max]
Glyma.10 G249000	-	-	-	-	-	-	-	-	-1.7228	9.50E-06	1706	ko00195//Photosynthesis;ko01100//Metabolic pathways	GO:0009526//plastid envelope;GO:0031976;GO:0009570//chloroplast stroma;GO:0009522//photosystem I	-	GO:0044237//cellular metabolic process	gi|351727030|ref|NP_001235355.1|/5.09919e-130/photosystem I subunit PsaD [Glycine max]
Glyma.15 G194300	-	-	-	-	-	-	-	-	-1.7228	2.15E-06	1577	ko00195//Photosynthesis;ko01100//Metabolic pathways	GO:0009521	GO:0046906//tetrapyrrole binding	GO:0044237//cellular metabolic process	gi|734325419|gb|KHN05287.1|/2.64334e-76/Photosystem I reaction center subunit psaK, chloroplastic [Glycine soja]

20 × 20

Glyma.20 G144700	-	-	-	-	-	-	1.38717	1.16E-05	-1.7241	9.00E-06	2825	ko00195//Photosynthesis;ko01100//Metabolic pathways	GO:0009526//plastid envelope;GO:0031976;GO:0009570//chloroplast stroma;GO:0009522//photosystem I	-	GO:0044237//cellular metabolic process	gi|734361807|gb|KHN15781.1|;gi|947041551|gb|KRG91275.1|/4.18381e-149;2.80762e-107/Photosystem I reaction center subunit II, chloroplastic [Glycine soja] ;hypothetical protein GLYMA_20G144700 [Glycine max]
Glyma.13 G299200	-	-	-	-	-	-	-	-	-1.7771	4.73E-06	982	ko00195//Photosynthesis;ko01100//Metabolic pathways	-	-	GO:0019684//photosynthesis, light reaction	gi|356549934|ref|XP_003543345.1|/3.90749e-87/PREDICTED: photosystem II repair protein PSB27-H1, chloroplastic-like [Glycine max]
Glyma.13 G127200	-	-	-	-	-	-	-	-	-1.81	0.00045	1027	ko00195//Photosynthesis;ko01100//Metabolic pathways	GO:0009523//photosystem II	-	GO:0044237//cellular metabolic process	gi|734438132|gb|KHN48946.1|/1.42389e-120/Photosystem II reaction center PSB28 protein, chloroplastic [Glycine soja]
Glyma.18 G114900	-	-	-	-	-	-	-	-	-1.8659	0.00807	1323	ko00195//Photosynthesis;ko01100//Metabolic pathways	GO:0009523//photosystem II;GO:0009534//chloroplast thylakoid	GO:0046872//metal ion binding	GO:0044237//cellular metabolic process	gi|734394466|gb|KHN28540.1|/0/Oxygen-evolving enhancer protein 2, chloroplastic [Glycine soja]
Glyma.05 G022900	-	-	-	-	-	-	-	-	-1.8739	1.98E-07	1212	ko00195//Photosynthesis;ko01100//Metabolic pathways	GO:0009522//photosystem I	-	-	gi|356511909|ref|XP_003524664.1|/1.03197e-134/PREDICTED: photosystem I reaction center subunit III, chloroplastic [Glycine max]
Glyma.11 G181100	-	-	-	-	-	-	1.04059	0.001002	-1.8837	6.96E-06	826	ko00195//Photosynthesis	-	-	-	gi|351734544|ref|NP_001236146.1|/7.06598e-79/uncharacterized protein LOC100305746 [Glycine max]
Glyma.09 G087700	-	-	-	-	-	-	-	-	-1.8851	0.00079	842	ko00195//Photosynthesis;ko01100//Metabolic pathways	GO:0009521	GO:0046906//tetrapyrrole binding	GO:0044237//cellular metabolic process	gi|734421586|gb|KHN41353.1|/8.16956e-79/Photosystem I reaction center subunit psaK, chloroplastic [Glycine soja]
Glyma.17 G154900	-	-	-	-	-	-	-	-	-1.9216	0.00013	950	ko00195//Photosynthesis	GO:0009536//plastid	GO:0051536//iron-sulfur cluster binding;GO:0043169//cation binding	GO:0006091//generation of precursor metabolites and energy	gi|356565441|ref|XP_003550948.1|/9.65017e-122/PREDICTED: ferredoxin-2-like [Glycine max]
Glyma.06 G067400	-	-	-	-	-	-	-	-	-1.936	2.36E-06	1358	ko00195//Photosynthesis;ko00190//Oxidative phosphorylation;ko01100//Metabolic pathways	GO:0033177//proton-transporting two-sector ATPase complex, proton-transporting domain;GO:0009534//chloroplast thylakoid	GO:1901363;GO:0036094//small molecule binding;GO:0015077//monovalent inorganic cation transmembrane transporter activity;GO:0097159//organic cyclic compound binding	GO:0006818//hydrogen transport;GO:0006754//ATP biosynthetic process	gi|571459243|ref|XP_006581353.1|/5.72054e-113/PREDICTED: ATP synthase subunit b', chloroplastic-like [Glycine max]
Glyma.11 G061300	-	-	-	-	-	-	-	-	-1.9456	1.02E-08	1703	ko00195//Photosynthesis;ko01100//Metabolic pathways	GO:0031224//intrinsic component of membrane;GO:0009523//photosystem II;GO:0019867//outer membrane	GO:0046872//metal ion binding	GO:0042548//regulation of photosynthesis, light reaction	gi|358249094|ref|NP_001239736.1|/0/uncharacterized protein LOC100798485 [Glycine max]

20 × 20

Glyma.06 G020400	-	-	-	-	-	-	-	-	-1.9641	8.07E-07	1253	ko00195//Photosynthesis	GO:0031976;GO:0044436;GO:0044434	-	-	gi|351727401|ref|NP_001238695.1|/2.34942e-87/uncharacterized protein LOC100499708 [Glycine max]
Glyma.05 G112100	-	-	-	-	-	-	-	-	-1.9851	8.75E-07	839	ko00195//Photosynthesis	GO:0009536//plastid	GO:0051536//iron-sulfur cluster binding;GO:0043169//cation binding	GO:0006091//generation of precursor metabolites and energy	gi|955320478|ref|XP_003524714.3|;gi|947109880|gb|KRH58206.1|;gi|947109881|gb|KRH58207.1|/3.96943e-122;3.11608e-115;4.2284e-105/PREDICTED: ferredoxin-2-like isoform X2 [Glycine max];hypothetical protein GLYMA_05G112100 [Glycine max];hypothetical protein GLYMA_05G112100 [Glycine max]
Glyma.11 G245400	-	-	-	-	-	-	1.7987	1.35E-07	-2.0021	0.00605	1001	ko00195//Photosynthesis;ko01100//Metabolic pathways	-	-	-	gi|356540203|ref|XP_003538579.1|/3.7911e-68/PREDICTED: photosystem II core complex proteins psbY, chloroplastic-like [Glycine max]
Glyma.12 G092000	-	-	-	-	-	-	-	-	-2.0302	5.95E-06	831	ko00195//Photosynthesis	-	-	-	gi|571492781|ref|XP_006592347.1|/8.1433e-90/PREDICTED: photosystem I subunit O [Glycine max]
Glyma.02 G141700	-	-	-	-	-	-	-	-	-2.0322	2.37E-06	771	ko00195//Photosynthesis;ko01100//Metabolic pathways	GO:0044424	-	-	gi|947123122|gb|KRH71328.1|/2.16326e-65/hypothetical protein GLYMA_02G141700 [Glycine max]
Glyma.13 G129400	-	-	-	-	-	-	-	-	-2.0393	9.93E-06	757	ko00195//Photosynthesis;ko01100//Metabolic pathways	GO:0044434;GO:0009522//photosystem I	-	-	gi|351722581|ref|NP_001236993.1|/5.52099e-62/uncharacterized protein LOC100526993 [Glycine max]
Glyma.14 G031800	-	-	-	-	-	-	-	-	-2.0778	1.32E-07	1121	ko00195//Photosynthesis;ko01100//Metabolic pathways	GO:0009532//plastid stroma;GO:0009526//plastid envelope;GO:0009534//chloroplast thylakoid;GO:0009523//photosystem II;GO:0005576//extracellular region	GO:0008187//poly-pyrimidine tract binding;GO:0046872//metal ion binding	GO:0009617//response to bacterium;GO:0044237//cellular metabolic process	gi|214011454|gb|ACJ61477.1|/0/oxygen-evolving enhancer protein 2 [Glycine max]
Glyma.03 G114600	-	-	-	-	-	-	-	-	-2.0798	5.46E-06	1073	ko00195//Photosynthesis;ko01100//Metabolic pathways	GO:0009523//photosystem II	GO:0046872//metal ion binding	GO:0044237//cellular metabolic process	gi|351727615|ref|NP_001236143.1|/6.80492e-156/uncharacterized protein LOC100499794 [Glycine max]
Glyma.06 G321900	-1.0182	2.38E-07	-	-	-	-	-	-	-2.0799	1.53E-07	785	ko00195//Photosynthesis;ko01100//Metabolic pathways	GO:0009522//photosystem I;GO:0009526//plastid envelope;GO:0031976	GO:0046906//tetrapyrrole binding	GO:0009767//photosynthetic electron transport chain;GO:0031647//regulation of protein stability;GO:0042548//regulation of photosynthesis, light reaction;GO:0006740//NADPH regeneration	gi|351725243|ref|NP_001235038.1|/2.09257e-113/uncharacterized protein LOC100499718 [Glycine max]

20 × 20

Glyma.07G112000	-	-	-	-	-	-	-	-	-2.1165	5.57E-08	1062	ko00195//Photosynthesis;ko01100//Metabolic pathways	GO:0031976;GO:0009523//photosystem II;GO:0009532//plastid stroma	GO:0046872//metal ion binding	GO:0044237//cellular metabolic process	gi|214011474|gb|ACJ61487.1|;gi|947100285|gb|KRH48777.1|/3.30313e-153;7.43809e-146/oxygen-evolving enhancer protein 3 [Glycine max] ;hypothetical protein GLYMA_07G112000 [Glycine max]
Glyma.02G282500	-	-	-	-	-	-	-	-	-2.1721	3.91E-08	1217	ko00195//Photosynthesis;ko01100//Metabolic pathways	GO:0009532//plastid stroma;GO:0009526//plastid envelope;GO:0009534//chloroplast thylakoid;GO:0009523//photosystem II;GO:0005576//extracellular region	GO:0008187//poly-pyrimidine tract binding;GO:0046872//metal ion binding	GO:0009617//response to bacterium;GO:0044237//cellular metabolic process	gi|734416475|gb|KHN38354.1|/0/Oxygen-evolving enhancer protein 2, chloroplastic [Glycine soja]
Glyma.10G042000	-	-	-	-	-	-	-	-	-2.2129	1.99E-05	820	ko00195//Photosynthesis;ko01100//Metabolic pathways	GO:0044434;GO:0009522//photosystem I	-	-	gi|947083556|gb|KRH32277.1|/3.15973e-97/hypothetical protein GLYMA_10G042000 [Glycine max]
Glyma.12G199400	-	-	-	-	-	-	-	-	-2.239	1.58E-08	1088	ko00195//Photosynthesis;ko01100//Metabolic pathways	GO:0031224//intrinsic component of membrane;GO:0009526//plastid envelope;GO:0031976;GO:0034357	GO:0043169//cation binding;GO:0015078//hydrogen ion transmembrane transporter activity;GO:0051536//iron-sulfur cluster binding;GO:0009055//electron carrier activity;GO:0052880	GO:1990066;GO:0044710;GO:0009617//response to bacterium;GO:0044237//cellular metabolic process;GO:0015992//proton transport	gi|734321874|gb|KHN04299.1|/1.5483e-156/Cytochrome b6-f complex iron-sulfur subunit, chloroplastic [Glycine soja]
Glyma.13G357300	-	-	-	-	-	-	1.34091	5.40E-06	-2.3494	1.61E-07	1254	ko00195//Photosynthesis;ko01100//Metabolic pathways	GO:0031976;GO:0009570//chloroplast stroma;GO:0009522//photosystem I	-	GO:0044237//cellular metabolic process	gi|734403802|gb|KHN32624.1|/2.63003e-70/Photosystem I reaction center subunit VI, chloroplastic [Glycine soja]
Glyma.07G019700	-	-	-	-	-	-	-	-	-2.3656	0.00014	884	ko00195//Photosynthesis;ko01100//Metabolic pathways	GO:0031976;GO:0009570//chloroplast stroma;GO:0009522//photosystem I	-	GO:0044237//cellular metabolic process	gi|351726538|ref|NP_001236618.1|;gi|571463893|ref|XP_006582847.1|/1.42596e-73;1.51029e-78/uncharacterized protein LOC100499832 [Glycine max];PREDICTED: uncharacterized protein LOC100499832 isoform X1 [Glycine max]
Glyma.13G204800	-	-	-	-	-	-	-	-	-2.4727	5.26E-05	1800	ko00195//Photosynthesis;ko00190//Oxidative phosphorylation;ko01100//Metabolic pathways	GO:0033178//proton-transporting two-sector ATPase complex, catalytic domain	GO:0015078//hydrogen ion transmembrane transporter activity;GO:0019829//cation-transporting ATPase activity	GO:0006754//ATP biosynthetic process	gi|734415192|gb|KHN37604.1|/0/ATP synthase gamma chain, chloroplastic [Glycine soja]
Glyma.15G107900	-	-	-	-	-	-	-	-	-2.4995	4.16E-10	1892	ko00195//Photosynthesis;ko00190//Oxidative phosphorylation;ko01100//Metabolic pathways	GO:0033178//proton-transporting two-sector ATPase complex, catalytic domain	GO:0015078//hydrogen ion transmembrane transporter activity;GO:0019829//cation-transporting ATPase activity	GO:0006754//ATP biosynthetic process	gi|734404516|gb|KHN33027.1|/0/ATP synthase gamma chain, chloroplastic [Glycine soja]

Glyma.12 G169600	-	-	-	-	-	-	-	-	-2.5352	6.82E-07	889	ko00195//Photosynthesis	GO:0009536//plastid	GO:0051536//iron-sulfur cluster binding	GO:0022900//electron transport chain	gi|356543418|ref|XP_003540157.1|/6.45877e-93/PREDICTED: ferredoxin-A-like [Glycine max]
Glyma.04 G020300	-	-	-	-	-	-	-	-	-2.5573	1.76E-09	1273	ko00195//Photosynthesis	GO:0031976;GO:0044436;GO:0044434	GO:0046872//metal ion binding	-	gi|734406057|gb|KHN33826.1|/1.33719e-85/Plastocyanin, chloroplastic [Glycine soja]
Glyma.12 G169500	-	-	-	-	-	-	1.17486	0.000723	-3.199	3.16E-08	881	ko00195//Photosynthesis	GO:0009536//plastid	GO:0051536//iron-sulfur cluster binding	GO:0044710	gi|351723843|ref|NP_001237037.1|/2.17897e-93/uncharacterized protein LOC100500372 [Glycine max]
Glyma.02 G184000	1.45087	0.00018	2.18905	2.86E-07	1.89131	0.00124	-	-	-4.1015	4.19E-07	461	ko00195//Photosynthesis;ko00190//Oxidative phosphorylation;ko01100//Metabolic pathways	GO:0033178//proton-transporting two-sector ATPase complex, catalytic domain;GO:0009534//chloroplast thylakoid	GO:0032550;GO:0019829//cation-transporting ATPase activity;GO:0015078//hydrogen ion transmembrane transporter activity	GO:0015986//ATP synthesis coupled proton transport	gi|947123790|gb|KRH71996.1|/1.26336e-85/hypothetical protein GLYMA_02G184000, partial [Glycine max]
Glyma.08 G082900	-	-	-	-	-	-	2.15566	5.66E-07	-	-	1357	ko01100//Metabolic pathways;ko00196//Photosynthesis - antenna proteins	GO:0009521;GO:0031224//intrinsic component of membrane;GO:0009534//chloroplast thylakoid	GO:0046906//tetrapyrrole binding;GO:0043169//cation binding	GO:0006464//cellular protein modification process;GO:0006091//generation of precursor metabolites and energy	gi|359806638|ref|NP_001241277.1|/0/chlorophyll a-b binding protein 3, chloroplastic-like [Glycine max]
Glyma.16 G162600	-	-	-	-	-	-	2.70875	6.86E-05	-	-	511	ko01100//Metabolic pathways;ko00196//Photosynthesis - antenna proteins	GO:0009521;GO:0031224//intrinsic component of membrane;GO:0009534//chloroplast thylakoid	GO:0046906//tetrapyrrole binding;GO:0043169//cation binding	GO:0006464//cellular protein modification process;GO:0006091//generation of precursor metabolites and energy	gi|947059228|gb|KRH08634.1|/2.29509e-86/hypothetical protein GLYMA_16G162600, partial [Glycine max]
Glyma.16 G165800	-1.6062	7.18E-10	-	-	-	-	2.70675	4.48E-12	-	-	1884	ko01100//Metabolic pathways;ko00196//Photosynthesis - antenna proteins	GO:0009521;GO:0031976	GO:0043169//cation binding	GO:0006091//generation of precursor metabolites and energy	gi|358248490|ref|NP_001240146.1|/0/chlorophyll a-b binding protein 21, chloroplastic-like [Glycine max]
Glyma.05 G128000	-	-	-	-	-	-	2.23169	2.28E-06	-1.0987	0.00637	1131	ko01100//Metabolic pathways;ko00196//Photosynthesis - antenna proteins	GO:0009521;GO:0031224//intrinsic component of membrane;GO:0009534//chloroplast thylakoid	GO:0046906//tetrapyrrole binding;GO:0043169//cation binding	GO:0006464//cellular protein modification process;GO:0006091//generation of precursor metabolites and energy	gi|255646685|gb|ACU23816.1|/0/unknown [Glycine max]
Glyma.15 G052400	-	-	-	-	-	-	-	-	-1.1359	0.00041	1645	ko01100//Metabolic pathways;ko00196//Photosynthesis - antenna proteins	GO:0009526//plastid envelope;GO:0009570//chloroplast stroma;GO:0009534//chloroplast thylakoid	-	GO:1990066;GO:0006091//generation of precursor metabolites and energy	gi|734405712|gb|KHN33514.1|/1.20949e-173/Chlorophyll a-b binding protein CP24 10A, chloroplastic [Glycine soja]
Glyma.16 G205200	-	-	-	-	-	-	-	-	-1.3178	0.001	1338	ko01100//Metabolic pathways;ko00196//Photosynthesis - antenna proteins	GO:0009503;GO:0009523//photosystem II;GO:0009570//chloroplast stroma	GO:0043169//cation binding	GO:1990066;GO:0009639//response to red or far red light;GO:0006091//generation of precursor metabolites and	gi|734384942|gb|KHN24427.1|/8.97488e-165/Chlorophyll a-b binding protein CP26, chloroplastic [Glycine soja]

20 × 20

															energy	
Glyma.12 G219300	-	-	-	-	-	-	-	-	-1.3908	0.00061	1237	ko01100//Metabolic pathways;ko00196//Photosynthesis - antenna proteins	GO:0009521;GO:0009536//plastid	GO:0043169//cation binding	GO:0006091//generation of precursor metabolites and energy	gi|359807444|ref|NP_001240880.1|/0/uncharacterized protein LOC100779387 [Glycine max]
Glyma.08 G180000	-	-	-	-	-	-	-	-	-1.4136	5.47E-05	1328	ko01100//Metabolic pathways;ko00196//Photosynthesis - antenna proteins	GO:0009526//plastid envelope;GO:0009570//chloroplast stroma;GO:0009534//chloroplast thylakoid	-	GO:1990066;GO:0006091//generation of precursor metabolites and energy	gi|734390448|gb|KHN26736.1|/4.09065e-174/Chlorophyll a-b binding protein CP24 10A, chloroplastic [Glycine soja]
Glyma.03 G262300	-	-	-	-	-	-	-	-	-1.4695	9.53E-05	1398	ko00196//Photosynthesis - antenna proteins	GO:0009521;GO:0009534//chloroplast thylakoid	GO:0043169//cation binding	GO:0006091//generation of precursor metabolites and energy	gi|947120744|gb|KRH68993.1|;gi|358249066|ref|NP_001239987.1|/0;0/hypothetical protein GLYMA_03G262300 [Glycine max];uncharacterized protein LOC100794944 [Glycine max]
Glyma.05 G119000	-	-	-	-	-	-	-	-	-1.4759	0.00909	732	ko01100//Metabolic pathways;ko00196//Photosynthesis - antenna proteins	GO:0009536//plastid	-	GO:0006091//generation of precursor metabolites and energy	gi|947109970|gb|KRH58296.1|/2.40783e-163/hypothetical protein GLYMA_05G119000 [Glycine max]
Glyma.06 G194900	-	-	-	-	-	-	-	-	-1.4808	3.12E-05	1114	ko00196//Photosynthesis - antenna proteins	GO:0009521;GO:0009526//plastid envelope;GO:0031976;GO:0009570//chloroplast stroma	GO:0043169//cation binding	GO:0009628//response to abiotic stimulus;GO:0006091//generation of precursor metabolites and energy	gi|734389434|gb|KHN26254.1|/0/Chlorophyll a-b binding protein P4, chloroplastic [Glycine soja]
Glyma.14 G008000	-	-	-	-	-	-	-	-	-1.5094	4.07E-05	1200	ko01100//Metabolic pathways;ko00196//Photosynthesis - antenna proteins	GO:0009521;GO:0031224//intrinsic component of membrane;GO:0009526//plastid envelope;GO:0044437;GO:0009534//chloroplast thylakoid;GO:0009570//chloroplast stroma	GO:0043169//cation binding;GO:0046906//tetrapyrrole binding	GO:0006091//generation of precursor metabolites and energy;GO:0006464//cellular protein modification process;GO:0009639//response to red or far red light	gi|359806053|ref|NP_001241179.1|/0/uncharacterized protein LOC100815789 [Glycine max]
Glyma.14 G003400	-	-	-	-	-	-	-	-	-1.575	5.65E-06	1290	ko00196//Photosynthesis - antenna proteins	GO:0009521;GO:0009507//chloroplast	GO:0043169//cation binding	GO:0006091//generation of precursor metabolites and energy	gi|734433643|gb|KHN46884.1|/3.8163e-165/Chlorophyll a-b binding protein 3, chloroplastic [Glycine soja]
Glyma.13 G282000	-	-	-	-	-	-	-	-	-1.8672	2.16E-06	1245	ko01100//Metabolic pathways;ko00196//Photosynthesis - antenna proteins	GO:0009521;GO:0009536//plastid	GO:0043169//cation binding	GO:0006091//generation of precursor metabolites and energy	gi|359806176|ref|NP_001241200.1|/0/uncharacterized protein LOC100790960 [Glycine max]
Glyma.10 G177200	-	-	-	-	-	-	-	-	-1.8739	1.03E-06	1123	ko01100//Metabolic pathways;ko00196//Photosynthesis - antenna proteins	GO:0009503;GO:0009523//photosystem II;GO:0009570//chloroplast stroma	GO:0043169//cation binding	GO:1990066;GO:0009639//response to red or far red light;GO:0006091//generation of precursor metabolites and	gi|356535308|ref|XP_003536189.1|/6.78912e-163/PREDICTED: chlorophyll a-b binding protein CP26, chloroplastic [Glycine max]

															energy	
Glyma.07 G047600	-	-	-	-	-	-	-	-	-1.8987	1.98E-06	1400	ko00196//Photosynthesis - antenna proteins	GO:0009521;GO:0009534//chloroplast thylakoid	GO:0043169//cation binding	GO:0006091//generation of precursor metabolites and energy	gi|734313594|gb|KHN01491.1|;gi|947099250|gb|KRH47742.1|/0;0/Chlorophyll a-b binding protein 7, chloroplastic [Glycine soja] ;hypothetical protein GLYMA_07G047600 [Glycine max]
Glyma.20 G212900	-	-	-	-	-	-	-	-	-2.0117	3.38E-05	1076	ko01100//Metabolic pathways;ko00196//Photosynthesis - antenna proteins	GO:0009503;GO:0009523//photosystem II;GO:0009570//chloroplast stroma	GO:0043169//cation binding	GO:1990066;GO:0009639//response to red or far red light;GO:0006091//generation of precursor metabolites and energy	gi|734324257|gb|KHN05032.1|/7.10859e-173/Chlorophyll a-b binding protein CP26, chloroplastic [Glycine soja]
Glyma.02 G309500	-	-	-	-	-	-	-	-	-2.0384	3.99E-08	1945	ko00196//Photosynthesis - antenna proteins	GO:0009521;GO:0009507//chloroplast	GO:0043169//cation binding	GO:0006091//generation of precursor metabolites and energy	gi|734396929|gb|KHN29869.1|/5.17725e-165/Chlorophyll a-b binding protein 3, chloroplastic [Glycine soja]
Glyma.02 G064700	-	-	-	-	-	-	-	-	-2.0793	6.02E-07	2033	ko00196//Photosynthesis - antenna proteins	GO:0009521;GO:0009536//plastid	GO:0043169//cation binding	GO:0006091//generation of precursor metabolites and energy	gi|947121839|gb|KRH70045.1|;gi|947121840|gb|KRH70046.1|;gi|734312433|gb|KHN00611.1|/3.16771e-162;3.01752e-138;7.33888e-176/hypothetical protein GLYMA_02G064700 [Glycine max];hypothetical protein GLYMA_02G064700 [Glycine max];Chlorophyll a-b binding protein 6A, chloroplastic [Glycine soja]
Glyma.16 G145800	-	-	-	-	-	-	-	-	-2.0802	2.09E-06	1161	ko00196//Photosynthesis - antenna proteins	GO:0009521;GO:0009536//plastid	GO:0043169//cation binding	GO:0006091//generation of precursor metabolites and energy	gi|955376843|ref|XP_006599410.2|/2.30353e-164/PREDICTED: chlorophyll a-b binding protein 6A, chloroplastic [Glycine max]
Glyma.09 G154700	-	-	-	-	-	-	1.16335	0.000718	-2.0875	2.44E-06	1191	ko01100//Metabolic pathways;ko00196//Photosynthesis - antenna proteins	GO:0009503;GO:0009523//photosystem II;GO:0009570//chloroplast stroma	GO:0043169//cation binding	GO:1990066;GO:0009639//response to red or far red light;GO:0006091//generation of precursor metabolites and energy	gi|734392935|gb|KHN27863.1|/2.73903e-164/Chlorophyll a-b binding protein CP26, chloroplastic [Glycine soja]
Glyma.18 G028400	-	-	-	-	-	-	-	-	-2.1646	3.76E-07	1218	ko01100//Metabolic pathways;ko00196//Photosynthesis - antenna proteins	GO:0009536//plastid	-	GO:0009639//response to red or far red light;GO:0006091//generation of precursor metabolites and energy	gi|356568441|ref|XP_003552419.1|/2.48927e-167/PREDICTED: chlorophyll a-b binding protein CP29.3, chloroplastic-like [Glycine max]
Glyma.08 G074000	-	-	-	-	-	-	-	-	-2.3629	8.30E-10	1090	ko01100//Metabolic pathways;ko00196//Photosynthesis - antenna proteins	GO:0009536//plastid	-	GO:0006091//generation of precursor metabolites and energy	gi|734417899|gb|KHN39211.1|/0/Chlorophyll a-b binding protein CP24 10A, chloroplastic [Glycine soja]

20 × 20

Glyma.02G305400	-	-	-	-	-	-	-	-	-2.7984	3.57E-12	1322	ko01100//Metabolic pathways;ko00196//Photosynthesis - antenna proteins	GO:0009521;GO:0009526//plastid envelope;GO:0009570//chloroplast stroma;GO:0009534//chloroplast thylakoid;GO:0044437;GO:0031224//intrinsic component of membrane	GO:0043169//cation binding;GO:0046906//tetrapyrrole binding	GO:0009639//response to red or far red light;GO:0006091//generation of precursor metabolites and energy;GO:0006464//cellular protein modification process	gi|947125798|gb|KRH74004.1|;gi|358248712|ref|NP_001240183.1|/1.06595e-163;0/hypothetical protein GLYMA_02G305400 [Glycine max];chlorophyll a-b binding protein 151, chloroplastic-like [Glycine max]

Supplementary Table S5: DEGs involved in starch and sucrose metabolism

GeneID	log2Ratio(2mlpa-1/2MWT-1)	P-value	log2Ratio(2mlpa-2/2MWT-2)	P-value	log2Ratio(2mlpa-3/2MWT-3)	P-value	log2Ratio(2mlpa-4/2MWT-4)	P-value	log2Ratio(2mlpa-5/2MWT-5)	P-value	Length	Pathway	GO Component	GO Function	GO Process	Blast nr
Glyma.01G007300	1.24442	0.00119	-	-	-	-	-	-	-	-	2189	ko00051//Fructose and mannose metabolism;ko00500//Starch and sucrose metabolism;ko00010//Glycolysis / Gluconeogenesis;ko01100//Metabolic pathways;ko01110//Biosynthesis of secondary metabolites;ko00520//Amino sugar and nucleotide sugar metabolism;ko00052//Galactose metabolism	GO:0044437;GO:0031306//intrinsic component of mitochondrial outer membrane	GO:0046914//transition metal ion binding;GO:0032550;GO:0004396//hexokinase activity	GO:0006833//water transport;GO:0010118//stomatal movement;GO:0016310//phosphorylation;GO:0006091//generation of precursor metabolites and energy;GO:0009757//hexose mediated signaling;GO:0019318//hexose metabolic process;GO:0008219//cell death	gi|356495847|ref|XP_003516783.1|/0/PREDICTED: hexokinase-1-like [Glycine max]
Glyma.01G209500	1.13084	3.17E-05	-	-	-	-	-	-	-	-	1443	ko00040//Pentose and glucuronate interconversions;ko00500//Starch and sucrose metabolism;ko01100//Metabolic pathways	GO:0030312//external encapsulating structure	GO:0052689//carboxylic ester hydrolase activity	GO:0071555//cell wall organization	gi|356497141|ref|XP_003517421.1|/0/PREDICTED: probable pectinesterase 68 [Glycine max]
Glyma.02G186800	-1.4574	4.16E-07	-	-	-1.4823	0.00069	-	-	-	-	1926	ko00500//Starch and sucrose metabolism;ko01100//Metabolic pathways;ko01110//Biosynthesis of secondary metabolites;ko00460//Cyanoamino acid metabolism;ko00940//Phenylpropanoid biosynthesis	GO:0005911//cell-cell junction;GO:0005618//cell wall	GO:0016798//hydrolase activity, acting on glycosyl bonds	GO:0044238//primary metabolic process	gi|734320135|gb|KHN03708.1|/0/ Lysosomal beta glucosidase [Glycine soja]
Glyma.02G272100	-1.2165	3.18E-06	-	-	-	-	-	-	-	-	2421	ko00500//Starch and sucrose metabolism;ko01100//Metabolic pathways;ko01110//Biosynthesis of secondary metabolites;ko00460//Cyanoamino acid metabolism;ko00940//Phenylpropanoid biosynthesis	GO:0005911//cell-cell junction;GO:0005618//cell wall	GO:0016798//hydrolase activity, acting on glycosyl bonds	GO:0044238//primary metabolic process	gi|955308958|ref|XP_014625681.1|;gi|947125214|gb|KRH73420.1|;gi|955308961|ref|XP_014625684.1|/0;0;0/PREDICTED: beta-glucosidase BoGH3B-like isoform X1 [Glycine max];hypothetical protein GLYMA_02G272100 [Glycine max];PREDICTED: beta-glucosidase BoGH3B-like isoform X3 [Glycine max]
Glyma.03G214600	-3.6427	2.08E-07	-	-	-	-	-	-	-	-	1194	ko00040//Pentose and glucuronate interconversions;ko00500//Starch and sucrose metabolism;ko01100//Metabolic pathways	-	GO:0004857//enzyme inhibitor activity;GO:0052689//carboxylic ester hydrolase activity	GO:0003006//developmental process involved in reproduction;GO:0044092//negative regulation of molecular function	gi|356505586|ref|XP_003521571.1|/8.44156e-131/PREDICTED: 21 kDa protein-like [Glycine max]
Glyma.04G103600	-	-	-	-	-	-	-1.7978	6.89E-06	-	-	1864	ko00500//Starch and sucrose metabolism	-	GO:0019203//carbohydrate phosphatase activity	GO:0006796//phosphate-containing compound metabolic process;GO:0005991//trehalose metabolic process	gi|571450061|ref|XP_003523824.2|/0/PREDICTED: probable trehalose-phosphate phosphatase J [Glycine max]
Glyma.05G216400	-	-	-	-	-	-	1.09973	0.00042	-	-	2663	ko00500//Starch and sucrose metabolism;ko01100//Metabolic pathways	-	GO:0005488;GO:0004553//hydrolase activity, hydrolyzing O-glycosyl compounds	GO:0044238//primary metabolic process	gi|734310523|gb|KHM99903.1|/0/ Endoglucanase 6 [Glycine soja]
Glyma.05G236400	-	-	-	-	1.20227	0.00027	2.30482	2.33E-09	-	-	2164	ko00500//Starch and sucrose metabolism;ko01100//Metabolic pathways	GO:0009536//plastid	GO:0016798//hydrolase activity, acting on glycosyl bonds	GO:0009825//multidimensional cell growth;GO:0044238//primary metabolic process	gi|356513078|ref|XP_003525241.1|/0/PREDICTED: endoglucanase 8-like [Glycine max]

Glyma.06G301500	-1.4048	8.58E-06	-	-	-	-	-	-	-	-	1891	ko00500//Starch and sucrose metabolism;ko01100//Metabolic pathways	-	GO:0016160//amylase activity	GO:0005976//polysaccharide metabolic process	gi|902938|dbj|BAA09462.1|/0/beta-amylase [Glycine max]
Glyma.06G318500	-1.2422	0.00011	-	-	-	-	-	-	-	-	2399	ko00500//Starch and sucrose metabolism;ko01100//Metabolic pathways;ko00052//Galactose metabolism	-	GO:0004558//alpha-1,4-glucosidase activity	GO:0044238//primary metabolic process	gi|356515372|ref|XP_003526374.1|/0/PREDICTED: beta-fructofuranosidase, soluble isoenzyme I-like [Glycine max]
Glyma.07G046000	-1.5628	1.03E-09	-	-	-	-	-	-	-	-	3558	ko00040//Pentose and glucuronate interconversions;ko00500//Starch and sucrose metabolism;ko01100//Metabolic pathways	GO:0005618//cell wall;GO:0043231//intracellular membrane-bounded organelle;GO:0044444;GO:0005576//extracellular region;GO:0016020//membrane	GO:0030234//enzyme regulator activity;GO:0052689//carboxylic ester hydrolase activity	GO:0044092//negative regulation of molecular function;GO:0071555//cell wall organization;GO:0000272//polysaccharide catabolic process	gi|947099228|gb|KRH47720.1|/0/hypothetical protein GLYMA_07G046000 [Glycine max]
Glyma.08G074700	-	-	-	-	1.33249	4.12E-06	-	-	-	-	3060	ko00500//Starch and sucrose metabolism;ko01100//Metabolic pathways;ko00520//Amino sugar and nucleotide sugar metabolism	GO:0031012//extracellular matrix	GO:0004553//hydrolase activity, hydrolyzing O-glycosyl compounds	GO:0000272//polysaccharide catabolic process	gi|947093612|gb|KRH42197.1|;gi|356524862|ref|XP_003531047.1|/0;0/hypothetical protein GLYMA_08G074700 [Glycine max];PREDICTED: beta-xylosidase/alpha-L-arabinofuranosidase 2 [Glycine max]
Glyma.08G120800	-2.1112	1.92E-05	-	-	-	-	-	-	-	-	3054	ko00500//Starch and sucrose metabolism	-	GO:0019203//carbohydrate phosphatase activity;GO:0035251//UDP-glucosyltransferase activity	GO:0006796//phosphate-containing compound metabolic process;GO:0005991//trehalose metabolic process	gi|955335317|ref|XP_014634392.1|/0/PREDICTED: alpha,alpha-trehalose-phosphate synthase [UDP-forming] 1-like [Glycine max]
Glyma.09G122000	-	-	-	-	-	-	1.25288	0.00104	-	-	838	ko00040//Pentose and glucuronate interconversions;ko00500//Starch and sucrose metabolism;ko01100//Metabolic pathways	-	-	-	gi|356530768|ref|XP_003533952.1|/4.81613e-96/PREDICTED: 21 kDa protein-like [Glycine max]
Glyma.09G208200	-1.9498	0.00026	-2.3099	0.00061	-	-	-	-	-	-	4334	ko00500//Starch and sucrose metabolism;ko01100//Metabolic pathways;ko00520//Amino sugar and nucleotide sugar metabolism	GO:0031224//intrinsic component of membrane	GO:0016759//cellulose synthase activity	GO:0030243//cellulose metabolic process;GO:0009856//pollination	gi|734417458|gb|KHN38958.1|/0/Cellulose synthase-like protein D3 [Glycine soja]
Glyma.10G074800	2.30522	0.00016	-	-	-	-	-	-	-	-	1959	ko00500//Starch and sucrose metabolism;ko01100//Metabolic pathways;ko00052//Galactose metabolism	-	GO:0016798//hydrolase activity, acting on glycosyl bonds	GO:0005985//sucrose metabolic process;GO:0046903//secretion;GO:0006073//cellular glucan metabolic process	gi|356534392|ref|XP_003535739.1|/0/PREDICTED: beta-fructofuranosidase, insoluble isoenzyme 1-like isoform X1 [Glycine max]
Glyma.10G230900	-	-	-	-	-	-	3.42196	0.0003	-	-	1577	ko00040//Pentose and glucuronate interconversions;ko00500//Starch and sucrose metabolism;ko01100//Metabolic pathways	-	GO:0004553//hydrolase activity, hydrolyzing O-glycosyl compounds	GO:0044238//primary metabolic process	gi|356534103|ref|XP_003535597.1|/0/PREDICTED: probable polygalacturonase [Glycine max]
Glyma.11G039400	-	-	-	-	-2.8434	2.02E-05	-	-	-	-	2028	ko00500//Starch and sucrose metabolism;ko01100//Metabolic pathways	GO:0009532//plastid stroma	GO:0016160//amylase activity	GO:0006950//response to stress;GO:0005976//polysaccharide metabolic process;GO:0000023//maltose metabolic process	gi|734408776|gb|KHN34883.1|/0/Beta-amylase 3, chloroplastic [Glycine soja]
Glyma.11G101300	-1.2561	0.00348	-	-	-	-	-	-	-	-	2321	ko00500//Starch and sucrose metabolism;ko01100//Metabolic pathways	-	GO:0016798//hydrolase activity, acting on glycosyl bonds;GO:0005488;GO:0004553//hydrolase activity, hydrolyzing O-glycosyl compounds	GO:0044238//primary metabolic process	gi|571487997|ref|XP_006590804.1|;gi|947080369|gb|KRH29158.1|;gi|947080368|gb|KRH29157.1|/0;0;0/PREDICTED: endoglucanase 5-like [Glycine max];hypothetical protein GLYMA_11G101300 [Glycine max];hypothetical protein GLYMA_11G101300 [Glycine max]
Glyma.12G004700	-	-	-4.0972	3.19E-07	-	-	-	-	-	-	1295	ko00500//Starch and sucrose metabolism;ko01100//Metabolic pathways	-	GO:0016798//hydrolase activity, acting on glycosyl bonds	GO:0044238//primary metabolic process	gi|947074959|gb|KRH23799.1|/0/hypothetical protein GLYMA_12G004700 [Glycine max]

Glyma.14G006100	-1.0249	0.00479	-	-	-	-	-	-	-	-	2078	ko00040//Pentose and glucuronate interconversions;ko00500//Starch and sucrose metabolism;ko01100//Metabolic pathways	-	GO:0004553//hydrolase activity, hydrolyzing O-glycosyl compounds	GO:0044238//primary metabolic process	gi|955362405|ref|XP_014622079.1|;gi|947064948|gb|KRH14091.1|;gi|356551512|ref|XP_003544118.1|;gi|947064949|gb|KRH14092.1|/0;0;0;0/PREDICTED: polygalacturonase-like isoform X2 [Glycine max];hypothetical protein GLYMA_14G006100 [Glycine max];PREDICTED: polygalacturonase-like isoform X1 [Glycine max];hypothetical protein GLYMA_14G006100 [Glycine max]
Glyma.14G027400	-1.192	0.00121	-	-	-	-	-	-	-	-	2094	ko00500//Starch and sucrose metabolism;ko01110//Biosynthesis of secondary metabolites;ko00520//Amino sugar and nucleotide sugar metabolism	-	GO:0016740//transferase activity	-	gi|356553593|ref|XP_003545139.1|/0/PREDICTED: probable galacturonosyltransferase 15 [Glycine max]
Glyma.14G029100	-1.2122	3.64E-07	-	-	-1.3526	4.48E-05	-	-	-	-	3743	ko00500//Starch and sucrose metabolism;ko01100//Metabolic pathways	-	GO:0035251//UDP-glucosyltransferase activity	GO:0005984//disaccharide metabolic process	gi|356553609|ref|XP_003545147.1|/0/PREDICTED: probable sucrose-phosphate synthase 3 [Glycine max]
Glyma.15G146800	1.08002	3.06E-05	-	-	-	-	-	-	-	-	2096	ko00040//Pentose and glucuronate interconversions;ko00500//Starch and sucrose metabolism;ko01100//Metabolic pathways	GO:0005618//cell wall;GO:0043231//intracellular membrane-bounded organelle	GO:0004553//hydrolase activity, hydrolyzing O-glycosyl compounds	GO:0044238//primary metabolic process	gi|571518634|ref|XP_006597719.1|/0/PREDICTED: probable polygalacturonase isoform X1 [Glycine max]
Glyma.15G184300	-1.5645	4.02E-07	-	-	-	-	-	-	-	-	1956	ko00040//Pentose and glucuronate interconversions;ko00500//Starch and sucrose metabolism;ko01100//Metabolic pathways	-	GO:0016788//hydrolase activity, acting on ester bonds	GO:0008152//metabolic process	gi|356558481|ref|XP_003547535.1|/0/PREDICTED: pectinesterase/pectinesterase inhibitor PPE8B-like [Glycine max]
Glyma.15G223500	-1.5335	2.50E-05	-	-	-	-	-	-	-	-	2432	ko00040//Pentose and glucuronate interconversions;ko00500//Starch and sucrose metabolism;ko01100//Metabolic pathways	GO:0030312//external encapsulating structure	GO:0030234//enzyme regulator activity;GO:0052689//carboxylic ester hydrolase activity	GO:0071555//cell wall organization;GO:0000272//polysaccharide catabolic process;GO:0044092//negative regulation of molecular function	gi|571520749|ref|XP_006598050.1|/0/PREDICTED: probable pectinesterase/pectinesterase inhibitor 47 [Glycine max]
Glyma.16G039300	-	-	-	-	-	-	1.58576	0.00107	-	-	1845	ko00500//Starch and sucrose metabolism;ko01100//Metabolic pathways;ko01110//Biosynthesis of secondary metabolites;ko00460//Cyanoamino acid metabolism;ko00940//Phenylpropanoid biosynthesis	-	GO:0016798//hydrolase activity, acting on glycosyl bonds	GO:0044238//primary metabolic process	gi|947057276|gb|KRH06682.1|/0/hypothetical protein GLYMA_16G039300 [Glycine max]
Glyma.16G118000	-6.9155	1.85E-28	-6.2802	2.06E-18	-5.5221	2.62E-12	-4.894	1.01E-08	-	-	412	ko00040//Pentose and glucuronate interconversions;ko00500//Starch and sucrose metabolism;ko01100//Metabolic pathways	-	GO:0004553//hydrolase activity, hydrolyzing O-glycosyl compounds	GO:0044238//primary metabolic process	gi|947058503|gb|KRH07909.1|/1.98927e-57/hypothetical protein GLYMA_16G118000 [Glycine max]
Glyma.17G138500	-	-	-	-	-	-	2.64156	0.00024	-	-	2505	ko00500//Starch and sucrose metabolism;ko01100//Metabolic pathways;ko00052//Galactose metabolism	GO:0005618//cell wall;GO:0043231//intracellular membrane-bounded organelle	GO:0004558//alpha-1,4-glucosidase activity	GO:0005984//disaccharide metabolic process;GO:0051707//response to other organism;GO:0048364//root development;GO:0009739//response to gibberellin	gi|947054630|gb|KRH04083.1|;gi|356563300|ref|XP_003549902.1|/0;0/hypothetical protein GLYMA_17G138500 [Glycine max];PREDICTED: acid beta-fructofuranosidase [Glycine max]
Glyma.17G188200	-	-	-	-	-2.3544	0.00018	-	-	-	-	2133	ko00500//Starch and sucrose metabolism;ko01100//Metabolic pathways;ko01110//Biosynthesis of secondary metabolites;ko00460//Cyanoamino acid metabolism;ko00940//Phenylpropanoid biosynthesis	-	GO:0016798//hydrolase activity, acting on glycosyl bonds	GO:0044238//primary metabolic process	gi|356565758|ref|XP_003551104.1|/0/PREDICTED: beta-glucosidase BoGH3B-like [Glycine max]
Glyma.18G06540	-	-	-	-	3.06312	2.84E-08	3.02159	1.77E-06	-	-	1968	ko00500//Starch and sucrose metabolism;ko01100//Metabolic pathways;ko01110//Biosynthesis of	GO:0044444	GO:0016787//hydrolase activity	-	gi|947048791|gb|KRG98319.1|/0/hypothetical protein GLYMA_18G065400 [Glycine

20 × 20

0												secondary metabolites;ko00460//Cyanoamino acid metabolism;ko00940//Phenylpropanoid biosynthesis				max]
Glyma.19G002500	-4.0922	2.07E-10	-	-	-	-	-	-	-	-	294	ko00500//Starch and sucrose metabolism;ko01100//Metabolic pathways	-	-	-	gi|947043560|gb|KRG93189.1|/4.91238e-68/hypothetical protein GLYMA_19G002500 [Glycine max]
Glyma.19G016100	-	-	-	-	-3.9277	0.00026	-	-	-	-	1822	ko00500//Starch and sucrose metabolism;ko01110//Biosynthesis of secondary metabolites;ko00520//Amino sugar and nucleotide sugar metabolism	-	GO:0016740//transferase activity	GO:0006950//response to stress	gi|356572000|ref|XP_003554158.1|/0/PREDICTED: probable galacturonosyltransferase-like 10 [Glycine max]
Glyma.19G078400	-	-	-	-	-	-	-5.1015	2.31E-07	-	-	1712	ko00040//Pentose and glucuronate interconversions;ko00500//Starch and sucrose metabolism;ko01100//Metabolic pathways	GO:0030312//external encapsulating structure	GO:0030234//enzyme regulator activity;GO:0052689//carboxylic ester hydrolase activity	GO:0071555//cell wall organization;GO:0000272//polysaccharide catabolic process;GO:0044092//negative regulation of molecular function	gi|955387229|ref|XP_003553884.2|/0/PREDICTED: probable pectinesterase/pectinesterase inhibitor 36 isoform X1 [Glycine max]
Glyma.19G223100	1.54648	0.00117	-	-	-	-	-	-	-	-	2289	ko00500//Starch and sucrose metabolism;ko01100//Metabolic pathways;ko00520//Amino sugar and nucleotide sugar metabolism	GO:0009536//plastid	GO:0070566//adenylyltransferase activity	GO:0005982//starch metabolic process;GO:0005977//glycogen metabolic process	gi|571559815|ref|XP_003553688.2|/0/PREDICTED: glucose-1-phosphate adenylyltransferase large subunit 1-like [Glycine max]
Glyma.19G231300	-1.0956	0.00487	-	-	-	-	-	-	-	-	2028	ko00040//Pentose and glucuronate interconversions;ko00500//Starch and sucrose metabolism;ko01100//Metabolic pathways	GO:0030312//external encapsulating structure	GO:0030234//enzyme regulator activity;GO:0052689//carboxylic ester hydrolase activity	GO:0071555//cell wall organization;GO:0000272//polysaccharide catabolic process;GO:0044092//negative regulation of molecular function	gi|356572956|ref|XP_003554631.1|/0/PREDICTED: putative pectinesterase/pectinesterase inhibitor 22 [Glycine max]
Glyma.20G029100	-1.7867	2.00E-05	-	-	-	-	-	-	-	-	2119	ko00500//Starch and sucrose metabolism;ko01100//Metabolic pathways;ko00052//Galactose metabolism	-	GO:0016798//hydrolase activity, acting on glycosyl bonds	GO:0005985//sucrose metabolic process;GO:0046903//secretion;GO:0006073//cellular glucan metabolic process	gi|571563934|ref|XP_006605559.1|;gi|356577241|ref|XP_003556736.1|/0;0/PREDICTED: beta-fructofuranosidase, insoluble isoenzyme 1-like isoform X2 [Glycine max];PREDICTED: beta-fructofuranosidase, insoluble isoenzyme 1-like isoform X1 [Glycine max]
Glyma.12G130200	-	-	-	-	-	-	-	-	8.47386	2.32E-05	1900	ko00500//Starch and sucrose metabolism;ko01100//Metabolic pathways;ko01110//Biosynthesis of secondary metabolites;ko00460//Cyanoamino acid metabolism;ko00940//Phenylpropanoid biosynthesis	-	GO:0016798//hydrolase activity, acting on glycosyl bonds	GO:0044238//primary metabolic process	gi|955354334|ref|XP_003540006.2|/0/PREDICTED: cyanogenic beta-glucosidase-like [Glycine max]
Glyma.12G053800	-	-	-	-	-	-	-	-	8.43265	2.44E-05	2190	ko00500//Starch and sucrose metabolism;ko01100//Metabolic pathways;ko01110//Biosynthesis of secondary metabolites;ko00460//Cyanoamino acid metabolism;ko00940//Phenylpropanoid biosynthesis	GO:0043231//intracellular membrane-bounded organelle	GO:0004553//hydrolase activity, hydrolyzing O-glycosyl compounds;GO:0016798//hydrolase activity, acting on glycosyl bonds	GO:0044238//primary metabolic process	gi|947075816|gb|KRH24656.1|;gi|734387515|gb|KHN25278.1|/0;0/hypothetical protein GLYMA_12G053800 [Glycine max];Non-cyanogenic beta-glucosidase [Glycine soja]
Glyma.11G129500	-	-	-	-	-	-	-	-	8.2002	3.81E-05	1893	ko00500//Starch and sucrose metabolism;ko01100//Metabolic pathways;ko01110//Biosynthesis of secondary metabolites;ko00460//Cyanoamino acid metabolism;ko00940//Phenylpropanoid biosynthesis	-	GO:0016798//hydrolase activity, acting on glycosyl bonds	GO:0044238//primary metabolic process	gi|734406299|gb|KHN33931.1|/0/Beta-glucosidase 24 [Glycine soja]
Glyma.12G054000	-	-	-	-	-	-	-	-	7.98202	5.82E-05	2046	ko00500//Starch and sucrose metabolism;ko01100//Metabolic pathways;ko01110//Biosynthesis of secondary metabolites;ko00460//Cyanoamino acid metabolism;ko00940//Phenylpropanoid biosynthesis	-	GO:0016798//hydrolase activity, acting on glycosyl bonds	GO:0044238//primary metabolic process	gi|734387513|gb|KHN25276.1|/0/Beta-glucosidase 24 [Glycine soja]

20 × 20

Glyma.01G240300	-	-	-	-	-	-	-	-	7.95868	6.55E-08	1921	ko00040//Pentose and glucuronate interconversions;ko00500//Starch and sucrose metabolism;ko01100//Metabolic pathways	GO:0030312//external encapsulating structure;GO:0044437	GO:0030234//enzyme regulator activity;GO:0052689//carboxylic ester hydrolase activity	GO:0071555//cell wall organization;GO:0000272//polysaccharide catabolic process;GO:0044092//negative regulation of molecular function	gi|571436840|ref|XP_006573891.1|/0/PREDICTED: pectinesterase-like [Glycine max]
Glyma.11G129700	-	-	-	-	-	-	-	-	7.37099	0.00012	1852	ko00500//Starch and sucrose metabolism;ko01100//Metabolic pathways;ko01110//Biosynthesis of secondary metabolites;ko00460//Cyanoamino acid metabolism;ko00940//Phenylpropanoid biosynthesis	-	GO:0016798//hydrolase activity, acting on glycosyl bonds	GO:0044238//primary metabolic process	gi|734406297|gb|KHN33929.1|/0/Beta-glucosidase 24 [Glycine soja]
Glyma.01G137700	-	-	-	-	-	-	-	-	7.21513	0.00016	1973	ko00040//Pentose and glucuronate interconversions;ko00500//Starch and sucrose metabolism;ko01100//Metabolic pathways	GO:0030312//external encapsulating structure	GO:0030234//enzyme regulator activity;GO:0052689//carboxylic ester hydrolase activity	GO:0071555//cell wall organization;GO:0000272//polysaccharide catabolic process;GO:0044092//negative regulation of molecular function	gi|356496344|ref|XP_003517028.1|/0/PREDICTED: pectinesterase 2-like [Glycine max]
Glyma.12G054100	-	-	-	-	-	-	-	-	7.17114	0.00017	1943	ko00500//Starch and sucrose metabolism;ko01100//Metabolic pathways;ko01110//Biosynthesis of secondary metabolites;ko00460//Cyanoamino acid metabolism;ko00940//Phenylpropanoid biosynthesis	-	GO:0016798//hydrolase activity, acting on glycosyl bonds	GO:0044238//primary metabolic process	gi|356544535|ref|XP_003540705.1|/0/PREDICTED: cyanogenic beta-glucosidase-like [Glycine max]
Glyma.03G029400	-	-	-	-	-	-	-	-	7.14773	0.00018	1185	ko00040//Pentose and glucuronate interconversions;ko00500//Starch and sucrose metabolism;ko01100//Metabolic pathways	GO:0030312//external encapsulating structure	GO:0030234//enzyme regulator activity;GO:0052689//carboxylic ester hydrolase activity	GO:0071555//cell wall organization;GO:0000272//polysaccharide catabolic process;GO:0044092//negative regulation of molecular function	gi|571444145|ref|XP_006576427.1|/0/PREDICTED: pectinesterase 2-like [Glycine max]
Glyma.19G212600	-1.5405	1.49E-08	-1.4655	1.04E-06	-	-	-	-	5.65883	0.00038	2056	ko00040//Pentose and glucuronate interconversions;ko00500//Starch and sucrose metabolism;ko01100//Metabolic pathways	GO:0030312//external encapsulating structure	GO:0030234//enzyme regulator activity;GO:0052689//carboxylic ester hydrolase activity	GO:0071555//cell wall organization;GO:0000272//polysaccharide catabolic process;GO:0044092//negative regulation of molecular function	gi|571559513|ref|XP_003553658.2|/0/PREDICTED: pectinesterase-like [Glycine max]
Glyma.17G227900	2.13904	0.00323	-	-	-	-	-	-	5.2227	0.00022	2333	ko00500//Starch and sucrose metabolism;ko01100//Metabolic pathways;ko00052//Galactose metabolism	-	GO:0016798//hydrolase activity, acting on glycosyl bonds	GO:0044238//primary metabolic process	gi|947055989|gb|KRH05442.1|;gi|571538935|ref|XP_003549347.2|/0;0/hypothetical protein GLYMA_17G227900 [Glycine max];PREDICTED: fructan 6-exohydrolase-like [Glycine max]
Glyma.20G026700	-	-	-	-	-	-	-	-	5.10843	9.60E-06	3557	ko00500//Starch and sucrose metabolism	-	GO:0043168//anion binding;GO:0004645//phosphorylase activity	GO:0044238//primary metabolic process;GO:0006950//response to stress;GO:0009628//response to abiotic stimulus	gi|571563869|ref|XP_006605545.1|;gi|356577161|ref|XP_003556696.1|/0;0/PREDICTED: alpha-1,4 glucan phosphorylase L-2 isozyme, chloroplastic/amyloplastic-like isoform X2 [Glycine max];PREDICTED: alpha-1,4 glucan phosphorylase L-2 isozyme, chloroplastic/amyloplastic-like isoform X1 [Glycine max]
Glyma.17G067800	-	-	-	-	-	-	-	-	4.33757	5.92E-05	3485	ko00500//Starch and sucrose metabolism	GO:0043231//intracellular membrane-bounded organelle;GO:0044444	-	GO:0005991//trehalose metabolic process	gi|955380139|ref|XP_014625413.1|;gi|947053485|gb|KRH02938.1|/0;0/PREDICTED: probable alpha,alpha-trehalose-phosphate synthase [UDP-forming] 11 [Glycine max];hypothetical protein GLYMA_17G067800 [Glycine max]
Glyma.16G01410	-1.8594	4.37E-13	-	-	-	-	-	-	4.29622	1.01E-06	1890	ko00040//Pentose and glucuronate interconversions;ko00500//Starch and sucrose metabolism;ko01100//Metabolic	GO:0005618//cell wall;GO:0005576//extracellular	GO:0030234//enzyme regulator activity;GO:0052689//c	GO:0071555//cell wall organization;GO:0051707//response to other	gi|955374748|ref|XP_006599763.2|/0/PREDICTED: pectinesterase-like [Glycine max]

0												pathways	region;GO:0016020//membrane;GO:0044424	arboxylic ester hydrolase activity	organism;GO:0000272//polysaccharide catabolic process;GO:0044092//negative regulation of molecular function	
Glyma.13G088300	-	-	-	-	-3.2661	0.0003	-	-	3.84987	0.00013	1763	ko00500//Starch and sucrose metabolism	-	GO:0019203//carbohydrate phosphatase activity	GO:0006796//phosphate-containing compound metabolic process;GO:0005991//trehalose metabolic process	gi|734371039|gb|KHN19585.1|/0/Trehalose-phosphate phosphatase [Glycine soja]
Glyma.18G002200	-	-	-	-	-	-	-	-	3.65548	1.44E-05	1320	ko00500//Starch and sucrose metabolism;ko01100//Metabolic pathways;ko00520//Amino sugar and nucleotide sugar metabolism	GO:0043231//intracellular membrane-bounded organelle	GO:0016854//racemase and epimerase activity;GO:0048037//cofactor binding	GO:0044238//primary metabolic process	gi|947047820|gb|KRG97348.1|/0/hypothetical protein GLYMA_18G002200 [Glycine max]
Glyma.01G203400	-	-	-	-	-	-	-	-	3.47236	0.00056	2041	ko00500//Starch and sucrose metabolism;ko01100//Metabolic pathways	GO:0009532//plastid stroma	GO:0016160//amylase activity	GO:0006950//response to stress;GO:0005976//polysaccharide metabolic process;GO:0000023//maltose metabolic process	gi|571436239|ref|XP_006573703.1|/0/PREDICTED: beta-amylase 3, chloroplastic [Glycine max]
Glyma.11G116600	-	-	-	-	-	-	-	-	3.39944	5.88E-05	2407	ko00500//Starch and sucrose metabolism;ko01100//Metabolic pathways;ko00520//Amino sugar and nucleotide sugar metabolism	GO:0009536//plastid	GO:0070566//adenylyltransferase activity	GO:0005982//starch metabolic process;GO:0005977//glycogen metabolic process	gi|734418875|gb|KHN39792.1|/0/Glucose-1-phosphate adenylyltransferase large subunit 1 [Glycine soja]
Glyma.08G109100	-	-	-	-	-	-	2.08716	6.96E-08	3.27683	3.49E-09	2343	ko00500//Starch and sucrose metabolism;ko01100//Metabolic pathways;ko00520//Amino sugar and nucleotide sugar metabolism	GO:0043231//intracellular membrane-bounded organelle	GO:0016854//racemase and epimerase activity;GO:0048037//cofactor binding	GO:0044238//primary metabolic process	gi|571470896|ref|XP_003532740.2|/0/PREDICTED: UDP-glucuronate 4-epimerase 6-like [Glycine max]
Glyma.06G318400	-	-	-	-	-	-	-	-	3.21001	4.15E-05	2252	ko00500//Starch and sucrose metabolism	-	GO:0019203//carbohydrate phosphatase activity	GO:0006796//phosphate-containing compound metabolic process;GO:0005991//trehalose metabolic process	gi|571462885|ref|XP_006582414.1|;gi|947107964|gb|KRH56347.1|/0;0/PREDICTED: trehalose-phosphate phosphatase A-like [Glycine max];hypothetical protein GLYMA_06G318400 [Glycine max]
Glyma.13G067900	-	-	-	-	-3.5906	2.72E-05	-	-	3.05666	0.00028	1651	ko00500//Starch and sucrose metabolism;ko01110//Biosynthesis of secondary metabolites;ko00520//Amino sugar and nucleotide sugar metabolism	-	GO:0016740//transferase activity	GO:0006950//response to stress	gi|356550372|ref|XP_003543561.1|/0/PREDICTED: probable galacturonosyltransferase-like 10 [Glycine max]
Glyma.08G150400	-	-	-	-	-	-	-	-	3.02677	0.00062	1332	ko00500//Starch and sucrose metabolism;ko01100//Metabolic pathways;ko01110//Biosynthesis of secondary metabolites;ko00460//Cyanoamino acid metabolism;ko00940//Phenylpropanoid biosynthesis	-	GO:0004553//hydrolase activity, hydrolyzing O-glycosyl compounds	GO:0005975//carbohydrate metabolic process	gi|955335734|ref|XP_014634459.1|/0/PREDICTED: vicianin hydrolase [Glycine max]
Glyma.06G104800	-1.1351	0.00392	-	-	-	-	-	-	2.91827	0.00059	2151	ko00500//Starch and sucrose metabolism	-	GO:0019203//carbohydrate phosphatase activity	GO:0006796//phosphate-containing compound metabolic process;GO:0005991//trehalose metabolic process	gi|947104706|gb|KRH53089.1|;gi|947104707|gb|KRH53090.1|;gi|356518491|ref|XP_003527912.1|;gi|571459884|ref|XP_006581545.1|/0;0;0;0/hypothetical protein GLYMA_06G104800 [Glycine max];hypothetical protein GLYMA_06G104800 [Glycine max];PREDICTED: probable trehalose-phosphate phosphatase J isoform X2 [Glycine max];PREDICTED: probable trehalose-phosphate phosphatase J isoform X1 [Glycine max]
Glyma.04G011900	-	-	-	-	-	-	-	-	2.88559	0.0021	2197	ko00500//Starch and sucrose metabolism;ko01100//Metabolic pathways;ko00520//Amino sugar and nucleotide sugar metabolism	GO:0009536//plastid	GO:0070566//adenylyltransferase activity	GO:0005982//starch metabolic process;GO:0005977//glycogen metabolic process	gi|947112523|gb|KRH60825.1|;gi|734405977|gb|KHN33746.1|/0;0/hypothetical protein GLYMA_04G011900 [Glycine max];Glucose-1-phosphate adenylyltransferase large subunit

20 × 20

																1 [Glycine soja]
Glyma.09G073600	-	-	-	-	-	-	-	-	2.70881	9.43E-06	3672	ko00500//Starch and sucrose metabolism;ko01100//Metabolic pathways	-	GO:0035251//UDP-glucosyltransferase activity	GO:0005984//disaccharide metabolic process;GO:0044403//symbiosis, encompassing mutualism through parasitism	gi|356530467|ref|XP_003533802.1|;gi|947088892|gb|KRH37557.1|;gi|947088893|gb|KRH37558.1|/0;0;0/PREDICTED: sucrose synthase [Glycine max];hypothetical protein GLYMA_09G073600 [Glycine max];hypothetical protein GLYMA_09G073600 [Glycine max]
Glyma.04G201600	-	-	3.13964	0.00038	3.22301	0.00034	3.01498	1.71E-06	2.61123	0.00121	1184	ko00500//Starch and sucrose metabolism;ko01100//Metabolic pathways;ko01110//Biosynthesis of secondary metabolites;ko00460//Cyanoamino acid metabolism;ko00940//Phenylpropanoid biosynthesis	-	GO:0016798//hydrolase activity, acting on glycosyl bonds	GO:0044238//primary metabolic process	gi|947115574|gb|KRH63876.1|/3.01797e-58/hypothetical protein GLYMA_04G201600 [Glycine max]
Glyma.01G014000	-1.2944	0.00329	-	-	-2.2916	0.00102	-	-	2.59794	0.00011	3967	ko00500//Starch and sucrose metabolism;ko01100//Metabolic pathways;ko00520//Amino sugar and nucleotide sugar metabolism	GO:0031224//intrinsic component of membrane	GO:0016759//cellulose synthase activity	GO:0030243//cellulose metabolic process;GO:0009856//pollination	gi|571433624|ref|XP_006572955.1|/0/PREDICTED: cellulose synthase-like protein D2 [Glycine max]
Glyma.09G038600	-	-	-	-	-	-	-	-	2.41606	0.00802	3203	ko00500//Starch and sucrose metabolism;ko01100//Metabolic pathways;ko01110//Biosynthesis of secondary metabolites;ko00460//Cyanoamino acid metabolism;ko00940//Phenylpropanoid biosynthesis	GO:0005618//cell wall;GO:0005576//extracellular region	GO:0016798//hydrolase activity, acting on glycosyl bonds	GO:0003006//developmental process involved in reproduction;GO:0044238//primary metabolic process	gi|955340041|ref|XP_003533205.3|;gi|947088349|gb|KRH37014.1|/0;0/PREDICTED: beta-D-xylosidase 1-like [Glycine max];hypothetical protein GLYMA_09G038600 [Glycine max]
Glyma.09G168300	-	-	-	-	-	-	-	-	2.39835	3.74E-05	2221	ko00500//Starch and sucrose metabolism;ko01100//Metabolic pathways	-	GO:0016160//amylase activity	GO:0005976//polysaccharide metabolic process	gi|356531040|ref|XP_003534086.1|/0/PREDICTED: beta-amylase 1, chloroplastic [Glycine max]
Glyma.14G170900	-	-	-	-	-	-	1.46113	5.28E-05	2.3936	9.41E-09	2739	ko00500//Starch and sucrose metabolism;ko01100//Metabolic pathways;ko01110//Biosynthesis of secondary metabolites;ko00460//Cyanoamino acid metabolism;ko00520//Amino sugar and nucleotide sugar metabolism;ko00940//Phenylpropanoid biosynthesis	GO:0005911//cell-cell junction;GO:0005576//extracellular region;GO:0005618//cell wall;GO:0009536//plastid	GO:0016798//hydrolase activity, acting on glycosyl bonds	GO:0044238//primary metabolic process	gi|356552866|ref|XP_003544783.1|;gi|947067550|gb|KRH16693.1|/0;0/PREDICTED: probable beta-D-xylosidase 7 [Glycine max];hypothetical protein GLYMA_14G170900 [Glycine max]
Glyma.17G247900	-	-	-	-	-	-	-	-	2.36796	0.0004	2261	ko00500//Starch and sucrose metabolism;ko01110//Biosynthesis of secondary metabolites;ko00520//Amino sugar and nucleotide sugar metabolism	-	GO:0016740//transferase activity	-	gi|356562321|ref|XP_003549420.1|/0/PREDICTED: probable galacturonosyltransferase-like 1 [Glycine max]
Glyma.15G133700	-	-	-	-	-	-	-	-	2.33848	0.00027	3365	ko00500//Starch and sucrose metabolism;ko01100//Metabolic pathways;ko00052//Galactose metabolism	-	GO:0016798//hydrolase activity, acting on glycosyl bonds;GO:0005488	GO:0044238//primary metabolic process	gi|947062578|gb|KRH11839.1|/0/hypothetical protein GLYMA_15G133700 [Glycine max]
Glyma.15G108000	-	-	-	-	-	-	-	-	2.3366	5.71E-07	4085	ko00500//Starch and sucrose metabolism;ko01100//Metabolic pathways	-	GO:0030247//polysaccharide binding;GO:0046527//glucosyltransferase activity	GO:0006073//cellular glucan metabolic process;GO:0008152//metabolic process	gi|356555668|ref|XP_003546152.1|/0/PREDICTED: starch synthase 3, chloroplastic/amyloplastic-like isoform X3 [Glycine max]
Glyma.02G287600	-	-	-	-	-	-	-	-	2.28077	0.00344	2135	ko00500//Starch and sucrose metabolism;ko01110//Biosynthesis of secondary metabolites;ko00520//Amino sugar and nucleotide sugar metabolism	-	GO:0016740//transferase activity	-	gi|356499376|ref|XP_003518517.1|;gi|955309155|ref|XP_014626011.1|/0;0/PREDICTED: probable galacturonosyltransferase 15 isoform X1 [Glycine max];PREDICTED: probable galacturonosyltransferase 15

																isoform X2 [Glycine max]
Glyma.05G056300	-	-	-	-	-	-	-	-	2.24595	5.03E-05	2656	ko00500//Starch and sucrose metabolism;ko01100//Metabolic pathways;ko00052//Galactose metabolism	GO:0005618//cell wall;GO:0043231//intracellular membrane-bounded organelle	GO:0004558//alpha-1,4-glucosidase activity	GO:0005984//disaccharide metabolic process;GO:0051707//response to other organism;GO:0048364//root development;GO:0009739//response to gibberellin	gi|947109026|gb|KRH57352.1|;gi|947109027|gb|KRH57353.1|;gi|356514218|ref|XP_003525803.1|0;0;0/hypothetical protein GLYMA_05G056300 [Glycine max];hypothetical protein GLYMA_05G056300 [Glycine max];PREDICTED: acid beta-fructofuranosidase [Glycine max]
Glyma.09G077100	-	-	-	-	-	-	-	-	2.24418	0.00039	1790	ko00040//Pentose and glucuronate interconversions;ko00500//Starch and sucrose metabolism;ko01100//Metabolic pathways	GO:0030312//external encapsulating structure	GO:0030234//enzyme regulator activity;GO:0052689//carboxylic ester hydrolase activity	GO:0071555//cell wall organization;GO:0000272//polysaccharide catabolic process;GO:0044092//negative regulation of molecular function	gi|734344035|gb|KHN10379.1|0/Pectinesterase/pectinesterase inhibitor PPE8B [Glycine soja]
Glyma.15G182600	-	-	-	-	-	-	-	-	2.23471	0.00185	3002	ko00500//Starch and sucrose metabolism;ko01100//Metabolic pathways	-	GO:0035251//UDP-glucosyltransferase activity	GO:0005984//disaccharide metabolic process;GO:0044403//symbiosis, encompassing mutualism through parasitism	gi|947063355|gb|KRH12616.1|;gi|571519746|ref|XP_006597891.1|0;0/hypothetical protein GLYMA_15G182600 [Glycine max];PREDICTED: sucrose synthase-like [Glycine max]
Glyma.04G119700	-	-	-	-	-	-	-	-	2.22626	0.00954	1963	ko00500//Starch and sucrose metabolism	-	GO:0019203//carbohydrate phosphatase activity	GO:0006796//phosphate-containing compound metabolic process;GO:0005991//trehalose metabolic process	gi|571450244|ref|XP_006578369.1|0/PREDICTED: trehalose-phosphate phosphatase A isoform X2 [Glycine max]
Glyma.06G314200	-1.2084	6.28E-08	-	-	-	-	-	-	2.17888	0.00652	2067	ko00040//Pentose and glucuronate interconversions;ko00500//Starch and sucrose metabolism;ko01100//Metabolic pathways	GO:0030312//external encapsulating structure	GO:0030234//enzyme regulator activity;GO:0052689//carboxylic ester hydrolase activity	GO:0071555//cell wall organization;GO:0000272//polysaccharide catabolic process;GO:0044092//negative regulation of molecular function	gi|955326411|ref|XP_003527536.3|0/PREDICTED: probable pectinesterase/pectinesterase inhibitor 47 [Glycine max]
Glyma.12G042400	-	-	-	-	-	-	-	-	2.1595	0.00219	2521	ko00500//Starch and sucrose metabolism;ko01100//Metabolic pathways;ko00520//Amino sugar and nucleotide sugar metabolism	GO:0009536//plastid	GO:0070566//adenylyltransferase activity	GO:0005982//starch metabolic process;GO:0005977//glycogen metabolic process	gi|734387621|gb|KHN25384.1|0/Glucose-1-phosphate adenylyltransferase large subunit 1 [Glycine soja]
Glyma.06G128500	-	-	-	-	-	-	-	-	2.14733	2.52E-05	2378	ko00040//Pentose and glucuronate interconversions;ko00500//Starch and sucrose metabolism;ko01100//Metabolic pathways	GO:0030312//external encapsulating structure	GO:0030234//enzyme regulator activity;GO:0052689//carboxylic ester hydrolase activity	GO:0071555//cell wall organization;GO:0000272//polysaccharide catabolic process;GO:0044092//negative regulation of molecular function	gi|356516053|ref|XP_003526711.1|0/PREDICTED: probable pectinesterase/pectinesterase inhibitor 34 [Glycine max]
Glyma.06G034600	-	-	-	-	-	-	-	-	2.10368	0.00783	1909	ko00500//Starch and sucrose metabolism;ko01110//Biosynthesis of secondary metabolites;ko00520//Amino sugar and nucleotide sugar metabolism	-	GO:0016740//transferase activity	GO:0003002//regionalization;GO:0042545//cell wall modification;GO:0006950//response to stress;GO:0048827//phyllome development;GO:0000003//reproduction;GO:0010413//glucuronoxylan metabolic process	gi|356519066|ref|XP_003528195.1|0/PREDICTED: probable galacturonosyltransferase-like 1 [Glycine max]
Glyma.01G232500	-	-	-	-	-	-	-	-	2.09502	0.00381	3949	ko00500//Starch and sucrose metabolism;ko01100//Metabolic pathways;ko00520//Amino sugar and nucleotide sugar metabolism	GO:0031224//intrinsic component of membrane	GO:0016759//cellulose synthase activity	GO:0030243//cellulose metabolic process	gi|356497399|ref|XP_003517548.1|0/PREDICTED: cellulose synthase-like protein D2 [Glycine max]
Glyma.09G071500	-	-	-	-	-	-	-	-	2.09074	0.00212	2110	ko00040//Pentose and glucuronate interconversions;ko00500//Starch and sucrose metabolism;ko01100//Metabolic pathways	-	GO:0004553//hydrolase activity, hydrolyzing O-glycosyl compounds	GO:0044238//primary metabolic process	gi|356530447|ref|XP_003533792.1|0/PREDICTED: probable polygalacturonase [Glycine max]
Glyma.08G180900	-	-	-	-	-	-	-	-	2.08141	0.00109	2807	ko00500//Starch and sucrose metabolism;ko01100//Metabolic pathways;ko00520//Amino sugar and nucleotide sugar metabolism	GO:0031012//extracellular matrix	GO:0004553//hydrolase activity, hydrolyzing O-glycosyl compounds	GO:0000272//polysaccharide catabolic process	gi|734390440|gb|KHN26728.1|;gi|356525896|ref|XP_003531557.1|0;0/Beta-xylosidase/alpha-L-arabinofuranosidase 1 [Glycine soja];PREDICTED: beta-xylosidase/alpha-L-arabinofuranosidase 1 [Glycine max]

Glyma.09G028000	-	-	-	-	-	-	-	-	2.06765	0.00718	3312	ko00500//Starch and sucrose metabolism;ko01100//Metabolic pathways;ko00052//Galactose metabolism	-	GO:0016798//hydrolase activity, acting on glycosyl bonds;GO:0005488	GO:0044238//primary metabolic process	gi|734428229|gb|KHN44656.1|/0/Alpha-glucosidase [Glycine soja]
Glyma.19G113600	-	-	-	-	-	-	-	-	2.05127	0.00029	2481	ko00500//Starch and sucrose metabolism;ko01100//Metabolic pathways;ko01110//Biosynthesis of secondary metabolites;ko00460//Cyanoamino acid metabolism;ko00940//Phenylpropanoid biosynthesis	-	GO:0016798//hydrolase activity, acting on glycosyl bonds	GO:0044238//primary metabolic process	gi|947045227|gb|KRG94856.1|;gi|571556301|ref|XP_006604248.1|/0;0/hypothetical protein GLYMA_19G113600 [Glycine max];PREDICTED: beta-glucosidase BoGH3B-like [Glycine max]
Glyma.15G143700	-1.1756	4.93E-06	-	-	-	-	-	-	2.03865	0.00115	2644	ko00500//Starch and sucrose metabolism;ko01100//Metabolic pathways;ko01110//Biosynthesis of secondary metabolites;ko00460//Cyanoamino acid metabolism;ko00940//Phenylpropanoid biosynthesis	GO:0005618//cell wall;GO:0005576//extracellular region	GO:0016798//hydrolase activity, acting on glycosyl bonds	GO:0003006//developmental process involved in reproduction;GO:0044238//primary metabolic process	gi|947062724|gb|KRH11985.1|;gi|356556038|ref|XP_003546334.1|/0;0/hypothetical protein GLYMA_15G143700 [Glycine max];PREDICTED: beta-D-xylosidase 1 [Glycine max]
Glyma.15G098100	-	-	-	-	-	-	-	-	1.9716	0.00519	2035	ko00500//Starch and sucrose metabolism;ko01100//Metabolic pathways	-	GO:0016160//amylase activity	GO:0005976//polysaccharide metabolic process	gi|351726594|ref|NP_001236364.1|/0/inactive beta-amylase-like [Glycine max]
Glyma.07G066900	-	-	-	-	-	-	-	-	1.93725	0.00184	2125	ko00040//Pentose and glucuronate interconversions;ko00500//Starch and sucrose metabolism;ko01100//Metabolic pathways	-	GO:0004553//hydrolase activity, hydrolyzing O-glycosyl compounds	GO:0044238//primary metabolic process	gi|356520375|ref|XP_003528838.1|/0/PREDICTED: probable polygalacturonase [Glycine max]
Glyma.19G113500	-	-	-	-	-	-	-	-	1.87299	0.00566	2193	ko00500//Starch and sucrose metabolism;ko01100//Metabolic pathways;ko01110//Biosynthesis of secondary metabolites;ko00460//Cyanoamino acid metabolism;ko00940//Phenylpropanoid biosynthesis	-	GO:0016798//hydrolase activity, acting on glycosyl bonds	GO:0044238//primary metabolic process	gi|571556289|ref|XP_006604246.1|/0/PREDICTED: beta-glucosidase BoGH3B-like [Glycine max]
Glyma.05G127800	-	-	-	-	-	-	-	-	1.83383	0.006	3636	ko00500//Starch and sucrose metabolism;ko01100//Metabolic pathways	-	GO:0046527//glucosyltransferase activity	GO:0006073//cellular glucan metabolic process	gi|356512165|ref|XP_003524791.1|/0/PREDICTED: probable starch synthase 4, chloroplastic/amyloplastic [Glycine max]
Glyma.08G296800	-	-	-	-	-	-	-	-	1.78994	1.57E-06	3346	ko00500//Starch and sucrose metabolism;ko01100//Metabolic pathways	-	GO:0046872//metal ion binding;GO:0016160//amylase activity	GO:0044238//primary metabolic process	gi|356526892|ref|XP_003532050.1|/0/PREDICTED: alpha-amylase 3, chloroplastic isoform X1 [Glycine max]
Glyma.16G014000	-	-	-	-	-	-	-	-	1.75683	0.00243	2123	ko00040//Pentose and glucuronate interconversions;ko00500//Starch and sucrose metabolism;ko01100//Metabolic pathways	GO:0005911//cell-cell junction;GO:0016020//membrane;GO:0030312//external encapsulating structure;GO:0044437	GO:0030234//enzyme regulator activity;GO:0052689//carboxylic ester hydrolase activity	GO:0071555//cell wall organization;GO:0000272//polysaccharide catabolic process;GO:0044092//negative regulation of molecular function	gi|356559248|ref|XP_003547912.1|/0/PREDICTED: pectinesterase 3 [Glycine max]
Glyma.11G254900	-	-	-	-	-	-	-	-	1.75178	0.0003	2470	ko00500//Starch and sucrose metabolism;ko01100//Metabolic pathways;ko00520//Amino sugar and nucleotide sugar metabolism	GO:0043231//intracellular membrane-bounded organelle	GO:0016854//racemase and epimerase activity;GO:0048037//cofactor binding	GO:0044238//primary metabolic process	gi|356540097|ref|XP_003538527.1|/0/PREDICTED: UDP-glucuronate 4-epimerase 6-like [Glycine max]
Glyma.13G215000	-	-	-	-	-1.7371	0.00033	-	-	1.74753	0.00367	2055	ko00500//Starch and sucrose metabolism;ko01100//Metabolic pathways	-	GO:0016160//amylase activity	GO:0005976//polysaccharide metabolic process	gi|356549058|ref|XP_003542915.1|/0/PREDICTED: inactive beta-amylase 9 [Glycine max]
Glyma.16G033000	-	-	-	-	-	-	-	-	1.69414	0.00232	3241	ko00040//Pentose and glucuronate interconversions;ko00500//Starch and sucrose metabolism;ko01100//Metabolic pathways	-	GO:0004553//hydrolase activity, hydrolyzing O-glycosyl compounds	GO:0044238//primary metabolic process	gi|571525292|ref|XP_003548644.2|;gi|571525296|ref|XP_006598941.1|/0;0/PREDICTED: probable polygalacturonase isoform X1 [Glycine max];PREDICTED: probable polygalacturonase

20 × 20

																isoform X2 [Glycine max]
Glyma.02G208000	-	-	-	-	-	-	-	-	1.64538	0.00233	2696	ko00500//Starch and sucrose metabolism;ko01100//Metabolic pathways;ko00520//Amino sugar and nucleotide sugar metabolism	-	GO:0048037//cofactor binding;GO:0016857//racemase and epimerase activity, acting on carbohydrates and derivatives	GO:0044238//primary metabolic process	gi|356500703|ref|XP_003519171.1|/0/PREDICTED: UDP-glucuronate 4-epimerase 1-like [Glycine max]
Glyma.05G152300	-	-	-	-	-	-	-	-	1.57244	0.00724	1879	ko00500//Starch and sucrose metabolism;ko01100//Metabolic pathways;ko00520//Amino sugar and nucleotide sugar metabolism	GO:0043231//intracellular membrane-bounded organelle	GO:0016854//racemase and epimerase activity;GO:0048037//cofactor binding	GO:0044238//primary metabolic process	gi|356512447|ref|XP_003524930.1|;gi|947110527|gb|KRH58853.1|/0;0/PREDICTED: UDP-glucuronate 4-epimerase 6-like [Glycine max];hypothetical protein GLYMA_05G152300 [Glycine max]
Glyma.04G030300	-	-	-	-	-	-	-	-	1.47644	0.00046	2955	ko00500//Starch and sucrose metabolism;ko01100//Metabolic pathways;ko00520//Amino sugar and nucleotide sugar metabolism	GO:0009536//plastid	GO:0070566//adenylyltransferase activity	GO:0005982//starch metabolic process;GO:0005977//glycogen metabolic process	gi|947112837|gb|KRH61139.1|;gi|734431667|gb|KHN45868.1|;gi|947112838|gb|KRH61140.1|;gi|571448915|ref|XP_006577992.1|/0;0;0;0/hypothetical protein GLYMA_04G030300 [Glycine max];Glucose-1-phosphate adenylyltransferase large subunit, chloroplastic/amyloplastic [Glycine soja] ;hypothetical protein GLYMA_04G030300 [Glycine max];PREDICTED: glucose-1-phosphate adenylyltransferase large subunit, chloroplastic/amyloplastic-like isoform X2 [Glycine max]
Glyma.18G265100	-	-	-	-	-	-	-	-	1.46292	0.00946	1846	ko00053//Ascorbate and aldarate metabolism;ko00040//Pentose and glucuronate interconversions;ko00500//Starch and sucrose metabolism;ko01100//Metabolic pathways;ko01110//Biosynthesis of secondary metabolites;ko00520//Amino sugar and nucleotide sugar metabolism	GO:0043231//intracellular membrane-bounded organelle;GO:0044444;GO:0005576//extracellular region	GO:0016616//oxidoreductase activity, acting on the CH-OH group of donors, NAD or NADP as acceptor;GO:0000166//nucleotide binding	GO:0009226//nucleotide-sugar biosynthetic process;GO:0009664//plant-type cell wall organization	gi|734354133|gb|KHN13571.1|/0/UDP-glucose 6-dehydrogenase [Glycine soja]
Glyma.17G069600	-	-	-	-	-	-	-	-	1.37586	0.00137	2621	ko00500//Starch and sucrose metabolism;ko01100//Metabolic pathways;ko00520//Amino sugar and nucleotide sugar metabolism	-	GO:0048037//cofactor binding;GO:0016857//racemase and epimerase activity, acting on carbohydrates and derivatives	GO:0044238//primary metabolic process	gi|356562525|ref|XP_003549520.1|/0/PREDICTED: UDP-glucuronate 4-epimerase 1 [Glycine max]
Glyma.01G037900	-	-	-	-	-	-	-	-	1.31205	0.00317	2201	ko00500//Starch and sucrose metabolism;ko01110//Biosynthesis of secondary metabolites;ko00520//Amino sugar and nucleotide sugar metabolism	-	GO:0016740//transferase activity	-	gi|356498063|ref|XP_003517873.1|/0/PREDICTED: probable galacturonosyltransferase-like 9 [Glycine max]
Glyma.14G159600	-	-	-	-	-	-	-	-	1.25984	0.00081	2387	ko00040//Pentose and glucuronate interconversions;ko00500//Starch and sucrose metabolism;ko01100//Metabolic pathways	GO:0030312//external encapsulating structure	GO:0030234//enzyme regulator activity;GO:0052689//carboxylic ester hydrolase activity	GO:0071555//cell wall organization;GO:0000272//polysaccharide catabolic process;GO:0044092//negative regulation of molecular function	gi|955364443|ref|XP_014622596.1|/0/PREDICTED: probable pectinesterase/pectinesterase inhibitor 61 isoform X1 [Glycine max]
Glyma.06G129400	-	-	-	-	-	-	-	-	1.14575	0.0104	2048	ko00500//Starch and sucrose metabolism;ko01100//Metabolic pathways	GO:0009532//plastid stroma	GO:0046527//glucosyltransferase activity	GO:0009059//macromolecule biosynthetic process;GO:0006073//cellular glucan metabolic process	gi|947105123|gb|KRH53506.1|/0/hypothetical protein GLYMA_06G129400, partial [Glycine max]
Glyma.13G161600	-	-	-	-	-	-	-	-	1.09513	0.00125	4100	ko00500//Starch and sucrose metabolism;ko01100//Metabolic pathways	GO:0005911//cell-cell junction;GO:0044444;GO:0016020//membrane	GO:0035251//UDP-glucosyltransferase activity	GO:0005985//sucrose metabolic process	gi|947071288|gb|KRH20179.1|;gi|356548520|ref|XP_003542649.1|/0;0/hypothetical protein GLYMA_13G161600 [Glycine max];PREDICTED: probable

																sucrose-phosphate synthase [Glycine max]
Glyma.11G010400	-	-	-	-	-	-	-	-	1.01237	0.01038	4001	ko00500//Starch and sucrose metabolism;ko01100//Metabolic pathways;ko00520//Amino sugar and nucleotide sugar metabolism	GO:0031224//intrinsic component of membrane	GO:0016759//cellulose synthase activity	GO:0030243//cellulose metabolic process	gi|356538575|ref|XP_003537778.1|/0/PREDICTED: cellulose synthase-like protein D2 [Glycine max]
Glyma.18G262500	-	-	-	-	-	-	-	-	-1.0727	0.00386	1745	ko00040//Pentose and glucuronate interconversions;ko00500//Starch and sucrose metabolism;ko01100//Metabolic pathways	GO:0030312//external encapsulating structure	GO:0052689//carboxylic ester hydrolase activity	GO:0071555//cell wall organization;GO:0010393	gi|571549278|ref|XP_006602929.1|/0/PREDICTED: pectinesterase 31-like [Glycine max]
Glyma.02G226600	-	-	-	-	-	-	-	-	-1.0739	0.00302	1688	ko00051//Fructose and mannose metabolism;ko00500//Starch and sucrose metabolism;ko01100//Metabolic pathways;ko00520//Amino sugar and nucleotide sugar metabolism	-	GO:0016772//transferase activity, transferring phosphorus-containing groups	GO:0032958//inositol phosphate biosynthetic process	gi|571441318|ref|XP_003518306.2|/0/PREDICTED: inositol 3-kinase-like [Glycine max]
Glyma.12G240000	-	-	-	-	-	-	-	-	-1.075	0.00544	1941	ko00500//Starch and sucrose metabolism;ko01100//Metabolic pathways;ko01110//Biosynthesis of secondary metabolites;ko00460//Cyanoamino acid metabolism;ko00940//Phenylpropanoid biosynthesis	GO:0005618//cell wall;GO:0043231//intracellular membrane-bounded organelle;GO:0005911//cell-cell junction	GO:0016798//hydrolase activity, acting on glycosyl bonds	GO:0071704//organic substance metabolic process;GO:0044238//primary metabolic process;GO:0000904//cell morphogenesis involved in differentiation	gi|734370810|gb|KHN19426.1|/0/Beta-glucosidase 13 [Glycine soja]
Glyma.04G008400	-	-	-	-	-	-	-	-	-1.5185	0.00171	2008	ko00500//Starch and sucrose metabolism;ko01100//Metabolic pathways	-	GO:0016798//hydrolase activity, acting on glycosyl bonds	GO:0044238//primary metabolic process	gi|356509549|ref|XP_003523510.1|/0/PREDICTED: endoglucanase 24-like [Glycine max]
Glyma.05G236600	-	-	-	-	-	-	-	-	-1.618	0.00341	1125	ko00040//Pentose and glucuronate interconversions;ko00500//Starch and sucrose metabolism;ko01100//Metabolic pathways	-	GO:0030234//enzyme regulator activity;GO:0052689//carboxylic ester hydrolase activity	GO:0044092//negative regulation of molecular function	gi|356513076|ref|XP_003525240.1|/2.77858e-145/PREDICTED: 21 kDa protein-like [Glycine max]
Glyma.17G044700	-	-	-	-	-	-	-1.7428	3.65E-06	-1.7283	8.46E-05	2227	ko00040//Pentose and glucuronate interconversions;ko00500//Starch and sucrose metabolism;ko01100//Metabolic pathways	GO:0030312//external encapsulating structure	GO:0030234//enzyme regulator activity;GO:0052689//carboxylic ester hydrolase activity	GO:0071555//cell wall organization;GO:0000272//polysaccharide catabolic process;GO:0044092//negative regulation of molecular function	gi|356564706|ref|XP_003550590.1|/0/PREDICTED: putative pectinesterase/pectinesterase inhibitor 45 [Glycine max]
Glyma.05G163600	-	-	-	-	-	-	-	-	-2.3975	0.00121	3005	ko00500//Starch and sucrose metabolism	-	GO:0019203//carbohydrate phosphatase activity;GO:0035251//UDP-glucosyltransferase activity	GO:0006796//phosphate-containing compound metabolic process;GO:0005991//trehalose metabolic process	gi|356510997|ref|XP_003524218.1|/0/PREDICTED: alpha,alpha-trehalose-phosphate synthase [UDP-forming] 1-like [Glycine max]
Glyma.06G008200	-	-	-	-	-	-	-	-	-2.5764	9.36E-06	1895	ko00500//Starch and sucrose metabolism;ko01100//Metabolic pathways	-	GO:0016798//hydrolase activity, acting on glycosyl bonds	GO:0044238//primary metabolic process	gi|356517881|ref|XP_003527614.1|/0/PREDICTED: endoglucanase 24-like [Glycine max]

Supplementary Table S6: DEGs involved in defense mechanisms

<table>
<tr><th>GeneID</th><th>log2Ratio(2mlpa-1/2MWT-1)</th><th>P-value</th><th>log2Ratio(2mlpa-2/2MWT-2)</th><th>P-value</th><th>log2Ratio(2mlpa-3/2MWT-3)</th><th>P-value</th><th>log2Ratio(2mlpa-4/2MWT-4)</th><th>P-value</th><th>log2Ratio(2mlpa-5/2MWT-5)</th><th>P-value</th><th>Length</th><th>Pathway</th><th>GO Component</th><th>GO Function</th><th>GO Process</th><th>Blast nr</th></tr>
<tr><td>Glyma.U035700</td><td>2.43731</td><td>7.73E-15</td><td>1.31096</td><td>0.000149</td><td>1.15885</td><td>0.00026</td><td>-</td><td>-</td><td>2.00447</td><td>0.00032</td><td>3324</td><td>ko04626//Plant-pathogen interaction</td><td>-</td><td>GO:0016462//pyrophosphatase activity;GO:0032550;GO:0036094//small molecule binding;GO:1901363;GO:0097159//organic cyclic compound binding</td><td>GO:0006950//response to stress;GO:0007154//cell communication</td><td>gi|947038010|gb|KRG88494.1|;gi|947038009|gb|KRG88493.1|;gi|947038006|gb|KRG88490.1|;gi|955395634|ref|XP_014628764.1|;gi|947038007|gb|KRG88491.1|;gi|947038002|gb|KRG88486.1|;gi|947038005|gb|KRG88489.1|;gi|947038004|gb|KRG88488.1|;gi|947038003|gb|KRG88487.1|/0;0;0;0;0;0;0;0;0/hypothetical protein GLYMA_U035700, partial [Glycine max];hypothetical protein GLYMA_U035700, partial [Glycine max];hypothetical protein GLYMA_U035700, partial [Glycine max];PREDICTED: TMV resistance protein N-like, partial [Glycine max];hypothetical protein GLYMA_U035700, partial [Glycine max];hypothetical protein GLYMA_U035700, partial [Glycine max];hypothetical protein GLYMA_U035700, partial [Glycine max];hypothetical protein GLYMA_U035700, partial [Glycine max];hypothetical protein GLYMA_U035700, partial [Glycine max]</td></tr>
<tr><td>Glyma.20G061300</td><td>-</td><td>-</td><td>-</td><td>-</td><td>-</td><td>-</td><td>-</td><td>-</td><td>2.77513</td><td>0.00674</td><td>3489</td><td>ko04626//Plant-pathogen interaction</td><td>-</td><td>GO:0032550</td><td>GO:0006950//response to stress;GO:0007154//cell communication</td><td>gi|947040285|gb|KRG90009.1|;gi|947040287|gb|KRG90011.1|;gi|947040286|gb|KRG90010.1|/0;0;0/hypothetical protein GLYMA_20G061300 [Glycine max];hypothetical protein GLYMA_20G061300 [Glycine max];hypothetical protein GLYMA_20G061300 [Glycine max]</td></tr>
<tr><td>Glyma.20G050700</td><td>-1.3244</td><td>6.58E-05</td><td>-</td><td>-</td><td>-</td><td>-</td><td>-</td><td>-</td><td>-</td><td>-</td><td>4266</td><td>ko00230//Purine metabolism;ko00240//Pyrimidine metabolism;ko01100//Metabolic pathways;ko03020//RNA polymerase;ko04626//Plant-pathogen interaction</td><td>-</td><td>GO:0032550</td><td>GO:0006950//response to stress</td><td>gi|947040118|gb|KRG89842.1|;gi|356577440|ref|XP_003556833.1|/0;0/hypothetical protein GLYMA_20G050700 [Glycine max];PREDICTED: putative disease resistance RPP13-like protein 1 isoform X1 [Glycine max]</td></tr>
<tr><td>Glyma.18G086900</td><td>-2.307</td><td>0.000137</td><td>-</td><td>-</td><td>-3.6406</td><td>9.15E-07</td><td>-</td><td>-</td><td>-</td><td>-</td><td>3010</td><td>ko04626//Plant-pathogen interaction</td><td>-</td><td>GO:0032550</td><td>GO:0006950//response to stress</td><td>gi|947049112|gb|KRG98640.1|/0/hypothetical protein GLYMA_18G086900 [Glycine max]</td></tr>
<tr><td>Glyma.18G086600</td><td>-</td><td>-</td><td>-</td><td>-</td><td>-</td><td>-</td><td>-</td><td>-</td><td>3.16077</td><td>5.28E-05</td><td>3221</td><td>ko04626//Plant-pathogen interaction</td><td>-</td><td>GO:0032550</td><td>GO:0006950//response to stress</td><td>gi|955384422|ref|XP_014626398.1|/0/PREDICTED: disease resistance protein RPM1-like [Glycine max]</td></tr>
<tr><td>Glyma.17G245500</td><td>-</td><td>-</td><td>-</td><td>-</td><td>-</td><td>-</td><td>-</td><td>-</td><td>2.83107</td><td>0.00242</td><td>2929</td><td>ko00230//Purine metabolism;ko00240//Pyrimidine metabolism;ko01100//Metabolic pathways;ko03020//RNA polymerase;ko04626//Plant-pathogen interaction</td><td>-</td><td>GO:0032550</td><td>GO:0009617//response to bacterium;GO:0009628//response to abiotic stimulus;GO:0006950//response to stress</td><td>gi|356564213|ref|XP_003550350.1|/0/PREDICTED: probable disease resistance protein At4g33300 [Glycine max]</td></tr>
</table>

20 × 20

Glyma.17G180100	-	-	-	-	-2.4985	0.00028	-	-	1.83128	0.00059	3836	ko00230//Purine metabolism;ko00240//Pyrimidine metabolism;ko01100//Metabolic pathways;ko03020//RNA polymerase;ko04626//Plant-pathogen interaction	-	GO:0032550	GO:0006950//response to stress	gi|571537529|ref|XP_006601011.1|/0/PREDICTED: probable disease resistance protein At5g66900 [Glycine max]
Glyma.16G214900	4.65642	9.36E-30	3.54707	1.78E-10	3.92223	3.43E-16	3.89277	1.63E-15	3.15223	2.29E-05	2927	ko04626//Plant-pathogen interaction	-	GO:0032550;GO:0016462//pyrophosphatase activity	GO:0050896//response to stimulus;GO:0006950//response to stress;GO:0007154//cell communication	gi|947060016|gb|KRH09422.1|;gi|571530234|ref|XP_006599700.1|;gi|947060020|gb|KRH09426.1|/0;0;0/hypothetical protein GLYMA_16G214900 [Glycine max];PREDICTED: TMV resistance protein N-like isoform X4 [Glycine max];hypothetical protein GLYMA_16G214900 [Glycine max]
Glyma.16G214800	-	-	-	-	-	-	-	-	2.03844	0.00432	1197	ko04626//Plant-pathogen interaction	-	GO:0046872//metal ion binding;GO:0016462//pyrophosphatase activity;GO:0032550	GO:0006950//response to stress;GO:0007154//cell communication	gi|947060014|gb|KRH09420.1|/0/hypothetical protein GLYMA_16G214800 [Glycine max]
Glyma.16G213700	-	-	-	-	-	-	-	-	4.79466	3.85E-08	3445	ko04626//Plant-pathogen interaction	-	GO:0032550	GO:0006950//response to stress;GO:0007154//cell communication;GO:0050896//response to stimulus	gi|571530198|ref|XP_006599690.1|;gi|571530196|ref|XP_006599689.1|;gi|955374931|ref|XP_014624138.1|/0;0;0/PREDICTED: TMV resistance protein N-like isoform X2 [Glycine max];PREDICTED: TMV resistance protein N-like isoform X1 [Glycine max];PREDICTED: TMV resistance protein N-like isoform X3 [Glycine max]
Glyma.16G210800	-	-	-	-	-	-	-	-	3.60977	0.00024	4638	ko00230//Purine metabolism;ko00240//Pyrimidine metabolism;ko01100//Metabolic pathways;ko03020//RNA polymerase;ko04626//Plant-pathogen interaction	-	GO:0032550;GO:0036094//small molecule binding;GO:1901363;GO:0097159//organic cyclic compound binding;GO:0016462//pyrophosphatase activity	GO:0050896//response to stimulus;GO:0006950//response to stress;GO:0007154//cell communication	gi|947059932|gb|KRH09338.1|;gi|947059931|gb|KRH09337.1|;gi|351724311|ref|NP_001237821.1|/0;0;0/hypothetical protein GLYMA_16G210800 [Glycine max];hypothetical protein GLYMA_16G210800 [Glycine max];candidate disease-resistance protein [Glycine max]
Glyma.16G210600	-	-	-	-	-	-	-	-	3.33939	0.00524	4549	ko00230//Purine metabolism;ko00240//Pyrimidine metabolism;ko01100//Metabolic pathways;ko03020//RNA polymerase;ko04626//Plant-pathogen interaction	-	GO:0032550;GO:0016462//pyrophosphatase activity	GO:0050896//response to stimulus;GO:0006950//response to stress;GO:0007154//cell communication	gi|356559997|ref|XP_003548282.1|;gi|947059928|gb|KRH09334.1|;gi|947059927|gb|KRH09333.1|/0;0;0/PREDICTED: TMV resistance protein N [Glycine max];hypothetical protein GLYMA_16G210600 [Glycine max];hypothetical protein GLYMA_16G210600 [Glycine max]
Glyma.16G087100	-	-	-	-	-	-	-	-	2.42611	0.00242	3617	ko00230//Purine metabolism;ko00240//Pyrimidine metabolism;ko01100//Metabolic pathways;ko03020//RNA polymerase;ko04626//Plant-pathogen interaction	-	GO:0032550	GO:0006950//response to stress;GO:0007154//cell communication;GO:0050896//response to stimulus	gi|571526878|ref|XP_006599163.1|;gi|947058007|gb|KRH07413.1|/0;0/PREDICTED: TMV resistance protein N-like [Glycine max];hypothetical protein GLYMA_16G087100 [Glycine max]
Glyma.16G086700	-	-	-	-	-	-	-	-	3.72714	0.00035	3682	ko00230//Purine metabolism;ko00240//Pyrimidine metabolism;ko01100//Metabolic	-	GO:0032550	GO:0006950//response to stress;GO:0007154//cell communication	gi|571526864|ref|XP_006599159.1|/0/PREDICTED: TMV resistance protein N-like [Glycine max]

												pathways;ko03020//RNA polymerase;ko04626//Plant-pathogen interaction				
Glyma.16G033900	-	-	-	-	-	-	-	-	1.22042	0.00321	3868	ko00230//Purine metabolism;ko00240//Pyrimidine metabolism;ko01100//Metabolic pathways;ko03020//RNA polymerase;ko04626//Plant-pathogen interaction	-	GO:0032550	GO:0006950//response to stress;GO:0007154//cell communication	gi|571525333|ref|XP_006598947.1|;gi|947057200|gb|KRH06606.1|;gi|571525345|ref|XP_006598950.1|/0;0;0/PREDICTED: TMV resistance protein N-like isoform X1 [Glycine max];hypothetical protein GLYMA_16G033900 [Glycine max];PREDICTED: TMV resistance protein N-like isoform X2 [Glycine max]
Glyma.16G006400	-	-	-	-	-	-	-	-	4.05615	8.10E-05	3809	ko00230//Purine metabolism;ko00240//Pyrimidine metabolism;ko01100//Metabolic pathways;ko03020//RNA polymerase;ko04626//Plant-pathogen interaction	-	GO:0032550	GO:0006950//response to stress;GO:0007154//cell communication	gi|571524432|ref|XP_006598818.1|;gi|947056747|gb|KRH06153.1|/0;0/PREDICTED: putative disease resistance protein At4g11170 [Glycine max];hypothetical protein GLYMA_16G006400 [Glycine max]
Glyma.15G230700	-	-	-	-	-	-	-	-	2.59016	0.00068	4001	ko00230//Purine metabolism;ko00240//Pyrimidine metabolism;ko01100//Metabolic pathways;ko03020//RNA polymerase;ko04626//Plant-pathogen interaction	-	GO:0032550	GO:0006950//response to stress	gi|571520977|ref|XP_006598091.1|;gi|947064056|gb|KRH13317.1|/0;0/PREDICTED: putative disease resistance protein At3g14460 isoform X1 [Glycine max];hypothetical protein GLYMA_15G230700 [Glycine max]
Glyma.15G168500	-	-	-	-	-	-	-	-	3.41448	8.30E-05	3138	ko04626//Plant-pathogen interaction	-	GO:0016462//pyrophosphatase activity;GO:0032550	GO:0006950//response to stress;GO:0008152//metabolic process	gi|571513784|ref|XP_006596935.1|;gi|351727306|ref|NP_001237924.1|/0;0/PREDICTED: CC-NBS-LRR class disease resistance protein isoform X1 [Glycine max];CC-NBS-LRR class disease resistance protein [Glycine max]
Glyma.15G127100	-1.8283	3.99E-05	-	-	-	-	-	-	-	-	3857	ko00230//Purine metabolism;ko00240//Pyrimidine metabolism;ko01100//Metabolic pathways;ko03020//RNA polymerase;ko04626//Plant-pathogen interaction	-	GO:0032550	GO:0006950//response to stress	gi|571522550|ref|XP_006598349.1|/0/PREDICTED: putative disease resistance protein RGA3 [Glycine max]
Glyma.14G079600	-	-	-	-	-	-	-	-	1.61914	0.00122	2732	ko00230//Purine metabolism;ko00240//Pyrimidine metabolism;ko01100//Metabolic pathways;ko03020//RNA polymerase;ko04626//Plant-pathogen interaction	-	GO:0032550	GO:0009617//response to bacterium;GO:0009628//response to abiotic stimulus;GO:0006950//response to stress	gi|356552172|ref|XP_003544443.1|/0/PREDICTED: probable disease resistance protein At4g33300 [Glycine max]
Glyma.14G079500	-	-	-	-	-	-	-	-	3.46527	0.00046	3226	ko00230//Purine metabolism;ko00240//Pyrimidine metabolism;ko01100//	-	GO:0032550	GO:0009617//response to bacterium;GO:0009628//response to abiotic stimulus;GO:0006950//respon	gi|947066147|gb|KRH15290.1|;gi|571508169|ref|XP_003544442.2|/0;0/hypothetical protein GLYMA_14G079500 [Glycine max];PREDICTED: probable disease

20 × 20

												Metabolic pathways;ko03020//RNA polymerase;ko04626//Plant-pathogen interaction			se to stress	resistance protein At4g33300 [Glycine max]
Glyma.13G285500	-	-	-	-	-	-	-	-	2.08844	0.00018	2506	ko04626//Plant-pathogen interaction	GO:0016020//membrane	GO:0032550;GO:0005515//protein binding;GO:0004702//receptor signaling protein serine/threonine kinase activity	GO:0035556//intracellular signal transduction;GO:0032147//activation of protein kinase activity;GO:0006950//response to stress	gi|356549811|ref|XP_003543284.1|/0/PREDICTED: probable leucine-rich repeat receptor-like serine/threonine-protein kinase At5g15730 [Glycine max]
Glyma.13G193300	-	-	-	-	-	-	-	-	2.68408	0.00202	2962	ko00230//Purine metabolism;ko00240//Pyrimidine metabolism;ko01100//Metabolic pathways;ko03020//RNA polymerase;ko04626//Plant-pathogen interaction	-	GO:0032550	GO:0006950//response to stress	gi|947071784|gb|KRH20675.1|/0/hypothetical protein GLYMA_13G193300 [Glycine max]
Glyma.13G190800	-2.642	0.000329	-	-	-	-	-	-	-	-	6542	ko00230//Purine metabolism;ko00240//Pyrimidine metabolism;ko01100//Metabolic pathways;ko03020//RNA polymerase;ko04626//Plant-pathogen interaction	-	GO:0032550	GO:0006950//response to stress	gi|351724721|ref|NP_001237835.1|/0/disease resistance protein [Glycine max]
Glyma.13G071900	-1.7804	2.90E-06	-	-	-	-	-	-	-	-	4266	ko00230//Purine metabolism;ko00240//Pyrimidine metabolism;ko01100//Metabolic pathways;ko03020//RNA polymerase;ko04626//Plant-pathogen interaction	-	GO:0032550	GO:0006950//response to stress	gi|356550917|ref|XP_003543829.1|/0/PREDICTED: putative disease resistance RPP13-like protein 1 [Glycine max]
Glyma.12G216000	-	-	-	-	-	-	-	-	2.00009	0.00115	1938	ko04626//Plant-pathogen interaction	GO:0016020//membrane	GO:0032550;GO:0005515//protein binding;GO:0004702//receptor signaling protein serine/threonine kinase activity	GO:0035556//intracellular signal transduction;GO:0032147//activation of protein kinase activity;GO:0006950//response to stress	gi|359806757|ref|NP_001241300.1|/0/probable leucine-rich repeat receptor-like serine/threonine-protein kinase At5g15730-like [Glycine max]
Glyma.11G072100	-	-	-	-	-	-	-	-	1.64713	0.00409	4775	ko04626//Plant-pathogen interaction	-	GO:0032550	GO:0006950//response to stress	gi|734423019|gb|KHN41940.1|/0/Disease resistance RPP8-like protein 3 [Glycine soja]
Glyma.10G152200	-	-	-	-	-	-	-	-	3.00807	0.00746	3529	ko04626//Plant-pathogen interaction	GO:0031224//intrinsic component of membrane	GO:0046872//metal ion binding;GO:0016209//antioxidant activity;GO:0050664//oxidoreductase activity, acting on NAD(P)H, oxygen as acceptor	GO:0044707;GO:0006950//response to stress;GO:0044710	gi|947085173|gb|KRH33894.1|/0/hypothetical protein GLYMA_10G152200 [Glycine max]
Glyma.09G258200	-	-	-2.3569	0.000468	-	-	-	-	-	-	2703	ko04626//Plant-pathogen interaction	-	GO:0032550	GO:0006950//response to stress	gi|947091771|gb|KRH40436.1|/0/hypothetical protein GLYMA_09G258200 [Glycine max]

20 × 20

Glyma. 09G210 600	-	-	-	-	-	-	-	-	2.82882	0.00115	3243	ko04626//Plant-pathoge n interaction	-	GO:0032550	GO:0006950//response to stress	gi|947090963|gb|KRH39628.1|;gi|356531473|r ef|XP_003534302.1|/0;0/hypothetical protein GLYMA_09G210600 [Glycine max];PREDICTED: disease resistance protein RPM1-like [Glycine max]
Glyma. 09G204 500	-	-	-	-	-	-	-	-	1.86353	0.00076	2748	ko04075//Plant hormone signal transduction;ko04626// Plant-pathogen interaction	-	GO:0005515//protein binding	GO:0080090//regulation of primary metabolic process;GO:1901576;GO:190 1700;GO:0009889//regulation of biosynthetic process;GO:0006950//respons e to stress	gi|571478541|ref|XP_003534274.2|/0/PREDIC TED: transcription factor MYC2-like [Glycine max]
Glyma. 08G301 200	-	-	-	-	-	-	-	-	2.4358	0.00107	4462	ko00230//Purine metabolism;ko00240//P yrimidine metabolism;ko01100// Metabolic pathways;ko03020//RN A polymerase;ko04626//P lant-pathogen interaction	-	GO:0032550	GO:0006950//response to stress;GO:0007154//cell communication	gi|571473774|ref|XP_006586027.1|/0/PREDIC TED: TMV resistance protein N-like isoform X1 [Glycine max]
Glyma. 07G077 700	-	-	-	-	-	-	-	-	4.26459	7.67E-0 5	8284	ko04626//Plant-pathoge n interaction	-	GO:0032550;GO:0016462 //pyrophosphatase activity	GO:0006950//response to stress	gi|571465371|ref|XP_006583342.1|/0/PREDIC TED: uncharacterized protein LOC100797322 [Glycine max]
Glyma. 07G065 000	-	-	-	-	-	-	-	-	-1.4807	0.00335	8012	ko04626//Plant-pathoge n interaction	-	GO:0032550;GO:0016462 //pyrophosphatase activity	GO:0006950//response to stress	gi|955330113|ref|XP_006583276.2|/0/PREDIC TED: uncharacterized protein LOC100810455 isoform X1 [Glycine max]
Glyma. 07G064 100	-1.5576	1.56E-1 2	-	-	-	-	-	-	1.29957	0.00024	9014	ko04626//Plant-pathoge n interaction	-	GO:0032550;GO:0016462 //pyrophosphatase activity	GO:0006950//response to stress	gi|955330053|ref|XP_014633259.1|/0/PREDIC TED: uncharacterized protein LOC100780862 isoform X5 [Glycine max]
Glyma. 07G063 600	-1.3486	2.93E-0 9	-	-	-	-	-	-	1.92709	2.67E-0 6	6075	ko04626//Plant-pathoge n interaction	-	GO:0036094//small molecule binding;GO:0097159//orga nic cyclic compound binding;GO:1901363;GO: 0032550;GO:0016462//pyr ophosphatase activity	GO:0006950//response to stress	gi|947099532|gb|KRH48024.1|;gi|947099531|g b|KRH48023.1|/0;0/hypothetical protein GLYMA_07G063600 [Glycine max];hypothetical protein GLYMA_07G063600 [Glycine max]
Glyma. 07G062 900	-2.9084	2.66E-1 9	-	-	-	-	-	-	2.7345	0.00014	8215	ko04626//Plant-pathoge n interaction	-	GO:0032550;GO:0016462 //pyrophosphatase activity	GO:0006950//response to stress	gi|947099522|gb|KRH48014.1|;gi|571465132|r ef|XP_006583266.1|;gi|955330079|ref|XP_014 633271.1|/0;0;0/hypothetical protein GLYMA_07G062900 [Glycine max];PREDICTED: uncharacterized protein LOC100808315 isoform X1 [Glycine max];PREDICTED: uncharacterized protein LOC100808315 isoform X2 [Glycine max]
Glyma. 07G037 000	-	-	-	-	-	-	-	-	3.45257	0.00152	3965	ko04626//Plant-pathoge n interaction	-	GO:0032550	GO:0006950//response to stress;GO:0007154//cell communication	gi|571464738|ref|XP_006583154.1|;gi|947099 087|gb|KRH47579.1|/0;0/PREDICTED: disease resistance protein RML1A-like [Glycine max];hypothetical protein GLYMA_07G037000 [Glycine max]
Glyma. 06G267 300	-	-	-	-	-	-	-	-	3.01267	0.0039	3171	ko04626//Plant-pathoge n interaction	-	GO:0032550	GO:0006950//response to stress;GO:0007154//cell communication	gi|351722777|ref|NP_001235720.1|/0/resistanc e protein KR3 [Glycine max]
Glyma. 06G261 500	-	-	-	-	-	-	-	-	1.69889	0.00331	3650	ko00230//Purine metabolism;ko00240//P yrimidine metabolism;ko01100// Metabolic pathways;ko03020//RN A polymerase;ko04626//P	-	GO:0032550	GO:0006950//response to stress;GO:0008037//cell recognition;GO:0007154//cell communication	gi|571462237|ref|XP_003526160.2|/0/PREDIC TED: TMV resistance protein N-like [Glycine max]

												lant-pathogen interaction				
Glyma. 06G260 100	-	-	-	-	-	-	-	-	2.04977	6.58E-05	3893	ko00230//Purine metabolism;ko00240//Pyrimidine metabolism;ko01100//Metabolic pathways;ko03020//RNA polymerase;ko04626//Plant-pathogen interaction	-	GO:0032550	GO:0006950//response to stress;GO:0008037//cell recognition;GO:0007154//cell communication	gi|356517237|ref|XP_003527295.1|/0/PREDICTED: TMV resistance protein N [Glycine max]
Glyma. 06G259 800	-	-	-	-	-2.2682	0.00131	-	-	3.24167	0.00055	4077	ko00230//Purine metabolism;ko00240//Pyrimidine metabolism;ko01100//Metabolic pathways;ko03020//RNA polymerase;ko04626//Plant-pathogen interaction	-	GO:0032550	GO:0006950//response to stress;GO:0008037//cell recognition;GO:0007154//cell communication;GO:0050896//response to stimulus	gi|947107122|gb|KRH55505.1|;gi|947107121|gb|KRH55504.1|/0;0/hypothetical protein GLYMA_06G259800 [Glycine max];hypothetical protein GLYMA_06G259800 [Glycine max]
Glyma. 06G259 400	-	-	-	-	-	-	-	-	3.81531	1.43E-06	3959	ko04626//Plant-pathogen interaction	-	GO:0032550	GO:0006950//response to stress;GO:0008037//cell recognition;GO:0007154//cell communication;GO:0050896//response to stimulus	gi|947107112|gb|KRH55495.1|;gi|947107117|gb|KRH55500.1|;gi|947107116|gb|KRH55499.1|;gi|947107115|gb|KRH55498.1|;gi|947107113|gb|KRH55496.1|;gi|947107114|gb|KRH55497.1|/0;0;0;0;0;0/hypothetical protein GLYMA_06G259400 [Glycine max];hypothetical protein GLYMA_06G259400 [Glycine max];hypothetical protein GLYMA_06G259400 [Glycine max];hypothetical protein GLYMA_06G259400 [Glycine max];hypothetical protein GLYMA_06G259400 [Glycine max];hypothetical protein GLYMA_06G259400 [Glycine max]
Glyma. 06G256 000	-	-	-	-	-	-	-	-	3.37412	1.43E-05	4316	ko00230//Purine metabolism;ko00240//Pyrimidine metabolism;ko01100//Metabolic pathways;ko03020//RNA polymerase;ko04626//Plant-pathogen interaction	-	GO:0032550	GO:0006950//response to stress;GO:0008037//cell recognition;GO:0007154//cell communication;GO:0050896//response to stimulus	gi|947107062|gb|KRH55445.1|;gi|947107064|gb|KRH55447.1|;gi|955325788|ref|XP_014632205.1|;gi|947107067|gb|KRH55450.1|;gi|947107063|gb|KRH55446.1|;gi|955325790|ref|XP_014632206.1|/0;0;0;0;0;0/hypothetical protein GLYMA_06G256000 [Glycine max];hypothetical protein GLYMA_06G256000 [Glycine max];PREDICTED: disease resistance-like protein CSA1 isoform X1 [Glycine max];hypothetical protein GLYMA_06G256000 [Glycine max];hypothetical protein GLYMA_06G256000 [Glycine max];PREDICTED: disease resistance-like protein CSA1 isoform X2 [Glycine max]
Glyma. 05G082 500	-	-	-	-	-2.2138	0.00091	-	-	2.48655	0.00114	3736	ko00230//Purine metabolism;ko00240//Pyrimidine metabolism;ko01100//Metabolic pathways;ko03020//RNA polymerase;ko04626//Plant-pathogen	-	GO:0032550	GO:0006950//response to stress	gi|571454745|ref|XP_006579882.1|/0/PREDICTED: probable disease resistance protein At5g66900 [Glycine max]

												interaction				
Glyma.05G082400	-	-	-	-	-1.2847	9.67E-05	-	-	-	-	2976	ko00230//Purine metabolism;ko00240//Pyrimidine metabolism;ko01100//Metabolic pathways;ko03020//RNA polymerase;ko04626//Plant-pathogen interaction	-	GO:0032550	GO:0006950//response to stress	gi|571454747|ref|XP_006579883.1|/0/PREDICTED: probable disease resistance protein At5g66900 [Glycine max]
Glyma.05G082200	-	-	-	-	-1.6823	0.00043	-	-	2.29377	0.00314	3498	ko04626//Plant-pathogen interaction	-	GO:0032550	GO:0006950//response to stress	gi|356511730|ref|XP_003524576.1|/0/PREDICTED: probable disease resistance protein At5g66900 isoform X1 [Glycine max]
Glyma.04G134700	-1.6685	2.87E-11	-	-	-	-	-	-	2.32564	0.00019	2957	ko04626//Plant-pathogen interaction	GO:0043231//intracellular membrane-bounded organelle	GO:0046914//transition metal ion binding;GO:0016301//kinase activity;GO:0032550;GO:0005515//protein binding;GO:0019787//ubiquitin-like protein transferase activity;GO:0050136//NADH dehydrogenase (quinone) activity	GO:0040007//growth;GO:1902582;GO:0032446//protein modification by small protein conjugation;GO:0006950//response to stress;GO:0009738//abscisic acid-activated signaling pathway;GO:0046903//secretion;GO:0022904//respiratory electron transport chain	gi|947114519|gb|KRH62821.1|/0/hypothetical protein GLYMA_04G134700 [Glycine max]
Glyma.03G236300	-	-	-	-	-	-	-	-	3.67642	0.00059	3154	ko04626//Plant-pathogen interaction	GO:0031224//intrinsic component of membrane	GO:0046872//metal ion binding;GO:0016209//antioxidant activity;GO:0050664//oxidoreductase activity, acting on NAD(P)H, oxygen as acceptor	GO:0044707;GO:0006950//response to stress;GO:0044710	gi|356505839|ref|XP_003521697.1|;gi|947120277|gb|KRH68526.1|/0;0/PREDICTED: respiratory burst oxidase homolog protein B-like [Glycine max];hypothetical protein GLYMA_03G236300 [Glycine max]
Glyma.03G087500	4.67502	2.13E-13	3.878	2.10E-08	1.88006	4.47E-05	-	-	2.60263	2.70E-06	3462	ko00230//Purine metabolism;ko00240//Pyrimidine metabolism;ko01100//Metabolic pathways;ko03020//RNA polymerase;ko04626//Plant-pathogen interaction	-	GO:0032550	GO:0050896//response to stimulus;GO:0006950//response to stress;GO:0007154//cell communication	gi|947117919|gb|KRH66168.1|;gi|947117917|gb|KRH66166.1|;gi|947117918|gb|KRH66167.1|/0;0;0/hypothetical protein GLYMA_03G087500 [Glycine max];hypothetical protein GLYMA_03G087500 [Glycine max];hypothetical protein GLYMA_03G087500 [Glycine max]
Glyma.03G075200	-	-	-	-	-	-	-	-	1.67137	0.00106	4750	ko00230//Purine metabolism;ko00240//Pyrimidine metabolism;ko01100//Metabolic pathways;ko03020//RNA polymerase;ko04626//Plant-pathogen interaction	-	GO:0032550	GO:0006950//response to stress	gi|351723333|ref|NP_001237787.1|/0/NB-LRR type disease resistance protein [Glycine max]
Glyma.02G041800	-1.0331	0.005334	-	-	-	-	-	-	-	-	4056	ko04626//Plant-pathogen interaction	-	GO:0032550	GO:0006950//response to stress;GO:0007154//cell communication	gi|571438700|ref|XP_006574650.1|;gi|571438704|ref|XP_006574651.1|/0;0/PREDICTED: TMV resistance protein N-like isoform X1 [Glycine max];PREDICTED: TMV resistance protein N-like isoform X2 [Glycine max]
Glyma.01G244500	-	-	-	-	-	-	-	-	-1.6733	0.00048	1475	ko04626//Plant-pathogen interaction	GO:0005911//cell-cell	-	GO:0006950//response to stress	gi|356497514|ref|XP_003517605.1|/7.23568e-107/PREDICTED: protein SRC2 homolog [Glycine max]

													junction;GO:0044444			
Glyma.01G183400	-	-	-	-	-	-	-	-	1.21537	0.00257	3041	ko00230//Purine metabolism;ko00240//Pyrimidine metabolism;ko01100//Metabolic pathways;ko03020//RNA polymerase;ko04626//Plant-pathogen interaction	-	GO:0032550	GO:0006950//response to stress	gi|356495456|ref|XP_003516593.1|/0/PREDICTED: probable disease resistance protein At5g66900 [Glycine max]
Glyma.01G039000	-1.5056	0.000133	-	-	-	-	-	-	-	-	4612	ko04626//Plant-pathogen interaction	-	GO:0016462//pyrophosphatase activity;GO:0032550	GO:0006950//response to stress;GO:0007154//cell communication	gi|571434029|ref|XP_006573080.1|;gi|947126871|gb|KRH74725.1|/0;0/PREDICTED: TMV resistance protein N-like [Glycine max];hypothetical protein GLYMA_01G039000 [Glycine max]
Glyma.01G010500	-	-	-	-	-3.5148	1.77E-05	-	-	-	-	3423	ko04626//Plant-pathogen interaction	-	GO:0032550	GO:0006950//response to stress	gi|351721361|ref|NP_001235671.1|/0/NBS-LRR type disease resistance protein [Glycine max]
Glyma.01G013500	-1.6818	0.000117	-	-	-	-	-	-	-	-	2710	ko04626//Plant-pathogen interaction	-	GO:0004672//protein kinase activity	GO:0044237//cellular metabolic process	gi|356497629|ref|XP_003517662.1|/0/PREDICTED: putative receptor-like protein kinase At4g00960 [Glycine max]
Glyma.01G019700	-	-	-	-	-	-	-1.2556	0.00045	-	-	1586	ko04075//Plant hormone signal transduction;ko04626//Plant-pathogen interaction	-	GO:0005515//protein binding	-	gi|918463553|gb|ALA09121.1|;gi|947126586|gb|KRH74440.1|;gi|571433718|ref|XP_006572984.1|/0;0;0/bHLH transcription factor, partial [Glycine max] ;hypothetical protein GLYMA_01G019700 [Glycine max];PREDICTED: transcription factor bHLH93-like isoform X2 [Glycine max]
Glyma.01G169500	-	-	-	-	-	-	1.34073	0.00063	-	-	1949	ko04626//Plant-pathogen interaction	-	-	-	gi|571435744|ref|XP_003516553.2|/0/PREDICTED: leucine-rich repeat receptor-like protein kinase PXL2 [Glycine max]
Glyma.02G030500	1.34889	0.004014	-	-	-	-	-	-	-	-	1808	ko00230//Purine metabolism;ko00240//Pyrimidine metabolism;ko01100//Metabolic pathways;ko03020//RNA polymerase;ko04626//Plant-pathogen interaction	-	-	-	gi|734394680|gb|KHN28666.1|/0/Disease resistance RPP13-like protein 4 [Glycine soja]
Glyma.02G060800	-	-	-	-	-	-	3.3442	2.71E-06	-	-	519	ko04075//Plant hormone signal transduction;ko04626//Plant-pathogen interaction	-	-	-	gi|571438947|ref|XP_003519913.2|/1.87092e-112/PREDICTED: STS14 protein-like [Glycine max]
Glyma.02G122000	-1.6118	0.001313	-	-	-	-	-	-	-	-	2634	ko04626//Plant-pathogen interaction	GO:0071944//cell periphery;GO:0044464	GO:0032550;GO:0004672//protein kinase activity	GO:0006464//cellular protein modification process;GO:0044767;GO:0006796//phosphate-containing compound metabolic process	gi|571439797|ref|XP_003520131.2|;gi|947122773|gb|KRH70979.1|/0;0/PREDICTED: receptor-like protein kinase FERONIA [Glycine max];hypothetical protein GLYMA_02G122000 [Glycine max]
Glyma.02G133000	-	-	-	-	-	-	-1.1072	0.00022	-	-	1274	ko04626//Plant-pathogen interaction	-	GO:0046872//metal ion binding;GO:0016491//oxidoreductase activity	-	gi|351721626|ref|NP_001235680.1|/2.1097e-124/uncharacterized protein LOC100499763 [Glycine max]
Glyma.02G186900	-3.6771	5.81E-08	-	-	-3.7279	6.25E-06	-	-	-	-	780	ko04626//Plant-pathogen interaction	-	-	-	gi|947123831|gb|KRH72037.1|/1.48469e-120/hypothetical protein GLYMA_02G186900 [Glycine max]

20 × 20

Glyma.02G189800	-7.1847	7.47E-18	-	-	-8	1.60E-06	-	-	-	-	1077	ko04626//Plant-pathogen interaction	-	GO:0004672//protein kinase activity	GO:0044237//cellular metabolic process	gi|947123865|gb|KRH72071.1|/0/hypothetical protein GLYMA_02G189800 [Glycine max]
Glyma.02G228300	-	-	-	-	-3.0422	0.0009	-	-	-	-	1506	ko04626//Plant-pathogen interaction	-	GO:0016301//kinase activity	GO:0044238//primary metabolic process;GO:0044260;GO:0016310//phosphorylation	gi|734432078|gb|KHN46141.1|/0/Mitogen-activated protein kinase kinase kinase 2 [Glycine soja]
Glyma.02G263900	-1.0188	0.002977	-	-	-	-	-	-	-	-	2483	ko04626//Plant-pathogen interaction	GO:0031224//intrinsic component of membrane	GO:0016301//kinase activity;GO:0032550	GO:0006796//phosphate-containing compound metabolic process;GO:0006464//cellular protein modification process	gi|356499209|ref|XP_003518434.1|/0/PREDICTED: probable inactive receptor-like protein kinase At3g56050 [Glycine max]
Glyma.03G037300	-1.7241	8.08E-05	-	-	-	-	-	-	-	-	4743	ko00230//Purine metabolism;ko00240//Pyrimidine metabolism;ko01100//Metabolic pathways;ko03020//RNA polymerase;ko04626//Plant-pathogen interaction	-	-	-	gi|571444245|ref|XP_006576452.1|/0/PREDICTED: putative disease resistance protein At3g14460 isoform X1 [Glycine max]
Glyma.03G087600	-1.0887	0.00035	-	-	-	-	-	-	-	-	3387	ko04626//Plant-pathogen interaction	-	-	-	gi|947117921|gb|KRH66170.1|;gi|947117920|gb|KRH66169.1|/0;0/hypothetical protein GLYMA_03G087600, partial [Glycine max];hypothetical protein GLYMA_03G087600, partial [Glycine max]
Glyma.03G165800	-3.1109	3.90E-06	-	-	-	-	-	-	-	-	4009	ko04626//Plant-pathogen interaction	GO:0031224//intrinsic component of membrane	GO:0004672//protein kinase activity;GO:0032550	GO:0006468//protein phosphorylation	gi|571445952|ref|XP_003520610.2|/0/PREDICTED: probable LRR receptor-like serine/threonine-protein kinase At4g08850 [Glycine max]
Glyma.03G225000	-2.187	0.000876	-	-	-	-	-	-	-	-	2036	ko04075//Plant hormone signal transduction;ko04712//Circadian rhythm - plant;ko04626//Plant-pathogen interaction	-	-	-	gi|571446784|ref|XP_003520776.2|/0/PREDICTED: transcription factor PIF3-like [Glycine max]
Glyma.03G232500	-	-	-	-	-2.101	0.00129	-	-	-	-	2258	ko04070//Phosphatidylinositol signaling system;ko04626//Plant-pathogen interaction	-	-	-	gi|356505783|ref|XP_003521669.1|;gi|571446892|ref|XP_006577216.1|/0;0/PREDICTED: calmodulin-binding protein 60 B isoform X1 [Glycine max];PREDICTED: calmodulin-binding protein 60 B isoform X2 [Glycine max]
Glyma.04G054200	4.36501	2.66E-13	-	-	-	-	-	-	-	-	987	ko04626//Plant-pathogen interaction	-	GO:0001071//nucleic acid binding transcription factor activity;GO:0003677//DNA binding	GO:0050896//response to stimulus;GO:0050794//regulation of cellular process;GO:0006351//transcription, DNA-templated	gi|356507032|ref|XP_003522275.1|/5.63141e-118/PREDICTED: probable WRKY transcription factor 50 [Glycine max]
Glyma.04G090100	1.39161	0.001806	-	-	-	-	-	-	-	-	1549	ko04075//Plant hormone signal transduction;ko04626//Plant-pathogen interaction	-	GO:0005515//protein binding	-	gi|947113857|gb|KRH62159.1|/0/hypothetical protein GLYMA_04G090100 [Glycine max]
Glyma.04G136200	-	-	-	-	-5.5982	2.78E-05	-	-	-	-	919	ko04626//Plant-pathogen interaction	-	GO:0046872//metal ion binding	-	gi|947114537|gb|KRH62839.1|/1.15828e-96/hypothetical protein GLYMA_04G136200 [Glycine max]
Glyma.04G188700	-	-	-	-	-	-	1.43891	1.01E-06	-	-	1936	ko04626//Plant-pathogen interaction	GO:0031981//nuclear lumen;GO	GO:0032550;GO:0017111//nucleoside-triphosphatase activity;GO:0008135//tran	GO:0006412//translation;GO:0009154//purine ribonucleotide catabolic process	gi|356508726|ref|XP_003523105.1|/0/PREDICTED: elongation factor Tu, chloroplastic-like [Glycine max]

20 × 20

													:0005576//extracellular region;GO:0009534//chloroplast thylakoid;GO:0009526//plastid envelope	slation factor activity, nucleic acid binding		
Glyma.05G021100	-1.1036	3.09E-05	-	-	-	-	-	-	-	-	3526	ko04626//Plant-pathogen interaction	GO:0031224//intrinsic component of membrane	GO:0046872//metal ion binding;GO:0016651//oxidoreductase activity, acting on NAD(P)H;GO:0016209//antioxidant activity	GO:0044710	gi|571453402|ref|XP_006579505.1|/0/PREDICTED: respiratory burst oxidase homolog protein A-like isoform X1 [Glycine max]
Glyma.05G085200	-	-	-	-	-2.5529	7.52E-06	-	-	-	-	3109	ko04626//Plant-pathogen interaction	-	GO:0046872//metal ion binding	-	gi|734386654|gb|KHN25017.1|/2.19482e-121/Calmodulin-like protein 1 [Glycine soja]
Glyma.06G147100	8.34201	1.33E-15	-	-	-	-	-	-	-	-	1180	ko04626//Plant-pathogen interaction	-	-	GO:0009987//cellular process	gi|356516303|ref|XP_003526835.1|/1.14509e-118/PREDICTED: probable WRKY transcription factor 51 [Glycine max]
Glyma.06G147500	1.2473	6.42E-05	-	-	-	-	-	-	-	-	1417	ko04626//Plant-pathogen interaction	-	-	-	gi|571460585|ref|XP_003526838.2|;gi|955324384|ref|XP_014631935.1|;gi|571460587|ref|XP_006581742.1|/2.30138e-139;1.08246e-113;8.10111e-123/PREDICTED: probable WRKY transcription factor 13 isoform X1 [Glycine max];PREDICTED: probable WRKY transcription factor 13 isoform X3 [Glycine max];PREDICTED: probable WRKY transcription factor 13 isoform X2 [Glycine max]
Glyma.06G155500	-	-	-	-	-	-	1.24487	0.00087	-	-	2762	ko04626//Plant-pathogen interaction	-	GO:0004672//protein kinase activity;GO:0032550	GO:0050896//response to stimulus;GO:0006464//cellular protein modification process	gi|356516377|ref|XP_003526871.1|/0/PREDICTED: probable receptor-like serine/threonine-protein kinase At5g57670 [Glycine max]
Glyma.06G176900	-	-	-	-	-	-	1.30953	1.99E-05	-	-	2054	ko04626//Plant-pathogen interaction	GO:0031981//nuclear lumen;GO:0005576//extracellular region;GO:0009534//chloroplast thylakoid;GO:0009526//plastid envelope	GO:0032550;GO:0017111//nucleoside-triphosphatase activity;GO:0008135//translation factor activity, nucleic acid binding	GO:0006412//translation;GO:0009154//purine ribonucleotide catabolic process	gi|356516623|ref|XP_003526993.1|/0/PREDICTED: elongation factor Tu, chloroplastic [Glycine max]
Glyma.06G256600	-2.2655	0.000243	-	-	-	-	-	-	-	-	2728	ko04626//Plant-pathogen interaction	-	-	-	gi|947107079|gb|KRH55462.1|;gi|947107078|gb|KRH55461.1|/0;0/hypothetical protein GLYMA_06G256600 [Glycine max];hypothetical protein GLYMA_06G256600 [Glycine max]
Glyma.07G003200	-	-	-	-	-	-	1.33451	0.00048	-	-	2281	ko04626//Plant-pathogen interaction	GO:0043231//intracellular membrane-bounded organelle	GO:0032550;GO:0004672//protein kinase activity	GO:0045682//regulation of epidermis development;GO:0045087//innate immune response;GO:0006464//cellular protein modification process;GO:0006796//phosphate-containing compound	gi|356521707|ref|XP_003529493.1|/0/PREDICTED: mitogen-activated protein kinase kinase 4 [Glycine max]

20 × 20

															metabolic process;GO:0090567	
Glyma.07G101100	-	-	-	-	-	-	1.64321	6.92E-05	-	-	911	ko04626//Plant-pathogen interaction	GO:0016020//membrane;GO:0043231//intracellular membrane-bounded organelle	GO:0046872//metal ion binding	-	gi|356520641|ref|XP_003528969.1|/3.56334e-128/PREDICTED: probable calcium-binding protein CML18 [Glycine max]
Glyma.07G143500	2.64784	0.000726	-	-	-	-	-	-	-	-	3222	ko04626//Plant-pathogen interaction	-	-	-	gi|947100766|gb|KRH49258.1|/0/hypothetical protein GLYMA_07G143500 [Glycine max]
Glyma.07G148500	-1.0211	0.00404	-	-	-	-	-	-	-	-	1859	ko04626//Plant-pathogen interaction	-	GO:0004672//protein kinase activity;GO:0032550	GO:0006464//cellular protein modification process	gi|571466375|ref|XP_006583644.1|/0/PREDICTED: putative serine/threonine-protein kinase isoform X1 [Glycine max]
Glyma.07G188800	-1.1234	0.004431	-	-	-	-	-	-	-	-	3106	ko04626//Plant-pathogen interaction	-	GO:0004672//protein kinase activity;GO:0032550	GO:0006796//phosphate-containing compound metabolic process;GO:0006464//cellular protein modification process;GO:0008037//cell recognition	gi|356519528|ref|XP_003528424.1|;gi|947101435|gb|KRH49927.1|/0;0/PREDICTED: G-type lectin S-receptor-like serine/threonine-protein kinase B120 isoform X1 [Glycine max];hypothetical protein GLYMA_07G188800 [Glycine max]
Glyma.07G229500	3.72869	2.51E-09	-	-	-	-	-	-	-	-	671	ko04626//Plant-pathogen interaction	-	-	-	gi|571467514|ref|XP_006583962.1|/3.62461e-119/PREDICTED: probable calcium-binding protein CML45 [Glycine max]
Glyma.08G089100	-1.1854	0.004227	-	-	-	-	-	-	-	-	2077	ko04075//Plant hormone signal transduction;ko04626//Plant-pathogen interaction	-	GO:0005515//protein binding	-	gi|734395411|gb|KHN28921.1|/0/Transcription factor bHLH91 [Glycine soja]
Glyma.08G140900	-1.259	1.42E-06	-	-	-	-	-	-	-	-	2896	ko04626//Plant-pathogen interaction	GO:0009526//plastid envelope	GO:0016740//transferase activity	-	gi|356525527|ref|XP_003531376.1|/0/PREDICTED: uncharacterized protein sll1770-like [Glycine max]
Glyma.08G300900	-	-	4.05723	7.94E-12	-	-	-	-	-	-	804	ko04626//Plant-pathogen interaction	GO:0043231//intracellular membrane-bounded organelle	-	-	gi|947097334|gb|KRH45919.1|/0/hypothetical protein GLYMA_08G300900 [Glycine max]
Glyma.08G317400	-1.281	0.004056	-	-	-	-	-	-	-	-	4364	ko04626//Plant-pathogen interaction	-	-	-	gi|955337909|ref|XP_014634887.1|/0/PREDICTED: disease resistance protein RPM1-like [Glycine max]
Glyma.09G048300	-1.2898	9.83E-12	-	-	-	-	-	-	-	-	4435	ko04626//Plant-pathogen interaction	GO:0016020//membrane	GO:0036094//small molecule binding;GO:0004672//protein kinase activity;GO:1901363;GO:0097159//organic cyclic compound binding	GO:0006796//phosphate-containing compound metabolic process	gi|571476366|ref|XP_003533521.2|/0/PREDICTED: LRR receptor-like serine/threonine-protein kinase GSO1 [Glycine max]
Glyma.09G060200	-1.6886	0.000164	-	-	-2.2121	9.07E-06	-	-	-	-	1321	ko04075//Plant hormone signal transduction;ko04626//Plant-pathogen interaction	-	GO:0005515//protein binding	-	gi|358248852|ref|NP_001239951.1|/4.42554e-170/transcription factor bHLH35-like [Glycine max]
Glyma.09G073200	-2.895	8.92E-08	-	-	-	-	-	-	-	-	3397	ko04626//Plant-pathogen interaction	GO:0031224//intrinsic component of membrane	GO:0046872//metal ion binding;GO:0016651//oxidoreductase activity, acting on NAD(P)H;GO:0016209//antioxidant activity	GO:0044710	gi|947088885|gb|KRH37550.1|;gi|571476753|ref|XP_006587062.1|/0;0/hypothetical protein GLYMA_09G073200 [Glycine max];PREDICTED: respiratory burst oxidase homolog protein E-like [Glycine max]

20 × 20

Glyma.09G131500	-	-	-	-	-2.5086	6.29E-08	-	-	-	-	2458	ko04141//Protein processing in endoplasmic reticulum;ko04626//Plant-pathogen interaction	-	GO:0005515//protein binding;GO:0032550	GO:0050896//response to stimulus;GO:0044267//cellular protein metabolic process	gi|959092738|ref|NP_001304616.1|/0/heat shock protein 83 [Glycine max]
Glyma.09G173800	-1.1171	0.000813	-	-	-	-	-	-	-	-	1965	ko04075//Plant hormone signal transduction;ko04626//Plant-pathogen interaction	GO:0005911//cell-cell junction;GO:0031224//intrinsic component of membrane;GO:0044437	GO:0016301//kinase activity;GO:0032550	GO:0006796//phosphate-containing compound metabolic process;GO:0006464//cellular protein modification process	gi|947090375|gb|KRH39040.1|/0/hypothetical protein GLYMA_09G173800 [Glycine max]
Glyma.09G208900	-1.4835	3.54E-05	-	-	-	-	-	-	-	-	2205	ko00230//Purine metabolism;ko00240//Pyrimidine metabolism;ko01100//Metabolic pathways;ko03020//RNA polymerase;ko04626//Plant-pathogen interaction	-	-	-	gi|947090939|gb|KRH39604.1|/0/hypothetical protein GLYMA_09G208900 [Glycine max]
Glyma.09G216400	-	-	8.34377	3.44E-12	-	-	-	-	-	-	4263	ko04626//Plant-pathogen interaction	GO:0031224//intrinsic component of membrane	GO:0004672//protein kinase activity;GO:0032550;GO:0016301//kinase activity	GO:0006796//phosphate-containing compound metabolic process;GO:0006464//cellular protein modification process	gi|947091060|gb|KRH39725.1|;gi|571478728|ref|XP_006587651.1|/0;0/hypothetical protein GLYMA_09G216400 [Glycine max];PREDICTED: probable LRR receptor-like serine/threonine-protein kinase At3g47570 isoform X2 [Glycine max]
Glyma.09G270400	-1.2044	0.001246	-	-	-	-	-	-	-	-	1365	ko04075//Plant hormone signal transduction;ko04626//Plant-pathogen interaction	-	GO:0016772//transferase activity, transferring phosphorus-containing groups	-	gi|955342880|ref|XP_014617928.1|/7.75255e-114/PREDICTED: cysteine-rich receptor-like protein kinase 24 [Glycine max]
Glyma.10G026000	-1.035	2.29E-06	-	-	-	-	-	-	-	-	2818	ko04075//Plant hormone signal transduction;ko04626//Plant-pathogen interaction	-	-	GO:0044763;GO:0031326;GO:0006351//transcription, DNA-templated	gi|571481338|ref|XP_006588626.1|/0/PREDICTED: basic helix-loop-helix protein A-like [Glycine max]
Glyma.10G109200	-1.9431	2.71E-07	-	-	-	-	-	-	-	-	2092	ko04626//Plant-pathogen interaction	-	GO:0036094//small molecule binding;GO:0004672//protein kinase activity;GO:1901363;GO:0097159//organic cyclic compound binding;GO:0016772//transferase activity, transferring phosphorus-containing groups;GO:0032550	GO:0006464//cellular protein modification process	gi|571482431|ref|XP_006588951.1|;gi|947084513|gb|KRH33234.1|;gi|571482433|ref|XP_006588952.1|;gi|955345591|ref|XP_014618594.1|;gi|571482429|ref|XP_006588950.1|/0;0;0;0;0/PREDICTED: cysteine-rich receptor-like protein kinase 19 isoform X2 [Glycine max];hypothetical protein GLYMA_10G109200 [Glycine max];PREDICTED: cysteine-rich receptor-like protein kinase 25 isoform X3 [Glycine max];PREDICTED: cysteine-rich receptor-like protein kinase 24 isoform X4 [Glycine max];PREDICTED: cysteine-rich receptor-like protein kinase 25 isoform X1 [Glycine max]
Glyma.10G186000	-1.1519	1.60E-06	-	-	-	-	-	-	-	-	1228	ko04626//Plant-pathogen interaction	-	GO:0004672//protein kinase activity;GO:0032550	GO:0006796//phosphate-containing compound metabolic process;GO:0006464//cellular protein modification process	gi|351723241|ref|NP_001237016.1|/0/phosphoenolpyruvate-carboxylase kinase [Glycine max]
Glyma.10G195700	1.37747	0.000323	2.14603	8.70E-05	-	-	-	-	-	-	3252	ko04626//Plant-pathogen interaction	GO:0031224//intrinsic component	GO:0004672//protein kinase activity;GO:0032550;GO:0016772//transferase	GO:0006796//phosphate-containing compound metabolic process;GO:0006464//cellular protein modification process	gi|356533862|ref|XP_003535477.1|;gi|734415812|gb|KHN37899.1|/0;0/PREDICTED: receptor-like protein kinase [Glycine max];Receptor-like protein kinase [Glycine

													of membrane	activity, transferring phosphorus-containing groups		soja]
Glyma. 10G252 700	1.93406	0.000344	-	-	-	-	-	-	-	-	2254	ko04075//Plant hormone signal transduction;ko04626// Plant-pathogen interaction	-	GO:0004672//protein kinase activity;GO:0032550	GO:0006796//phosphate-containing compound metabolic process;GO:0006464//cellular protein modification process	gi|947086881|gb|KRH35602.1|;gi|571480841|ref|XP_006588454.1|;gi|947086882|gb|KRH35603.1|/0;0;0/hypothetical protein GLYMA_10G252700 [Glycine max];PREDICTED: cysteine-rich receptor-like protein kinase 10-like isoform X1 [Glycine max];hypothetical protein GLYMA_10G252700 [Glycine max]
Glyma. 10G253 200	3.2775	5.20E-09	-	-	-	-	-	-	-	-	2615	ko04075//Plant hormone signal transduction;ko04626// Plant-pathogen interaction	-	-	-	gi|356534242|ref|XP_003535666.1|/0/PREDICTED: cysteine-rich receptor-like protein kinase 26 [Glycine max]
Glyma. 10G263 200	-	-	-	-	1.04849	0.00087	1.62627	2.85E-07	-	-	1670	ko04626//Plant-pathogen interaction	-	-	-	gi|356536184|ref|XP_003536619.1|/0/PREDICTED: probable LRR receptor-like serine/threonine-protein kinase At4g20940 [Glycine max]
Glyma. 11G045 700	-	-	-	-	-	-	1.34784	3.23E-05	-	-	1666	ko04075//Plant hormone signal transduction;ko04626// Plant-pathogen interaction	-	-	-	gi|359806711|ref|NP_001241548.1|/1.26439e-162/LRR receptor-like serine/threonine-protein kinase GSO2-like precursor [Glycine max]
Glyma. 11G056 500	1.43199	0.002725	-	-	-	-	-	-	-	-	1480	ko04626//Plant-pathogen interaction	-	GO:0046872//metal ion binding	-	gi|734423154|gb|KHN42075.1|/2.89802e-135/ Calmodulin-like protein 1 [Glycine soja]
Glyma. 11G073 800	-	-	-	-	-	-	1.5327	1.36E-06	-	-	1986	ko04626//Plant-pathogen interaction	-	-	-	gi|359807401|ref|NP_001241130.1|/0/LRR receptor-like serine/threonine-protein kinase GSO1-like precursor [Glycine max]
Glyma. 12G143 000	-2.3424	0.000585	-	-	-	-	-	-	-	-	2742	ko04626//Plant-pathogen interaction	-	GO:0016301//kinase activity;GO:0004672//protein kinase activity;GO:0032550	GO:0009987//cellular process;GO:0006464//cellular protein modification process	gi|734414229|gb|KHN37217.1|;gi|947077133|gb|KRH25973.1|;gi|571493480|ref|XP_006592565.1|;gi|571493478|ref|XP_006592564.1|/0;0;0;0/G-type lectin S-receptor-like serine/threonine-protein kinase [Glycine soja] ;hypothetical protein GLYMA_12G143000 [Glycine max];PREDICTED: G-type lectin S-receptor-like serine/threonine-protein kinase SD1-1 isoform X3 [Glycine max];PREDICTED: G-type lectin S-receptor-like serine/threonine-protein kinase At4g27290 isoform X2 [Glycine max]
Glyma. 12G172 700	1.32281	0.003308	-	-	-	-	-	-	-	-	2039	ko04626//Plant-pathogen interaction	-	GO:0016301//kinase activity;GO:0032550	GO:0006464//cellular protein modification process	gi|571493920|ref|XP_006592693.1|/0/PREDICTED: serine/threonine-protein kinase CDL1-like [Glycine max]
Glyma. 12G175 300	-1.6315	1.28E-10	-	-	-	-	-	-	-	-	2971	ko04626//Plant-pathogen interaction	GO:0044440	GO:0032550;GO:0004672//protein kinase activity;GO:0019899//enzyme binding	GO:0010087//phloem or xylem histogenesis;GO:0006464//cellular protein modification process;GO:0006796//phosphate-containing compound metabolic process	gi|955351956|ref|XP_014619794.1|;gi|947077612|gb|KRH26452.1|;gi|351725463|ref|NP_001237605.1|;gi|955351954|ref|XP_014619793.1|/0;0;0;0/PREDICTED: protein kinase family protein isoform X3 [Glycine max];hypothetical protein GLYMA_12G175300 [Glycine max];protein kinase family protein precursor [Glycine max];PREDICTED: protein kinase family protein isoform X2 [Glycine max]
Glyma. 12G198 600	-1.2985	0.001953	-	-	-	-	-	-	-	-	2860	ko04075//Plant hormone signal transduction;ko04626// Plant-pathogen interaction	-	GO:0004672//protein kinase activity;GO:0032550	GO:0006796//phosphate-containing compound metabolic process;GO:0006464//cellular protein modification process;GO:0008037//cell recognition	gi|955355212|ref|XP_014620518.1|;gi|571494369|ref|XP_006592830.1|/0;0/PREDICTED: G-type lectin S-receptor-like serine/threonine-protein kinase At2g19130 isoform X1 [Glycine max];PREDICTED: G-type lectin S-receptor-like

																serine/threonine-protein kinase At2g19130 isoform X2 [Glycine max]
Glyma.13G053700	4.07098	2.44E-16	2.81783	8.92E-06	-	-	-	-	-	-	3047	ko04626//Plant-pathogen interaction	-	GO:0004672//protein kinase activity;GO:0032550	GO:0006796//phosphate-containing compound metabolic process;GO:0006464//cellular protein modification process	gi|947069467|gb|KRH18358.1|/0/hypothetical protein GLYMA_13G053700 [Glycine max]
Glyma.13G054300	-2.8374	1.24E-07	-2.5232	0.000178	-	-	-	-	-	-	2535	ko04626//Plant-pathogen interaction	-	GO:0004672//protein kinase activity;GO:0032550	GO:0006796//phosphate-containing compound metabolic process;GO:0006464//cellular protein modification process	gi|351727166|ref|NP_001238431.1|/0/FERONIA receptor-like kinase precursor [Glycine max]
Glyma.13G080700	-1.5216	0.000874	-	-	-	-	-	-	-	-	1185	ko04626//Plant-pathogen interaction	GO:0031225//anchored component of membrane	GO:0005539//glycosaminoglycan binding	GO:0044036//cell wall macromolecule metabolic process;GO:0002376//immune system process	gi|947069872|gb|KRH18763.1|;gi|947069871|gb|KRH18762.1|/0;0/hypothetical protein GLYMA_13G080700 [Glycine max];hypothetical protein GLYMA_13G080700 [Glycine max]
Glyma.13G184800	-1.6136	2.18E-06	-	-	-1.7479	0.00035	-1.9949	1.42E-05	-	-	4261	ko00230//Purine metabolism;ko00240//Pyrimidine metabolism;ko01100//Metabolic pathways;ko03020//RNA polymerase;ko04626//Plant-pathogen interaction	-	-	-	gi|955358201|ref|XP_014621131.1|/0/PREDICTED: putative disease resistance protein At3g14460 isoform X1 [Glycine max]
Glyma.13G190000	-2.2244	1.57E-07	-	-	-1.9935	0.00074	-2.0565	0.00053	-	-	3735	ko00230//Purine metabolism;ko00240//Pyrimidine metabolism;ko01100//Metabolic pathways;ko03020//RNA polymerase;ko04626//Plant-pathogen interaction	-	-	-	gi|947071732|gb|KRH20623.1|/0/hypothetical protein GLYMA_13G190000 [Glycine max]
Glyma.13G195600	-1.4096	0.003179	-	-	-	-	-	-	-	-	3826	ko00230//Purine metabolism;ko00240//Pyrimidine metabolism;ko01100//Metabolic pathways;ko03020//RNA polymerase;ko04626//Plant-pathogen interaction	-	-	-	gi|356546346|ref|XP_003541587.1|/0/PREDICTED: putative disease resistance RPP13-like protein 1 isoform X1 [Glycine max]
Glyma.13G365900	-	-	-	-	1.39809	0.00012	1.54189	0.00031	-	-	1531	ko04626//Plant-pathogen interaction	-	GO:0016772//transferase activity, transferring phosphorus-containing groups	GO:0006796//phosphate-containing compound metabolic process;GO:0051301//cell division;GO:0001101//response to acid chemical	gi|947074694|gb|KRH23585.1|/0/hypothetical protein GLYMA_13G365900 [Glycine max]
Glyma.14G080100	-1.7885	2.52E-05	-	-	-	-	-	-	-	-	2915	ko04626//Plant-pathogen interaction	-	GO:0016301//kinase activity;GO:0032550;GO:0016772//transferase activity, transferring phosphorus-containing groups	GO:0006464//cellular protein modification process	gi|947066158|gb|KRH15301.1|;gi|571508197|ref|XP_006595955.1|;gi|356553923|ref|XP_003545300.1|;gi|571508200|ref|XP_006595956.1|/0;0;0;0/hypothetical protein GLYMA_14G080100 [Glycine max];PREDICTED: mitogen-activated protein kinase kinase kinase 1-like isoform X2 [Glycine max];PREDICTED: MAP kinase kinase kinase mkh1-like isoform X1 [Glycine max];PREDICTED: mitogen-activated protein kinase kinase kinase YODA-like isoform X3 [Glycine max]

Glyma.14G173100	-1.7206	0.001058	-	-	-	-	-	-	-	-	909	ko04626//Plant-pathogen interaction	-	-	-	gi|947067578|gb|KRH16721.1|/0/hypothetical protein GLYMA_14G173100 [Glycine max]
Glyma.14G178000	-	-	-	-	-	-	-1.2401	0.0004	-	-	1985	ko04075//Plant hormone signal transduction;ko04626//Plant-pathogen interaction	-	GO:0005515//protein binding	-	gi|356552929|ref|XP_003544814.1|/0/PREDICTED: transcription factor bHLH96-like [Glycine max]
Glyma.14G186600	-1.5149	0.006118	-	-	-	-	-	-	-	-	4349	ko04626//Plant-pathogen interaction	-	-	-	gi|571511306|ref|XP_006596398.1|/0/PREDICTED: probable disease resistance protein At4g27220 [Glycine max]
Glyma.14G199400	-2.7345	3.53E-10	-	-	-	-	-	-	-	-	2634	ko04626//Plant-pathogen interaction	-	-	-	gi|955362015|ref|XP_014622020.1|/0/PREDICTED: disease resistance protein RPP13-like [Glycine max]
Glyma.14G215800	-1.686	2.05E-05	-	-	-	-	-	-	-	-	426	ko04626//Plant-pathogen interaction	GO:0016020//membrane	GO:0046872//metal ion binding	GO:0009642//response to light intensity;GO:0006952//defense response;GO:0010038//response to metal ion;GO:0001101//response to acid chemical;GO:0000302//response to reactive oxygen species;GO:0009908//flower development;GO:0009725//response to hormone;GO:0048571//long-day photoperiodism;GO:0051171//regulation of nitrogen compound metabolic process	gi|356553299|ref|XP_003544994.1|/2.55184e-97/PREDICTED: probable calcium-binding protein CML18 [Glycine max]
Glyma.15G003300	-	-	-	-	-3.0234	6.87E-05	-	-	-	-	1619	ko04626//Plant-pathogen interaction	-	GO:0001071//nucleic acid binding transcription factor activity;GO:0003677//DNA binding	GO:0098542//defense response to other organism;GO:0006351//transcription, DNA-templated;GO:0010033//response to organic substance;GO:0050789//regulation of biological process	gi|947060391|gb|KRH09652.1|/0/hypothetical protein GLYMA_15G003300 [Glycine max]
Glyma.15G005100	-2.1938	1.92E-05	-	-	-	-	-	-	-	-	1293	ko04075//Plant hormone signal transduction;ko04626//Plant-pathogen interaction	-	GO:0005515//protein binding	-	gi|947060425|gb|KRH09686.1|;gi|356558556|ref|XP_003547571.1|/0;0/hypothetical protein GLYMA_15G005100 [Glycine max];PREDICTED: transcription factor bHLH18-like [Glycine max]
Glyma.15G021300	-1.0055	0.000331	-	-	-	-	-	-	-	-	3188	ko04626//Plant-pathogen interaction	-	GO:0004672//protein kinase activity;GO:0032550	GO:0016310//phosphorylation;GO:0006464//cellular protein modification process	gi|947060712|gb|KRH09973.1|;gi|571514932|ref|XP_006597179.1|/0;0/hypothetical protein GLYMA_15G021300 [Glycine max];PREDICTED: putative leucine-rich repeat receptor-like protein kinase At2g19210 [Glycine max]
Glyma.16G025400	1.45455	0.002021	-	-	-	-	-	-	-	-	2578	ko04626//Plant-pathogen interaction	GO:0031224//intrinsic component of membrane	GO:0005267//potassium channel activity	GO:0034220//ion transmembrane transport;GO:0051707//response to other organism;GO:0030001//metal ion transport	gi|571525040|ref|XP_006598906.1|/0/PREDICTED: putative cyclic nucleotide-gated ion channel 13 [Glycine max]
Glyma.16G031700	-1.2156	7.50E-05	-	-	-	-	-	-	-	-	8487	ko04626//Plant-pathogen interaction	-	-	-	gi|947057171|gb|KRH06577.1|/0/hypothetical protein GLYMA_16G031700 [Glycine max]

Glyma.16G137300	-3.0028	2.00E-08	-	-	-	-	-	-	-	-	3711	ko00230//Purine metabolism;ko00240//Pyrimidine metabolism;ko01100//Metabolic pathways;ko03020//RNA polymerase;ko04626//Plant-pathogen interaction	-	-	GO:0050896//response to stimulus	gi|947058822|gb|KRH08228.1|;gi|947058821|gb|KRH08227.1|/0;0/hypothetical protein GLYMA_16G137300 [Glycine max];hypothetical protein GLYMA_16G137300 [Glycine max]
Glyma.16G172100	1.36638	0.005729	-	-	-	-	-	-	-	-	3246	ko04626//Plant-pathogen interaction	-	-	-	gi|947059346|gb|KRH08752.1|/0/hypothetical protein GLYMA_16G172100 [Glycine max]
Glyma.16G174400	-	-	2.82197	2.07E-09	3.5381	9.27E-06	2.91755	3.96E-06	-	-	1314	ko04626//Plant-pathogen interaction	-	-	-	gi|947059385|gb|KRH08791.1|/0/hypothetical protein GLYMA_16G174400 [Glycine max]
Glyma.16G174500	-	-	2.63316	2.74E-16	3.89368	2.36E-13	3.43608	1.18E-16	-	-	1698	ko04626//Plant-pathogen interaction	-	-	-	gi|947059386|gb|KRH08792.1|/0/hypothetical protein GLYMA_16G174500, partial [Glycine max]
Glyma.16G178800	-	-	-	-	-2.5748	3.91E-08	-	-	-	-	2787	ko04141//Protein processing in endoplasmic reticulum;ko04626//Plant-pathogen interaction	-	GO:0005515//protein binding;GO:0032550	GO:0050896//response to stimulus;GO:0044267//cellular protein metabolic process	gi|571529336|ref|XP_006599549.1|/0/PREDICTED: heat shock protein 83-like [Glycine max]
Glyma.16G202400	4.13681	2.07E-09	-	-	-	-	-	-	-	-	2553	ko04626//Plant-pathogen interaction	-	GO:0036094//small molecule binding;GO:0004672//protein kinase activity;GO:1901363;GO:0097159//organic cyclic compound binding;GO:0032550	GO:0006464//cellular protein modification process	gi|947059794|gb|KRH09200.1|;gi|947059792|gb|KRH09198.1|;gi|356561745|ref|XP_003549139.1|/1.83213e-162;0;0/hypothetical protein GLYMA_16G202400 [Glycine max];hypothetical protein GLYMA_16G202400 [Glycine max];PREDICTED: cysteine-rich receptor-like protein kinase 10 [Glycine max]
Glyma.16G214600	-	-	3.12897	1.70E-06	-	-	-	-	-	-	3135	ko00230//Purine metabolism;ko00240//Pyrimidine metabolism;ko01100//Metabolic pathways;ko03020//RNA polymerase;ko04626//Plant-pathogen interaction	-	GO:0032550	GO:0050896//response to stimulus	gi|947060012|gb|KRH09418.1|/0/hypothetical protein GLYMA_16G214600 [Glycine max]
Glyma.17G085000	-	-	-	-	-	-	1.37737	0.00018	-	-	2698	ko04626//Plant-pathogen interaction	-	GO:0004672//protein kinase activity;GO:0032550	GO:0006464//cellular protein modification process	gi|356562682|ref|XP_003549598.1|/0/PREDICTED: L-type lectin-domain containing receptor kinase S.1-like [Glycine max]
Glyma.17G150400	-1.8486	0.000146	-	-	-	-	-	-	-	-	882	ko04075//Plant hormone signal transduction;ko04626//Plant-pathogen interaction	-	GO:0004672//protein kinase activity;GO:0032550	GO:0006796//phosphate-containing compound metabolic process;GO:0006464//cellular protein modification process	gi|947054816|gb|KRH04269.1|/0/hypothetical protein GLYMA_17G150400 [Glycine max]
Glyma.17G150600	-1.4012	0.003606	-	-	-	-	-	-	-	-	2166	ko04075//Plant hormone signal transduction;ko04626//Plant-pathogen interaction	-	GO:0004672//protein kinase activity;GO:0032550	GO:0006464//cellular protein modification process	gi|571536659|ref|XP_003550928.2|/0/PREDICTED: probable L-type lectin-domain containing receptor kinase S.7 [Glycine max]
Glyma.17G185300	-1.8004	0.002111	-	-	6.25467	2.26E-09	-	-	-	-	516	ko04626//Plant-pathogen interaction	-	-	-	gi|947055314|gb|KRH04767.1|/1.54976e-111/hypothetical protein GLYMA_17G185300 [Glycine max]
Glyma.17G187200	-1.0292	4.88E-05	-	-	-	-	-	-	-	-	2779	ko04075//Plant hormone signal transduction;ko04626//	-	GO:0016301//kinase activity;GO:0032550	GO:0006796//phosphate-containing compound metabolic process;GO:0006464//cellular	gi|955377781|ref|XP_014624839.1|/0/PREDICTED: probable inactive leucine-rich repeat receptor-like protein kinase At3g03770

												Plant-pathogen interaction			protein modification process	[Glycine max]
Glyma. 18G026 900	-12.34	2.08E-46	-9.977	4.88E-37	-10.577	9.89E-09	-3.3592	0.00107	-	-	1413	ko04626//Plant-pathogen interaction	-	GO:0016772//transferase activity, transferring phosphorus-containing groups	-	gi|955382928|ref|XP_003552842.3|/0/PREDICTED: putative serine/threonine-protein kinase-like protein CCR3 [Glycine max]
Glyma. 18G076 200	1.01857	0.001701	-	-	-	-	-	-	-	-	1764	ko04075//Plant hormone signal transduction;ko04626//Plant-pathogen interaction	-	GO:0004672//protein kinase activity;GO:0032550	GO:0006796//phosphate-containing compound metabolic process;GO:0006464//cellular protein modification process	gi|947048945|gb|KRG98473.1|/0/hypothetical protein GLYMA_18G076200 [Glycine max]
Glyma. 18G091 800	-	-	-2.3481	0.000322	-	-	-2.8488	0.00028	-	-	2859	ko04626//Plant-pathogen interaction	-	-	-	gi|947049105|gb|KRG98633.1|/5.16573e-129/hypothetical protein GLYMA_18G086300 [Glycine max]
Glyma. 18G204 500	-	-	-	-	5.03732	7.39E-27	6.40279	6.09E-28	-	-	3138	ko04626//Plant-pathogen interaction	-	-	-	gi|947050770|gb|KRH00299.1|/0/hypothetical protein GLYMA_18G204500 [Glycine max]
Glyma. 18G219 800	-2.6256	6.49E-11	-	-	-	-	3.43879	9.85E-05	-	-	2944	ko04626//Plant-pathogen interaction	-	GO:0016301//kinase activity;GO:0036094//small molecule binding;GO:0004672//protein kinase activity;GO:1901363;GO:0097159//organic cyclic compound binding	GO:0044237//cellular metabolic process	gi|571548055|ref|XP_006602745.1|;gi|947051019|gb|KRH00548.1|/0;0/PREDICTED: cysteine-rich receptor-like protein kinase 7 isoform X1 [Glycine max];hypothetical protein GLYMA_18G219800 [Glycine max]
Glyma. 18G221 400	-	-	-	-	2.33321	0.00077	-	-	-	-	2001	ko04626//Plant-pathogen interaction	GO:0043231//intracellular membrane-bounded organelle	GO:0005085//guanyl-nucleotide exchange factor activity	GO:0033124;GO:0009553//embryo sac development;GO:0032011//ARF protein signal transduction	gi|947051053|gb|KRH00582.1|/1.48183e-178/hypothetical protein GLYMA_18G221400 [Glycine max]
Glyma. 18G226 300	-6.9116	4.87E-32	-	-	-	-	-6.3361	2.09E-13	-	-	9435	ko04626//Plant-pathogen interaction	-	-	-	gi|947051119|gb|KRH00648.1|/0/hypothetical protein GLYMA_18G226300 [Glycine max]
Glyma. 18G226 500	-10.449	1.13E-25	-	-	-8.3636	1.56E-06	-7.747	3.75E-13	-	-	10583	ko04626//Plant-pathogen interaction	-	GO:0036094//small molecule binding;GO:0097159//organic cyclic compound binding;GO:1901363	-	gi|947051121|gb|KRH00650.1|/0/hypothetical protein GLYMA_18G226500 [Glycine max]
Glyma. 18G226 800	-6.9125	2.43E-45	-	-	-4.311	1.55E-07	-4.3603	2.88E-12	-	-	6246	ko04626//Plant-pathogen interaction	-	GO:0036094//small molecule binding;GO:0097159//organic cyclic compound binding;GO:1901363	-	gi|947051124|gb|KRH00653.1|/0/hypothetical protein GLYMA_18G226800 [Glycine max]
Glyma. 18G226 900	-9.0754	1.98E-16	-	-	-	-	-	-	-	-	4664	ko04626//Plant-pathogen interaction	-	-	-	gi|947051125|gb|KRH00654.1|/0/hypothetical protein GLYMA_18G226900 [Glycine max]
Glyma. 18G270 800	-	-	-	-	3.77425	7.90E-08	-	-	-	-	2730	ko04626//Plant-pathogen interaction	-	GO:0004672//protein kinase activity	GO:0006796//phosphate-containing compound metabolic process	gi|947051816|gb|KRH01345.1|/0/hypothetical protein GLYMA_18G270800 [Glycine max]
Glyma. 18G281 700	1.61515	0.002441	-	-	-	-	-	-	-	-	4047	ko04626//Plant-pathogen interaction	-	-	-	gi|571549792|ref|XP_003551801.2|/0/PREDICTED: probable disease resistance protein At4g27220 [Glycine max]
Glyma. 18G287 000	-	-	-2.3485	5.02E-05	-	-	-	-	-	-	3158	ko04626//Plant-pathogen interaction	-	-	-	gi|571549977|ref|XP_006603027.1|/0/PREDICTED: disease resistance protein RPP13-like [Glycine max]

Glyma.20G034200	6.78477	1.36E-25	-	-	-	-	-	-	-	-	828	ko04626//Plant-pathogen interaction	-	-	-	gi|947039863|gb|KRG89587.1|;gi|734395097|gb|KHN28802.1|/2.21565e-122;3.13952e-127/hypothetical protein GLYMA_20G034200 [Glycine max];Putative calcium-binding protein CML45 [Glycine soja]
Glyma.20G132400	-	-	-	-	-1.0367	0.00092	-	-	-	-	1141	ko04075//Plant hormone signal transduction;ko04626//Plant-pathogen interaction	-	GO:0016772//transferase activity, transferring phosphorus-containing groups	GO:0006796//phosphate-containing compound metabolic process	gi|955390929|ref|XP_014627745.1|/1.80076e-124/PREDICTED: somatic embryogenesis receptor kinase 1-like isoform X1 [Glycine max]
Glyma.U008900	-1.4994	0.000507	-	-	-	-	-	-	-	-	2589	ko04626//Plant-pathogen interaction	-	-	-	gi|947038196|gb|KRG88521.1|/0/hypothetical protein GLYMA_U008900 [Glycine max]
Glyma.U008800	5.37147	1.07E-22	5.18968	1.69E-24	-	-	-	-	10.5015	2.00E-10	1738	ko04626//Plant-pathogen interaction	-	-	-	gi|947038195|gb|KRG88520.1|/0/hypothetical protein GLYMA_U008800 [Glycine max]
Glyma.06G125600	-	-	-	-	-	-	-	-	7.82888	9.42E-06	1743	ko04626//Plant-pathogen interaction	-	GO:0003677//DNA binding;GO:0001071//nucleic acid binding transcription factor activity	GO:0006351//transcription, DNA-templated	gi|959092742|ref|NP_001304644.1|/0/probable WRKY transcription factor 53 [Glycine max]
Glyma.10G002200	-	-	-	-	-	-	-	-	7.59311	8.50E-05	1062	ko04626//Plant-pathogen interaction	-	GO:0046872//metal ion binding	GO:0019932//second-messenger-mediated signaling	gi|351727589|ref|NP_001236910.1|/1.00985e-102/uncharacterized protein LOC100526987 [Glycine max]
Glyma.16G137600	3.06089	4.26E-05	2.32125	3.36E-06	-	-	3.371	2.26E-06	6.77516	1.60E-16	3555	ko00230//Purine metabolism;ko00240//Pyrimidine metabolism;ko01100//Metabolic pathways;ko03020//RNA polymerase;ko04626//Plant-pathogen interaction	-	-	GO:0050896//response to stimulus	gi|955374830|ref|XP_003548883.2|/0/PREDICTED: TMV resistance protein N-like [Glycine max]
Glyma.16G026400	-	-	-	-	-	-	-	-	5.61978	6.51E-11	1825	ko04626//Plant-pathogen interaction	-	GO:0003677//DNA binding;GO:0001071//nucleic acid binding transcription factor activity	GO:0006351//transcription, DNA-templated	gi|918463837|gb|ALA09263.1|/0/WRKY transcription factor, partial [Glycine max]
Glyma.09G005700	-	-	-	-	-	-	-	-	5.51639	5.20E-08	1942	ko04626//Plant-pathogen interaction	-	-	-	gi|356532095|ref|XP_003534609.1|/0/PREDICTED: probable WRKY transcription factor 31 [Glycine max]
Glyma.20G137900	-	-	-	-	-	-	-	-	5.32995	1.07E-05	2338	ko04626//Plant-pathogen interaction	-	GO:0016301//kinase activity;GO:0004672//protein kinase activity	-	gi|356575757|ref|XP_003556003.1|;gi|947041445|gb|KRG91169.1|/0;0/PREDICTED: putative receptor-like protein kinase At4g00960 [Glycine max];hypothetical protein GLYMA_20G137900 [Glycine max]
Glyma.09G099900	-	-	-	-	-	-	-	-	4.98321	1.33E-11	3235	ko04075//Plant hormone signal transduction;ko04626//Plant-pathogen interaction	-	GO:0004672//protein kinase activity;GO:0032550	GO:0006464//cellular protein modification process	gi|955340766|ref|XP_006587157.2|;gi|955340768|ref|XP_014617538.1|/0;0/PREDICTED: probable LRR receptor-like serine/threonine-protein kinase At1g56130 isoform X1 [Glycine max];PREDICTED: probable LRR receptor-like serine/threonine-protein kinase At1g56130 isoform X2 [Glycine max]
Glyma.12G011700	-	-	-	-	-	-	-	-	4.85284	5.67E-12	3539	ko04626//Plant-pathogen interaction	-	-	-	gi|571491619|ref|XP_006591996.1|/0/PREDICTED: disease resistance protein RPP13-like [Glycine max]
Glyma.03G054900	-	-	-	-	-	-	-	-	4.77784	1.80E-06	3248	ko04626//Plant-pathogen interaction	-	GO:0004672//protein kinase activity;GO:0032550	GO:0006796//phosphate-containing compound metabolic process;GO:0006464//cellular protein modification process;GO:0008037//cell	gi|571444460|ref|XP_006576519.1|;gi|571444463|ref|XP_006576520.1|/0;0/PREDICTED: G-type lectin S-receptor-like serine/threonine-protein kinase At4g27290 isoform X1 [Glycine max];PREDICTED:

															recognition	G-type lectin S-receptor-like serine/threonine-protein kinase At4g27290 isoform X3 [Glycine max]
Glyma.16G137200	1.91235	0.000121	-	-	-	-	-	-	4.77561	7.66E-06	1648	ko04626//Plant-pathogen interaction	-	-	-	gi|955374835|ref|XP_003547976.2|/0/PREDICTED: TMV resistance protein N [Glycine max]
Glyma.11G163300	-	-	-	-	-	-	-	-	4.73518	0.001	2355	ko04626//Plant-pathogen interaction	-	-	-	gi|356539680|ref|XP_003538323.1|/0/PREDICTED: probable WRKY transcription factor 25 [Glycine max]
Glyma.07G078000	-	-	-	-	-	-	-	-	4.40047	0.00015	3561	ko04626//Plant-pathogen interaction	-	GO:0036094//small molecule binding;GO:0097159//organic cyclic compound binding;GO:1901363	-	gi|955328410|ref|XP_014632873.1|/0/PREDICTED: disease resistance protein RPS2-like [Glycine max]
Glyma.04G078400	-	-	-	-	-	-	-	-	4.29879	1.95E-05	955	ko04626//Plant-pathogen interaction	-	GO:0046872//metal ion binding	-	gi|351721210|ref|NP_001236178.1|/6.40392e-110/uncharacterized protein LOC100527700 [Glycine max]
Glyma.15G110300	-	-	-	-	-	-	-	-	4.29237	0.0001	2099	ko04626//Plant-pathogen interaction	-	-	-	gi|356555684|ref|XP_003546160.1|/0/PREDICTED: probable WRKY transcription factor 31 [Glycine max]
Glyma.13G266100	-	-	-	-	-	-	-	-	4.22161	6.33E-05	3569	ko04626//Plant-pathogen interaction	-	GO:0004672//protein kinase activity;GO:0032550	GO:0006796//phosphate-containing compound metabolic process;GO:0006464//cellular protein modification process	gi|734413477|gb|KHN36922.1|/0/Putative leucine-rich repeat receptor-like serine/threonine-protein kinase [Glycine soja]
Glyma.13G303900	-	-	-	-	-	-	-	-	4.15709	3.46E-05	965	ko04626//Plant-pathogen interaction	-	-	-	gi|356547045|ref|XP_003541928.1|/1.29939e-69/PREDICTED: calcium-binding protein PBP1-like [Glycine max]
Glyma.08G229100	-	-	1.83596	1.93E-10	2.12347	5.74E-11	-	-	4.08162	2.23E-14	3020	ko04626//Plant-pathogen interaction	-	GO:0016301//kinase activity;GO:0032550	GO:0006464//cellular protein modification process	gi|571472730|ref|XP_006585700.1|/0/PREDICTED: probable inactive leucine-rich repeat receptor-like protein kinase At3g03770 [Glycine max]
Glyma.07G001400	2.29703	1.07E-05	3.38976	2.07E-11	3.1603	9.54E-10	3.1691	4.00E-05	4.06632	5.32E-06	2996	ko04626//Plant-pathogen interaction	-	GO:0016772//transferase activity, transferring phosphorus-containing groups	-	gi|571464153|ref|XP_006582973.1|/0/PREDICTED: U-box domain-containing protein 35-like isoform X1 [Glycine max]
Glyma.18G141500	-1.3615	0.000655	-	-	-	-	-	-	4.055	1.77E-06	2617	ko04075//Plant hormone signal transduction;ko04626//Plant-pathogen interaction	GO:0005911//cell-cell junction;GO:0016020//membrane	GO:0004672//protein kinase activity;GO:0032550	GO:0006468//protein phosphorylation;GO:0000302//response to reactive oxygen species	gi|571545655|ref|XP_003551216.2|/0/PREDICTED: cysteine-rich receptor-like protein kinase 2 [Glycine max]
Glyma.01G204400	-	-	-	-	-	-	-	-	4.0261	3.69E-05	1572	ko04075//Plant hormone signal transduction;ko04626//Plant-pathogen interaction	-	-	-	gi|734424424|gb|KHN42652.1|;gi|947129438|gb|KRH77292.1|/7.232e-138;1.9617e-107/Protein TIFY 10B [Glycine soja];hypothetical protein GLYMA_01G204400 [Glycine max]
Glyma.07G127100	-	-	-	-	-	-	-	-	4.01405	1.43E-05	3329	ko04626//Plant-pathogen interaction	-	GO:0004672//protein kinase activity;GO:0032550	GO:0006796//phosphate-containing compound metabolic process;GO:0006464//cellular protein modification process	gi|571463975|ref|XP_006582888.1|;gi|955328256|ref|XP_014632820.1|/0;0/PREDICTED: probable LRR receptor-like serine/threonine-protein kinase At4g29180-like isoform X1 [Glycine max];PREDICTED: probable LRR receptor-like serine/threonine-protein kinase At4g29180-like isoform X2 [Glycine max]
Glyma.09G182200	-	-	-	-	-	-	-	-	3.98403	6.48E-05	1785	ko04075//Plant hormone signal transduction;ko04626//Plant-pathogen interaction	-	GO:0016301//kinase activity	-	gi|947090495|gb|KRH39160.1|/0/hypothetical protein GLYMA_09G182200 [Glycine max]

20 × 20

Glyma.08G142400	1.38593	0.004566	-	-	-	-	-	-	3.90859	1.61E-14	1059	ko04626//Plant-pathogen interaction	-	GO:0001071//nucleic acid binding transcription factor activity;GO:0003677//DNA binding	GO:0050896//response to stimulus;GO:0006351//transcription, DNA-templated	gi|255639287|gb|ACU19941.1|/1.44336e-110/unknown [Glycine max]
Glyma.05G215900	-	-	-	-	-	-	-	-	3.90576	0.00018	1807	ko04626//Plant-pathogen interaction	-	GO:0003677//DNA binding;GO:0001071//nucleic acid binding transcription factor activity	GO:0006351//transcription, DNA-templated	gi|356513295|ref|XP_003525349.1|/0/PREDICTED: probable WRKY transcription factor 41 [Glycine max]
Glyma.08G235900	-	-	-	-	-	-	-	-	3.84501	0.00738	3545	ko04075//Plant hormone signal transduction;ko04626//Plant-pathogen interaction	-	GO:0004672//protein kinase activity;GO:0032550	GO:0006464//cellular protein modification process	gi|356526421|ref|XP_003531816.1|/0/PREDICTED: probable LRR receptor-like serine/threonine-protein kinase At1g56130 isoform X1 [Glycine max]
Glyma.03G047700	-	-	-	-	-	-	-	-	3.78387	8.11E-05	2490	ko04626//Plant-pathogen interaction	-	-	GO:0050896//response to stimulus	gi|947117339|gb|KRH65588.1|/0/hypothetical protein GLYMA_03G047700, partial [Glycine max]
Glyma.11G075200	-	-	-	-	-	-	-	-	3.70245	2.53E-05	4641	ko04626//Plant-pathogen interaction	GO:0031224//intrinsic component of membrane	GO:0004672//protein kinase activity;GO:0032550	GO:0006796//phosphate-containing compound metabolic process;GO:0006464//cellular protein modification process	gi|955349679|ref|XP_014619378.1|/0/PREDICTED: probable LRR receptor-like serine/threonine-protein kinase At4g36180 [Glycine max]
Glyma.16G127900	-	-	-	-	-	-	-	-	3.69577	4.64E-08	7518	ko00230//Purine metabolism;ko00240//Pyrimidine metabolism;ko01100//Metabolic pathways;ko03020//RNA polymerase;ko04626//Plant-pathogen interaction	-	GO:0032550	GO:0050896//response to stimulus	gi|947058663|gb|KRH08069.1|;gi|947058664|gb|KRH08070.1|;gi|947058662|gb|KRH08068.1|/0;0;0/hypothetical protein GLYMA_16G127900 [Glycine max];hypothetical protein GLYMA_16G127900 [Glycine max];hypothetical protein GLYMA_16G127900 [Glycine max]
Glyma.01G117900	-	-	-	-	-	-	-	-	3.67399	0.00036	4216	ko04075//Plant hormone signal transduction;ko04626//Plant-pathogen interaction	-	GO:0004672//protein kinase activity;GO:0032550	GO:0006796//phosphate-containing compound metabolic process;GO:0006464//cellular protein modification process;GO:0008037//cell recognition	gi|571435020|ref|XP_006573359.1|;gi|947128048|gb|KRH75902.1|;gi|571435014|ref|XP_006573356.1|;gi|947128051|gb|KRH75905.1|;gi|947128060|gb|KRH75914.1|/0;0;0;0;0/PREDICTED: G-type lectin S-receptor-like serine/threonine-protein kinase At4g27290 isoform X2 [Glycine max];hypothetical protein GLYMA_01G117900 [Glycine max];PREDICTED: G-type lectin S-receptor-like serine/threonine-protein kinase At4g27290 isoform X1 [Glycine max];hypothetical protein GLYMA_01G117900 [Glycine max];hypothetical protein GLYMA_01G117900 [Glycine max]
Glyma.07G023300	-	-	-	-	-4.0866	0.00042	-	-	3.67244	0.00039	1692	ko04626//Plant-pathogen interaction	-	-	GO:0098542//defense response to other organism;GO:0010033//response to organic substance;GO:0050789//regulation of biological process;GO:0009987//cellular process	gi|918463825|gb|ALA09257.1|/8.85387e-180/WRKY transcription factor, partial [Glycine max]
Glyma.18G281600	-	-	-	-	-	-	-	-	3.67154	0.00016	3793	ko04626//Plant-pathogen interaction	-	-	-	gi|947051977|gb|KRH01506.1|/0/hypothetical protein GLYMA_18G2816002, partial [Glycine max]
Glyma.05G119500	-	-	-	-	-3.3414	0.00015	-	-	3.57315	0.0002	2761	ko04075//Plant hormone signal transduction;ko04626//Plant-pathogen	GO:0032991//macromolecular complex;G	GO:0005102//receptor binding;GO:0032550;GO:0004672//protein kinase activity;GO:0046983//prot	GO:0006984//ER-nucleus signaling pathway;GO:0009620//response to	gi|356510695|ref|XP_003524071.1|/0/PREDICTED: BRASSINOSTEROID INSENSITIVE 1-associated receptor kinase 1-like [Glycine max]

												interaction	O:0043231//intracellular membrane-bounded organelle;GO:0031224//intrinsic component of membrane	ein dimerization activity	fungus;GO:0040007//growth;GO:0010260//organ senescence;GO:0002239//response to oomycetes;GO:0009617//response to bacterium;GO:0006605//protein targeting;GO:0048580;GO:0006796//phosphate-containing compound metabolic process;GO:0010243//response to organonitrogen compound;GO:0002252;GO:0043401//steroid hormone mediated signaling pathway;GO:0006464//cellular protein modification process;GO:0009626//plant-type hypersensitive response	
Glyma.08G044400	-	-	-	-	-	-	-	-	3.54849	5.37E-05	2095	ko04070//Phosphatidylinositol signaling system;ko04626//Plant-pathogen interaction	GO:0043231//intracellular membrane-bounded organelle	GO:0001071//nucleic acid binding transcription factor activity;GO:0003677//DNA binding	GO:0002831;GO:0010337//regulation of salicylic acid metabolic process;GO:0006351//transcription, DNA-templated	gi|947093104|gb|KRH41689.1|;gi|356524378|ref|XP_003530806.1|/0;0/hypothetical protein GLYMA_08G044400 [Glycine max];PREDICTED: protein SAR DEFICIENT 1 [Glycine max]
Glyma.06G265000	-	-	-	-	-	-	-	-	3.47598	0.00051	3933	ko04626//Plant-pathogen interaction	-	-	-	gi|955326975|ref|XP_014632562.1|/0/PREDICTED: disease resistance protein TAO1-like [Glycine max]
Glyma.13G181400	-	-	-	-	-	-	-	-	3.38634	5.12E-07	1719	ko04626//Plant-pathogen interaction	-	GO:0004672//protein kinase activity;GO:0032550	GO:0006464//cellular protein modification process	gi|571498823|ref|XP_006594324.1|/0/PREDICTED: putative serine/threonine-protein kinase [Glycine max]
Glyma.09G274000	-	-	-	-	-	-	-	-	3.33606	0.00369	1194	ko04626//Plant-pathogen interaction	-	GO:0003677//DNA binding;GO:0001071//nucleic acid binding transcription factor activity	GO:0006351//transcription, DNA-templated	gi|937500778|ref|NP_001302438.1|/0/probable WRKY transcription factor 70-like [Glycine max]
Glyma.10G178400	-	-	-	-	-	-	-	-	3.24413	0.00016	1548	ko04626//Plant-pathogen interaction	-	GO:0046872//metal ion binding	GO:0019932//second-messenger-mediated signaling	gi|351726666|ref|NP_001237902.1|/1.76727e-103/calmodulin [Glycine max]
Glyma.08G039400	-	-	-	-	-	-	-	-	3.21692	0.0003	2865	ko04626//Plant-pathogen interaction	-	-	-	gi|356527793|ref|XP_003532491.1|/0/PREDICTED: leucine-rich repeat extensin-like protein 4 [Glycine max]
Glyma.06G255900	-	-	-	-	-	-	-	-	3.19763	0.00047	2845	ko04626//Plant-pathogen interaction	-	GO:0036094//small molecule binding;GO:0004672//protein kinase activity;GO:1901363;GO:0097159//organic cyclic compound binding	GO:0006796//phosphate-containing compound metabolic process	gi|356514857|ref|XP_003526119.1|/0/PREDICTED: G-type lectin S-receptor-like serine/threonine-protein kinase At1g11300 [Glycine max]
Glyma.03G046500	-1.4483	0.000234	-	-	1.29715	0.00065	-	-	3.19249	9.50E-05	3498	ko00230//Purine metabolism;ko00240//Pyrimidine metabolism;ko01100//Metabolic pathways;ko03020//RNA polymerase;ko04626//Plant-pathogen interaction	-	-	-	gi|955310888|ref|XP_006577442.2|/0/PREDICTED: putative disease resistance RPP13-like protein 1 [Glycine max]
Glyma.05G237200	2.50182	0.000159	-	-	-	-	-	-	3.17974	0.00137	2018	ko04070//Phosphatidylinositol signaling system;ko04626//Plant-	GO:0043231//intracellular	GO:0001071//nucleic acid binding transcription factor	GO:0002831;GO:0010337//regulation of salicylic acid metabolic	gi|356513070|ref|XP_003525237.1|/0/PREDICTED: protein SAR DEFICIENT 1-like [Glycine max]

												pathogen interaction	membrane -bounded organelle	activity;GO:0003677//DNA binding	process;GO:0006351//transcription, DNA-templated	
Glyma. 12G103 600	-	-	-	-	-	-	-	-	3.16545	2.50E-05	1075	ko04070//Phosphatidylinositol signaling system;ko04626//Plant-pathogen interaction	-	GO:0046872//metal ion binding	-	gi|734380494|gb|KHN22869.1|/4.28219e-76/Calcium-binding protein PBP1 [Glycine soja]
Glyma. 11G157 200	-	-	-	-	-	-	-	-	3.15232	0.00203	816	ko04626//Plant-pathogen interaction	-	GO:0046872//metal ion binding	-	gi|947081295|gb|KRH30084.1|/4.03735e-97/hypothetical protein GLYMA_11G157200 [Glycine max]
Glyma. 02G232 600	-	-	-	-	-	-	-	-	3.13126	2.66E-06	2179	ko04626//Plant-pathogen interaction	-	-	-	gi|356500910|ref|XP_003519273.1|/0/PREDICTED: probable WRKY transcription factor 25 [Glycine max]
Glyma. 14G199 800	-1.3358	0.001056	-	-	-	-	-	-	3.12085	0.00119	1328	ko04626//Plant-pathogen interaction	-	-	GO:0009987//cellular process	gi|571511635|ref|XP_006596452.1|/0/PREDICTED: uncharacterized protein LOC102667674 [Glycine max]
Glyma. 14G200 200	-	-	-	-	-3.8331	0.00015	-	-	3.11345	0.00081	2778	ko04626//Plant-pathogen interaction	-	GO:0003677//DNA binding;GO:0001071//nucleic acid binding transcription factor activity	GO:0006351//transcription, DNA-templated	gi|356553124|ref|XP_003544908.1|;gi|947067983|gb|KRH17126.1|/0;0/PREDICTED: probable WRKY transcription factor 25 [Glycine max];hypothetical protein GLYMA_14G200200 [Glycine max]
Glyma. 19G229 400	-	-	-	-	-	-	-	-	3.09356	0.00074	2279	ko04070//Phosphatidylinositol signaling system;ko04626//Plant-pathogen interaction	-	-	-	gi|571559977|ref|XP_006604792.1|/0/PREDICTED: calmodulin-binding protein 60 D isoform X1 [Glycine max]
Glyma. 02G150 600	-	-	-	-	-	-	-	-	3.0682	0.00046	2237	ko04075//Plant hormone signal transduction;ko04626//Plant-pathogen interaction	-	GO:0004672//protein kinase activity;GO:0032550	GO:0006464//cellular protein modification process	gi|356502836|ref|XP_003520221.1|/0/PREDICTED: probable LRR receptor-like serine/threonine-protein kinase RKF3 [Glycine max]
Glyma. 10G113 300	-	-	-	-	-	-	-	-	3.06199	0.00077	708	ko04626//Plant-pathogen interaction	GO:0005622//intracellular	GO:0097159//organic cyclic compound binding;GO:1901363	GO:0071704//organic substance metabolic process;GO:0044238//primary metabolic process;GO:0044237//cellular metabolic process	gi|356537413|ref|XP_003537222.1|/5.59253e-109/PREDICTED: elongation factor Tu, chloroplastic-like [Glycine max]
Glyma. 08G021 900	-	-	-	-	-	-	-	-	3.05728	0.0035	1990	ko04626//Plant-pathogen interaction	-	GO:0003677//DNA binding;GO:0001071//nucleic acid binding transcription factor activity	GO:0006351//transcription, DNA-templated	gi|918463829|gb|ALA09259.1|/0/WRKY transcription factor [Glycine max]
Glyma. 06G142 000	-	-	-	-	-	-	-	-	3.05627	0.00207	1605	ko04626//Plant-pathogen interaction	-	GO:0003677//DNA binding;GO:0001071//nucleic acid binding transcription factor activity	GO:0006351//transcription, DNA-templated	gi|356516231|ref|XP_003526799.1|/0/PREDICTED: probable WRKY transcription factor 70 [Glycine max]
Glyma. 11G043 700	-	-	-	-	-	-	-	-	3.01001	0.00418	1758	ko04075//Plant hormone signal transduction;ko04626//Plant-pathogen interaction	-	-	-	gi|356540613|ref|XP_003538782.1|/0/PREDICTED: transcription factor bHLH25-like [Glycine max]
Glyma. 20G169 000	-	-	-	-	-	-	-	-	2.9442	1.53E-07	2125	ko04075//Plant hormone signal transduction;ko04626//Plant-pathogen interaction	-	GO:0004672//protein kinase activity;GO:0032550	GO:0016310//phosphorylation;GO:0006464//cellular protein modification process	gi|351723997|ref|NP_001238066.1|/0/serine/threonine protein kinase-like protein [Glycine max]
Glyma. 18G281 500	-	-	-	-	-	-	-	-	2.93243	0.00026	4414	ko04626//Plant-pathogen interaction	-	-	-	gi|955383602|ref|XP_014626225.1|/0/PREDICTED: probable disease resistance protein At5g47250 isoform X1 [Glycine max]
Glyma. 11G207 500	-	-	-	-	-	-	-	-	2.90831	0.00068	2592	ko04075//Plant hormone signal transduction;ko04626//Plant-pathogen	-	GO:0004672//protein kinase activity;GO:0032550	GO:0006464//cellular protein modification process	gi|356537805|ref|XP_003537415.1|/0/PREDICTED: cysteine-rich receptor-like protein kinase 2 [Glycine max]

												interaction				
Glyma.05G139500	-	-	-	-	-	-	-	-	2.90743	0.00015	1643	ko04626//Plant-pathogen interaction	-	GO:0004672//protein kinase activity;GO:0032550	GO:0006796//phosphate-containing compound metabolic process;GO:0006464//cellular protein modification process	gi|955320825|ref|XP_014631147.1|;gi|734432012|gb|KHN46091.1|/0;0/PREDICTED: cysteine-rich receptor-like protein kinase 10 isoform X2 [Glycine max];Cysteine-rich receptor-like protein kinase 10 [Glycine soja]
Glyma.15G064900	-	-	-	-	-	-	-	-	2.90364	0.00227	2760	ko04626//Plant-pathogen interaction	-	GO:0004672//protein kinase activity;GO:0032550	GO:0006796//phosphate-containing compound metabolic process;GO:0006464//cellular protein modification process;GO:0008037//cell recognition	gi|955366151|ref|XP_006597385.2|/0/PREDICTED: G-type lectin S-receptor-like serine/threonine-protein kinase At4g27290 isoform X2 [Glycine max]
Glyma.13G316100	-	-	-	-	-	-	-	-	2.88609	0.0017	1153	ko04626//Plant-pathogen interaction	-	GO:0046872//metal ion binding	-	gi|947073780|gb|KRH22671.1|;gi|947073781|gb|KRH22672.1|/7.33047e-134;3.22811e-132/hypothetical protein GLYMA_13G316100 [Glycine max];hypothetical protein GLYMA_13G316100 [Glycine max]
Glyma.02G100300	-	-	-	-	-	-	-	-	2.85311	0.00188	2319	ko04626//Plant-pathogen interaction	-	GO:0001871;GO:0004672//protein kinase activity;GO:0032550	GO:0006796//phosphate-containing compound metabolic process;GO:0006464//cellular protein modification process	gi|351725961|ref|NP_001238646.1|/0/stress-induced receptor-like kinase precursor [Glycine max]
Glyma.17G062300	-	-	-	-	-	-	-	-	2.82178	0.0016	1829	ko04626//Plant-pathogen interaction	-	GO:0004672//protein kinase activity;GO:0032550	GO:0006796//phosphate-containing compound metabolic process;GO:0006464//cellular protein modification process	gi|947053400|gb|KRH02853.1|;gi|947053399|gb|KRH02852.1|;gi|351724607|ref|NP_001238599.1|/0;0;0/hypothetical protein GLYMA_17G062300 [Glycine max];hypothetical protein GLYMA_17G062300 [Glycine max];protein kinase family protein [Glycine max]
Glyma.01G118300	-1.3246	0.000513	-	-	-	-	-	-	2.81805	0.0014	1776	ko04626//Plant-pathogen interaction	-	-	-	gi|947128075|gb|KRH75929.1|;gi|947128074|gb|KRH75928.1|/0;0/hypothetical protein GLYMA_01G118300 [Glycine max];hypothetical protein GLYMA_01G118300 [Glycine max]
Glyma.07G057400	-	-	-	-	-	-	-	-	2.79984	0.00379	1907	ko04626//Plant-pathogen interaction	-	GO:0003677//DNA binding;GO:0001071//nucleic acid binding transcription factor activity	GO:0006351//transcription, DNA-templated	gi|918463827|gb|ALA09258.1|/0/WRKY transcription factor [Glycine max]
Glyma.18G260700	-	-	-	-	-	-	-	-	2.7992	0.00068	1307	ko04626//Plant-pathogen interaction	-	GO:0046872//metal ion binding	-	gi|955382091|ref|XP_014625776.1|;gi|571549120|ref|XP_006602906.1|/1.78052e-91;1.33475e-135/PREDICTED: probable calcium-binding protein CML45 isoform X2 [Glycine max];PREDICTED: probable calcium-binding protein CML45 isoform X1 [Glycine max]
Glyma.11G157100	-	-	-	-	-	-	-	-	2.79134	0.00455	806	ko04626//Plant-pathogen interaction	-	GO:0046872//metal ion binding	-	gi|947081294|gb|KRH30083.1|/6.00745e-97/hypothetical protein GLYMA_11G157100 [Glycine max]
Glyma.09G236800	-	-	-	-	-	-	-	-	2.78314	0.00455	1250	ko04626//Plant-pathogen interaction	-	GO:0046872//metal ion binding	-	gi|356531760|ref|XP_003534444.1|/7.58254e-149/PREDICTED: probable calcium-binding protein CML45 [Glycine max]
Glyma.18G213200	-	-	-	-	-	-	-	-	2.77615	0.00754	1171	ko04626//Plant-pathogen interaction	-	GO:0003677//DNA binding;GO:0001071//nucleic acid binding transcription factor activity	GO:0006351//transcription, DNA-templated	gi|351725863|ref|NP_001237619.1|/0/transcription factor [Glycine max]
Glyma.12G073000	-	-	-	-	-4.3858	4.89E-05	-	-	2.75161	2.02E-06	2041	ko04650//Natural killer cell mediated cytotoxicity;ko04626//Plant-pathogen interaction	-	GO:0004702//receptor signaling protein serine/threonine kinase activity;GO:0032550	GO:0045682//regulation of epidermis development;GO:0051707//response to other organism;GO:0009411//response to	gi|356542571|ref|XP_003539740.1|/0/PREDICTED: mitogen-activated protein kinase 3 [Glycine max]

															UV;GO:0009700//indole phytoalexin biosynthetic process;GO:0010243//response to organonitrogen compound;GO:0090567;GO:0080135//regulation of cellular response to stress;GO:0000187//activation of MAPK activity	
Glyma.17G096000	-	-	-	-	-	-	-	-	2.74555	0.0005	2393	ko04626//Plant-pathogen interaction	-	GO:0046872//metal ion binding;GO:0004672//protein kinase activity;GO:0032550	GO:0006464//cellular protein modification process	gi|356565063|ref|XP_003550764.1|/0/PREDICTED: calcium-dependent protein kinase 10-like [Glycine max]
Glyma.15G043800	-	-	-	-	-	-	-	-	2.7409	0.00023	1878	ko04626//Plant-pathogen interaction	-	GO:0004672//protein kinase activity;GO:0032550	GO:0006796//phosphate-containing compound metabolic process;GO:0006464//cellular protein modification process	gi|356557471|ref|XP_003547039.1|/0/PREDICTED: probable serine/threonine-protein kinase RLCKVII [Glycine max]
Glyma.16G172300	-	-	-	-	-	-	-	-	2.7281	0.00374	3398	ko04626//Plant-pathogen interaction	-	-	-	gi|571529127|ref|XP_006599514.1|/0/PREDICTED: leucine-rich repeat receptor protein kinase MSL1-like [Glycine max]
Glyma.09G061900	-	-	-	-	-	-	-	-	2.71067	3.81E-06	1572	ko04626//Plant-pathogen interaction	-	-	-	gi|947088702|gb|KRH37367.1|/1.4299e-148/hypothetical protein GLYMA_09G061900 [Glycine max]
Glyma.04G245000	-	-	-	-	-	-	-	-	2.69679	0.00116	955	ko04626//Plant-pathogen interaction	-	-	-	gi|947116302|gb|KRH64604.1|/1.88213e-99/hypothetical protein GLYMA_04G245000 [Glycine max]
Glyma.05G157000	-	-	-	-	-	-	-	-	2.69023	0.00092	1648	ko04650//Natural killer cell mediated cytotoxicity;ko04626//Plant-pathogen interaction	-	GO:0003677//DNA binding;GO:0001071//nucleic acid binding transcription factor activity;GO:0046983//protein dimerization activity	GO:0006351//transcription, DNA-templated	gi|351727477|ref|NP_001237162.1|/8.92484e-99/bZIP transcription factor bZIP111 [Glycine max]
Glyma.17G018800	-	-	-	-	-	-	-	-	2.67929	3.42E-06	2593	ko04650//Natural killer cell mediated cytotoxicity;ko04075//Plant hormone signal transduction;ko04626//Plant-pathogen interaction	-	GO:0004702//receptor signaling protein serine/threonine kinase activity;GO:0032550	GO:0035556//intracellular signal transduction;GO:0006796//phosphate-containing compound metabolic process;GO:0006464//cellular protein modification process	gi|947052688|gb|KRH02141.1|;gi|947052687|gb|KRH02140.1|/0;0/hypothetical protein GLYMA_17G018800 [Glycine max];hypothetical protein GLYMA_17G018800 [Glycine max]
Glyma.06G068100	-	-	-	-	-	-	-	-	2.6787	0.00064	3397	ko04075//Plant hormone signal transduction;ko04626//Plant-pathogen interaction	-	GO:0004672//protein kinase activity;GO:0032550	GO:0006796//phosphate-containing compound metabolic process;GO:0006464//cellular protein modification process	gi|356518250|ref|XP_003527792.1|/0/PREDICTED: G-type lectin S-receptor-like serine/threonine-protein kinase SD2-5 [Glycine max]
Glyma.12G133700	-	-	-	-	-	-	-	-	2.6664	1.69E-06	2444	ko04626//Plant-pathogen interaction	-	-	-	gi|356545139|ref|XP_003541002.1|/0/PREDICTED: cyclic nucleotide-gated ion channel 4-like isoform X1 [Glycine max]
Glyma.17G167600	-	-	-	-	-	-	1.08213	0.00056	2.65017	0.00021	2795	ko04075//Plant hormone signal transduction;ko04626//Plant-pathogen interaction	GO:0016020//membrane	GO:0016301//kinase activity	GO:0044237//cellular metabolic process	gi|356563574|ref|XP_003550036.1|/0/PREDICTED: probable LRR receptor-like serine/threonine-protein kinase At4g37250 [Glycine max]
Glyma.13G033500	-	-	-	-	-	-	-	-	2.57301	0.00362	2293	ko04075//Plant hormone signal transduction;ko04626//Plant-pathogen interaction	-	GO:0001871;GO:0004672//protein kinase activity;GO:0032550	GO:0006796//phosphate-containing compound metabolic process;GO:0006464//cellular protein modification process	gi|955361655|ref|XP_014621916.1|;gi|571497140|ref|XP_006593813.1|/0;0/PREDICTED: probable receptor-like protein kinase At1g67000 isoform X2 [Glycine max];PREDICTED: probable receptor-like protein kinase At1g67000 isoform X1 [Glycine max]

20 × 20

Glyma.07G080700	-	-	-	-	-	-	-	-	2.5727	0.00436	2840	ko04626//Plant-pathogen interaction	-	GO:0004672//protein kinase activity;GO:0032550	GO:0006796//phosphate-containing compound metabolic process;GO:0006464//cellular protein modification process;GO:0008037//cell recognition	gi|571465418|ref|XP_003529974.2|/0/PREDICTED: putative receptor protein kinase ZmPK1 [Glycine max]
Glyma.03G044000	-	-	-	-	-	-	-	-	2.56707	0.0004	3606	ko00230//Purine metabolism;ko00240//Pyrimidine metabolism;ko01100//Metabolic pathways;ko03020//RNA polymerase;ko04626//Plant-pathogen interaction	-	-	-	gi|947117295|gb|KRH65544.1|/0/hypothetical protein GLYMA_03G044000 [Glycine max]
Glyma.06G275000	-	-	-	-	-	-	-	-	2.55626	0.00041	1599	ko04626//Plant-pathogen interaction	-	-	-	gi|947107330|gb|KRH55713.1|/0/hypothetical protein GLYMA_06G275000 [Glycine max]
Glyma.14G062100	-	-	-	-	-	-	-	-	2.5506	0.00265	2448	ko04075//Plant hormone signal transduction;ko04626//Plant-pathogen interaction	GO:0031461//cullin-RING ubiquitin ligase complex	-	GO:0006952//defense response;GO:0001101//response to acid chemical;GO:0043161//proteasome-mediated ubiquitin-dependent protein catabolic process;GO:0009620//response to fungus;GO:0009908//flower development;GO:0009617//response to bacterium;GO:0009755//hormone-mediated signaling pathway;GO:0009639//response to red or far red light	gi|947065850|gb|KRH14993.1|;gi|947065849|gb|KRH14992.1|/0;0/hypothetical protein GLYMA_14G062100 [Glycine max];hypothetical protein GLYMA_14G062100 [Glycine max]
Glyma.07G095000	-	-	-	-	-	-	-	-	2.53735	0.00062	2645	ko04075//Plant hormone signal transduction;ko04626//Plant-pathogen interaction	-	-	-	gi|571465619|ref|XP_006583417.1|/0/PREDICTED: probable receptor-like protein kinase At1g67000 isoform X2 [Glycine max]
Glyma.13G128200	-	-	-	-	-	-	-	-	2.53242	0.00081	3095	ko04626//Plant-pathogen interaction	-	GO:0004672//protein kinase activity;GO:0032550	GO:0006464//cellular protein modification process	gi|947070756|gb|KRH19647.1|;gi|947070757|gb|KRH19648.1|;gi|571497907|ref|XP_003541379.2|/0;0;0/hypothetical protein GLYMA_13G128200 [Glycine max];hypothetical protein GLYMA_13G128200 [Glycine max];PREDICTED: receptor-like serine/threonine-protein kinase ALE2 isoform X2 [Glycine max]
Glyma.02G129300	-	-	-	-	-	-	-	-	2.52908	9.51E-07	3294	ko04626//Plant-pathogen interaction	-	GO:0004672//protein kinase activity;GO:0032550	GO:0006464//cellular protein modification process	gi|955307115|ref|XP_014622348.1|/0/PREDICTED: proline-rich receptor-like protein kinase PERK9 [Glycine max]
Glyma.10G142600	-	-	-	-	-	-	-	-	2.52666	1.90E-06	3434	ko04075//Plant hormone signal transduction;ko04712//Circadian rhythm - plant;ko04626//Plant-pathogen interaction	-	-	-	gi|947085014|gb|KRH33735.1|;gi|918463621|gb|ALA09155.1|/0;0/hypothetical protein GLYMA_10G142600 [Glycine max];bHLH transcription factor, partial [Glycine max]
Glyma.03G043000	-1.3551	0.000207	-	-	-	-	-	-	2.5216	0.0037	3748	ko00230//Purine metabolism;ko00240//Pyrimidine metabolism;ko01100//Metabolic	-	-	-	gi|955310860|ref|XP_014628983.1|/0/PREDICTED: putative disease resistance protein At3g14460 [Glycine max]

												pathways;ko03020//RNA polymerase;ko04626//Plant-pathogen interaction				
Glyma.01G093100	-1.0863	0.005497	-	-	-	-	-	-	2.5204	3.80E-07	2915	ko04626//Plant-pathogen interaction	-	GO:0004672//protein kinase activity;GO:0032550	GO:0006464//cellular protein modification process	gi|955302504|ref|XP_003516269.2|/0/PREDICTED: proline-rich receptor-like protein kinase PERK10, partial [Glycine max]
Glyma.11G020700	-	-	-	-	-	-	-	-	2.51182	0.00031	4134	ko04626//Plant-pathogen interaction	GO:0031224//intrinsic component of membrane	GO:0046872//metal ion binding;GO:0016651//oxidoreductase activity, acting on NAD(P)H;GO:0016209//antioxidant activity	GO:0044710	gi|947079092|gb|KRH27881.1|;gi|356539557|ref|XP_003538264.1|/0;0/hypothetical protein GLYMA_11G020700 [Glycine max];PREDICTED: respiratory burst oxidase homolog protein A [Glycine max]
Glyma.04G076200	-	-	-	-	-	-	-	-	2.50612	0.0009	1659	ko04626//Plant-pathogen interaction	-	GO:0005488	GO:0050896//response to stimulus	gi|151934217|gb|ABS18446.1|/1.29563e-158/WRKY50 [Glycine max]
Glyma.08G284100	-	-	-	-	-	-	-	-	2.49575	0.00626	2697	ko04075//Plant hormone signal transduction;ko04626//Plant-pathogen interaction	-	GO:0004672//protein kinase activity;GO:0032550	GO:0016310//phosphorylation;GO:0006464//cellular protein modification process	gi|571468833|ref|XP_006584462.1|/0/PREDICTED: protein kinase family protein isoform X1 [Glycine max]
Glyma.13G341500	-	-	-	-	-	-	-	-	2.49335	6.07E-06	2209	ko04626//Plant-pathogen interaction	-	-	-	gi|359806061|ref|NP_001240925.1|/0/DNA-damage-repair/toleration protein DRT100-like precursor [Glycine max]
Glyma.13G283600	-	-	-	-	-4.0713	3.79E-06	-	-	2.49105	0.0005	1652	ko04070//Phosphatidylinositol signaling system;ko04626//Plant-pathogen interaction	-	GO:0046872//metal ion binding	GO:0009725//response to hormone;GO:0009628//response to abiotic stimulus	gi|351727745|ref|NP_001238195.1|/1.28283e-78/uncharacterized protein LOC100306396 [Glycine max]
Glyma.03G054800	-	-	-	-	-	-	-	-	2.46534	0.00109	3125	ko04626//Plant-pathogen interaction	-	-	-	gi|947117434|gb|KRH65683.1|/0/hypothetical protein GLYMA_03G054800 [Glycine max]
Glyma.17G209000	-	-	-	-	-	-	-	-	2.45987	0.00299	1364	ko04075//Plant hormone signal transduction;ko04626//Plant-pathogen interaction	-	GO:0016491//oxidoreductase activity	-	gi|571538353|ref|XP_006601141.1|/1.5634e-173/PREDICTED: transcription factor MYC2-like [Glycine max]
Glyma.17G118300	-	-	-	-	-	-	-	-	2.45514	1.78E-05	2965	ko04626//Plant-pathogen interaction	GO:0031224//intrinsic component of membrane	GO:0005249//voltage-gated potassium channel activity;GO:0005515//protein binding	GO:0010119//regulation of stomatal movement;GO:0048588//developmental cell growth;GO:0006970//response to osmotic stress;GO:0071805//potassium ion transmembrane transport	gi|947054292|gb|KRH03745.1|;gi|947054293|gb|KRH03746.1|/0;0/hypothetical protein GLYMA_17G118300 [Glycine max];hypothetical protein GLYMA_17G118300 [Glycine max]
Glyma.13G032100	-	-	-	-	-	-	-	-	2.45312	0.00093	2332	ko04626//Plant-pathogen interaction	-	GO:0004672//protein kinase activity;GO:0032550	GO:0006464//cellular protein modification process	gi|356547489|ref|XP_003542144.1|/0/PREDICTED: probable receptor-like protein kinase At1g11050 [Glycine max]
Glyma.03G043600	-	-	-	-	-	-	-	-	2.45282	0.00876	3606	ko00230//Purine metabolism;ko00240//Pyrimidine metabolism;ko01100//Metabolic pathways;ko03020//RNA polymerase;ko04626//Plant-pathogen interaction	-	-	-	gi|947117290|gb|KRH65539.1|/0/hypothetical protein GLYMA_03G043600 [Glycine max]

20 × 20

Glyma.18G283200	-	-	-	-	-	-	-	-	2.45281	0.00037	2511	ko04626//Plant-pathogen interaction	-	-	-	gi|947052005|gb|KRH01534.1|/0/hypothetical protein GLYMA_18G283200 [Glycine max]
Glyma.12G198800	-	-	-	-	-	-	-	-	2.43841	0.00664	2355	ko04075//Plant hormone signal transduction;ko04626//Plant-pathogen interaction	-	GO:0004672//protein kinase activity;GO:0032550	GO:0006796//phosphate-containing compound metabolic process;GO:0006464//cellular protein modification process;GO:0008037//cell recognition	gi|947078031|gb|KRH26871.1|/0/hypothetical protein GLYMA_12G198800 [Glycine max]
Glyma.12G236500	-	-	-	-	-	-	-	-	2.42635	0.01014	3554	ko04626//Plant-pathogen interaction	-	-	-	gi|571494846|ref|XP_006592960.1|;gi|571494848|ref|XP_006592961.1|/0;0/PREDICTED: probable disease resistance protein At1g61300 isoform X1 [Glycine max];PREDICTED: probable disease resistance protein At1g61300 isoform X2 [Glycine max]
Glyma.07G096300	-	-	-	-	-	-	-	-	2.42266	0.01016	2095	ko04626//Plant-pathogen interaction	-	GO:0004672//protein kinase activity;GO:0032550	GO:0006464//cellular protein modification process	gi|947100059|gb|KRH48551.1|/0/hypothetical protein GLYMA_07G0963002, partial [Glycine max]
Glyma.07G078100	-	-	-	-	-	-	-	-	2.40109	0.00924	4442	ko04626//Plant-pathogen interaction	-	-	-	gi|571465379|ref|XP_006583346.1|/0/PREDICTED: uncharacterized protein LOC100797869 isoform X2 [Glycine max]
Glyma.02G115200	-	-	-	-	-	-	-	-	2.40077	0.00017	1754	ko04626//Plant-pathogen interaction	-	GO:0001071//nucleic acid binding transcription factor activity;GO:0003677//DNA binding	GO:0006970//response to osmotic stress;GO:0006351//transcription, DNA-templated	gi|571439709|ref|XP_006574933.1|/0/PREDICTED: transcription factor [Glycine max]
Glyma.02G075600	-	-	-	-	-	-	-	-	2.39931	0.00061	2511	ko04075//Plant hormone signal transduction;ko04626//Plant-pathogen interaction	-	GO:0004672//protein kinase activity;GO:0032550	GO:0006796//phosphate-containing compound metabolic process;GO:0006464//cellular protein modification process;GO:0008037//cell recognition	gi|356502317|ref|XP_003519966.1|/0/PREDICTED: G-type lectin S-receptor-like serine/threonine-protein kinase At1g34300 [Glycine max]
Glyma.09G071600	-	-	-	-	-	-	-	-	2.38848	9.38E-05	1296	ko04075//Plant hormone signal transduction;ko04626//Plant-pathogen interaction	-	-	-	gi|947088863|gb|KRH37528.1|;gi|734350125|gb|KHN12298.1|;gi|947088864|gb|KRH37529.1|/5.27161e-146;1.94042e-171;2.31796e-142/hypothetical protein GLYMA_09G071600 [Glycine max];Protein TIFY 10A [Glycine soja] ;hypothetical protein GLYMA_09G071600 [Glycine max]
Glyma.02G245700	-	-	-	-	-	-	-	-	2.38419	0.00332	1530	ko04626//Plant-pathogen interaction	GO:0016020//membrane	GO:0046872//metal ion binding	GO:0009642//response to light intensity;GO:0006952//defense response;GO:0010038//response to metal ion;GO:0001101//response to acid chemical;GO:0000302//response to reactive oxygen species;GO:0009908//flower development;GO:0009725//response to hormone;GO:0048571//long-day photoperiodism;GO:0051171//regulation of nitrogen compound metabolic process	gi|356501039|ref|XP_003519336.1|/3.57948e-97/PREDICTED: probable calcium-binding protein CML18 [Glycine max]
Glyma.10G253000	-	-	-	-	-	-	-	-	2.33106	2.41E-05	2565	ko04626//Plant-pathogen interaction	-	GO:0004672//protein kinase activity;GO:0032550	GO:0006796//phosphate-containing compound metabolic process;GO:0006464//cellular protein modification process	gi|955347300|ref|XP_014618865.1|/0/PREDICTED: cysteine-rich receptor-like protein kinase 10 isoform X1 [Glycine max]

20 × 20

Glyma.18G050700	-	-	-	-	-	-	-	-	2.32729	0.00104	3603	ko04626//Plant-pathogen interaction	GO:0031224//intrinsic component of membrane	GO:0004672//protein kinase activity;GO:0032550	GO:0006796//phosphate-containing compound metabolic process;GO:0006464//cellular protein modification process	gi|356569432|ref|XP_003552905.1|/0/PREDICTED: probable LRR receptor-like serine/threonine-protein kinase At1g06840 [Glycine max]
Glyma.11G077300	-	-	-	-	-	-	-	-	2.31765	8.47E-05	2362	ko04626//Plant-pathogen interaction	-	GO:0046872//metal ion binding;GO:0004672//protein kinase activity;GO:0032550	GO:0006796//phosphate-containing compound metabolic process;GO:0006464//cellular protein modification process	gi|947080010|gb|KRH28799.1|;gi|356540813|ref|XP_003538879.1|/0;0/hypothetical protein GLYMA_11G077300 [Glycine max];PREDICTED: calcium-dependent protein kinase 28-like [Glycine max]
Glyma.15G179600	-	-	-	-	-1.9649	0.00127	-	-	2.30492	1.76E-05	1680	ko04075//Plant hormone signal transduction;ko04626//Plant-pathogen interaction	-	-	-	gi|947063310|gb|KRH12571.1|;gi|947063309|gb|KRH12570.1|;gi|734424752|gb|KHN42787.1|/7.42797e-148;1.06902e-172;0/hypothetical protein GLYMA_15G179600 [Glycine max];hypothetical protein GLYMA_15G179600 [Glycine max];Protein TIFY 10A [Glycine soja]
Glyma.07G063300	-2.2096	8.89E-20	-	-	-	-	-	-	2.28114	7.66E-06	6579	ko04626//Plant-pathogen interaction	-	GO:0036094//small molecule binding;GO:0097159//organic cyclic compound binding;GO:1901363	-	gi|947099527|gb|KRH48019.1|/0/hypothetical protein GLYMA_07G0633001, partial [Glycine max]
Glyma.13G244200	-	-	-	-	-	-	-	-	2.27949	0.00369	3079	ko04626//Plant-pathogen interaction	-	GO:0032550;GO:0016301//kinase activity	GO:0048827//phyllome development;GO:0009913//epidermal cell differentiation;GO:0006464//cellular protein modification process;GO:0006796//phosphate-containing compound metabolic process;GO:0001708//cell fate specification;GO:0009653//anatomical structure morphogenesis;GO:0003006//developmental process involved in reproduction	gi|571500270|ref|XP_006594611.1|/0/PREDICTED: protein STRUBBELIG-RECEPTOR FAMILY 3-like [Glycine max]
Glyma.08G271900	-	-	-	-	-	-	-	-	2.27188	2.14E-07	2704	ko04075//Plant hormone signal transduction;ko04626//Plant-pathogen interaction	-	-	-	gi|356526715|ref|XP_003531962.1|/0/PREDICTED: transcription factor MYC2 [Glycine max]
Glyma.08G303700	-	-	-	-	-	-	-	-	2.27129	0.00448	4369	ko00230//Purine metabolism;ko00240//Pyrimidine metabolism;ko01100//Metabolic pathways;ko03020//RNA polymerase;ko04626//Plant-pathogen interaction	-	-	-	gi|356524185|ref|XP_003530712.1|/0/PREDICTED: TMV resistance protein N-like isoform X2 [Glycine max]
Glyma.16G031600	-	-	-	-	-	-	-	-	2.24955	7.05E-05	8938	ko04626//Plant-pathogen interaction	-	-	-	gi|947057170|gb|KRH06576.1|;gi|947057169|gb|KRH06575.1|;gi|571525240|ref|XP_003548632.2|/0;0;0/hypothetical protein GLYMA_16G031600 [Glycine max];hypothetical protein GLYMA_16G031600 [Glycine max];PREDICTED: uncharacterized protein LOC100809946 isoform X2 [Glycine max]
Glyma.06G263900	-	-	-	-	-	-	-	-	2.24884	0.00448	4654	ko00230//Purine metabolism;ko00240//Pyrimidine	-	-	-	gi|955325893|ref|XP_014632232.1|/0/PREDICTED: disease resistance protein TAO1-like isoform X1 [Glycine max]

												metabolism;ko01100//Metabolic pathways;ko03020//RNA polymerase;ko04626//Plant-pathogen interaction				
Glyma.15G168200	-	-	-	-	-	-	-	-	2.24855	6.12E-07	1264	ko04626//Plant-pathogen interaction	-	-	-	gi|571519233|ref|XP_006597810.1|/1.06697e-143/PREDICTED: probable WRKY transcription factor 11 [Glycine max]
Glyma.02G037200	-1.7656	8.60E-08	-	-	-	-	-	-	2.23956	0.00123	2518	ko04075//Plant hormone signal transduction;ko04626//Plant-pathogen interaction	-	GO:0004672//protein kinase activity;GO:0032550	GO:0006468//protein phosphorylation	gi|734312199|gb|KHN00377.1|/0/Cysteine-rich receptor-like protein kinase 3 [Glycine soja]
Glyma.06G262700	-	-	-	-	-	-	-	-	2.21301	0.00241	3570	ko04626//Plant-pathogen interaction	-	GO:0004672//protein kinase activity	GO:0006796//phosphate-containing compound metabolic process	gi|955325868|ref|XP_014632226.1|;gi|955325866|ref|XP_014632225.1|/0;0/PREDICTED: G-type lectin S-receptor-like serine/threonine-protein kinase At4g27290 isoform X2 [Glycine max];PREDICTED: G-type lectin S-receptor-like serine/threonine-protein kinase At4g27290 isoform X1 [Glycine max]
Glyma.15G030000	-	-	-	-	-	-	-	-	2.20226	0.00691	2706	ko04626//Plant-pathogen interaction	GO:0016023//cytoplasmic membrane-bounded vesicle	GO:0032550;GO:0004672//protein kinase activity;GO:0046983//protein dimerization activity	GO:0048364//root development;GO:0006464//cellular protein modification process;GO:0008356//asymmetric cell division;GO:0009791//post-embryonic development;GO:0006796//phosphate-containing compound metabolic process;GO:0003006//developmental process involved in reproduction	gi|947060869|gb|KRH10130.1|/0/hypothetical protein GLYMA_15G030000 [Glycine max]
Glyma.U021800	-	-	-	-	-4.4647	4.84E-06	-	-	2.1905	9.34E-05	2236	ko04650//Natural killer cell mediated cytotoxicity;ko04626//Plant-pathogen interaction	-	GO:0004702//receptor signaling protein serine/threonine kinase activity;GO:0032550	GO:0045682//regulation of epidermis development;GO:0051707//response to other organism;GO:0009411//response to UV;GO:0009700//indole phytoalexin biosynthetic process;GO:0010243//response to organonitrogen compound;GO:0090567;GO:0080135//regulation of cellular response to stress;GO:0000187//activation of MAPK activity	gi|734378345|gb|KHN22030.1|;gi|947039232|gb|KRG89067.1|;gi|947039231|gb|KRG89066.1|/0;0;1.25479e-175/Mitogen-activated protein kinase 3 [Glycine soja] ;hypothetical protein GLYMA_U021800 [Glycine max];hypothetical protein GLYMA_U021800 [Glycine max]
Glyma.06G171700	-	-	-	-	-	-	-	-	2.17475	0.00317	3031	ko04626//Plant-pathogen interaction	-	GO:0016301//kinase activity;GO:0032550	GO:0006796//phosphate-containing compound metabolic process;GO:0006464//cellular protein modification process	gi|356518897|ref|XP_003528113.1|;gi|947105826|gb|KRH54209.1|/0;0/PREDICTED: probable inactive leucine-rich repeat receptor-like protein kinase At3g03770 isoform X1 [Glycine max];hypothetical protein GLYMA_06G171700 [Glycine max]
Glyma.12G217500	-	-	-	-	-	-	-	-	2.14759	1.86E-05	744	ko04070//Phosphatidylinositol signaling system;ko04626//Plant-pathogen interaction	-	GO:0046872//metal ion binding	GO:0009725//response to hormone;GO:0009628//response to abiotic stimulus	gi|351722363|ref|NP_001238521.1|/5.24239e-78/uncharacterized protein LOC100500465 [Glycine max]

Glyma.01G166100	-	-	-	-	-2.1941	0.00121	-	-	2.1374	4.51E-05	2572	ko04626//Plant-pathogen interaction	-	GO:0046872//metal ion binding;GO:0004672//protein kinase activity;GO:0032550	GO:0006796//phosphate-containing compound metabolic process;GO:0006464//cellular protein modification process	gi|947128803|gb|KRH76657.1|;gi|571435690|ref|XP_006573556.1|/0;0/hypothetical protein GLYMA_01G166100 [Glycine max];PREDICTED: calcium-dependent protein kinase 28-like [Glycine max]
Glyma.09G172500	-	-	-1.5482	0.000245	-	-	-	-	2.12165	0.0002	1444	ko04626//Plant-pathogen interaction	-	GO:0032550;GO:0004672//protein kinase activity;GO:0019887//protein kinase regulator activity	GO:0001934//positive regulation of protein phosphorylation;GO:0006351//transcription, DNA-templated;GO:0002239//response to oomycetes;GO:0009617//response to bacterium;GO:0009692;GO:0009700//indole phytoalexin biosynthetic process;GO:0006970//response to osmotic stress;GO:0000160//phosphorelay signal transduction system;GO:0060918//auxin transport;GO:0009627//systemic acquired resistance	gi|356531126|ref|XP_003534129.1|/0/PREDICTED: mitogen-activated protein kinase kinase 9 [Glycine max]
Glyma.17G218500	-	-	-	-	-	-	-	-	2.10669	9.07E-06	2394	ko04626//Plant-pathogen interaction	-	GO:0004672//protein kinase activity;GO:0032550	GO:0006796//phosphate-containing compound metabolic process;GO:0006464//cellular protein modification process	gi|356563936|ref|XP_003550213.1|/0/PREDICTED: L-type lectin-domain containing receptor kinase IX.1-like [Glycine max]
Glyma.03G047000	-	-	-	-	-	-	-	-	2.07611	0.00894	5472	ko00230//Purine metabolism;ko00240//Pyrimidine metabolism;ko01100//Metabolic pathways;ko03020//RNA polymerase;ko04626//Plant-pathogen interaction	-	-	-	gi|955310901|ref|XP_006576474.2|/0/PREDICTED: putative disease resistance RPP13-like protein 1 [Glycine max]
Glyma.16G136600	-2.1744	1.09E-08	-	-	-3.2628	4.42E-06	-	-	2.07556	0.00594	4635	ko00230//Purine metabolism;ko00240//Pyrimidine metabolism;ko01100//Metabolic pathways;ko03020//RNA polymerase;ko04626//Plant-pathogen interaction	-	-	-	gi|571528161|ref|XP_006599364.1|/0/PREDICTED: protein SUPPRESSOR OF npr1-1, CONSTITUTIVE 1 [Glycine max]
Glyma.09G191300	-	-	-	-	-	-	-	-	2.06922	8.50E-05	2501	ko04626//Plant-pathogen interaction	-	GO:0004672//protein kinase activity;GO:0032550;GO:0005198//structural molecule activity	GO:0006468//protein phosphorylation	gi|571475633|ref|XP_006586722.1|/0/PREDICTED: putative receptor protein kinase PERK1 isoform X1 [Glycine max]
Glyma.14G013300	-	-	-	-	-	-	-	-	2.06612	0.00472	1947	ko04075//Plant hormone signal transduction;ko04626//Plant-pathogen interaction	-	GO:0004672//protein kinase activity;GO:0032550	GO:0006796//phosphate-containing compound metabolic process;GO:0006464//cellular protein modification process	gi|571506326|ref|XP_006595690.1|/0/PREDICTED: probable L-type lectin-domain containing receptor kinase S.7 [Glycine max]
Glyma.03G220800	-	-	-	-	-	-	-	-	2.04961	0.00933	1466	ko04626//Plant-pathogen interaction	-	GO:0003677//DNA binding;GO:0001071//nucleic acid binding transcription factor activity	GO:0006351//transcription, DNA-templated	gi|918463819|gb|ALA09254.1|/0/WRKY transcription factor, partial [Glycine max]
Glyma.06G077400	-	-	-	-	-	-	-	-	2.04228	0.00024	1510	ko04626//Plant-pathogen interaction	-	GO:0001071//nucleic acid binding transcription factor	GO:0009617//response to bacterium;GO:0010243//response to organonitrogen	gi|351726405|ref|NP_001238661.1|/2.62285e-180/transcription factor [Glycine max]

20 × 20

														activity;GO:0003677//DNA binding;GO:0005515//protein binding	compound;GO:0006351//transcription, DNA-templated	
Glyma.20G211700	-	-	-	-	-	-	-	-	2.03992	0.01021	1000	ko04626//Plant-pathogen interaction	-	GO:0046872//metal ion binding	GO:0019932//second-messenger-mediated signaling	gi|734324268|gb|KHN05043.1|/2.79458e-103/Calmodulin-like protein 11 [Glycine soja]
Glyma.15G209300	-	-	-	-	-	-	2.01415	8.15E-07	2.03631	0.00073	1603	ko04075//Plant hormone signal transduction;ko04626//Plant-pathogen interaction	-	-	-	gi|358248196|ref|NP_001239837.1|/0/polygalacturonase inhibitor 1-like precursor [Glycine max]
Glyma.09G188600	-	-	-	-	-	-	-	-	2.012	3.63E-08	1689	ko04626//Plant-pathogen interaction	-	-	-	gi|947090594|gb|KRH39259.1|/0/hypothetical protein GLYMA_09G188600 [Glycine max]
Glyma.15G166800	-	-	-	-	-	-	-	-	1.98857	0.00304	1468	ko04075//Plant hormone signal transduction;ko04626//Plant-pathogen interaction	-	GO:0005515//protein binding	-	gi|734359974|gb|KHN15341.1|;gi|947063074|gb|KRH12335.1|/4.00382e-169;8.36369e-155/Transcription factor bHLH35 [Glycine soja] ;hypothetical protein GLYMA_15G166800 [Glycine max]
Glyma.13G351500	-	-	-	-	-	-	-	-	1.96196	0.0004	2061	ko04626//Plant-pathogen interaction	-	GO:0004672//protein kinase activity;GO:0032550	GO:0006468//protein phosphorylation	gi|947074444|gb|KRH23335.1|/0/hypothetical protein GLYMA_13G351500 [Glycine max]
Glyma.08G018900	-	-	-	-	-	-	-	-	1.94321	0.00026	4160	ko04626//Plant-pathogen interaction	GO:0031224//intrinsic component of membrane	GO:0046872//metal ion binding;GO:0016651//oxidoreductase activity, acting on NAD(P)H;GO:0016209//antioxidant activity	GO:0044710	gi|356528819|ref|XP_003532995.1|/0/PREDICTED: respiratory burst oxidase homolog protein A-like [Glycine max]
Glyma.06G077300	-	-	-	-	-	-	-	-	1.92549	0.00109	2515	ko04626//Plant-pathogen interaction	GO:0031224//intrinsic component of membrane	GO:0015075//ion transmembrane transporter activity	GO:0006811//ion transport;GO:0044763	gi|356518290|ref|XP_003527812.1|/0/PREDICTED: putative cyclic nucleotide-gated ion channel 18 [Glycine max]
Glyma.03G130800	-	-	-	-	-	-	-	-	1.9233	0.0013	1292	ko04626//Plant-pathogen interaction	GO:0016020//membrane	GO:0046872//metal ion binding	-	gi|356504734|ref|XP_003521150.1|/1.60881e-141/PREDICTED: probable calcium-binding protein CML36 [Glycine max]
Glyma.07G056400	-	-	-	-	-	-	-	-	1.9013	0.00691	2697	ko04626//Plant-pathogen interaction	GO:0031224//intrinsic component of membrane	GO:0005267//potassium channel activity	GO:0034220//ion transmembrane transport;GO:0030001//metal ion transport	gi|571465040|ref|XP_006583243.1|/0/PREDICTED: cyclic nucleotide-gated ion channel 1-like [Glycine max]
Glyma.05G232000	-	-	-	-	-	-	-	-	1.89839	2.35E-05	2142	ko04626//Plant-pathogen interaction	-	-	-	gi|734377070|gb|KHN21547.1|/0/Leucine-rich repeat extensin-like protein 5 [Glycine soja]
Glyma.09G199900	-	-	-	-	-	-	-	-	1.8369	0.00246	2123	ko04626//Plant-pathogen interaction	GO:0044444	GO:0032550;GO:0004672//protein kinase activity;GO:0019899//enzyme binding	GO:0006468//protein phosphorylation;GO:0009723//response to ethylene;GO:0009610//response to symbiotic fungus;GO:0045087//innate immune response	gi|356531361|ref|XP_003534246.1|/0/PREDICTED: receptor-like cytosolic serine/threonine-protein kinase RBK1 [Glycine max]
Glyma.07G088300	-1.037	2.95E-05	-	-	-	-	-	-	1.82871	2.70E-07	1467	ko04626//Plant-pathogen interaction	-	-	-	gi|947099935|gb|KRH48427.1|/0/hypothetical protein GLYMA_07G088300 [Glycine max]
Glyma.06G161200	-	-	-	-	-	-	-	-	1.82348	5.84E-07	1974	ko04626//Plant-pathogen interaction	-	GO:0032550;GO:0004672//protein kinase activity;GO:0046872//metal ion binding	GO:0006464//cellular protein modification process;GO:0009755//hormone-mediated signaling	gi|734341045|gb|KHN09683.1|/0/Calcium-dependent protein kinase SK5 [Glycine soja]

														pathway;GO:0006796//phosphate-containing compound metabolic process	
Glyma.08G290200	-	-	-	-	-	-	-	-	1.8229	0.00302	2192	ko04626//Plant-pathogen interaction	-	GO:0004672//protein kinase activity;GO:0032550	GO:0006796//phosphate-containing compound metabolic process;GO:0006464//cellular protein modification process	gi|734345203|gb|KHN10619.1|/0/Serine/threonine-protein kinase PBS1 [Glycine soja]
Glyma.15G065200	-	-	-	-	-	-	-	-	1.78024	1.08E-05	3227	ko04626//Plant-pathogen interaction	-	GO:0004672//protein kinase activity;GO:0032550	GO:0006796//phosphate-containing compound metabolic process;GO:0006464//cellular protein modification process;GO:0008037//cell recognition	gi|955372320|ref|XP_014623591.1|;gi|947061453|gb|KRH10714.1|/0;0/PREDICTED: G-type lectin S-receptor-like serine/threonine-protein kinase B120 [Glycine max];hypothetical protein GLYMA_15G065200 [Glycine max]
Glyma.01G196000	-	-	-	-	-	-	-	-	1.76396	0.00241	1433	ko04626//Plant-pathogen interaction	-	-	-	gi|947129312|gb|KRH77166.1|/1.33443e-161/hypothetical protein GLYMA_01G196000 [Glycine max]
Glyma.15G023200	-	-	-	-	-	-	-	-	1.74923	0.00147	3534	ko04626//Plant-pathogen interaction	-	GO:0004672//protein kinase activity;GO:0032550	GO:0006468//protein phosphorylation	gi|571514979|ref|XP_003546664.2|/0/PREDICTED: inactive protein kinase SELMODRAFT_444075-like [Glycine max]
Glyma.11G246200	-	-	-	-	-	-	-	-	1.71692	0.01048	3308	ko04075//Plant hormone signal transduction;ko04626//Plant-pathogen interaction	-	GO:0004672//protein kinase activity;GO:0032550	GO:0006796//phosphate-containing compound metabolic process;GO:0006464//cellular protein modification process	gi|955351777|ref|XP_014619757.1|;gi|356538111|ref|XP_003537548.1|;gi|947082611|gb|KRH31400.1|;gi|947082608|gb|KRH31397.1|;gi|947082610|gb|KRH31399.1|;gi|947082607|gb|KRH31396.1|/0;0;0;0;0;0/PREDICTED: probable LRR receptor-like serine/threonine-protein kinase At1g67720 isoform X2 [Glycine max];PREDICTED: probable LRR receptor-like serine/threonine-protein kinase At1g67720 isoform X1 [Glycine max];hypothetical protein GLYMA_11G246200 [Glycine max];hypothetical protein GLYMA_11G246200 [Glycine max];hypothetical protein GLYMA_11G246200 [Glycine max];hypothetical protein GLYMA_11G246200 [Glycine max]
Glyma.07G015300	-	-	-	-	-	-	-	-	1.71489	0.00012	2873	ko04626//Plant-pathogen interaction	-	GO:0004672//protein kinase activity;GO:0032550	GO:0006796//phosphate-containing compound metabolic process;GO:0006464//cellular protein modification process;GO:0044036//cell wall macromolecule metabolic process	gi|571464387|ref|XP_006583047.1|/0/PREDICTED: lysM domain receptor-like kinase 3 isoform X2 [Glycine max]
Glyma.16G157400	-	-	-	-	-	-	-	-	1.6993	0.00871	3424	ko04075//Plant hormone signal transduction;ko04626//Plant-pathogen interaction	-	GO:0004672//protein kinase activity;GO:0032550	GO:0006796//phosphate-containing compound metabolic process;GO:0006464//cellular protein modification process;GO:0008037//cell recognition	gi|947059159|gb|KRH08565.1|/0/hypothetical protein GLYMA_16G157400 [Glycine max]
Glyma.18G185400	-	-	-	-	-	-	-	-	1.69531	0.00323	2562	ko04075//Plant hormone signal transduction;ko04626//Plant-pathogen interaction	-	GO:0004672//protein kinase activity;GO:0032550	GO:0006468//protein phosphorylation	gi|734323185|gb|KHN04723.1|/0/L-type lectin-domain containing receptor kinase IV.1 [Glycine soja]
Glyma.16G085900	-	-	-	-	-	-	-	-	1.68542	4.80E-05	5021	ko00230//Purine metabolism;ko00240//Pyrimidine metabolism;ko01100//Metabolic pathways;ko03020//RNA polymerase;ko04626//P	-	-	GO:0050896//response to stimulus	gi|571526823|ref|XP_003548631.2|;gi|947057991|gb|KRH07397.1|;gi|947057995|gb|KRH07401.1|;gi|734363398|gb|KHN16501.1|/0;0;0;0/PREDICTED: TMV resistance protein N-like [Glycine max];hypothetical protein GLYMA_16G085900 [Glycine max];hypothetical protein GLYMA_16G085900 [Glycine max];TMV

												lant-pathogen interaction				resistance protein N [Glycine soja]
Glyma. 14G161 600	-	-	-	-	-	-	-	-	1.67161	0.00057	2733	ko04626//Plant-pathoge n interaction	GO:00312 24//intrinsi c component of membrane	GO:0005217//intracellular ligand-gated ion channel activity	GO:0044763;GO:0070838//di valent metal ion transport	gi|356551532|ref|XP_003544128.1|/0/PREDIC TED: cyclic nucleotide-gated ion channel 1-like [Glycine max]
Glyma. 08G060 500	-	-	-	-	-	-	-	-	1.65122	0.00695	3278	ko04626//Plant-pathoge n interaction	-	GO:0004672//protein kinase activity;GO:0032550	GO:0006796//phosphate-cont aining compound metabolic process;GO:0006464//cellular protein modification process;GO:0008037//cell recognition	gi|947093372|gb|KRH41957.1|;gi|947093371|g b|KRH41956.1|/0;0/hypothetical protein GLYMA_08G060500 [Glycine max];hypothetical protein GLYMA_08G060500 [Glycine max]
Glyma. 19G132 800	-	-	-	-	-	-	-	-	1.64426	0.00042	1542	ko04626//Plant-pathoge n interaction	GO:00160 20//membr ane	GO:0046872//metal ion binding	-	gi|356571963|ref|XP_003554140.1|/8.59197e-143/PREDICTED: probable calcium-binding protein CML36 [Glycine max]
Glyma. 08G259 000	-1.145	0.00099 2	-	-	-	-	-	-	1.62578	0.00246	2737	ko04626//Plant-pathoge n interaction	-	-	-	gi|571473175|ref|XP_006585845.1|/0/PREDIC TED: putative disease resistance RPP13-like protein 3 [Glycine max]
Glyma. U00830 0	1.95409	3.68E-0 9	1.94453	2.21E-0 5	-	-	-	-	1.62528	0.00067	3851	ko04626//Plant-pathoge n interaction	-	-	GO:0050896//response to stimulus	gi|947038217|gb|KRG88524.1|;gi|955395582|r ef|XP_014628748.1|/0;0/hypothetical protein GLYMA_U008300 [Glycine max];PREDICTED: protein SUPPRESSOR OF npr1-1, CONSTITUTIVE 1-like isoform X1 [Glycine max]
Glyma. 01G183 300	-	-	-	-	-1.9253	0.00106	-	-	1.61762	0.00318	2893	ko04626//Plant-pathoge n interaction	-	-	-	gi|571435938|ref|XP_006573620.1|/0/PREDIC TED: probable disease resistance protein At5g66900 [Glycine max]
Glyma. 17G250 800	-	-	-	-	-	-	-	-	1.61735	0.00638	4171	ko04626//Plant-pathoge n interaction	-	GO:0016301//kinase activity;GO:0032550	GO:0006464//cellular protein modification process	gi|356562351|ref|XP_003549435.1|/0/PREDIC TED: probable LRR receptor-like serine/threonine-protein kinase At2g16250 [Glycine max]
Glyma. 01G032 900	-	-	-	-	-	-	-	-	1.61077	0.00705	3261	ko04626//Plant-pathoge n interaction	-	GO:0036094//small molecule binding;GO:1901363;GO: 0097159//organic cyclic compound binding	-	gi|947126778|gb|KRH74632.1|/0/hypothetical protein GLYMA_01G032900 [Glycine max]
Glyma. 03G138 000	-	-	-	-	-	-	-	-	1.60409	8.51E-0 5	2274	ko04626//Plant-pathoge n interaction	-	GO:0046872//metal ion binding;GO:0004674//prot ein serine/threonine kinase activity;GO:0032550	GO:0006796//phosphate-cont aining compound metabolic process;GO:0006464//cellular protein modification process	gi|356504799|ref|XP_003521182.1|;gi|947118 710|gb|KRH66959.1|/0;0/PREDICTED: calcium-dependent protein kinase 32-like [Glycine max];hypothetical protein GLYMA_03G138000 [Glycine max]
Glyma. 08G223 400	-	-	-	-	-	-	-	-	1.58341	0.00227	1927	ko04626//Plant-pathoge n interaction	GO:00444 24	GO:0032550;GO:0004672 //protein kinase activity	GO:2000026//regulation of multicellular organismal development;GO:0006464//ce llular protein modification process;GO:0006796//phosph ate-containing compound metabolic process;GO:0090567	gi|356526330|ref|XP_003531771.1|/0/PREDIC TED: mitogen-activated protein kinase kinase 5-like [Glycine max]
Glyma. 17G166 200	-	-	-	-	-	-	-	-	1.57639	0.00599	3103	ko04626//Plant-pathoge n interaction	-	GO:0004672//protein kinase activity;GO:0032550	GO:0006796//phosphate-cont aining compound metabolic process;GO:0006464//cellular protein modification process	gi|734391140|gb|KHN27061.1|/0/Putative receptor-like protein kinase [Glycine soja]
Glyma. 01G189 100	-	-	-	-	-	-	-	-	1.56901	0.00711	1870	ko04626//Plant-pathoge n interaction	-	-	-	gi|571436024|ref|XP_006573647.1|;gi|356496 927|ref|XP_003517316.1|/1.38499e-170;5.940 37e-177/PREDICTED: probable WRKY transcription factor 15 isoform X2 [Glycine max];PREDICTED: probable WRKY transcription factor 15 isoform X1 [Glycine

																max]
Glyma.03G043200	-	-	-	-	-	-	-	-	1.5684	0.00751	3597	ko00230//Purine metabolism;ko00240//Pyrimidine metabolism;ko01100//Metabolic pathways;ko03020//RNA polymerase;ko04626//Plant-pathogen interaction	-	-	-	gi|955310862|ref|XP_006577438.2|/0/PREDICTED: putative disease resistance protein At3g14460 [Glycine max]
Glyma.12G053400	-	-	-	-	-	-	-	-	1.56451	0.0046	2238	ko04626//Plant-pathogen interaction	-	GO:0046872//metal ion binding;GO:0004674//protein serine/threonine kinase activity;GO:0032550	GO:0006796//phosphate-containing compound metabolic process;GO:0006464//cellular protein modification process	gi|356544533|ref|XP_003540704.1|/0/PREDICTED: calcium-dependent protein kinase 24-like [Glycine max]
Glyma.10G023100	-	-	-	-	-	-	-	-	1.5572	0.00031	1206	ko04626//Plant-pathogen interaction	-	GO:0016628//oxidoreductase activity, acting on the CH-CH group of donors, NAD or NADP as acceptor;GO:0016772//transferase activity, transferring phosphorus-containing groups	GO:0044710;GO:0006796//phosphate-containing compound metabolic process	gi|356536745|ref|XP_003536896.1|/0/PREDICTED: probably inactive leucine-rich repeat receptor-like protein kinase IMK2 [Glycine max]
Glyma.08G297400	-	-	-	-	-	-	-	-	1.52401	0.00032	3139	ko04626//Plant-pathogen interaction	-	GO:0016301//kinase activity;GO:0032550	GO:0006464//cellular protein modification process	gi|955337649|ref|XP_014634833.1|/0/PREDICTED: probable LRR receptor-like serine/threonine-protein kinase At1g14390 [Glycine max]
Glyma.12G135600	-	-	-	-	-	-	-	-	1.52116	3.34E-05	4421	ko04626//Plant-pathogen interaction	-	-	-	gi|734430980|gb|KHN45708.1|/0/TMV resistance protein N [Glycine soja]
Glyma.14G071400	-	-	-	-	-	-	-	-	1.48845	0.00331	1733	ko04650//Natural killer cell mediated cytotoxicity;ko04626//Plant-pathogen interaction	-	-	-	gi|356551950|ref|XP_003544335.1|/3.19685e-83/PREDICTED: ocs element-binding factor 1 [Glycine max]
Glyma.06G036400	-	-	-	-	-	-	-	-	1.4688	0.00658	2659	ko04626//Plant-pathogen interaction	-	GO:0016301//kinase activity;GO:0032550;GO:0036094//small molecule binding;GO:1901363;GO:0097159//organic cyclic compound binding	GO:0006464//cellular protein modification process;GO:0044237//cellular metabolic process	gi|947103557|gb|KRH51940.1|;gi|571458793|ref|XP_006581230.1|/0;0/hypothetical protein GLYMA_06G036400 [Glycine max];PREDICTED: mitogen-activated protein kinase kinase kinase YODA-like [Glycine max]
Glyma.07G105700	-	-	-	-	-	-	1.54223	5.88E-05	1.44889	0.00611	1376	ko04626//Plant-pathogen interaction	-	GO:0032550;GO:0004672//protein kinase activity;GO:0019887//protein kinase regulator activity	GO:0001934//positive regulation of protein phosphorylation;GO:0006351//transcription, DNA-templated;GO:0002239//response to oomycetes;GO:0009617//response to bacterium;GO:0009692;GO:0009700//indole phytoalexin biosynthetic process;GO:0006970//response to osmotic stress;GO:0000160//phosphorelay signal transduction system;GO:0060918//auxin transport;GO:0009627//systemic acquired resistance	gi|356520673|ref|XP_003528985.1|/0/PREDICTED: mitogen-activated protein kinase kinase 9 [Glycine max]

<table>
<tr><td>Glyma. 03G054 100</td><td>-</td><td>-</td><td>-</td><td>-</td><td>-</td><td>-</td><td>-</td><td>-</td><td>1.44814</td><td>0.00481</td><td>3425</td><td>ko00230//Purine metabolism;ko00240//Pyrimidine metabolism;ko01100//Metabolic pathways;ko03020//RNA polymerase;ko04626//Plant-pathogen interaction</td><td>-</td><td>GO:0036094//small molecule binding;GO:1901363;GO:0097159//organic cyclic compound binding</td><td>-</td><td>gi|947117426|gb|KRH65675.1|;gi|947117424|gb|KRH65673.1|;gi|947117423|gb|KRH65672.1|;gi|947117427|gb|KRH65676.1|;gi|947117425|gb|KRH65674.1|/4.13447e-155;0;0;0;0/hypothetical protein GLYMA_03G054100 [Glycine max];hypothetical protein GLYMA_03G054100 [Glycine max];hypothetical protein GLYMA_03G054100 [Glycine max];hypothetical protein GLYMA_03G054100 [Glycine max];hypothetical protein GLYMA_03G054100 [Glycine max]</td></tr>
<tr><td>Glyma. 10G009 100</td><td>-</td><td>-</td><td>-</td><td>-</td><td>-</td><td>-</td><td>-</td><td>-</td><td>1.44445</td><td>0.00037</td><td>1920</td><td>ko04626//Plant-pathogen interaction</td><td>-</td><td>GO:0032550;GO:0004702//receptor signaling protein serine/threonine kinase activity</td><td>GO:0035556//intracellular signal transduction;GO:0032147//activation of protein kinase activity</td><td>gi|359811321|ref|NP_001241285.1|/0/pto-interacting protein 1-like [Glycine max]</td></tr>
<tr><td>Glyma. 06G319 700</td><td>-</td><td>-</td><td>-</td><td>-</td><td>-</td><td>-</td><td>-</td><td>-</td><td>1.42848</td><td>0.00687</td><td>2068</td><td>ko04626//Plant-pathogen interaction</td><td>GO:0005618//cell wall;GO:0009536//plastid</td><td>GO:0016491//oxidoreductase activity</td><td>GO:0009628//response to abiotic stimulus;GO:0008152//metabolic process</td><td>gi|351724553|ref|NP_001235526.1|/0/disease resistance protein/LRR protein-related protein precursor [Glycine max]</td></tr>
<tr><td>Glyma. 03G043 500</td><td>-</td><td>-</td><td>-</td><td>-</td><td>-</td><td>-</td><td>-</td><td>-</td><td>1.40762</td><td>0.00445</td><td>5300</td><td>ko00230//Purine metabolism;ko00240//Pyrimidine metabolism;ko01100//Metabolic pathways;ko03020//RNA polymerase;ko04626//Plant-pathogen interaction</td><td>-</td><td>-</td><td>-</td><td>gi|955310855|ref|XP_014628981.1|/0/PREDICTED: putative disease resistance protein At3g14460 [Glycine max]</td></tr>
<tr><td>Glyma. 06G153 200</td><td>-</td><td>-</td><td>-</td><td>-</td><td>-</td><td>-</td><td>-</td><td>-</td><td>1.40116</td><td>3.16E-05</td><td>2548</td><td>ko04626//Plant-pathogen interaction</td><td>-</td><td>GO:0016301//kinase activity;GO:0032550</td><td>GO:0006796//phosphate-containing compound metabolic process;GO:0006464//cellular protein modification process</td><td>gi|356516360|ref|XP_003526863.1|/0/PREDICTED: mitogen-activated protein kinase kinase kinase YODA-like [Glycine max]</td></tr>
<tr><td>Glyma. 02G000 400</td><td>-</td><td>-</td><td>-</td><td>-</td><td>-</td><td>-</td><td>-</td><td>-</td><td>1.39581</td><td>0.00149</td><td>2481</td><td>ko04626//Plant-pathogen interaction</td><td>-</td><td>GO:0004672//protein kinase activity;GO:0032550</td><td>GO:0016310//phosphorylation;GO:0006464//cellular protein modification process;GO:0044036//cell wall macromolecule metabolic process</td><td>gi|356499893|ref|XP_003518770.1|/0/PREDICTED: lysM domain receptor-like kinase 4 [Glycine max]</td></tr>
<tr><td>Glyma. 09G259 100</td><td>-</td><td>-</td><td>-</td><td>-</td><td>-</td><td>-</td><td>-</td><td>-</td><td>1.37109</td><td>0.00132</td><td>4213</td><td>ko04626//Plant-pathogen interaction</td><td>GO:0044424</td><td>GO:0004672//protein kinase activity;GO:0019787//ubiquitin-like protein transferase activity;GO:0032550</td><td>GO:0032446//protein modification by small protein conjugation</td><td>gi|571479402|ref|XP_006587850.1|;gi|356531997|ref|XP_003534561.1|/0;0/PREDICTED: U-box domain-containing protein 33-like isoform X2 [Glycine max];PREDICTED: U-box domain-containing protein 33-like isoform X1 [Glycine max]</td></tr>
<tr><td>Glyma. 04G220 400</td><td>-</td><td>-</td><td>-</td><td>-</td><td>-</td><td>-</td><td>-</td><td>-</td><td>1.36427</td><td>0.00919</td><td>3932</td><td>ko04626//Plant-pathogen interaction</td><td>GO:0031224//intrinsic component of membrane;GO:0044444</td><td>GO:0016301//kinase activity;GO:0032550</td><td>GO:0006796//phosphate-containing compound metabolic process;GO:0006464//cellular protein modification process</td><td>gi|356509054|ref|XP_003523267.1|/0/PREDICTED: probable inactive receptor kinase At5g10020 [Glycine max]</td></tr>
<tr><td>Glyma. 01G218 800</td><td>-</td><td>-</td><td>-</td><td>-</td><td>-</td><td>-</td><td>-</td><td>-</td><td>1.36221</td><td>0.01007</td><td>3590</td><td>ko04075//Plant hormone signal transduction;ko04626//Plant-pathogen interaction</td><td>GO:0031224//intrinsic component of membrane</td><td>GO:0016301//kinase activity;GO:0032550</td><td>GO:0006464//cellular protein modification process</td><td>gi|947129672|gb|KRH77526.1|;gi|571436477|ref|XP_006573774.1|/0;0/hypothetical protein GLYMA_01G218800 [Glycine max];PREDICTED: probable LRR receptor-like serine/threonine-protein kinase At1g63430 [Glycine max]</td></tr>
</table>

Glyma.02G037100	-	-	-	-	-1.1594	0.00107	-	-	1.35489	0.00125	3167	ko04075//Plant hormone signal transduction;ko04626//Plant-pathogen interaction	GO:0005911//cell-cell junction;GO:0016020//membrane	GO:0004672//protein kinase activity;GO:0032550	GO:0006468//protein phosphorylation;GO:0000302//response to reactive oxygen species	gi|571437782|ref|XP_006574321.1|/0/PREDICTED: protein kinase family protein isoform X1 [Glycine max]
Glyma.06G267400	-	-	-	-	-	-	-	-	1.35229	0.00013	4506	ko04626//Plant-pathogen interaction	-	GO:0032550	GO:0050896//response to stimulus	gi|947107241|gb|KRH55624.1|;gi|571457908|ref|XP_006580944.1|/0;0/hypothetical protein GLYMA_06G267400 [Glycine max];PREDICTED: uncharacterized protein LOC100500528 isoform X1 [Glycine max]
Glyma.03G037000	-	-	-	-	-	-	-	-	1.33316	0.00315	4395	ko00230//Purine metabolism;ko00240//Pyrimidine metabolism;ko01100//Metabolic pathways;ko03020//RNA polymerase;ko04626//Plant-pathogen interaction	-	-	-	gi|356506465|ref|XP_003522002.1|/0/PREDICTED: putative disease resistance RPP13-like protein 1 [Glycine max]
Glyma.13G112000	-	-	-	-	-	-	-	-	1.31771	0.00145	2360	ko04075//Plant hormone signal transduction;ko04626//Plant-pathogen interaction	-	-	-	gi|918463851|gb|ALA09270.1|/0/ZIM transcription factor, partial [Glycine max]
Glyma.01G118800	-	-	-	-	-	-	-	-	1.31486	0.00236	2442	ko04626//Plant-pathogen interaction	-	-	-	gi|955302776|ref|XP_014630170.1|/0/PREDICTED: probable leucine-rich repeat receptor-like serine/threonine-protein kinase At3g14840 isoform X1 [Glycine max]
Glyma.14G074700	-	-	-	-	-	-	-	-	1.31383	0.00712	4087	ko04626//Plant-pathogen interaction	-	GO:0016301//kinase activity;GO:0032550	GO:0006464//cellular protein modification process	gi|356552103|ref|XP_003544410.1|/0/PREDICTED: probable LRR receptor-like serine/threonine-protein kinase At2g16250 [Glycine max]
Glyma.08G218600	-	-	-	-	-3.5617	4.69E-06	-	-	1.27268	0.00373	1828	ko04626//Plant-pathogen interaction	-	GO:0001071//nucleic acid binding transcription factor activity;GO:0003677//DNA binding	GO:0098542//defense response to other organism;GO:0006351//transcription, DNA-templated;GO:0010033//response to organic substance;GO:0050789//regulation of biological process;GO:0051707//response to other organism;GO:0009987//cellular process;GO:0065007//biological regulation;GO:0006952//defense response	gi|255637165|gb|ACU18913.1|;gi|947095978|gb|KRH44563.1|;gi|571468821|ref|XP_006584456.1|;gi|947095977|gb|KRH44562.1|/0;6.6146 4e-70;0;1.08096e-125/unknown [Glycine max];hypothetical protein GLYMA_08G218600 [Glycine max];PREDICTED: transcription factor isoform X1 [Glycine max];hypothetical protein GLYMA_08G218600 [Glycine max]
Glyma.06G145700	-	-	-	-	-	-	-	-	1.27244	0.0079	2947	ko04626//Plant-pathogen interaction	GO:0009526//plastid envelope	GO:0016740//transferase activity	-	gi|947105396|gb|KRH53779.1|/0/hypothetical protein GLYMA_06G145700 [Glycine max]
Glyma.11G054600	-	-	-	-	-	-	-	-	1.27213	0.00834	1836	ko04075//Plant hormone signal transduction;ko04712//Circadian rhythm - plant;ko04626//Plant-pathogen interaction	-	-	GO:0007275//multicellular organismal development;GO:0048608//reproductive structure development;GO:0009628//response to abiotic stimulus;GO:0009791//post-embryonic development	gi|734423171|gb|KHN42092.1|;gi|947079665|gb|KRH28454.1|;gi|947079664|gb|KRH28453.1|/0;6.4743e-165;0/Transcription factor SPATULA [Glycine soja];hypothetical protein GLYMA_11G0546001, partial [Glycine max];hypothetical protein GLYMA_11G0546001 [Glycine max]

20 × 20

Glyma.13G266300	-	-	-	-	-	-	-	-	1.26018	0.00725	3585	ko04075//Plant hormone signal transduction;ko04626//Plant-pathogen interaction	-	GO:0046914//transition metal ion binding;GO:0004672//protein kinase activity;GO:0032550	GO:0006796//phosphate-containing compound metabolic process;GO:0006464//cellular protein modification process	gi|947073017|gb|KRH21908.1|/0/hypothetical protein GLYMA_13G266300 [Glycine max]
Glyma.10G159500	-	-	-	-	-	-	-	-	1.22398	0.00048	5775	ko04626//Plant-pathogen interaction	-	-	-	gi|571483241|ref|XP_006589174.1|;gi|947085312|gb|KRH34033.1|;gi|571483243|ref|XP_006589175.1|/0;0;0/PREDICTED: protein MON2 homolog isoform X1 [Glycine max];hypothetical protein GLYMA_10G159500 [Glycine max];PREDICTED: protein MON2 homolog isoform X2 [Glycine max]
Glyma.15G001500	-	-	-	-	-	-	-	-	1.21865	0.00162	3906	ko04626//Plant-pathogen interaction	GO:0031224//intrinsic component of membrane	GO:0032550;GO:0004672//protein kinase activity	GO:0050896//response to stimulus;GO:0006464//cellular protein modification process;GO:0006796//phosphate-containing compound metabolic process	gi|571514319|ref|XP_003546285.2|/0/PREDICTED: receptor-like protein kinase [Glycine max]
Glyma.13G248800	-	-	-	-	-	-	-	-	1.1914	0.00564	3053	ko04626//Plant-pathogen interaction	-	GO:0004672//protein kinase activity;GO:0032550	GO:0006796//phosphate-containing compound metabolic process;GO:0006464//cellular protein modification process;GO:0008037//cell recognition	gi|571500423|ref|XP_006594639.1|/0/PREDICTED: G-type lectin S-receptor-like serine/threonine-protein kinase B120 isoform X1 [Glycine max]
Glyma.06G223800	-	-	-	-	-	-	-	-	1.18334	0.00204	2664	ko04626//Plant-pathogen interaction	GO:0031224//intrinsic component of membrane	GO:0005267//potassium channel activity	GO:0034220//ion transmembrane transport;GO:0030001//metal ion transport	gi|571461788|ref|XP_006582102.1|;gi|955325484|ref|XP_014632152.1|/0;0/PREDICTED: probable cyclic nucleotide-gated ion channel 17 isoform X1 [Glycine max];PREDICTED: probable cyclic nucleotide-gated ion channel 17 isoform X2 [Glycine max]
Glyma.05G034600	-	-	-	-	-	-	-	-	1.1776	0.00402	2410	ko04070//Phosphatidylinositol signaling system;ko04626//Plant-pathogen interaction	-	-	-	gi|571453593|ref|XP_006579554.1|/0/PREDICTED: calmodulin-binding protein 60 A-like isoform X1 [Glycine max]
Glyma.19G140800	-	-	-	-	-	-	-	-	1.17675	0.00579	2209	ko04626//Plant-pathogen interaction	-	GO:0046872//metal ion binding;GO:0004674//protein serine/threonine kinase activity;GO:0032550	GO:0006796//phosphate-containing compound metabolic process;GO:0006464//cellular protein modification process	gi|356572042|ref|XP_003554179.1|/0/PREDICTED: calcium-dependent protein kinase 32 [Glycine max]
Glyma.13G102000	-	-	-	-	-	-	-	-	1.16015	0.00302	1764	ko04626//Plant-pathogen interaction	-	GO:0003677//DNA binding;GO:0001071//nucleic acid binding transcription factor activity	GO:0006351//transcription, DNA-templated	gi|947070229|gb|KRH19120.1|/7.85339e-164/hypothetical protein GLYMA_13G102000 [Glycine max]
Glyma.05G010600	-	-	-	-	-	-	-	-	1.12633	0.00748	2947	ko04626//Plant-pathogen interaction	GO:0031224//intrinsic component of membrane	GO:0005249//voltage-gated potassium channel activity;GO:0005515//protein binding	GO:0010119//regulation of stomatal movement;GO:0048588//developmental cell growth;GO:0006970//response to osmotic stress;GO:0071805//potassium ion transmembrane transport	gi|571454296|ref|XP_003524528.2|/0/PREDICTED: potassium channel AKT1-like [Glycine max]
Glyma.11G232200	-	-	-	-	-	-	-	-	1.12323	0.00569	2463	ko04626//Plant-pathogen interaction	GO:0031224//intrinsic component of membrane	GO:0016301//kinase activity;GO:0032550	GO:0006796//phosphate-containing compound metabolic process;GO:0006464//cellular protein modification process	gi|356537999|ref|XP_003537493.1|/0/PREDICTED: probable inactive receptor-like protein kinase At3g56050 [Glycine max]
Glyma.02G008500	-	-	-	-	-	-	-	-	1.09708	0.00085	1853	ko04626//Plant-pathogen interaction	-	GO:0032550;GO:0004702//receptor signaling protein serine/threonine kinase activity	GO:0035556//intracellular signal transduction;GO:0032147//activation of protein kinase activity	gi|571438081|ref|XP_006574465.1|;gi|358248598|ref|NP_001239908.1|/0;0/PREDICTED: pto-interacting protein 1-like isoform X1 [Glycine max];pto-interacting protein 1-like [Glycine max]

Glyma.05G014600	-	-	-	-	-	-	-	-	1.0923	0.00905	1818	ko04626//Plant-pathogen interaction	-	-	-	gi|734359148|gb|KHN15161.1|/0/Piriformospora indica-insensitive protein 2 [Glycine soja]
Glyma.08G316500	-	-	-	-	-	-	-	-	1.07526	0.00663	2702	ko04626//Plant-pathogen interaction	-	GO:0046872//metal ion binding;GO:0004672//protein kinase activity;GO:0032550	GO:0006796//phosphate-containing compound metabolic process;GO:0006464//cellular protein modification process	gi|214011508|gb|ACJ61504.1|/0/calcium-dependent protein kinase [Glycine max]
Glyma.03G075300	-	-	-	-	-	-	-	-	1.07027	0.00772	5431	ko04626//Plant-pathogen interaction	-	GO:0032550	GO:0050896//response to stimulus	gi|571444675|ref|XP_006576576.1|/0/PREDICTED: TMV resistance protein N-like isoform X1 [Glycine max]
Glyma.09G215700	-	-	-	-	-	-	-	-	1.06993	0.00209	2750	ko04075//Plant hormone signal transduction;ko04626//Plant-pathogen interaction	GO:0031224//intrinsic component of membrane	GO:0032550;GO:0004713//protein tyrosine kinase activity	GO:0006468//protein phosphorylation;GO:0071555//cell wall organization;GO:0000902//cell morphogenesis	gi|358248000|ref|NP_001239788.1|;gi|734348233|gb|KHN11811.1|/0;0/LRR receptor-like serine/threonine-protein kinase FEI 1 precursor [Glycine max];LRR receptor-like serine/threonine-protein kinase FEI 1 [Glycine soja]
Glyma.07G151000	-	-	-	-	-	-	-	-	1.04984	0.00622	2685	ko04626//Plant-pathogen interaction	-	GO:0046872//metal ion binding;GO:0004674//protein serine/threonine kinase activity;GO:0032550	GO:0006796//phosphate-containing compound metabolic process;GO:0006464//cellular protein modification process	gi|356521026|ref|XP_003529159.1|/0/PREDICTED: calcium-dependent protein kinase 13 [Glycine max]
Glyma.08G265200	-	-	-	-	-	-	-	-	-1.0116	0.00326	1614	ko04626//Plant-pathogen interaction	GO:0044424	GO:0046872//metal ion binding	-	gi|571469172|ref|XP_006584629.1|/1.81094e-167/PREDICTED: uncharacterized protein LOC100820628 isoform X1 [Glycine max]
Glyma.18G166800	-	-	-	-	-	-	-	-	-1.0201	0.00267	1362	ko04626//Plant-pathogen interaction	-	-	-	gi|351723541|ref|NP_001235235.1|/0/RIN4c protein [Glycine max]
Glyma.17G154500	-	-	-	-	-	-	-	-	-1.0432	0.00577	1888	ko04626//Plant-pathogen interaction	-	-	-	gi|734314283|gb|KHN01686.1|/0/Leucine-rich repeat-containing protein 40 [Glycine soja]
Glyma.16G049400	-	-	-	-	-	-	-	-	-1.0439	0.00284	1935	ko04075//Plant hormone signal transduction;ko04626//Plant-pathogen interaction	-	-	-	gi|359806583|ref|NP_001241268.1|;gi|947057436|gb|KRH06842.1|/0;0/transcription factor ICE1-like [Glycine max];hypothetical protein GLYMA_16G049400 [Glycine max]
Glyma.20G222600	-	-	-	-	-	-	1.28669	0.00069	-1.0641	0.00474	1224	ko04626//Plant-pathogen interaction	-	GO:0032550;GO:0004672//protein kinase activity	GO:0009314//response to radiation;GO:0009267//cellular response to starvation;GO:0006464//cellular protein modification process;GO:0010243//response to organonitrogen compound;GO:0006796//phosphate-containing compound metabolic process	gi|351727763|ref|NP_001236660.1|/0/phosphoenolpyruvate carboxylase kinase [Glycine max]
Glyma.19G005700	-	-	-	-	-	-	1.83488	0.00051	-1.086	0.00784	2512	ko04075//Plant hormone signal transduction;ko04626//Plant-pathogen interaction	-	GO:0004672//protein kinase activity;GO:0032550	GO:0006464//cellular protein modification process	gi|356573321|ref|XP_003554810.1|/0/PREDICTED: cysteine-rich receptor-like protein kinase 42 [Glycine max]
Glyma.09G203000	-	-	-	-	-	-	-1.0423	0.00122	-1.2186	0.00052	1485	ko04075//Plant hormone signal transduction;ko04626//Plant-pathogen interaction	-	GO:0005515//protein binding	-	gi|571475524|ref|XP_006586670.1|;gi|947090855|gb|KRH39520.1|/0;0/PREDICTED: uncharacterized protein LOC100784145 isoform X1 [Glycine max];hypothetical protein GLYMA_09G203000 [Glycine max]
Glyma.02G100000	-	-	-	-	-	-	-	-	-1.2253	0.00325	2109	ko04626//Plant-pathogen interaction	-	GO:0004672//protein kinase activity;GO:0032550	GO:0006796//phosphate-containing compound metabolic process;GO:0006464//cellular protein modification process;GO:0010109//regulation of photosynthesis;GO:0048511//	gi|356502478|ref|XP_003520046.1|/0/PREDICTED: serine/threonine-protein kinase STN7, chloroplastic-like [Glycine max]

															rhythmic process	
Glyma. 14G219 700	-	-	-	-	-	-	-	-	-1.2448	0.00024	3030	ko04141//Protein processing in endoplasmic reticulum;ko04626//Plant-pathogen interaction	GO:0009536//plastid;GO:0044437;GO:0005911//cell-cell junction;GO:0005576//extracellular region;GO:0016020//membrane	GO:0032550;GO:0005515//protein binding	GO:0010038//response to metal ion;GO:0044267//cellular protein metabolic process;GO:0009933//meristem structural organization;GO:0010073//meristem maintenance;GO:0006970//response to osmotic stress;GO:0015031//protein transport	gi|947068301|gb|KRH17444.1|;gi|571512225|ref|XP_006596543.1|;gi|734418176|gb|KHN39421.1|/0;0;0/hypothetical protein GLYMA_14G219700 [Glycine max];PREDICTED: endoplasmin homolog isoform X2 [Glycine max];Endoplasmin like [Glycine soja]
Glyma. 12G219 000	-	-	-	-	-	-	-	-	-1.2588	0.00143	2275	ko04626//Plant-pathogen interaction	-	-	-	gi|356542246|ref|XP_003539580.1|/0/PREDICTED: cyclic nucleotide-gated ion channel 4-like [Glycine max]
Glyma. 14G040 600	-	-	-	-	-	-	-	-	-1.2752	0.00043	995	ko04070//Phosphatidylinositol signaling system;ko04626//Plant-pathogen interaction	GO:0044437;GO:0016020//membrane	GO:0046872//metal ion binding	GO:0019932//second-messenger-mediated signaling;GO:0006637//acyl-CoA metabolic process;GO:0009640//photomorphogenesis;GO:0009593//detection of chemical stimulus;GO:0009605//response to external stimulus	gi|413945839|gb|AFW78488.1|/1.43783e-104/calmodulin1 [Zea mays]
Glyma. 03G240 000	-	-	-	-	-	-	-	-	-1.2762	0.00036	2166	ko04075//Plant hormone signal transduction;ko04626//Plant-pathogen interaction	-	GO:0005515//protein binding	-	gi|947120341|gb|KRH68590.1|;gi|571447011|ref|XP_006577251.1|/0;0/hypothetical protein GLYMA_03G240000 [Glycine max];PREDICTED: transcription factor bHLH94-like [Glycine max]
Glyma. 19G216 200	-	-	-	-	-	-	-	-	-1.281	0.00029	1266	ko04650//Natural killer cell mediated cytotoxicity;ko04075//Plant hormone signal transduction;ko04626//Plant-pathogen interaction	-	GO:0001071//nucleic acid binding transcription factor activity;GO:0003677//DNA binding;GO:0046983//protein dimerization activity	GO:0033554//cellular response to stress;GO:0051240;GO:0006351//transcription, DNA-templated	gi|351721812|ref|NP_001237222.1|/1.50678e-94/bZIP transcription factor bZIP41 [Glycine max]
Glyma. 03G170 300	-	-	-	-	-	-	-	-	-1.2884	0.00842	3015	ko04075//Plant hormone signal transduction;ko04712//Circadian rhythm - plant;ko04626//Plant-pathogen interaction	-	GO:0005515//protein binding	-	gi|918463563|gb|ALA09126.1|;gi|947119269|gb|KRH67518.1|/0;0/bHLH transcription factor, partial [Glycine max] ;hypothetical protein GLYMA_03G170300 [Glycine max]
Glyma. 17G258 700	-	-	-	-	-	-	-	-	-1.3062	8.68E-05	2887	ko04141//Protein processing in endoplasmic reticulum;ko04626//Plant-pathogen interaction	GO:0009536//plastid;GO:0044437;GO:0005911//cell-cell junction;GO:0005576//extracellular region;GO:0016020//membrane	GO:0032550;GO:0005515//protein binding	GO:0010038//response to metal ion;GO:0044267//cellular protein metabolic process;GO:0009933//meristem structural organization;GO:0010073//meristem maintenance;GO:0006970//response to osmotic stress;GO:0015031//protein transport	gi|571539837|ref|XP_006601356.1|;gi|734351227|gb|KHN12637.1|/0;0/PREDICTED: endoplasmin homolog isoform X2 [Glycine max];Endoplasmin like [Glycine soja]
Glyma. 15G100 100	-	-	-	-	-	-	-	-	-1.3487	0.00032	1828	ko04075//Plant hormone signal transduction;ko04626//Plant-pathogen	GO:0042579//microbody	GO:0004672//protein kinase activity;GO:0032550;GO:0036094//small molecule	GO:0006468//protein phosphorylation	gi|947062043|gb|KRH11304.1|;gi|955369108|ref|XP_014623058.1|/4.33873e-143;0/hypothetical protein GLYMA_15G1001002 [Glycine max];PREDICTED: receptor-like cytosolic

20 × 20

												interaction		binding;GO:1901363;GO:0097159//organic cyclic compound binding		serine/threonine-protein kinase RBK2 [Glycine max]
Glyma.02G275600	-	-	-	-	-	-	-	-	-1.3904	0.00011	945	ko04070//Phosphatidylinositol signaling system;ko04626//Plant-pathogen interaction	GO:0044437;GO:0016020//membrane	GO:0046872//metal ion binding	GO:0019932//second-messenger-mediated signaling;GO:0006637//acyl-CoA metabolic process;GO:0009640//photomorphogenesis;GO:0009593//detection of chemical stimulus;GO:0009605//response to external stimulus	gi|413945839|gb|AFW78488.1|/1.43783e-104/calmodulin1 [Zea mays]
Glyma.07G212000	-	-	-	-	-	-	-	-	-1.4109	0.00985	2030	ko04626//Plant-pathogen interaction	GO:0043231//intracellular membrane-bounded organelle	GO:0046872//metal ion binding	-	gi|574584784|ref|NP_001276189.1|/1.00228e-123/uncharacterized protein LOC100803158 [Glycine max]
Glyma.15G161200	-	-	-	-	-	-	-	-	-1.4248	5.78E-05	1658	ko04626//Plant-pathogen interaction	-	GO:0004672//protein kinase activity;GO:0032550	GO:0006796//phosphate-containing compound metabolic process;GO:0006464//cellular protein modification process	gi|356556186|ref|XP_003546407.1|/0/PREDICTED: probable receptor-like serine/threonine-protein kinase At5g57670 [Glycine max]
Glyma.02G247900	-	-	-	-	-	-	-	-	-1.4861	0.00028	1012	ko04626//Plant-pathogen interaction	-	-	-	gi|351721110|ref|NP_001235151.1|;gi|947124831|gb|KRH73037.1|/1.61515e-111;6.5414e-125/uncharacterized protein LOC100500493 [Glycine max];hypothetical protein GLYMA_02G247900 [Glycine max]
Glyma.11G217400	-	-	-	-	-	-	-	-	-1.4973	0.0019	2085	ko04626//Plant-pathogen interaction	-	GO:0004672//protein kinase activity;GO:0032550	GO:0006464//cellular protein modification process	gi|947082168|gb|KRH30957.1|/0/hypothetical protein GLYMA_11G217400 [Glycine max]
Glyma.16G059700	-	-	-	-	-	-	-	-	-1.5447	0.00118	3377	ko04626//Plant-pathogen interaction	-	GO:0016301//kinase activity;GO:0032550	GO:0006796//phosphate-containing compound metabolic process;GO:0006464//cellular protein modification process	gi|571526053|ref|XP_006599042.1|;gi|571526049|ref|XP_006599041.1|/0;0/PREDICTED: probable inactive leucine-rich repeat receptor-like protein kinase At3g03770 isoform X2 [Glycine max];PREDICTED: probable inactive leucine-rich repeat receptor-like protein kinase At3g03770 isoform X1 [Glycine max]
Glyma.04G013800	-	-	-	-	-	-	-	-	-1.5499	0.00949	918	ko04075//Plant hormone signal transduction;ko04626//Plant-pathogen interaction	-	-	-	gi|734405996|gb|KHN33765.1|/8.27238e-149/Protein TIFY 10B [Glycine soja]
Glyma.13G035100	-	-	-	-	-	-	-	-	-1.6389	0.00304	1130	ko04626//Plant-pathogen interaction	-	-	-	gi|356547218|ref|XP_003542013.1|/4.33954e-91/PREDICTED: probable calcium-binding protein CML44 [Glycine max]
Glyma.16G099600	-	-	-	-	-	-	-	-	-1.6533	5.97E-05	969	ko04626//Plant-pathogen interaction	-	GO:0046872//metal ion binding;GO:0016628//oxidoreductase activity, acting on the CH-CH group of donors, NAD or NADP as acceptor	GO:0044710	gi|356559132|ref|XP_003547855.1|/8.65453e-114/PREDICTED: probable calcium-binding protein CML16 [Glycine max]
Glyma.13G370100	-	-	-	-	-	-	-	-	-1.6542	0.00019	1579	ko04626//Plant-pathogen interaction	-	-	-	gi|356550659|ref|XP_003543702.1|/0/PREDICTED: probable WRKY transcription factor 40 [Glycine max]
Glyma.14G185100	-	-	-	-	-	-	-	-	-1.7714	0.00542	1692	ko04075//Plant hormone signal transduction;ko04626//Plant-pathogen interaction	-	GO:0005515//protein binding	-	gi|734419257|gb|KHN39978.1|/0/Transcription factor bHLH93 [Glycine soja]

Glyma.15G078200	-	-	-	-	-	-	-	-	-1.7774	0.00012	1808	ko04626//Plant-pathogen interaction	-	GO:0016301//kinase activity;GO:0032550	GO:0006796//phosphate-containing compound metabolic process;GO:0006464//cellular protein modification process	gi|955373512|ref|XP_014623829.1|/0/PREDICTED: serine/threonine-protein kinase-like protein ACR4 [Glycine max]
Glyma.19G181200	-	-	-	-	-	-	-	-	-1.8218	0.00077	1608	ko04626//Plant-pathogen interaction	-	GO:0004672//protein kinase activity;GO:0032550	GO:0006464//cellular protein modification process	gi|571558485|ref|XP_006604574.1|;gi|947046350|gb|KRG95979.1|;gi|356570752|ref|XP_003553549.1|/0;0;0/PREDICTED: putative serine/threonine-protein kinase isoform X3 [Glycine max];hypothetical protein GLYMA_19G181200 [Glycine max];PREDICTED: putative serine/threonine-protein kinase isoform X1 [Glycine max]
Glyma.13G234800	-	-	-	-	-	-	-	-	-1.832	0.0026	1843	ko04626//Plant-pathogen interaction	-	GO:0016301//kinase activity;GO:0032550	GO:0006796//phosphate-containing compound metabolic process;GO:0006464//cellular protein modification process	gi|356549280|ref|XP_003543022.1|/0/PREDICTED: serine/threonine-protein kinase-like protein ACR4 [Glycine max]
Glyma.06G092000	-	-	-	-	-	-	-	-	-1.9707	0.00501	1587	ko04075//Plant hormone signal transduction;ko04626//Plant-pathogen interaction	-	GO:0005515//protein binding	-	gi|734339619|gb|KHN09034.1|/0/Transcription factor bHLH93 [Glycine soja]
Glyma.14G206500	-	-	-	-	-	-	-	-	-2.0492	0.00723	4627	ko04626//Plant-pathogen interaction	-	-	-	gi|734398662|gb|KHN30617.1|/0/Disease resistance protein [Glycine soja]
Glyma.06G271200	-	-	-	-	-	-	-	-	-2.0504	0.00562	2469	ko04626//Plant-pathogen interaction	-	-	-	gi|356515024|ref|XP_003526201.1|/0/PREDICTED: cyclic nucleotide-gated ion channel 4-like [Glycine max]
Glyma.20G185100	-	-	-	-	-	-	-	-	-2.238	0.00042	2296	ko04626//Plant-pathogen interaction	-	-	GO:0009608//response to symbiont;GO:0044419//interspecies interaction between organisms	gi|571568702|ref|XP_003556257.2|/0/PREDICTED: piriformospora indica-insensitive protein 2-like [Glycine max]
Glyma.03G148800	-	-	-	-	-	-	-	-	-2.3239	6.41E-06	2137	ko04075//Plant hormone signal transduction;ko04626//Plant-pathogen interaction	-	GO:0004672//protein kinase activity;GO:0032550	GO:0006464//cellular protein modification process	gi|356504892|ref|XP_003521228.1|/0/PREDICTED: receptor-like cytosolic serine/threonine-protein kinase RBK2 [Glycine max]
Glyma.19G152100	-	-	-	-	-	-	-	-	-2.44	0.00019	2285	ko04626//Plant-pathogen interaction	-	GO:0004672//protein kinase activity;GO:0032550	GO:0006464//cellular protein modification process	gi|947045825|gb|KRG95454.1|;gi|356570544|ref|XP_003553445.1|/0;0/hypothetical protein GLYMA_19G152100 [Glycine max];PREDICTED: receptor-like cytosolic serine/threonine-protein kinase RBK2 [Glycine max]
Glyma.18G039400	-	-	-	-	-	-	-	-	-2.4444	1.98E-05	2076	ko04626//Plant-pathogen interaction	-	GO:0004672//protein kinase activity;GO:0032550	GO:0006464//cellular protein modification process	gi|356569560|ref|XP_003552967.1|/0/PREDICTED: serine/threonine-protein kinase CDL1-like [Glycine max]
Glyma.09G265700	-	-	-	-	-	-	-	-	-2.4727	0.00336	3737	ko00230//Purine metabolism;ko00240//Pyrimidine metabolism;ko01100//Metabolic pathways;ko03020//RNA polymerase;ko04626//Plant-pathogen interaction	-	-	-	gi|955342818|ref|XP_014617918.1|/0/PREDICTED: putative disease resistance protein RGA4 [Glycine max]
Glyma.13G187600	-	-	-	-	-	-	-3.5659	3.23E-08	-2.5846	0.00174	2287	ko04626//Plant-pathogen interaction	-	GO:0004672//protein kinase activity;GO:0032550	GO:0006468//protein phosphorylation	gi|947071700|gb|KRH20591.1|/0/hypothetical protein GLYMA_13G187600 [Glycine max]

20 × 20

Glyma.13G083700	-	-	-	-	-	-	-	-	-2.7088	0.00153	833	ko04070//Phosphatidylinositol signaling system;ko04626//Plant-pathogen interaction	-	GO:0046872//metal ion binding	-	gi|356548725|ref|XP_003542750.1|/7.13349e-108/PREDICTED: probable calcium-binding protein CML29 [Glycine max]
Glyma.08G271000	-	-	-	-	-	-	-	-	-3.2491	3.55E-11	1909	ko04075//Plant hormone signal transduction;ko04626//Plant-pathogen interaction	-	GO:0005515//protein binding	-	gi|955337298|ref|XP_014634765.1|/0/PREDICTED: transcription factor bHLH71-like isoform X1 [Glycine max]
Glyma.18G271000	-1.6706	0.000559	-1.9606	1.60E-05	-2.0219	4.61E-06	-	-	-3.4182	5.75E-05	2592	ko04626//Plant-pathogen interaction	-	GO:0004672//protein kinase activity	GO:0006796//phosphate-containing compound metabolic process	gi|571549488|ref|XP_006602953.1|/0/PREDICTED: receptor-like protein kinase FERONIA [Glycine max]
Glyma.17G261000	-	-	-	-	-	-	-	-	-4.0149	7.89E-08	1809	ko04626//Plant-pathogen interaction	GO:0043231//intracellular membrane-bounded organelle;GO:0016020//membrane	GO:0046872//metal ion binding;GO:0004674//protein serine/threonine kinase activity;GO:0032550	GO:0006796//phosphate-containing compound metabolic process;GO:0006464//cellular protein modification process	gi|571539899|ref|XP_003549475.2|/0/PREDICTED: calcium-dependent protein kinase 2-like [Glycine max]
Glyma.03G088100	-11.6	1.16E-58	-11.133	1.42E-39	-10.886	7.06E-38	-5.5575	2.36E-18	-4.2575	5.55E-10	4005	ko00230//Purine metabolism;ko00240//Pyrimidine metabolism;ko01100//Metabolic pathways;ko03020//RNA polymerase;ko04626//Plant-pathogen interaction	-	-	GO:0050896//response to stimulus	gi|947117928|gb|KRH66177.1|;gi|571443502|ref|XP_006576200.1|;gi|571443498|ref|XP_006576198.1|/0;0;0/hypothetical protein GLYMA_03G088100 [Glycine max];PREDICTED: uncharacterized LOC100527480 isoform X2 [Glycine max];PREDICTED: uncharacterized LOC100527480 isoform X1 [Glycine max]
Glyma.15G209200	-12.016	2.53E-13	-11.865	1.38E-11	-10.627	8.23E-11	-	-	-4.6062	0.00013	1119	ko04626//Plant-pathogen interaction	-	-	-	gi|947063715|gb|KRH12976.1|/0/hypothetical protein GLYMA_15G209200 [Glycine max]
Glyma.03G088000	-10.378	3.06E-31	-9.5189	1.88E-20	-9.4617	1.56E-21	-5.2323	1.81E-11	-5.2689	7.23E-09	3143	ko04626//Plant-pathogen interaction	-	-	-	gi|571444898|ref|XP_006576646.1|;gi|947117926|gb|KRH66175.1|/0;0/PREDICTED: TMV resistance protein N-like isoform X3 [Glycine max];hypothetical protein GLYMA_03G088000 [Glycine max]
Glyma.16G065700	-4.0878	2.47E-10	-5.3886	3.68E-12	-5.2294	2.02E-13	-3.0335	2.23E-06	-8.6387	2.44E-10	4096	ko04626//Plant-pathogen interaction	-	GO:0016301//kinase activity	GO:0016310//phosphorylation	gi|955377186|ref|XP_014624710.1|/0/PREDICTED: probable LRR receptor-like serine/threonine-protein kinase At4g08850 [Glycine max]
Glyma.01G010200	-	-	-	-	-	-	-	-	-1.5559	0.00272	1827	ko01100//Metabolic pathways;ko00710//Carbon fixation in photosynthetic organisms	GO:0009532//plastid stroma;GO:0009526//plastid envelope;GO:0009534//chloroplast thylakoid;GO:0005576//extracellular region	GO:0032550;GO:0016301//kinase activity	GO:0044238//primary metabolic process;GO:0009617//response to bacterium;GO:0006796//phosphate-containing compound metabolic process;GO:0006950//response to stress	gi|356495988|ref|XP_003516852.1|/0/PREDICTED: phosphoribulokinase, chloroplastic [Glycine max]
Glyma.01G153300	-	-	-	-	-3.0901	0.00016	-	-	-	-	2099	ko00906//Carotenoid biosynthesis	-	GO:0046906//tetrapyrrole binding;GO:0046914//transition metal ion binding;GO:0016709//oxidoreductase activity, acting on paired donors, with	GO:0010162//seed dormancy process;GO:0009620//response to fungus;GO:0009416//response to light stimulus;GO:0006714//sesqui	gi|947128604|gb|KRH76458.1|;gi|947128603|gb|KRH76457.1|/0;0/hypothetical protein GLYMA_01G153300 [Glycine max];hypothetical protein GLYMA_01G153300 [Glycine max]

														incorporation or reduction of molecular oxygen, NAD(P)H as one donor, and incorporation of one atom of oxygen	terpenoid metabolic process;GO:0006950//response to stress	
Glyma.01G163100	-	-	-	-	-	-	-	-	2.47807	0.00109	1698	ko01100//Metabolic pathways;ko01110//Biosynthesis of secondary metabolites;ko00360//Phenylalanine metabolism;ko00940//Phenylpropanoid biosynthesis	-	GO:0003824//catalytic activity;GO:0016209//antioxidant activity;GO:0043169//cation binding;GO:0046906//tetrapyrrole binding	GO:0044710;GO:0006950//response to stress	gi|734411617|gb|KHN36003.1|;gi|947128754|gb|KRH76608.1|/0;0/Peroxidase 64 [Glycine soja] ;hypothetical protein GLYMA_01G163100 [Glycine max]
Glyma.01G171100	-	-	-	-	-	-	-	-	4.48764	0.00016	1661	ko01100//Metabolic pathways;ko01110//Biosynthesis of secondary metabolites;ko00360//Phenylalanine metabolism;ko00940//Phenylpropanoid biosynthesis	GO:0005618//cell wall	GO:0043169//cation binding;GO:0046906//tetrapyrrole binding;GO:0016209//antioxidant activity;GO:0003824//catalytic activity	GO:0010383;GO:0009699//phenylpropanoid biosynthetic process;GO:0044702//single organism reproductive process;GO:0006950//response to stress	gi|356496705|ref|XP_003517206.1|/0/PREDICTED: peroxidase 72-like [Glycine max]
Glyma.01G203400	-	-	-	-	-	-	-	-	3.47236	0.00056	2041	ko00500//Starch and sucrose metabolism;ko01100//Metabolic pathways	GO:0009532//plastid stroma	GO:0016160//amylase activity	GO:0006950//response to stress;GO:0005976//polysaccharide metabolic process;GO:0000023//maltose metabolic process	gi|571436239|ref|XP_006573703.1|/0/PREDICTED: beta-amylase 3, chloroplastic [Glycine max]
Glyma.01G214200	-1.6511	6.82E-11	-	-	-	-	-	-	4.63818	4.28E-25	1320	ko00941//Flavonoid biosynthesis;ko01100//Metabolic pathways;ko01110//Biosynthesis of secondary metabolites	-	GO:0016705//oxidoreductase activity, acting on paired donors, with incorporation or reduction of molecular oxygen	GO:0009699//phenylpropanoid biosynthetic process;GO:0006950//response to stress;GO:0001101//response to acid chemical;GO:0006996//organelle organization	gi|358248024|ref|NP_001239794.1|/0/anthocyanidin synthase [Glycine max]
Glyma.01G219400	-1.1738	5.77E-07	-	-	-	-	-	-	-	-	820	ko00480//Glutathione metabolism;ko00590//Arachidonic acid metabolism	-	GO:0004601//peroxidase activity	GO:0006950//response to stress;GO:0044710	gi|351727154|ref|NP_001236895.1|/8.45814e-121/uncharacterized protein LOC100306570 [Glycine max]
Glyma.02G032300	-1.7307	4.20E-07	-	-	-	-	-	-	-	-	866	ko04075//Plant hormone signal transduction	-	-	GO:0006950//response to stress	gi|359807353|ref|NP_001241124.1|/1.75331e-109/uncharacterized protein LOC100783267 [Glycine max]
Glyma.02G093000	-	-	-	-	-	-	-	-	-1.499	0.00924	1491	-	-	GO:0046983//protein dimerization activity;GO:0004661//protein geranylgeranyltransferase activity	GO:0018342//protein prenylation;GO:0006950//response to stress;GO:0009725//response to hormone;GO:0001101//response to acid chemical	gi|734391396|gb|KHN27225.1|/0/Geranylgeranyl transferase type-1 subunit beta [Glycine soja]
Glyma.02G157900	2.17653	4.63E-14	-	-	-	-	-	-	-	-	1215	-	GO:0043231//intracellular membrane-bounded organelle	-	GO:0006950//response to stress	gi|947123394|gb|KRH71600.1|;gi|356500303|ref|XP_003518972.1|/1.23787e-86;1.80694e-58/hypothetical protein GLYMA_02G157900 [Glycine max];PREDICTED: uncharacterized protein LOC100527260 [Glycine max]
Glyma.02G224100	-	-	-	-	-	-	-	-	-1.7523	0.00032	826	-	GO:0031981//nuclear lumen;GO:0009536//plastid;GO:0044437;GO:0000785//chromatin;GO:0	GO:0003676//nucleic acid binding;GO:0046983//protein dimerization activity	GO:0034728//nucleosome organization;GO:0006950//response to stress	gi|475622287|gb|EMT31824.1|/1.35134e-52/Histone H4 [Aegilops tauschii]

													005911//cell-cell junction;GO:0016020//membrane			
Glyma.02G228200	-	-	-	-	-	-	-	-	2.69927	0.00104	1955	ko04075//Plant hormone signal transduction	GO:0043231//intracellular membrane-bounded organelle	GO:0043169//cation binding;GO:0004721//phosphoprotein phosphatase activity	GO:0009620//response to fungus;GO:0006464//cellular protein modification process;GO:0009755//hormone-mediated signaling pathway;GO:0006950//response to stress	gi|356500878|ref|XP_003519257.1|;gi|947124500|gb|KRH72706.1|/0;9.96677e-128/PREDICTED: probable protein phosphatase 2C 25 [Glycine max];hypothetical protein GLYMA_02G228200 [Glycine max]
Glyma.02G255400	-	-	-	-	-	-	-	-	2.45564	0.00324	2207	-	-	-	GO:0006950//response to stress	gi|947124958|gb|KRH73164.1|/0/hypothetical protein GLYMA_02G255400 [Glycine max]
Glyma.03G056700	-	-	-	-	-	-	-	-	1.71214	0.00042	2411	ko01040//Biosynthesis of unsaturated fatty acids	GO:0009526//plastid envelope	GO:0016705//oxidoreductase activity, acting on paired donors, with incorporation or reduction of molecular oxygen	GO:0006950//response to stress;GO:0044710;GO:0044238//primary metabolic process	gi|358249324|ref|NP_001239777.1|/0/omega-3 fatty acid desaturase, chloroplastic-like [Glycine max]
Glyma.03G116600	-	-	-	-	-	-	-	-	-1.2228	0.00022	1885	ko04141//Protein processing in endoplasmic reticulum	-	GO:0043169//cation binding;GO:0005515//protein binding;GO:0032550	GO:0006950//response to stress;GO:0044267//cellular protein metabolic process	gi|734426241|gb|KHN43746.1|/0/Chaperone protein dnaJ 2 [Glycine soja]
Glyma.03G144400	-1.1908	0.000171	-	-	-	-	-	-	-	-	767	-	-	-	GO:0006950//response to stress;GO:0009791//post-embryonic development	gi|351727178|ref|NP_001236128.1|/1.64464e-90/soybean seed maturation polypeptides [Glycine max]
Glyma.03G253500	-	-	-	-	-	-	-	-	-1.2355	0.00138	2562	ko00906//Carotenoid biosynthesis;ko01100//Metabolic pathways;ko01110//Biosynthesis of secondary metabolites	GO:0031976;GO:0044436	GO:0016491//oxidoreductase activity	GO:0032787//monocarboxylic acid metabolic process;GO:0006950//response to stress;GO:0006778//porphyrin-containing compound metabolic process;GO:0016122//xanthophyll metabolic process	gi|359807530|ref|NP_001241404.1|;gi|571443623|ref|XP_006576259.1|/0;0/violaxanthin de-epoxidase, chloroplastic-like precursor [Glycine max];PREDICTED: violaxanthin de-epoxidase, chloroplastic-like isoform X5 [Glycine max]
Glyma.04G015900	-	-	-	-	-	-	-	-	-1.2822	0.00762	1767	ko01100//Metabolic pathways;ko00710//Carbon fixation in photosynthetic organisms	GO:0009521;GO:0031224//intrinsic component of membrane;GO:0009526//plastid envelope;GO:0009534//chloroplast thylakoid;GO:0005576//extracellular region	GO:0016620//oxidoreductase activity, acting on the aldehyde or oxo group of donors, NAD or NADP as acceptor;GO:0000166//nucleotide binding	GO:0009314//response to radiation;GO:0010038//response to metal ion;GO:0019318//hexose metabolic process;GO:0015977//carbon fixation;GO:0034285;GO:0044237//cellular metabolic process;GO:0006950//response to stress	gi|358248146|ref|NP_001240080.1|;gi|947112595|gb|KRH60897.1|/0;0/uncharacterized protein LOC100806482 [Glycine max];hypothetical protein GLYMA_04G015900 [Glycine max]
Glyma.04G042300	-	-	-	-	-	-	-	-	-1.715	8.19E-06	1307	-	-	GO:0003676//nucleic acid binding	GO:0001101//response to acid chemical;GO:0009725//response to hormone;GO:0006950//response to stress	gi|918463735|gb|ALA09212.1|/4.09473e-165/MYB/HD-like transcription factor, partial [Glycine max]

20 × 20

Glyma.04G095500	-	-	-	-	-	-	-	-	1.02603	0.00198	2834	-	GO:0042175//nuclear outer membrane-endoplasmic reticulum membrane network;GO:0043231//intracellular membrane-bounded organelle;GO:0044444	GO:0046914//transition metal ion binding;GO:0032553//ribonucleotide binding;GO:0016653//oxidoreductase activity, acting on NAD(P)H, heme protein as acceptor	GO:0006950//response to stress;GO:0044710;GO:0001101//response to acid chemical	gi|734336550|gb|KHN08276.1|/0/NADPH--cytochrome P450 reductase [Glycine soja]
Glyma.04G103900	-	-	-	-	-	-	-	-	1.35648	0.00552	1940	-	-	GO:0003676//nucleic acid binding	GO:0009628//response to abiotic stimulus;GO:0006950//response to stress;GO:0006351//transcription, DNA-templated	gi|918463511|gb|ALA09100.1|/6.86589e-168/AP2-EREBP transcription factor, partial [Glycine max]
Glyma.04G171800	-	-	-	-	-	-	-	-	-2.1105	1.72E-07	1156	-	GO:0031224//intrinsic component of membrane	GO:0005215//transporter activity	GO:0044765;GO:0044763;GO:0009628//response to abiotic stimulus;GO:0006950//response to stress	gi|947115071|gb|KRH63373.1|/0/hypothetical protein GLYMA_04G171800 [Glycine max]
Glyma.04G198200	-2.6678	5.29E-07	-3.3658	6.42E-11	-2.741	3.18E-05	-3.0729	1.59E-07	-4.8711	1.08E-13	1469	-	-	-	GO:0006950//response to stress	gi|955317145|ref|XP_014630314.1|;gi|571451376|ref|XP_006578711.1|;gi|947115507|gb|KRH63809.1|;gi|947115506|gb|KRH63808.1|/8.85606e-114;9.6204e-126;2.55023e-92;4.98088e-104/PREDICTED: protein TAPETUM DETERMINANT 1-like isoform X2 [Glycine max];PREDICTED: protein TAPETUM DETERMINANT 1-like isoform X1 [Glycine max];hypothetical protein GLYMA_04G198200 [Glycine max];hypothetical protein GLYMA_04G198200 [Glycine max]
Glyma.04G234900	-	-	-	-	-	-	-	-	-1.1283	0.00084	1920	-	GO:0044444	-	GO:0006950//response to stress;GO:0007165//signal transduction	gi|734370890|gb|KHN19500.1|/0/PP2A regulatory subunit TAP46 [Glycine soja]
Glyma.05G055600	2.14597	1.57E-22	1.87087	1.70E-07	1.72717	8.21E-09	1.51907	5.33E-05	1.89239	0.00019	2415	-	GO:0044424	-	GO:0010035//response to inorganic substance;GO:0006950//response to stress;GO:0009628//response to abiotic stimulus	gi|356514200|ref|XP_003525794.1|/0/PREDICTED: hsp70-Hsp90 organizing protein 1-like [Glycine max]
Glyma.05G103600	1.14818	1.02E-06	-	-	-	-	-	-	3.03169	0.00478	1511	ko01100//Metabolic pathways;ko01110//Biosynthesis of secondary metabolites;ko00360//Phenylalanine metabolism;ko00940//Phenylpropanoid biosynthesis	-	GO:0003824//catalytic activity;GO:0016209//antioxidant activity;GO:0043169//cation binding;GO:0046906//tetrapyrrole binding	GO:0044710;GO:0006950//response to stress	gi|734328591|gb|KHN06005.1|/0/Peroxidase 73 [Glycine soja]
Glyma.05G150100	-	-	-	-	-	-	-	-	3.25457	0.00072	533	-	-	-	GO:0051707//response to other organism;GO:0001101//response to acid chemical;GO:0009694//jasmonic acid metabolic	gi|947110493|gb|KRH58819.1|/3.72565e-46/hypothetical protein GLYMA_05G150100 [Glycine max]

20 × 20

															process;GO:0010243//response to organonitrogen compound;GO:0009755//hormone-mediated signaling pathway;GO:0006950//response to stress;GO:0006984//ER-nucleus signaling pathway	
Glyma.05G192800	1.11757	1.37E-06	1.20541	0.000287	1.06995	0.00074	-	-	-	-	1167	-	-	-	GO:0006950//response to stress;GO:0010118//stomatal movement	gi|571453058|ref|XP_006579344.1|/3.27039e-180/PREDICTED: uncharacterized protein LOC100305573 isoform X2 [Glycine max]
Glyma.05G193500	-1.0648	7.82E-06	-	-	-	-	-	-	-	-	1538	-	-	GO:0046914//transition metal ion binding;GO:0003824//catalytic activity	GO:0009617//response to bacterium;GO:0006950//response to stress;GO:0009755//hormone-mediated signaling pathway;GO:0032446//protein modification by small protein conjugation	gi|734406748|gb|KHN34095.1|/0/E3 ubiquitin-protein ligase RGLG1 [Glycine soja]
Glyma.05G195000	-	-	-	-	-2.6388	0.00091	-	-	3.46722	1.71E-05	1488	-	GO:0009536//plastid	GO:0003676//nucleic acid binding;GO:0019901//protein kinase binding	GO:0009314//response to radiation;GO:0051707//response to other organism;GO:0001101//response to acid chemical;GO:0032535//regulation of cellular component size;GO:0034285;GO:0006950//response to stress;GO:0006351//transcription, DNA-templated	gi|959092818|ref|NP_001304649.1|/0/NAC domain-containing protein 2 [Glycine max]
Glyma.05G211700	-	-	#######	2.36E-05	#######	1.97E-08	-	-	-	-	2131	ko00270//Cysteine and methionine metabolism;ko01100//Metabolic pathways;ko01110//Biosynthesis of secondary metabolites	-	GO:0043168//anion binding;GO:0005515//protein binding;GO:0016846//carbon-sulfur lyase activity	GO:0001101//response to acid chemical;GO:0010039//response to iron ion;GO:0009692;GO:0009725//response to hormone;GO:0018871;GO:0003006//developmental process involved in reproduction;GO:0006950//response to stress;GO:0009605//response to external stimulus	gi|351726068|ref|NP_001236858.1|/0/1-aminocyclopropane-1-carboxylate synthase [Glycine max]
Glyma.06G002000	-	-	-	-	-	-	-	-	1.70251	2.14E-05	2049	-	GO:0031224//intrinsic component of membrane	GO:0005515//protein binding	GO:0006950//response to stress	gi|356517840|ref|XP_003527594.1|/0/PREDICTED: MLO-like protein 1 [Glycine max]
Glyma.06G034600	-	-	-	-	-	-	-	-	2.10368	0.00783	1909	ko00500//Starch and sucrose metabolism;ko01110//Biosynthesis of secondary metabolites;ko00520//Amino sugar and nucleotide sugar metabolism	-	GO:0016740//transferase activity	GO:0003002//regionalization;GO:0042545//cell wall modification;GO:0006950//response to stress;GO:0048827//phyllome development;GO:0000003//reproduction;GO:0010413//glucuronoxylan metabolic process	gi|356519066|ref|XP_003528195.1|/0/PREDICTED: probable galacturonosyltransferase-like 1 [Glycine max]
Glyma.06G058500	-	-	-	-	-	-	1.45219	0.00012	-	-	1998	ko00062//Fatty acid elongation;ko01110//Biosynthesis of secondary metabolites	GO:0043231//intracellular membrane-bounded organelle	GO:0016746//transferase activity, transferring acyl groups	GO:0009314//response to radiation;GO:0006950//response to stress;GO:0006631//fatty acid metabolic process	gi|356514790|ref|XP_003526086.1|/0/PREDICTED: 3-ketoacyl-CoA synthase 10-like [Glycine max]

Glyma.06G114400	-	-	-	-	-	-	-	-	1.75694	0.00525	1499	ko00053//Ascorbate and aldarate metabolism;ko00480//Glutathione metabolism	GO:0031976	GO:0046906//tetrapyrrole binding;GO:0016209//antioxidant activity	GO:0009409//response to cold;GO:0007165//signal transduction;GO:0044710;GO:0006950//response to stress;GO:0044699;GO:0050896//response to stimulus	gi|947104871|gb|KRH53254.1|;gi|356515910|ref|XP_003526640.1|;gi|947104870|gb|KRH53253.1|/9.09605e-131;0;0/hypothetical protein GLYMA_06G114400 [Glycine max];PREDICTED: probable L-ascorbate peroxidase 6, chloroplastic isoform X1 [Glycine max];hypothetical protein GLYMA_06G114400 [Glycine max]
Glyma.06G146200	-	-	-	-	-	-	-	-	2.64031	3.46E-03	669	-	-	GO:0016462//pyrophosphatase activity;GO:0032550	GO:0006950//response to stress;GO:0007154//cell communication	gi|947105410|gb|KRH53793.1|/2.22017e-164/hypothetical protein GLYMA_06G146200 [Glycine max]
Glyma.06G157400	-	-	-	-	-3.1491	8.92E-05	-	-	-	-	1609	-	GO:0009536//plastid	GO:0003676//nucleic acid binding;GO:0019901//protein kinase binding	GO:0009314//response to radiation;GO:0051707//response to other organism;GO:0001101//response to acid chemical;GO:0032535//regulation of cellular component size;GO:0034285;GO:0006950//response to stress;GO:0006351//transcription, DNA-templated	gi|513044651|gb|AGO14648.1|;gi|947105577|gb|KRH53960.1|/0;0/NAC transcription factor [Glycine max];hypothetical protein GLYMA_06G157400 [Glycine max]
Glyma.06G160300	-	-	-	-	-	-	-	-	-1.8168	0.00013	584	-	-	-	GO:0001906//cell killing;GO:0006950//response to stress;GO:0009620//response to fungus	gi|947105631|gb|KRH54014.1|/2.52264e-26/hypothetical protein GLYMA_06G160300 [Glycine max]
Glyma.06G160400	-	-	-	-	-	-	-	-	4.86255	6.53E-05	642	-	-	-	GO:0006950//response to stress	gi|734341038|gb|KHN09676.1|/1.27287e-30/Defensin-like protein 6 [Glycine soja]
Glyma.06G167300	1.65043	1.31E-05	-	-	-	-	-	-	-	-	1714	-	-	-	GO:0006950//response to stress	gi|571460950|ref|XP_006581852.1|/1.48014e-100/PREDICTED: protein TAPETUM DETERMINANT 1-like [Glycine max]
Glyma.06G179200	-1.3003	0.0001	-	-	-	-	-	-	-	-	2618	ko00052//Galactose metabolism	GO:0009536//plastid	GO:0008378//galactosyltransferase activity	GO:0006950//response to stress;GO:0001101//response to acid chemical;GO:0044238//primary metabolic process	gi|571461147|ref|XP_003527005.2|;gi|571461149|ref|XP_006581911.1|/0;0/PREDICTED: galactinol--sucrose galactosyltransferase isoform X1 [Glycine max];PREDICTED: galactinol--sucrose galactosyltransferase isoform X2 [Glycine max]
Glyma.06G192100	-	-	-	-	-3.0559	3.37E-06	-4.4806	1.14E-16	-5.5694	3.88E-10	1957	-	GO:0031224//intrinsic component of membrane	GO:0005215//transporter activity	GO:0044763;GO:0009628//response to abiotic stimulus;GO:0006950//response to stress	gi|356519052|ref|XP_003528188.1|/0/PREDICTED: organic cation/carnitine transporter 3-like [Glycine max]
Glyma.06G203800	-	-	-	-	-	-	-	-	1.96284	0.0008	2929	ko03015//mRNA surveillance pathway;ko03040//Spliceosome	GO:0032991//macromolecular complex;GO:0019028//viral capsid	GO:0036094//small molecule binding;GO:0097159//organic cyclic compound binding;GO:0046914//transition metal ion binding;GO:0032550	GO:0006950//response to stress	gi|571461496|ref|XP_006582018.1|;gi|955325195|ref|XP_014632096.1|;gi|571461494|ref|XP_003528224.2|/0;0;0/PREDICTED: glycine-rich cell wall structural protein 1.8-like isoform X2 [Glycine max];PREDICTED: glycine-rich cell wall structural protein 1.8-like isoform X3 [Glycine max];PREDICTED: glycine-rich cell wall structural protein 1.8-like isoform X1 [Glycine max]
Glyma.06G248900	-	-	-	-	-3.8586	2.79E-05	-	-	-	-	1786	-	GO:0043231//intracellular membrane-bounded organelle	GO:0003676//nucleic acid binding	GO:0001101//response to acid chemical;GO:0006950//response to stress;GO:0006351//transcription, DNA-templated	gi|564010296|gb|AHB63576.1|/0/NAC transcription factor [Glycine max]

Glyma.06G309100	-	-	-	-	-	-	-	-	-1.2445	0.00242	1565	-	GO:0043231//intracellular membrane-bounded organelle	-	GO:0006950//response to stress	gi|571457925|ref|XP_006580951.1|;gi|351727048|ref|NP_001237915.1|/1.7048e-78;1.30928e-53/PREDICTED: uncharacterized protein LOC100527051 isoform X1 [Glycine max];uncharacterized protein LOC100527051 [Glycine max]
Glyma.07G026300	-	-	-	-	-	-	-	-	-1.0438	0.0035	1418	ko01100//Metabolic pathways;ko01110//Biosynthesis of secondary metabolites;ko00940//Phenylpropanoid biosynthesis	-	GO:0048037//cofactor binding	GO:0008152//metabolic process;GO:0006950//response to stress;GO:0010038//response to metal ion;GO:0001101//response to acid chemical	gi|734313393|gb|KHN01290.1|/0/Dihydroflavonol-4-reductase [Glycine soja]
Glyma.07G151300	-	-	-	-	-	-	-	-	1.40593	0.00181	2130	ko01040//Biosynthesis of unsaturated fatty acids	GO:0009941//chloroplast envelope	GO:0016705//oxidoreductase activity, acting on paired donors, with incorporation or reduction of molecular oxygen	GO:0006633//fatty acid biosynthetic process;GO:0006950//response to stress	gi|734428355|gb|KHN44706.1|/0/Omega-3 fatty acid desaturase, chloroplastic [Glycine soja]
Glyma.07G152900	-	-	-	-	-	-	-	-	-1.5243	5.53E-06	2549	ko04141//Protein processing in endoplasmic reticulum	GO:0005911//cell-cell junction;GO:0044432	GO:0003824//catalytic activity	GO:0006950//response to stress;GO:0000904//cell morphogenesis involved in differentiation;GO:0044710	gi|356523332|ref|XP_003530294.1|;gi|947100925|gb|KRH49417.1|/0;0/PREDICTED: dnaJ protein ERDJ3A-like [Glycine max];hypothetical protein GLYMA_07G152900 [Glycine max]
Glyma.07G153100	-	-	-	-	-	-	-	-	1.84364	1.86E-07	2322	ko00010//Glycolysis / Gluconeogenesis;ko01100//Metabolic pathways;ko01110//Biosynthesis of secondary metabolites	GO:0044444	GO:0046872//metal ion binding;GO:0019842//vitamin binding	GO:0006950//response to stress	gi|356521044|ref|XP_003529168.1|/0/PREDICTED: pyruvate decarboxylase 2 [Glycine max]
Glyma.07G252100	-	-	-	-	-	-	-	-	1.43578	0.00029	12033	-	-	GO:0017111//nucleoside-triphosphatase activity;GO:0032550;GO:0003676//nucleic acid binding	GO:0048731//system development;GO:0009755//hormone-mediated signaling pathway;GO:0009966//regulation of signal transduction;GO:0008152//metabolic process;GO:0006950//response to stress	gi|571467801|ref|XP_006584045.1|/0/PREDICTED: chromatin structure-remodeling complex protein SYD-like isoform X1 [Glycine max]
Glyma.07G266200	-	-	-	-	-	-	-	-	2.87174	0.00144	1525	ko00562//Inositol phosphate metabolism;ko00053//Ascorbate and aldarate metabolism;ko04070//Phosphatidylinositol signaling system;ko01100//Metabolic pathways;ko01110//Biosynthesis of secondary metabolites	GO:0044444;GO:0016020//membrane	GO:0070456	GO:0019852//L-ascorbic acid metabolic process;GO:0046488//phosphatidylinositol metabolic process;GO:0009628//response to abiotic stimulus;GO:0006950//response to stress	gi|734423661|gb|KHN42287.1|;gi|947102679|gb|KRH51171.1|/0;0/Inositol monophosphatase 3 [Glycine soja] ;hypothetical protein GLYMA_07G266200 [Glycine max]
Glyma.07G273800	-	-	-	-	-	-	-	-	1.5463	0.00852	3844	ko00562//Inositol phosphate metabolism;ko04070//Phosphatidylinositol signaling system;ko01100//Metabolic pathways	-	GO:0046030//inositol trisphosphate phosphatase activity	GO:0009888//tissue development;GO:0001101//response to acid chemical;GO:0046488//phosphatidylinositol metabolic process;GO:0009416//response to light stimulus;GO:0031667//response to nutrient levels;GO:0009756//carbohydrate mediated signaling;GO:0048878//chemical	gi|571468135|ref|XP_006584139.1|/0/PREDICTED: type I inositol polyphosphate 5-phosphatase 12-like isoform X2 [Glycine max]

20 × 20

															homeostasis;GO:0044723;GO:0022622//root system development;GO:0006950//response to stress	
Glyma.08G003900	-	-	-	-	-	-	-	-	7.60589	7.09E-06	2090	ko00564//Glycerophospholipid metabolism;ko04070//Phosphatidylinositol signaling system;ko00561//Glycerolipid metabolism;ko01100//Metabolic pathways	-	GO:0016301//kinase activity	GO:0006796//phosphate-containing compound metabolic process;GO:0006950//response to stress;GO:0048827//phyllome development;GO:0007186//G-protein coupled receptor signaling pathway;GO:0022622//root system development	gi|734363861|gb|KHN16922.1|/5.93051e-137/Diacylglycerol kinase 1 [Glycine soja]
Glyma.08G018000	-	-	-	-	-3.58	2.36E-05	-	-	2.58475	0.01002	2236	ko00270//Cysteine and methionine metabolism;ko01100//Metabolic pathways;ko01110//Biosynthesis of secondary metabolites	-	GO:0043168//anion binding;GO:0005515//protein binding;GO:0016846//carbon-sulfur lyase activity	GO:0001101//response to acid chemical;GO:0010039//response to iron ion;GO:0009692;GO:0009725//response to hormone;GO:0018871;GO:0003006//developmental process involved in reproduction;GO:0006950//response to stress;GO:0009605//response to external stimulus	gi|947092656|gb|KRH41241.1|/0/hypothetical protein GLYMA_08G018000 [Glycine max]
Glyma.08G165500	-	-	-	-	-	-	-	-	-1.1441	0.00085	1946	ko00010//Glycolysis / Gluconeogenesis;ko01100//Metabolic pathways;ko01110//Biosynthesis of secondary metabolites;ko00710//Carbon fixation in photosynthetic organisms	GO:0030312//external encapsulating structure; GO:0009532//plastid stroma;GO:0009526//plastid envelope; GO:0005576//extracellular region	GO:0032550;GO:0016301//kinase activity	GO:0010038//response to metal ion;GO:0006091//generation of precursor metabolites and energy;GO:0006796//phosphate-containing compound metabolic process;GO:0006950//response to stress	gi|356525742|ref|XP_003531482.1|/0/PREDICTED: phosphoglycerate kinase, cytosolic [Glycine max]
Glyma.08G181500	-	-	-	-	-	-	-	-	1.45236	0.00429	1365	ko01100//Metabolic pathways;ko01110//Biosynthesis of secondary metabolites;ko00360//Phenylalanine metabolism;ko00940//Phenylpropanoid biosynthesis	-	GO:0003824//catalytic activity;GO:0016209//antioxidant activity;GO:0043169//cation binding;GO:0046906//tetrapyrrole binding	GO:0044710;GO:0006950//response to stress	gi|571471963|ref|XP_006585454.1|/0/PREDICTED: peroxidase 43-like [Glycine max]
Glyma.08G196800	-	-	-	-	-	-	-	-	1.92843	2.28E-05	1631	-	GO:0016020//membrane	GO:0010178//IAA-amino acid conjugate hydrolase activity	GO:0002831;GO:0006950//response to stress	gi|734347256|gb|KHN11319.1|;gi|947095629|gb|KRH44214.1|/0;0/IAA-amino acid hydrolase ILR1-like 4 [Glycine soja] ;hypothetical protein GLYMA_08G196800 [Glycine max]
Glyma.08G230500	-	-	-	-	-	-	-	-	10.4957	1.50E-13	790	-	-	-	GO:0006950//response to stress	gi|351726098|ref|NP_001236859.1|/1.45163e-110/uncharacterized protein LOC100499848 [Glycine max]
Glyma.08G346700	3.14144	1.95E-49	2.80158	9.56E-17	2.41662	2.60E-14	1.91113	4.83E-08	2.85661	6.60E-10	2216	-	GO:0043231//intracellular membrane-bounded	GO:0016859//cis-trans isomerase activity;GO:1902936	GO:0044707;GO:0018208//peptidyl-proline modification;GO:0006950//response to stress;GO:0010038//response	gi|955338317|ref|XP_014634970.1|/0/PREDICTED: peptidyl-prolyl cis-trans isomerase FKBP62-like [Glycine max]

20 × 20

													organelle;GO:0044444		to metal ion	
Glyma.09G011100	-	-	-	-	-	-	-	-	-1.5069	0.00123	1825	ko00562//Inositol phosphate metabolism;ko00053//Ascorbate and aldarate metabolism;ko04070//Phosphatidylinositol signaling system;ko01100//Metabolic pathways;ko01110//Biosynthesis of secondary metabolites	GO:0044444;GO:0016020//membrane	GO:0070456	GO:0019852//L-ascorbic acid metabolic process;GO:0046488//phosphatidylinositol metabolic process;GO:0009628//response to abiotic stimulus;GO:0006950//response to stress	gi|734420434|gb|KHN40821.1|;gi|947087895|gb|KRH36560.1|/0;4.89277e-157/Inositol monophosphatase 3 [Glycine soja] ;hypothetical protein GLYMA_09G011100 [Glycine max]
Glyma.09G022300	-2.8098	1.83E-20	-	-	-	-	-	-	-	-	1289	ko01100//Metabolic pathways;ko01110//Biosynthesis of secondary metabolites;ko00360//Phenylalanine metabolism;ko00940//Phenylpropanoid biosynthesis	-	GO:0003824//catalytic activity;GO:0016209//antioxidant activity;GO:0043169//cation binding;GO:0046906//tetrapyrrole binding	GO:0044710;GO:0006950//response to stress	gi|351723793|ref|NP_001238315.1|/0/peroxidase precursor [Glycine max]
Glyma.09G022400	-	-	-	-	-	-	-	-	2.90683	0.00075	1409	ko01100//Metabolic pathways;ko01110//Biosynthesis of secondary metabolites;ko00360//Phenylalanine metabolism;ko00940//Phenylpropanoid biosynthesis	-	GO:0003824//catalytic activity;GO:0016209//antioxidant activity;GO:0043169//cation binding;GO:0046906//tetrapyrrole binding	GO:0044710;GO:0006950//response to stress	gi|255637517|gb|ACU19085.1|/0/unknown [Glycine max]
Glyma.09G023000	-	-	-	-	-	-	-	-	8.80154	1.13E-05	1449	ko01100//Metabolic pathways;ko01110//Biosynthesis of secondary metabolites;ko00360//Phenylalanine metabolism;ko00940//Phenylpropanoid biosynthesis	-	GO:0003824//catalytic activity;GO:0016209//antioxidant activity;GO:0043169//cation binding;GO:0046906//tetrapyrrole binding	GO:0044710;GO:0006950//response to stress	gi|734380812|gb|KHN22992.1|/0/Peroxidase C3 [Glycine soja]
Glyma.09G040600	-	-	-	-	-	-	-	-	8.06853	4.50E-05	936	-	-	GO:0003824//catalytic activity	GO:0006950//response to stress	gi|351726932|ref|NP_001237911.1|/1.98544e-110/uncharacterized protein LOC100305867 [Glycine max]
Glyma.09G048400	-5.5581	2.27E-17	-	-	-	-	-	-	-	-	945	ko01100//Metabolic pathways;ko01110//Biosynthesis of secondary metabolites;ko00360//Phenylalanine metabolism;ko00940//Phenylpropanoid biosynthesis	-	GO:0003824//catalytic activity;GO:0016209//antioxidant activity;GO:0043169//cation binding;GO:0046906//tetrapyrrole binding	GO:0044710;GO:0006950//response to stress	gi|947088498|gb|KRH37163.1|/0/hypothetical protein GLYMA_09G048400 [Glycine max]
Glyma.09G056300	-	-	-	-	-	-	-	-	1.39593	0.00325	3594	ko00190//Oxidative phosphorylation	GO:0031224//intrinsic component of membrane;GO:0005911//cell-cell junction;GO:0043231//intracellular	GO:0032550;GO:0017111//nucleoside-triphosphatase activity;GO:0043169//cation binding;GO:0008324//cation transmembrane transporter activity;GO:0046872//metal ion binding	GO:0009152//purine ribonucleotide biosynthetic process;GO:0006970//response to osmotic stress;GO:0009154//purine ribonucleotide catabolic process;GO:0006811//ion transport;GO:0015988//energy coupled proton transmembrane transport, against electrochemical gradient;GO:0010118//stomatal	gi|571476496|ref|XP_006586984.1|;gi|947088618|gb|KRH37283.1|/0;0/PREDICTED: plasma membrane ATPase 4-like [Glycine max];hypothetical protein GLYMA_09G056300 [Glycine max]

													membrane-bounded organelle		movement;GO:0001101//response to acid chemical;GO:0006950//response to stress	
Glyma.09G195800	-	-	-	-	-	-	-	-	1.1164	0.00922	1663	-	-	GO:0004721//phosphoprotein phosphatase activity	GO:0006796//phosphate-containing compound metabolic process;GO:0006950//response to stress;GO:0030522//intracellular receptor signaling pathway	gi|947090724|gb|KRH39389.1|;gi|356531319|ref|XP_003534225.1|/0;0/hypothetical protein GLYMA_09G195800 [Glycine max];PREDICTED: serine/threonine-protein phosphatase 7-like [Glycine max]
Glyma.09G197000	-	-	-	-	-	-	-	-	1.23284	0.00143	3119	ko04141//Protein processing in endoplasmic reticulum	-	GO:0032550;GO:0004672//protein kinase activity;GO:0004518//nuclease activity	GO:0090501//RNA phosphodiester bond hydrolysis;GO:0006396//RNA processing;GO:0006468//protein phosphorylation;GO:0030968//endoplasmic reticulum unfolded protein response;GO:0010033//response to organic substance;GO:0006950//response to stress	gi|571478408|ref|XP_003533406.2|;gi|947090746|gb|KRH39411.1|/0;0/PREDICTED: serine/threonine-protein kinase/endoribonuclease IRE1a-like isoform X1 [Glycine max];hypothetical protein GLYMA_09G197000 [Glycine max]
Glyma.09G202000	1.15195	0.003352	-	-	-	-	-	-	-	-	1905	ko00630//Glyoxylate and dicarboxylate metabolism;ko01100//Metabolic pathways;ko01110//Biosynthesis of secondary metabolites;ko00460//Cyanoamino acid metabolism;ko00670//One carbon pool by folate;ko00260//Glycine, serine and threonine metabolism	GO:0005840//ribosome;GO:0009532//plastid stroma;GO:0009526//plastid envelope;GO:0031976;GO:0005576//extracellular region;GO:0016020//membrane	GO:0008187//poly-pyrimidine tract binding;GO:0043168//anion binding;GO:0016742//hydroxymethyl-, formyl- and related transferase activity	GO:0009314//response to radiation;GO:0010038//response to metal ion;GO:0009069//serine family amino acid metabolic process;GO:0006730//one-carbon metabolic process;GO:0048511//rhythmic process;GO:0043094//cellular metabolic compound salvage;GO:0006950//response to stress	gi|734417401|gb|KHN38901.1|;gi|947090836|gb|KRH39501.1|;gi|947090837|gb|KRH39502.1|/0;0;0/Serine hydroxymethyltransferase, mitochondrial [Glycine soja];hypothetical protein GLYMA_09G202000 [Glycine max];hypothetical protein GLYMA_09G202000 [Glycine max]
Glyma.09G210900	-	-	-	-	-	-	-	-	-1.2504	0.00378	1894	ko01100//Metabolic pathways;ko00710//Carbon fixation in photosynthetic organisms	GO:0009532//plastid stroma;GO:0009526//plastid envelope;GO:0009534//chloroplast thylakoid;GO:0005576//extracellular region	GO:0032550;GO:0016301//kinase activity	GO:0044238//primary metabolic process;GO:0009617//response to bacterium;GO:0006796//phosphate-containing compound metabolic process;GO:0006950//response to stress	gi|356495988|ref|XP_003516852.1|/0/PREDICTED: phosphoribulokinase, chloroplastic [Glycine max]
Glyma.09G218600	-	-	-	-	-	-	-	-	2.90812	0.00085	2072	ko00906//Carotenoid biosynthesis	-	GO:0046906//tetrapyrrole binding;GO:0046914//transition metal ion binding;GO:0016709//oxidoreductase activity, acting on paired donors, with incorporation or reduction of molecular oxygen, NAD(P)H as one donor, and incorporation of one atom of oxygen;GO:0016491//oxid	GO:0010162//seed dormancy process;GO:0009620//response to fungus;GO:0009416//response to light stimulus;GO:0006714//sesquiterpenoid metabolic process;GO:0006950//response to stress;GO:0044710	gi|734348207|gb|KHN11785.1|;gi|947091113|gb|KRH39778.1|;gi|947091114|gb|KRH39779.1|;gi|947091112|gb|KRH39777.1|;gi|947091110|gb|KRH39775.1|/0;0;0;0;0/Abscisic acid 8'-hydroxylase 1 [Glycine soja] ;hypothetical protein GLYMA_09G218600 [Glycine max];hypothetical protein GLYMA_09G218600 [Glycine max];hypothetical protein GLYMA_09G218600 [Glycine max];hypothetical protein GLYMA_09G218600 [Glycine max]

														oreductase activity		
Glyma.10G001700	-	-	-	-	-	-	-	-	1.83199	1.06E-03	1812	ko04140//Regulation of autophagy	-	GO:0004672//protein kinase activity;GO:0032550	GO:0006796//phosphate-containing compound metabolic process;GO:0006950//response to stress;GO:0006464//cellular protein modification process;GO:0007154//cell communication	gi|947082911|gb|KRH31632.1|/0/hypothetical protein GLYMA_10G001700 [Glycine max]
Glyma.10G019000	-	-	-	-	-	-	-	-	2.03198	2.80E-06	6495	ko02010//ABC transporters	GO:0005911//cell-cell junction;GO:0031224//intrinsic component of membrane;GO:0044437	GO:0032550;GO:0017111//nucleoside-triphosphatase activity;GO:0015405;GO:0051183//vitamin transporter activity;GO:0016628//oxidoreductase activity, acting on the CH-CH group of donors, NAD or NADP as acceptor	GO:0006835//dicarboxylic acid transport;GO:0051707//response to other organism;GO:0009154//purine ribonucleotide catabolic process;GO:0006950//response to stress	gi|356536723|ref|XP_003536885.1|/0/PREDICTED: ABC transporter C family member 4-like [Glycine max]
Glyma.10G050800	-	-	-	-	-3.2757	0.00016	-	-	-	-	1493	ko01100//Metabolic pathways;ko01110//Biosynthesis of secondary metabolites;ko00360//Phenylalanine metabolism;ko00940//Phenylpropanoid biosynthesis	GO:0044444	GO:0046906//tetrapyrrole binding;GO:0016209//antioxidant activity;GO:0043169//cation binding;GO:0003824//catalytic activity	GO:0006950//response to stress;GO:0044710;GO:0009620//response to fungus	gi|356537521|ref|XP_003537275.1|/0/PREDICTED: peroxidase 21 [Glycine max]
Glyma.10G058500	-	-	-	-	-	-	-	-	-1.1006	0.00133	1495	ko03015//mRNA surveillance pathway;ko03040//Spliceosome	GO:0009526//plastid envelope;GO:0009534//chloroplast thylakoid	GO:0003676//nucleic acid binding;GO:0036094//small molecule binding	GO:0006950//response to stress	gi|356533254|ref|XP_003535181.1|/4.03851e-170/PREDICTED: 29 kDa ribonucleoprotein A, chloroplastic [Glycine max]
Glyma.10G127800	-	-	-	-	-	-	-1.132	0.00019	-	-	2177	ko03018//RNA degradation	GO:0005840//ribosome;GO:0009532//plastid stroma;GO:0009526//plastid envelope;GO:0044437;GO:0044429;GO:0016020//membrane	GO:0032550;GO:0046914//transition metal ion binding	GO:0010038//response to metal ion;GO:0006457//protein folding;GO:0006950//response to stress	gi|734343222|gb|KHN10297.1|/0/Chaperonin CPN60-2, mitochondrial [Glycine soja]
Glyma.10G139500	-2.3686	8.16E-19	-2.5732	1.67E-12	-2.4054	3.41E-08	-1.8652	0.0001	-	-	1869	-	GO:0044436;GO:0044434;GO:0009579//thylakoid	GO:0016853//isomerase activity	GO:0044267//cellular protein metabolic process;GO:0009628//response to abiotic stimulus;GO:1901700;GO:0006950//response to stress;GO:0010033//response to organic substance	gi|947084964|gb|KRH33685.1|;gi|947084962|gb|KRH33683.1|;gi|356534945|ref|XP_003536011.1|/1.08891e-130;0;0/hypothetical protein GLYMA_10G139500 [Glycine max];hypothetical protein GLYMA_10G139500 [Glycine max];PREDICTED: peptidyl-prolyl cis-trans isomerase, chloroplastic [Glycine max]
Glyma.10G172600	-	-	-	-	-	-	-	-	4.92439	2.02E-07	2373	ko00564//Glycerophospholipid metabolism	GO:0031012//extracellular matrix	GO:0003824//catalytic activity	GO:0009059//macromolecule biosynthetic process;GO:0009688//abscisic acid biosynthetic process;GO:0006950//respons	gi|947085533|gb|KRH34254.1|;gi|571483425|ref|XP_003535395.2|/0;0/hypothetical protein GLYMA_10G172600 [Glycine max];PREDICTED: uncharacterized protein LOC100811400 [Glycine max]

20 × 20

															e to stress	
Glyma. 10G193 200	-	-	-	-	-	-	-1.1061	0.0008	-	-	2162	ko03018//RNA degradation	GO:0005840//ribosome;GO:0009532//plastid stroma;GO:0009526//plastid envelope;GO:0044437;GO:0044429;GO:0016020//membrane	GO:0032550;GO:0046914//transition metal ion binding	GO:0010038//response to metal ion;GO:0006457//protein folding;GO:0006950//response to stress	gi|734398893|gb|KHN30688.1|/0/Chaperonin CPN60-2, mitochondrial [Glycine soja]
Glyma. 10G222 400	-	-	-	-	-	-	-	-	1.82533	0.00098	1050	ko01100//Metabolic pathways;ko01110//Biosynthesis of secondary metabolites;ko00360//Phenylalanine metabolism;ko00940//Phenylpropanoid biosynthesis	GO:0005911//cell-cell junction;GO:0005618//cell wall	GO:0003824//catalytic activity;GO:0016209//antioxidant activity;GO:0043169//cation binding;GO:0046906//tetrapyrrole binding	GO:0044710;GO:0006950//response to stress	gi|947086384|gb|KRH35105.1|/0/hypothetical protein GLYMA_10G222400, partial [Glycine max]
Glyma. 10G222 500	-	-	2.40311	7.89E-07	-	-	2.37907	1.98E-07	-	-	1415	ko01100//Metabolic pathways;ko01110//Biosynthesis of secondary metabolites;ko00360//Phenylalanine metabolism;ko00940//Phenylpropanoid biosynthesis	-	GO:0003824//catalytic activity;GO:0016209//antioxidant activity;GO:0043169//cation binding;GO:0046906//tetrapyrrole binding	GO:0044710;GO:0006950//response to stress	gi|356535764|ref|XP_003536413.1|/0/PREDICTED: peroxidase 12 [Glycine max]
Glyma. 10G257 900	-	-	-	-	-	-	-	-	1.81463	0.00142	1392	-	-	-	GO:0009628//response to abiotic stimulus;GO:0006950//response to stress	gi|356536109|ref|XP_003536582.1|/2.10555e-136/PREDICTED: zinc finger protein ZAT10-like [Glycine max]
Glyma. 10G268 500	-	-	-	-	-	-	-	-	1.4185	0.00012	1884	ko00051//Fructose and mannose metabolism;ko00030//Pentose phosphate pathway;ko00010//Glycolysis / Gluconeogenesis;ko01100//Metabolic pathways;ko01110//Biosynthesis of secondary metabolites;ko00710//Carbon fixation in photosynthetic organisms	GO:0031976;GO:0009532//plastid stroma	GO:0016832//aldehyde-lyase activity	GO:0006950//response to stress;GO:0010038//response to metal ion;GO:0006091//generation of precursor metabolites and energy	gi|947087127|gb|KRH35848.1|;gi|571484814|ref|XP_006589659.1|/0;0/hypothetical protein GLYMA_10G268500 [Glycine max];PREDICTED: probable fructose-bisphosphate aldolase 3, chloroplastic [Glycine max]
Glyma. 11G024 100	-1.5635	2.21E-06	-	-	-	-	-	-	-1.0682	0.00486	924	ko00480//Glutathione metabolism;ko00590//Arachidonic acid metabolism	-	GO:0004601//peroxidase activity	GO:0006950//response to stress;GO:0044710	gi|947079142|gb|KRH27931.1|/8.36502e-121/hypothetical protein GLYMA_11G024100 [Glycine max]
Glyma. 11G027 700	-1.7055	8.96E-12	-	-	-	-	-	-	4.20015	4.44E-17	1311	ko00941//Flavonoid biosynthesis;ko01100//Metabolic pathways;ko01110//Biosynthesis of secondary metabolites	-	GO:0016705//oxidoreductase activity, acting on paired donors, with incorporation or reduction of molecular oxygen	GO:0009699//phenylpropanoid biosynthetic process;GO:0006950//response to stress;GO:0001101//response to acid chemical;GO:0006996//organelle organization	gi|359807474|ref|NP_001240884.1|/0/leucoanthocyanidin dioxygenase-like [Glycine max]

20 × 20

Glyma.11G035200	-1.0134	5.17E-06	-	-	-	-	-	-	1.68646	0.00065	2408	ko00071//Fatty acid metabolism;ko01040//Biosynthesis of unsaturated fatty acids;ko01100//Metabolic pathways;ko04146//Peroxisome;ko00592//alpha-Linolenic acid metabolism	GO:0042579//microbody;GO:0005911//cell-cell junction	GO:0016634//oxidoreductase activity, acting on the CH-CH group of donors, oxygen as acceptor;GO:0000166//nucleotide binding	GO:0009625//response to insect;GO:0010038//response to metal ion;GO:0009694//jasmonic acid metabolic process;GO:0048229//gametophyte development;GO:0009062//fatty acid catabolic process;GO:0006950//response to stress	gi|947079359|gb|KRH28148.1|;gi|734408808|gb|KHN34915.1|/0;0/hypothetical protein GLYMA_11G035200 [Glycine max];Peroxisomal acyl-coenzyme A oxidase 1 [Glycine soja]
Glyma.11G039400	-	-	-	-	-2.8434	2.02E-05	-	-	-	-	2028	ko00500//Starch and sucrose metabolism;ko01100//Metabolic pathways	GO:0009532//plastid stroma	GO:0016160//amylase activity	GO:0006950//response to stress;GO:0005976//polysaccharide metabolic process;GO:0000023//maltose metabolic process	gi|734408776|gb|KHN34883.1|/0/Beta-amylase 3, chloroplastic [Glycine soja]
Glyma.11G078300	-	-	-	-	-	-	-	-	3.1502	0.00035	4585	ko04075//Plant hormone signal transduction	-	GO:0004673//protein histidine kinase activity;GO:0032550;GO:0004888//transmembrane signaling receptor activity	GO:0003002//regionalization;GO:0006796//phosphate-containing compound metabolic process;GO:0007165//signal transduction;GO:0044702//single organism reproductive process;GO:0006950//response to stress	gi|571487712|ref|XP_006590729.1|;gi|947080024|gb|KRH28813.1|/0;0/PREDICTED: histidine kinase 1-like isoform X2 [Glycine max];hypothetical protein GLYMA_11G078300 [Glycine max]
Glyma.11G080300	-	-	-	-	-	-	-	-	2.73761	0.00021	1522	ko01100//Metabolic pathways;ko01110//Biosynthesis of secondary metabolites;ko00360//Phenylalanine metabolism;ko00940//Phenylpropanoid biosynthesis	-	GO:0003824//catalytic activity;GO:0016209//antioxidant activity;GO:0043169//cation binding;GO:0046906//tetrapyrrole binding	GO:0044710;GO:0006950//response to stress	gi|947080055|gb|KRH28844.1|/0/hypothetical protein GLYMA_11G080300 [Glycine max]
Glyma.11G107200	-	-	-	-	-	-	-	-	1.80125	3.89E-04	1304	ko00053//Ascorbate and aldarate metabolism;ko00480//Glutathione metabolism	GO:0005911//cell-cell junction;GO:0009526//plastid envelope;GO:0044437	GO:0046906//tetrapyrrole binding;GO:0016209//antioxidant activity	GO:0006950//response to stress;GO:0044710	gi|356538634|ref|XP_003537806.1|/0/PREDICTED: L-ascorbate peroxidase 3, peroxisomal [Glycine max]
Glyma.11G111100	-	-	-	-	-	-	-	-	-1.1788	0.00358	1962	ko00051//Fructose and mannose metabolism;ko00030//Pentose phosphate pathway;ko00010//Glycolysis / Gluconeogenesis;ko01100//Metabolic pathways;ko01110//Biosynthesis of secondary metabolites;ko00710//Carbon fixation in photosynthetic organisms	GO:0005840//ribosome;GO:0009532//plastid stroma;GO:0009526//plastid envelope;GO:0031976;GO:0005576//extracellular region;GO:0044436	GO:0016832//aldehyde-lyase activity	GO:0010038//response to metal ion;GO:0001101//response to acid chemical;GO:0006091//generation of precursor metabolites and energy;GO:0006950//response to stress	gi|356538694|ref|XP_003537836.1|/0/PREDICTED: fructose-bisphosphate aldolase 1, chloroplastic [Glycine max]
Glyma.11G153800	-	-	-	-	-	-	-	-	-1.2154	0.00099	1575	-	-	GO:0046872//metal ion binding;GO:0005543//phospholipid binding;GO:0043167//ion binding	GO:0006970//response to osmotic stress;GO:0006950//response to stress;GO:0009628//response to abiotic stimulus	gi|571486420|ref|XP_006590331.1|;gi|947081231|gb|KRH30020.1|;gi|947081232|gb|KRH30021.1|/0;3.81041e-174;0/PREDICTED: uncharacterized protein LOC100806472 isoform X1 [Glycine max];hypothetical protein GLYMA_11G153800 [Glycine max];hypothetical protein GLYMA_11G153800 [Glycine max]

20 × 20

Glyma.11G178100	-	-	-	-	-	-	-1.3773	0.00135	-	-	868	-	GO:0031981//nuclear lumen;GO:0009536//plastid;GO:0044437;GO:0000785//chromatin;GO:0005911//cell-cell junction;GO:0016020//membrane	GO:0003676//nucleic acid binding;GO:0046983//protein dimerization activity	GO:0034728//nucleosome organization;GO:0006950//response to stress	gi|475622287|gb|EMT31824.1|/1.35134e-52/Histone H4 [Aegilops tauschii]
Glyma.11G212900	-	-	-	-	-	-	-	-	8.84592	1.57E-38	1005	ko00480//Glutathione metabolism	GO:0000325//plant-type vacuole	GO:0043167//ion binding;GO:0016765//transferase activity, transferring alkyl or aryl (other than methyl) groups	GO:0006950//response to stress;GO:0009962//regulation of flavonoid biosynthetic process	gi|959092507|ref|NP_001304475.1|/1.32038e-156/glutathione S-transferase F11-like [Glycine max]
Glyma.11G247600	1.56653	1.40E-10	-	-	-	-	-	-	1.77344	1.23E-05	1578	ko00010//Glycolysis / Gluconeogenesis;ko01100//Metabolic pathways;ko01110//Biosynthesis of secondary metabolites	GO:0030312//external encapsulating structure;GO:0031981//nuclear lumen;GO:0009536//plastid;GO:0005911//cell-cell junction;GO:0016020//membrane	GO:0046914//transition metal ion binding;GO:0016903//oxidoreductase activity, acting on the aldehyde or oxo group of donors;GO:0000166//nucleotide binding	GO:0010038//response to metal ion;GO:0019318//hexose metabolic process;GO:0009617//response to bacterium;GO:0006950//response to stress	gi|359806082|ref|NP_001241184.1|/0/uncharacterized protein LOC100807342 [Glycine max]
Glyma.11G252900	-	-	-	-	-	-	-	-	-2.371	0.00025	1685	ko03010//Ribosome	GO:0009532//plastid stroma	GO:0003676//nucleic acid binding;GO:0003824//catalytic activity	GO:0006950//response to stress	gi|947082755|gb|KRH31544.1|;gi|356538073|ref|XP_003537529.1|/0;0/hypothetical protein GLYMA_11G252900 [Glycine max];PREDICTED: 30S ribosomal protein S1 homolog isoform X1 [Glycine max]
Glyma.12G037400	-	-	-	-	-	-	-	-	-1.0625	0.00433	1707	ko00051//Fructose and mannose metabolism;ko00030//Pentose phosphate pathway;ko00010//Glycolysis / Gluconeogenesis;ko01100//Metabolic pathways;ko01110//Biosynthesis of secondary metabolites;ko00710//Carbon fixation in photosynthetic organisms	GO:0005840//ribosome;GO:0009532//plastid stroma;GO:0009526//plastid envelope;GO:0031976;GO:0005576//extracellular region;GO:0044436	GO:0016832//aldehyde-lyase activity	GO:0010038//response to metal ion;GO:0001101//response to acid chemical;GO:0006091//generation of precursor metabolites and energy;GO:0006950//response to stress	gi|734387664|gb|KHN25427.1|/0/Fructose-bisphosphate aldolase 1, chloroplastic [Glycine soja]
Glyma.12G064000	-	-	-	-	-	-	-	-	1.3953	0.00866	2285	ko04144//Endocytosis;ko04141//Protein processing in endoplasmic reticulum;ko03040//Spliceosome	GO:0005840//ribosome;GO:0030312//external encapsulat	GO:0032550;GO:0019899//enzyme binding	GO:0051707//response to other organism;GO:0010038//response to metal ion;GO:0009628//response to abiotic	gi|734402634|gb|KHN32117.1|/0/Heat shock cognate 70 kDa protein [Glycine soja]

20 × 20

													ing structure;GO:0009526//plastid envelope;GO:0044437;GO:0005911//cell-cell junction;GO:0005576//extracellular region;GO:0016020//membrane		stimulus;GO:0006950//response to stress	
Glyma.13G028200	-	-	3.83427	1.88E-21	2.99907	6.70E-06	1.79323	0.00016	-	-	816	ko00195//Photosynthesis;ko01100//Metabolic pathways	GO:0009521;GO:0031224//intrinsic component of membrane;GO:0009534//chloroplast thylakoid	GO:0046914//transition metal ion binding;GO:0009055//electron carrier activity;GO:0003824//catalytic activity	GO:0006950//response to stress;GO:0009767//photosynthetic electron transport chain	gi|947069046|gb|KRH17937.1|/0/hypothetical protein GLYMA_13G028200, partial [Glycine max]
Glyma.13G058600	-	-	-	-	-	-	-	-	1.47973	4.86E-05	3288	-	GO:0009532//plastid stroma	GO:0016462//pyrophosphatase activity;GO:0032550	GO:0006950//response to stress;GO:0009657//plastid organization;GO:0051604//protein maturation	gi|734331638|gb|KHN07152.1|/0/Chaperone protein ClpB3, chloroplastic [Glycine soja]
Glyma.13G067900	-	-	-	-	-3.5906	2.72E-05	-	-	3.05666	0.00028	1651	ko00500//Starch and sucrose metabolism;ko01110//Biosynthesis of secondary metabolites;ko00520//Amino sugar and nucleotide sugar metabolism	-	GO:0016740//transferase activity	GO:0006950//response to stress	gi|356550372|ref|XP_003543561.1|/0/PREDICTED: probable galacturonosyltransferase-like 10 [Glycine max]
Glyma.13G088100	-	-	-	-	-	-	-	-	2.25151	0.0007	1919	-	-	-	GO:0009987//cellular process;GO:0009628//response to abiotic stimulus;GO:0006950//response to stress	gi|356548125|ref|XP_003542454.1|/2.69255e-175/PREDICTED: ethylene-responsive transcription factor RAP2-4 [Glycine max]
Glyma.13G112500	-	-	-	-	-	-	-	-	-1.5724	0.0004	1219	-	GO:0009532//plastid stroma;GO:0009526//plastid envelope;GO:0009534//chloroplast thylakoid;GO:0005576//extracellular region	GO:0032550;GO:0046914//transition metal ion binding	GO:0010038//response to metal ion;GO:0044267//cellular protein metabolic process;GO:0051353//positive regulation of oxidoreductase activity;GO:0006950//response to stress	gi|571497607|ref|XP_006593958.1|/1.45306e-162/PREDICTED: 20 kDa chaperonin, chloroplastic [Glycine max]
Glyma.13G147000	-	-	-	-	-	-	-	-	-1.7739	8.30E-06	687	-	GO:0031981//nuclear lumen;GO:0009536//plastid;GO	GO:0003676//nucleic acid binding;GO:0046983//protein dimerization activity	GO:0034728//nucleosome organization;GO:0006950//response to stress	gi|475622287|gb|EMT31824.1|/1.35134e-52/Histone H4 [Aegilops tauschii]

													:0044437;GO:0000785//chromatin;GO:0005911//cell-cell junction;GO:0016020//membrane			
Glyma.13G195200	-	-	-	-	-	-	-	-	1.70917	5.94E-05	5403	-	GO:0043231//intracellular membrane-bounded organelle	GO:0046914//transition metal ion binding;GO:0016301//kinase activity;GO:0032550;GO:0005515//protein binding;GO:0019787//ubiquitin-like protein transferase activity;GO:0050136//NADH dehydrogenase (quinone) activity	GO:0040007//growth;GO:1902582;GO:0032446//protein modification by small protein conjugation;GO:0006950//response to stress;GO:0009738//abscisic acid-activated signaling pathway;GO:0046903//secretion;GO:0022904//respiratory electron transport chain	gi|947071810|gb|KRH20701.1|/0/hypothetical protein GLYMA_13G195200 [Glycine max]
Glyma.13G196600	-	-	-	-	-	-	-2.2378	8.30E-06	-2.2229	0.0008	1567	-	GO:0009532//plastid stroma;GO:0016020//membrane;GO:0009536//plastid	GO:0003824//catalytic activity	GO:0009617//response to bacterium;GO:0006970//response to osmotic stress;GO:0044710;GO:0006950//response to stress	gi|571495833|ref|XP_006593398.1|;gi|351722504|ref|NP_001237502.1|/4.24891e-129;1.45189e-127/PREDICTED: uncharacterized protein LOC100527538 isoform X1 [Glycine max];uncharacterized protein LOC100527538 [Glycine max]
Glyma.13G243100	-	-	-	-	-	-	-	-	-1.3442	0.00026	1716	ko03010//Ribosome	GO:0015934//large ribosomal subunit;GO:0016020//membrane;GO:0044437;GO:0009536//plastid;GO:0031981//nuclear lumen	GO:0005198//structural molecule activity	GO:0006950//response to stress;GO:0010467//gene expression	gi|734337622|gb|KHN08559.1|/1.12516e-80/60S ribosomal protein L26-1 [Glycine soja]
Glyma.13G301600	2.80312	2.63E-10	-	-	-	-	-	-	-	-	720	-	GO:0043231//intracellular membrane-bounded organelle	-	GO:0006950//response to stress	gi|351727659|ref|NP_001238192.1|/1.19665e-49/uncharacterized protein LOC100305628 [Glycine max]
Glyma.13G325900	-	-	-	-	-	-	-	-	-1.1247	0.00165	1652	-	GO:0031224//intrinsic component of membrane;GO:0005911//cell-cell junction	GO:0005372//water transmembrane transporter activity	GO:0055085//transmembrane transport;GO:0001101//response to acid chemical;GO:0048588//developmental cell growth;GO:0042044//fluid transport;GO:0006950//response to stress;GO:0051234//establishment of localization	gi|162457846|ref|NP_001105639.1|;gi|947073999|gb|KRH22890.1|/0;3.82382e-137/aquaporin PIP2-7 [Zea mays];hypothetical protein GLYMA_13G325900 [Glycine max]
Glyma.13G367000	-	-	-	-	-	-	-	-	-1.1564	0.00288	1219	ko00190//Oxidative phosphorylation;ko01100//Metabolic pathways	GO:0005746//mitochondrial respiratory chain	GO:0046914//transition metal ion binding;GO:0051536//iron-sulfur cluster binding;GO:0003824//catalytic activity	GO:0006950//response to stress;GO:0044710	gi|947074709|gb|KRH23600.1|;gi|947074707|gb|KRH23598.1|;gi|947074708|gb|KRH23599.1|/1.886e-169;0;0/hypothetical protein GLYMA_13G367000 [Glycine max];hypothetical protein GLYMA_13G367000 [Glycine

																max];hypothetical protein GLYMA_13G367000 [Glycine max]
Glyma. 14G061 200	-	-	-	-	-3.4096	0.00087	-	-	3.54047	1.14E-0 5	1734	-	-	-	GO:0006950//response to stress	gi|571507539|ref|XP_003545237.2|/0/PREDICTED: nematode resistance protein-like HSPRO2 [Glycine max]
Glyma. 14G078 600	-	-	-	-	-	-	1.27601	5.93E-0 4	2.69163	2.01E-0 7	1720	ko01100//Metabolic pathways;ko00592//alpha-Linolenic acid metabolism	GO:0009526//plastid envelope; GO:0009534//chloro plast thylakoid	GO:0046906//tetrapyrrole binding;GO:0046914//transition metal ion binding;GO:0016491//oxidoreductase activity;GO:0016836//hydro-lyase activity	GO:0051707//response to other organism;GO:0006631//fatty acid metabolic process;GO:0001101//response to acid chemical;GO:0006950//response to stress	gi|947066131|gb|KRH15274.1|/0/hypothetical protein GLYMA_14G078600 [Glycine max]
Glyma. 14G084 700	-1.636	8.73E-1 0	-	-	-	-	-	-	-	-	1592	-	GO:0043231//intracellular membrane-bounded organelle	GO:0003676//nucleic acid binding;GO:0001071//nucleic acid binding transcription factor activity	GO:0006950//response to stress;GO:0006351//transcription, DNA-templated	gi|351724471|ref|NP_001235779.1|/2.00806e-130/dehydration responsive element binding protein [Glycine max]
Glyma. 14G088 300	-	-	-1.6406	0.00031 1	-3.5117	1.76E-0 5	-	-	2.38448	4.30E-0 5	1097	-	-	GO:0043169//cation binding	GO:0050789//regulation of biological process;GO:0001101//response to acid chemical;GO:0009719;GO:0009628//response to abiotic stimulus;GO:0007275//multicellular organismal development;GO:0006950//response to stress;GO:0010033//response to organic substance	gi|390517035|ref|NP_001254622.1|/2.3512e-131/zinc finger protein ZAT10-like [Glycine max]
Glyma. 14G111 800	-	-	-	-	-	-	-1.4101	9.21E-0 5	-	-	2504	ko00270//Cysteine and methionine metabolism;ko00330//Arginine and proline metabolism;ko00960//Tropane, piperidine and pyridine alkaloid biosynthesis;ko01100//Metabolic pathways;ko01110//Biosynthesis of secondary metabolites;ko00360//Phenylalanine metabolism;ko00250//Alanine, aspartate and glutamate metabolism;ko00400//Phenylalanine, tyrosine and tryptophan biosynthesis;ko00950//Isoquinoline alkaloid biosynthesis;ko00350//Tyrosine metabolism	GO:0009532//plastid stroma;GO:0009526//plastid envelope; GO:0044429;GO:0005576//extracellular region	GO:0043168//anion binding;GO:0070546	GO:0010038//response to metal ion;GO:0043648//dicarboxylic acid metabolic process;GO:0009064//glutamine family amino acid metabolic process;GO:0009066;GO:0006950//response to stress	gi|734321388|gb|KHN04158.1|;gi|947066652|gb|KRH15795.1|/0;0/Aspartate aminotransferase P2, mitochondrial [Glycine soja] ;hypothetical protein GLYMA_14G111800 [Glycine max]
Glyma. 14G171 500	-	-	-	-	-1.8387	5.17E-0 5	-	-	-	-	1795	-	-	-	GO:0009987//cellular process;GO:0009628//response to abiotic stimulus;GO:0006950//response to stress	gi|571510835|ref|XP_006596336.1|/6.02977e-159/PREDICTED: ethylene-responsive transcription factor RAP2-4-like [Glycine max]
Glyma. 14G176 700	-	-	-	-	-	-	-	-	2.10905	4.99E-0 3	1728	ko04075//Plant hormone signal transduction	GO:0043231//intracellular	GO:0004672//protein kinase activity;GO:0032550	GO:0006796//phosphate-containing compound metabolic process;GO:0009628//respons	gi|734344379|gb|KHN10464.1|/0/Serine/threonine-protein kinase SAPK2 [Glycine soja]

20 × 20

													membrane -bounded organelle		e to abiotic stimulus;GO:0006950//response to stress;GO:0006464//cellular protein modification process	
Glyma.14G190800	-	-	-	-	-	-	-	-	-1.2147	0.00602	1491	-	GO:0031981//nuclear lumen;GO:0009536//plastid;GO:0044437;GO:0000785//chromatin;GO:0005911//cell-cell junction;GO:0016020//membrane	GO:0003676//nucleic acid binding;GO:0046983//protein dimerization activity	GO:0034728//nucleosome organization;GO:0006950//response to stress	gi|475622287|gb|EMT31824.1|/1.35134e-52/Histone H4 [Aegilops tauschii]
Glyma.14G195200	-	-	-	-	-3.7808	3.31E-05	-	-	3.02087	0.00017	1825	ko04075//Plant hormone signal transduction	GO:0043231//intracellular membrane -bounded organelle	GO:0043169//cation binding;GO:0004721//phosphoprotein phosphatase activity	GO:0009620//response to fungus;GO:0006464//cellular protein modification process;GO:0009755//hormone-mediated signaling pathway;GO:0006950//response to stress	gi|356553088|ref|XP_003544890.1|;gi|947067914|gb|KRH17057.1|/0;5.22623e-137/PREDICTED: probable protein phosphatase 2C 25 [Glycine max];hypothetical protein GLYMA_14G195200 [Glycine max]
Glyma.14G201700	1.10966	0.004665	-	-	-	-	-	-	-	-	1549	ko01100//Metabolic pathways;ko01110//Biosynthesis of secondary metabolites;ko00360//Phenylalanine metabolism;ko00940//Phenylpropanoid biosynthesis	-	GO:0003824//catalytic activity;GO:0016209//antioxidant activity;GO:0043169//cation binding;GO:0046906//tetrapyrrole binding	GO:0044710;GO:0006950//response to stress	gi|947068000|gb|KRH17143.1|/0/hypothetical protein GLYMA_14G201700 [Glycine max]
Glyma.15G006400	-	-	-	-	-	-	-	-	-1.1887	0.00164	1964	ko00190//Oxidative phosphorylation;ko01100//Metabolic pathways	GO:0005746//mitochondrial respiratory chain	GO:0046914//transition metal ion binding;GO:0051536//iron-sulfur cluster binding;GO:0003824//catalytic activity	GO:0006950//response to stress;GO:0044710	gi|947074707|gb|KRH23598.1|/0/hypothetical protein GLYMA_13G367000 [Glycine max]
Glyma.15G034000	-	-	-	-	-	-	-	-	1.3977	0.00982	3882	ko00230//Purine metabolism;ko00232//Caffeine metabolism;ko01100//Metabolic pathways;ko01110//Biosynthesis of secondary metabolites;ko04146//Peroxisome	GO:0044444	GO:0046914//transition metal ion binding;GO:0051536//iron-sulfur cluster binding;GO:0016616//oxidoreductase activity, acting on the CH-OH group of donors, NAD or NADP as acceptor;GO:0000166//nucleotide binding	GO:0006144//purine nucleobase metabolic process;GO:0006950//response to stress;GO:0006801//superoxide metabolic process	gi|947060936|gb|KRH10197.1|/0/hypothetical protein GLYMA_15G034000 [Glycine max]
Glyma.15G052700	-	-	-	-	-	-	-	-	4.56792	0.00383	1267	ko01100//Metabolic pathways;ko01110//Biosynthesis of secondary metabolites;ko00360//Phenylalanine metabolism;ko00940//Phenylpropanoid biosynthesis	GO:0043231//intracellular membrane -bounded organelle; GO:0030312//external encapsulating structure; GO:00059	GO:0043169//cation binding;GO:0046906//tetrapyrrole binding;GO:0016209//antioxidant activity;GO:0003824//catalytic activity	GO:0009620//response to fungus;GO:0009698//phenylpropanoid metabolic process;GO:0042743//hydrogen peroxide metabolic process;GO:0006950//response to stress	gi|955366031|ref|XP_014622898.1|/0/PREDICTED: cationic peroxidase 2 [Glycine max]

													11//cell-cell junction;GO:0005576//extracellular region;GO:0016020//membrane			
Glyma.15G088000	-	-	-	-	-	-	1.6912	0.00012	2.7109	1.87E-06	2418	ko04144//Endocytosis;ko04141//Protein processing in endoplasmic reticulum;ko03040//Spliceosome	GO:0030312//external encapsulating structure;GO:0009536//plastid;GO:0016020//membrane	GO:0032550;GO:0016891//endoribonuclease activity, producing 5'-phosphomonoesters	GO:0051707//response to other organism;GO:0090501//RNA phosphodiester bond hydrolysis;GO:0006950//response to stress	gi|947061823|gb|KRH11084.1|/0/hypothetical protein GLYMA_15G088000 [Glycine max]
Glyma.15G100700	-	-	-	-	-	-	-	-	-1.516	8.64E-05	833	-	-	GO:0016671//oxidoreductase activity, acting on a sulfur group of donors, disulfide as acceptor	GO:0044710;GO:0044267//cellular protein metabolic process;GO:0006950//response to stress	gi|351724743|ref|NP_001237068.1|/4.00215e-99/uncharacterized protein LOC100305558 [Glycine max]
Glyma.15G124500	-	-	-	-	-	-	-	-	-2.3622	0.0076	1927	-	GO:0016020//membrane;GO:0031224//intrinsic component of membrane	GO:0005515//protein binding	GO:0050896//response to stimulus;GO:0006950//response to stress	gi|947062437|gb|KRH11698.1|;gi|571518103|ref|XP_006597646.1|/0;0/hypothetical protein GLYMA_15G124500 [Glycine max];PREDICTED: MLO-like protein 13 [Glycine max]
Glyma.15G128700	-	-	-	-	-	-	-	-	2.99002	0.00284	1464	ko01100//Metabolic pathways;ko01110//Biosynthesis of secondary metabolites;ko00360//Phenylalanine metabolism;ko00940//Phenylpropanoid biosynthesis	-	GO:0003824//catalytic activity;GO:0016209//antioxidant activity;GO:0043169//cation binding;GO:0046906//tetrapyrrole binding	GO:0044710;GO:0006950//response to stress	gi|734356908|gb|KHN14345.1|/0/Peroxidase 22 [Glycine soja]
Glyma.15G132600	-	-	-	-	-	-	-	-	1.5412	0.01003	2044	ko00908//Zeatin biosynthesis	GO:0030312//external encapsulating structure; GO:0005911//cell-cell junction	GO:0046993;GO:0016616//oxidoreductase activity, acting on the CH-OH group of donors, NAD or NADP as acceptor;GO:0000166//nucleotide binding	GO:0044710;GO:0000741//karyogamy;GO:0003006//developmental process involved in reproduction;GO:0006950//response to stress	gi|356555926|ref|XP_003546280.1|/0/PREDICTED: inactive tetrahydrocannabinolic acid synthase-like [Glycine max]
Glyma.15G132800	-	-	-	-	-	-	-	-	3.43451	8.07E-05	2173	-	GO:0005911//cell-cell junction;GO:0005618//cell wall;GO:0043231//intracellular membrane-bounded organelle; GO:0044444;GO:0005576//extracellular	GO:0016616//oxidoreductase activity, acting on the CH-OH group of donors, NAD or NADP as acceptor;GO:0000166//nucleotide binding	GO:0006950//response to stress;GO:0044710	gi|734356870|gb|KHN14307.1|/0/Reticuline oxidase-like protein [Glycine soja]

20 × 20

													region;GO:0016020//membrane			
Glyma.15G162600	-	-	-	-	-	-	-	-	1.31971	0.00704	3504	ko00190//Oxidative phosphorylation	GO:0005911//cell-cell junction;GO:0043231//intracellular membrane-bounded organelle;GO:0031224//intrinsic component of membrane	GO:0032550;GO:0017111//nucleoside-triphosphatase activity;GO:0046872//metal ion binding;GO:0008324//cation transmembrane transporter activity	GO:0015988//energy coupled proton transmembrane transport, against electrochemical gradient;GO:0009152//purine ribonucleotide biosynthetic process;GO:0010118//stomatal movement;GO:0001101//response to acid chemical;GO:0009154//purine ribonucleotide catabolic process;GO:0006950//response to stress	gi|356556196|ref|XP_003546412.1|/0/PREDICTED: plasma membrane ATPase 4-like [Glycine max]
Glyma.15G217100	2.88696	3.31E-08	-	-	-	-	-	-	-	-	823	-	-	-	GO:0006950//response to stress	gi|351726383|ref|NP_001235333.1|/4.13848e-109/uncharacterized protein LOC100306201 [Glycine max]
Glyma.15G218900	-	-	-	-	-	-	-	-	5.54997	0.00036	848	-	-	-	GO:0006950//response to stress	gi|734420599|gb|KHN40931.1|/6.60831e-110/MLP-like protein 43 [Glycine soja]
Glyma.15G261900	-	-	-	-	-	-	-	-	-1.0382	0.00211	2027	ko00010//Glycolysis / Gluconeogenesis;ko01100//Metabolic pathways;ko01110//Biosynthesis of secondary metabolites;ko00710//Carbon fixation in photosynthetic organisms	GO:0030312//external encapsulating structure;GO:0009532//plastid stroma;GO:0009526//plastid envelope;GO:0005576//extracellular region	GO:0032550;GO:0016301//kinase activity	GO:0010038//response to metal ion;GO:0006091//generation of precursor metabolites and energy;GO:0006796//phosphate-containing compound metabolic process;GO:0006950//response to stress	gi|947064495|gb|KRH13756.1|;gi|356557028|ref|XP_003546820.1|/0;0/hypothetical protein GLYMA_15G261900 [Glycine max];PREDICTED: phosphoglycerate kinase, cytosolic [Glycine max]
Glyma.15G274300	-	-	-	-	-	-	-	-	-2.6561	0.00072	720	-	GO:0009536//plastid	-	GO:0006950//response to stress	gi|734419109|gb|KHN39933.1|/1.97483e-113/MLP-like protein 28 [Glycine soja]
Glyma.16G044900	-	-	-	-	-	-	-	-	-1.2431	0.00194	1619	ko01100//Metabolic pathways;ko00710//Carbon fixation in photosynthetic organisms	GO:0009526//plastid envelope;GO:0009534//chloroplast thylakoid;GO:0005576//extracellular region	GO:0016620//oxidoreductase activity, acting on the aldehyde or oxo group of donors, NAD or NADP as acceptor;GO:0000166//nucleotide binding	GO:0009314//response to radiation;GO:0019318//hexose metabolic process;GO:0034285;GO:0006950//response to stress	gi|356561064|ref|XP_003548805.1|/0/PREDICTED: glyceraldehyde-3-phosphate dehydrogenase A, chloroplastic [Glycine max]
Glyma.16G055900	-	-	-	-	-	-	2.35556	0.00016	-	-	1382	ko01100//Metabolic pathways;ko01110//Biosynthesis of secondary metabolites;ko00360//Phenylalanine metabolism;ko00940//Phenylpropanoid	-	GO:0003824//catalytic activity;GO:0016209//antioxidant activity;GO:0043169//cation binding;GO:0046906//tetrapyrrole binding	GO:0044710;GO:0006950//response to stress	gi|571525981|ref|XP_003548517.2|/0/PREDICTED: peroxidase 55-like [Glycine max]

												biosynthesis				
Glyma.16G080100	-	-	-	-	-	-	-	-	2.30011	0.00039	2340	-	GO:0031224//intrinsic component of membrane	GO:0005515//protein binding	GO:0006950//response to stress	gi|947057895|gb|KRH07301.1|;gi|734311089|gb|KHM99994.1|/0;0/hypothetical protein GLYMA_16G080100 [Glycine max];MLO-like protein 1 [Glycine soja]
Glyma.16G098700	-3.903	1.84E-10	-	-	-	-	-	-	-	-	1682	ko04141//Protein processing in endoplasmic reticulum	GO:0005911//cell-cell junction;GO:0044432	GO:0003824//catalytic activity	GO:0006950//response to stress;GO:0000904//cell morphogenesis involved in differentiation;GO:0044710	gi|955373994|ref|XP_014623933.1|/0/PREDICTED: dnaJ protein ERDJ3A-like [Glycine max]
Glyma.16G100400	-	-	-	-	-	-	1.29943	0.00097	-	-	537	-	GO:0005911//cell-cell junction;GO:0016020//membrane;GO:0005618//cell wall	GO:0016787//hydrolase activity	GO:0016485//protein processing;GO:0006950//response to stress	gi|734383103|gb|KHN23780.1|/2.1965e-29/Defensin SD2 [Glycine soja]
Glyma.16G129800	-	-	-	-	-	-	-	-	-1.1645	0.00248	1290	-	-	GO:0016671//oxidoreductase activity, acting on a sulfur group of donors, disulfide as acceptor	GO:0044710;GO:0044267//cellular protein metabolic process;GO:0006950//response to stress	gi|356559314|ref|XP_003547945.1|/8.30954e-177/PREDICTED: peptide methionine sulfoxide reductase A5-like [Glycine max]
Glyma.16G159700	2.02261	8.96E-05	-	-	-	-	-	-	4.85712	3.48E-05	3395	ko00230//Purine metabolism;ko00240//Pyrimidine metabolism;ko01100//Metabolic pathways;ko03020//RNA polymerase	-	GO:0016462//pyrophosphatase activity;GO:0032550	GO:0006950//response to stress;GO:0007154//cell communication	gi|571528807|ref|XP_006594456.1|/0/PREDICTED: TMV resistance protein N-like [Glycine max]
Glyma.16G164400	-	-	-	-	-	-	-	-	10.1391	2.44E-08	1424	ko01100//Metabolic pathways;ko01110//Biosynthesis of secondary metabolites;ko00360//Phenylalanine metabolism;ko00940//Phenylpropanoid biosynthesis	-	GO:0003824//catalytic activity;GO:0016209//antioxidant activity;GO:0043169//cation binding;GO:0046906//tetrapyrrole binding	GO:0044710;GO:0006950//response to stress	gi|947059246|gb|KRH08652.1|;gi|358249064|ref|NP_001239731.1|/0;0/hypothetical protein GLYMA_16G164400 [Glycine max];uncharacterized protein LOC100795412 precursor [Glycine max]
Glyma.16G211400	-	-	-	-	-	-	-	-	3.37201	0.00075	5242	-	-	GO:0016462//pyrophosphatase activity;GO:0032550	GO:0006950//response to stress;GO:0007154//cell communication	gi|947059957|gb|KRH09363.1|;gi|947059961|gb|KRH09367.1|;gi|947059960|gb|KRH09366.1|/0;0;0/hypothetical protein GLYMA_16G211400 [Glycine max];hypothetical protein GLYMA_16G211400 [Glycine max];hypothetical protein GLYMA_16G211400 [Glycine max]
Glyma.16G212300	-1.8558	0.000242	-	-	-	-	-	-	-	-	3786	ko00230//Purine metabolism;ko00240//Pyrimidine metabolism;ko01100//Metabolic pathways;ko03020//RNA polymerase	-	GO:0016462//pyrophosphatase activity;GO:0032550	GO:0006950//response to stress;GO:0007154//cell communication	gi|358248732|ref|NP_001239675.1|/0/TMV resistance protein N-like [Glycine max]
Glyma.17G030000	-	-	-	-	-2.7144	0.00105	-	-	2.67176	0.00631	1005	-	-	-	GO:0006950//response to stress	gi|734319289|gb|KHN03329.1|/1.47272e-115/Pathogenesis-related protein STH-2 [Glycine soja]

20 × 20

Glyma.17G030100	-	-	-	-	-	-	-	-	8.97181	8.60E-06	867	-	-	-	GO:0006950//response to stress	gi|734319288|gb|KHN03328.1|/4.37402e-111/Major allergen Pru av 1 [Glycine soja]
Glyma.17G030200	-3.5199	3.28E-08	-	-	-	-	1.77618	0.00059	5.06797	8.56E-10	947	-	-	-	GO:0006950//response to stress	gi|351725047|ref|NP_001236055.1|/4.96124e-92/uncharacterized protein LOC547916 [Glycine max]
Glyma.17G030300	-	-	-	-	-	-	-	-	9.30716	5.30E-06	896	-	-	-	GO:0006950//response to stress	gi|359807211|ref|NP_001241617.1|/4.85297e-92/uncharacterized protein LOC100791036 [Glycine max]
Glyma.17G047200	-	-	-	-	-	-	-	-	-1.3922	0.00167	908	-	GO:0009532//plastid stroma;GO:0009526//plastid envelope;GO:0009534//chloroplast thylakoid;GO:0005576//extracellular region	GO:0032550;GO:0046914//transition metal ion binding	GO:0010038//response to metal ion;GO:0044267//cellular protein metabolic process;GO:0051353//positive regulation of oxidoreductase activity;GO:0006950//response to stress	gi|947053124|gb|KRH02577.1|;gi|947053123|gb|KRH02576.1|/2.70356e-142;7.30452e-139/hypothetical protein GLYMA_17G047200 [Glycine max];hypothetical protein GLYMA_17G047200 [Glycine max]
Glyma.17G063200	-	-	-	-	-	-	-	-	-1.4648	0.00012	815	-	GO:0031981//nuclear lumen;GO:0009536//plastid;GO:0044437;GO:0000785//chromatin;GO:0005911//cell-cell junction;GO:0016020//membrane	GO:0003676//nucleic acid binding;GO:0046983//protein dimerization activity	GO:0034728//nucleosome organization;GO:0006950//response to stress	gi|475622287|gb|EMT31824.1|/1.35134e-52/Histone H4 [Aegilops tauschii]
Glyma.17G072400	-1.0878	2.04E-05	-	-	-	-	-	-	-	-	2310	ko04144//Endocytosis;ko04141//Protein processing in endoplasmic reticulum;ko03040//Spliceosome	GO:0016020//membrane;GO:0030312//external encapsulating structure;GO:0009536//plastid	GO:0032550	GO:0006950//response to stress;GO:0051707//response to other organism	gi|356562559|ref|XP_003549537.1|/0/PREDICTED: heat shock 70 kDa protein [Glycine max]
Glyma.17G145100	-	-	-	-	-	-	-	-	3.21091	0.00114	2868	-	-	GO:0004551//nucleotide diphosphatase activity;GO:0016787//hydrolase activity;GO:0003824//catalytic activity	GO:0006950//response to stress	gi|571532280|ref|XP_006600227.1|;gi|947054735|gb|KRH04188.1|;gi|947054734|gb|KRH04187.1|;gi|359806679|ref|NP_001241031.1|/0;0;0;0/PREDICTED: uncharacterized protein LOC100815507 isoform X1 [Glycine max];hypothetical protein GLYMA_17G145100 [Glycine max];hypothetical protein GLYMA_17G145100 [Glycine max];uncharacterized protein LOC100815507 [Glycine max]

20 × 20

Glyma.17G218700	-	-	-	-	-	-	-	-	1.35911	0.00956	3043	ko04075//Plant hormone signal transduction	-	GO:0043169//cation binding;GO:0004721//phosphoprotein phosphatase activity	GO:0009767//photosynthetic electron transport chain;GO:0001933//negative regulation of protein phosphorylation;GO:0009738//abscisic acid-activated signaling pathway;GO:0006950//response to stress;GO:0044763;GO:0006464//cellular protein modification process;GO:0031324//negative regulation of cellular metabolic process;GO:0006796//phosphate-containing compound metabolic process;GO:0009737//response to abscisic acid	gi|356563938|ref|XP_003550214.1|;gi|734373231|gb|KHN20160.1|;gi|947055849|gb|KRH05302.1|;gi|571538689|ref|XP_006601194.1|/0;0;0;0/PREDICTED: protein phosphatase 2C 77-like isoform X3 [Glycine max];Protein phosphatase 2C 16 [Glycine soja] ;hypothetical protein GLYMA_17G218700 [Glycine max];PREDICTED: protein phosphatase 2C 77-like isoform X4 [Glycine max]
Glyma.17G237900	-	-	-	-	-	-	-	-	-1.2807	6.55E-04	2177	-	-	GO:0005488	GO:0010035//response to inorganic substance;GO:0051707//response to other organism;GO:0001101//response to acid chemical;GO:0009628//response to abiotic stimulus;GO:0009725//response to hormone;GO:0009966//regulation of signal transduction;GO:0006950//response to stress	gi|947056168|gb|KRH05621.1|/3.38894e-144/hypothetical protein GLYMA_17G237900 [Glycine max]
Glyma.17G246500	-	-	-	-	-	-	1.31886	0.00049	2.51887	2.71E-09	2795	ko01100//Metabolic pathways;ko00592//alpha-Linolenic acid metabolism	GO:0009526//plastid envelope;GO:0009534//chloroplast thylakoid	GO:0046906//tetrapyrrole binding;GO:0046914//transition metal ion binding;GO:0016491//oxidoreductase activity;GO:0016836//hydro-lyase activity	GO:0051707//response to other organism;GO:0006631//fatty acid metabolic process;GO:0001101//response to acid chemical;GO:0006950//response to stress	gi|351721547|ref|NP_001236445.1|/0/allene oxide synthase [Glycine max]
Glyma.18G009700	1.38422	6.90E-11	-	-	-	-	-	-	-	-	3145	ko00010//Glycolysis / Gluconeogenesis;ko01100//Metabolic pathways;ko01110//Biosynthesis of secondary metabolites	GO:0030312//external encapsulating structure;GO:0031981//nuclear lumen;GO:0009536//plastid;GO:0005911//cell-cell junction;GO:0016020//membrane;GO:0043231//intracellular membrane-bounded organelle	GO:0046914//transition metal ion binding;GO:0016903//oxidoreductase activity, acting on the aldehyde or oxo group of donors;GO:0000166//nucleotide binding;GO:0005488	GO:0010038//response to metal ion;GO:0019318//hexose metabolic process;GO:0009617//response to bacterium;GO:0006950//response to stress;GO:0050896//response to stimulus	gi|947047940|gb|KRG97468.1|;gi|358248010|ref|NP_001240046.1|;gi|947047937|gb|KRG97465.1|;gi|947047939|gb|KRG97467.1|/2.43442e-146;0;0;0/hypothetical protein GLYMA_18G009700 [Glycine max];uncharacterized protein LOC100782924 [Glycine max];hypothetical protein GLYMA_18G009700 [Glycine max];hypothetical protein GLYMA_18G009700 [Glycine max]

20 × 20

Glyma.18G043700	1.0456	0.005885	-	-	-	-	-	-	3.30405	7.82E-07	3445	ko00480//Glutathione metabolism	GO:0000325//plant-type vacuole	GO:0043167//ion binding;GO:0016765//transferase activity, transferring alkyl or aryl (other than methyl) groups	GO:0006950//response to stress;GO:0009962//regulation of flavonoid biosynthetic process	gi|359807572|ref|NP_001241411.1|/1.0728e-157/glutathione S-transferase F11-like [Glycine max]
Glyma.18G055300	-1.8657	6.03E-09	-	-	-	-	-	-	-	-	1252	ko01100//Metabolic pathways;ko01110//Biosynthesis of secondary metabolites;ko00360//Phenylalanine metabolism;ko00940//Phenylpropanoid biosynthesis	-	GO:0003824//catalytic activity;GO:0016209//antioxidant activity;GO:0043169//cation binding;GO:0046906//tetrapyrrole binding	GO:0044710;GO:0006950//response to stress	gi|356565900|ref|XP_003551174.1|/0/PREDICTED: peroxidase 4-like [Glycine max]
Glyma.18G118100	-	-	-	-	-	-	-	-	1.32268	0.00626	2113	ko00909//Sesquiterpenoid and triterpenoid biosynthesis;ko01100//Metabolic pathways;ko01110//Biosynthesis of secondary metabolites	GO:0031224//intrinsic component of membrane	GO:0016709//oxidoreductase activity, acting on paired donors, with incorporation or reduction of molecular oxygen, NAD(P)H as one donor, and incorporation of one atom of oxygen;GO:0000166//nucleotide binding	GO:0006694//steroid biosynthetic process;GO:0006950//response to stress;GO:0001101//response to acid chemical;GO:0044710	gi|947049536|gb|KRG99064.1|;gi|356569981|ref|XP_003553171.1|;gi|947049537|gb|KRG99065.1|/0;0;0/hypothetical protein GLYMA_18G118100 [Glycine max];PREDICTED: squalene epoxidase 3-like [Glycine max];hypothetical protein GLYMA_18G118100 [Glycine max]
Glyma.18G204000	-	-	-	-	-	-	-	-	-1.8469	6.16E-07	2159	-	GO:0005911//cell-cell junction;GO:0044432	GO:0003824//catalytic activity	GO:0006950//response to stress;GO:0000904//cell morphogenesis involved in differentiation;GO:0044710	gi|356566692|ref|XP_003551564.1|/0/PREDICTED: dnaJ protein ERDJ3A [Glycine max]
Glyma.18G204200	-	-	-	-	-	-	-	-	1.55495	1.39E-05	2393	ko00010//Glycolysis / Gluconeogenesis;ko01100//Metabolic pathways;ko01110//Biosynthesis of secondary metabolites	GO:0044444	GO:0046872//metal ion binding;GO:0019842//vitamin binding	GO:0006950//response to stress	gi|356568132|ref|XP_003552267.1|/0/PREDICTED: pyruvate decarboxylase 2 [Glycine max]
Glyma.18G215000	-	-	-	-	-	-	-	-	-1.2931	0.00044	1168	-	GO:0016020//membrane	-	GO:0006950//response to stress;GO:0048229//gametophyte development	gi|359806290|ref|NP_001241475.1|;gi|947050936|gb|KRH00465.1|/2.96668e-150;6.13158e-117/uncharacterized protein LOC100810722 precursor [Glycine max] ;hypothetical protein GLYMA_18G215000 [Glycine max]
Glyma.18G219100	-	-	-	-	-	-	-	-	1.04107	0.00572	2336	ko00010//Glycolysis / Gluconeogenesis;ko01100//Metabolic pathways;ko01110//Biosynthesis of secondary metabolites;ko00260//Glycine, serine and threonine metabolism	GO:0031967;GO:0009536//plastid;GO:0005911//cell-cell junction;GO:0005576//extracellular region;GO:0016020//membrane	GO:0046914//transition metal ion binding;GO:0004619//phosphoglycerate mutase activity	GO:0010038//response to metal ion;GO:0044763;GO:0048229//gametophyte development;GO:0006091//generation of precursor metabolites and energy;GO:0006006//glucose metabolic process;GO:0006950//response to stress	gi|734420079|gb|KHN40585.1|/0/2,3-bisphosphoglycerate-independent phosphoglycerate mutase [Glycine soja]
Glyma.18G238500	-	-	-	-	-	-	-	-	-1.36	0.00195	1650	-	-	GO:0016301//kinase activity	GO:0019751//polyol metabolic process;GO:0006796//phosphate-containing compound metabolic process;GO:0006950//response to stress;GO:0007186//G-protein coupled receptor signaling pathway	gi|947051332|gb|KRH00861.1|;gi|356566923|ref|XP_003551674.1|/0;0/hypothetical protein GLYMA_18G238500 [Glycine max];PREDICTED: sphingoid long-chain bases kinase 2, mitochondrial-like [Glycine max]

20 × 20

Glyma.18G278900	-	-	-	-	-	-	-	-	2.57163	0.00135	2019	ko00330//Arginine and proline metabolism;ko01100//Metabolic pathways;ko01110//Biosynthesis of secondary metabolites	-	GO:0016645//oxidoreductase activity, acting on the CH-NH group of donors	GO:0009617//response to bacterium;GO:0006560//proline metabolic process;GO:0006536//glutamate metabolic process;GO:0006950//response to stress	gi|356568869|ref|XP_003552630.1|/0/PREDICTED: proline dehydrogenase 2, mitochondrial [Glycine max]
Glyma.19G011700	-	-	-	-	-	-	-	-	8.60825	1.84E-05	2324	ko00592//alpha-Linolenic acid metabolism	-	GO:0016209//antioxidant activity;GO:0046906//tetrapyrrole binding	GO:0044710;GO:0006950//response to stress	gi|734382169|gb|KHN23570.1|/0/Prostaglandin G/H synthase 2 [Glycine soja]
Glyma.19G016100	-	-	-	-	-3.9277	0.00026	-	-	-	-	1822	ko00500//Starch and sucrose metabolism;ko01110//Biosynthesis of secondary metabolites;ko00520//Amino sugar and nucleotide sugar metabolism	-	GO:0016740//transferase activity	GO:0006950//response to stress	gi|356572000|ref|XP_003554158.1|/0/PREDICTED: probable galacturonosyltransferase-like 10 [Glycine max]
Glyma.19G066200	-	-	-	-	-	-	-	-	2.37898	6.23E-05	1166	ko01100//Metabolic pathways;ko01110//Biosynthesis of secondary metabolites;ko00360//Phenylalanine metabolism;ko00940//Phenylpropanoid biosynthesis	-	GO:0003824//catalytic activity;GO:0016209//antioxidant activity;GO:0043169//cation binding;GO:0046906//tetrapyrrole binding	GO:0044710;GO:0006950//response to stress	gi|734305902|gb|KHM98789.1|/0/Peroxidase 44 [Glycine soja]
Glyma.19G091800	-2.9749	1.32E-05	-	-	-	-	-	-	-	-	1509	ko01100//Metabolic pathways;ko01110//Biosynthesis of secondary metabolites;ko00360//Phenylalanine metabolism;ko00940//Phenylpropanoid biosynthesis	-	GO:0003824//catalytic activity;GO:0016209//antioxidant activity;GO:0043169//cation binding;GO:0046906//tetrapyrrole binding	GO:0044710;GO:0006950//response to stress	gi|356571531|ref|XP_003553930.1|/0/PREDICTED: peroxidase 55 [Glycine max]
Glyma.19G105100	-	-	-1.25	0.000682	-	-	2.71529	3.54E-11	-	-	1531	ko00941//Flavonoid biosynthesis;ko01100//Metabolic pathways;ko01110//Biosynthesis of secondary metabolites;ko04712//Circadian rhythm - plant	GO:0000325//plant-type vacuole	GO:0016746//transferase activity, transferring acyl groups	GO:0009411//response to UV;GO:0060918//auxin transport;GO:0001101//response to acid chemical;GO:0009718//anthocyanin-containing compound biosynthetic process;GO:0009725//response to hormone;GO:0006950//response to stress	gi|356571645|ref|XP_003553986.1|/0/PREDICTED: chalcone synthase J-like [Glycine max]
Glyma.19G172500	-	-	-	-	-	-	-	-	-1.1328	0.00146	1082	-	GO:0031981//nuclear lumen;GO:0009536//plastid;GO:0044437;GO:0000785//chromatin;GO:0005911//cell-cell junction;GO:0016020//membrane	GO:0003676//nucleic acid binding;GO:0046983//protein dimerization activity	GO:0034728//nucleosome organization;GO:0006950//response to stress	gi|571558188|ref|XP_006604532.1|/6.08435e-89/PREDICTED: histone H4-like [Glycine max]

20 × 20

Glyma.19G181300	-	-	-	-	-	-	-	-	-2.2821	5.65E-07	1588	-	GO:0031224//intrinsic component of membrane;GO:0005911//cell-cell junction;GO:0009536//plastid	GO:0044389//ubiquitin-like protein ligase binding;GO:0005372//water transmembrane transporter activity	GO:0051707//response to other organism;GO:0042044//fluid transport;GO:0006950//response to stress;GO:0055085//transmembrane transport;GO:0001101//response to acid chemical	gi|947046352|gb|KRG95981.1|;gi|356572440|ref|XP_003554376.1|/3.54333e-159;0/hypothetical protein GLYMA_19G181300 [Glycine max];PREDICTED: aquaporin PIP2-1 [Glycine max]
Glyma.19G240400	-	-	-	-	-	-	-	-	-1.0373	0.00366	1574	ko04146//Peroxisome	GO:0044424	GO:0043169//cation binding;GO:0016209//antioxidant activity;GO:0003824//catalytic activity	GO:0009642//response to light intensity;GO:0010193//response to ozone;GO:0010224//response to UV-B;GO:0044710;GO:0009651//response to salt stress;GO:0072593//reactive oxygen species metabolic process;GO:0009617//response to bacterium;GO:0009744//response to sucrose;GO:0035194//posttranscriptional gene silencing by RNA;GO:0046688//response to copper ion;GO:0009987//cellular process;GO:0010035//response to inorganic substance;GO:0009628//response to abiotic stimulus;GO:0008152//metabolic process;GO:0006950//response to stress	gi|351725359|ref|NP_001235298.1|;gi|947047281|gb|KRG96910.1|;gi|947047280|gb|KRG96909.1|/1.08219e-93;1.44827e-53;8.32826e-66/superoxide dismutase [Cu-Zn] [Glycine max];hypothetical protein GLYMA_19G240400 [Glycine max];hypothetical protein GLYMA_19G240400 [Glycine max]
Glyma.20G017900	5.90078	5.72E-14	-	-	-	-	-	-	-	-	1145	-	-	-	GO:0006950//response to stress	gi|359807343|ref|NP_001240867.1|/1.92537e-107/MLP-like protein 28-like [Glycine max]
Glyma.20G026700	-	-	-	-	-	-	-	-	5.10843	9.60E-06	3557	ko00500//Starch and sucrose metabolism	-	GO:0043168//anion binding;GO:0004645//phosphorylase activity	GO:0044238//primary metabolic process;GO:0006950//response to stress;GO:0009628//response to abiotic stimulus	gi|571563869|ref|XP_006605545.1|;gi|356577161|ref|XP_003556696.1|/0;0/PREDICTED: alpha-1,4 glucan phosphorylase L-2 isozyme, chloroplastic/amyloplastic-like isoform X2 [Glycine max];PREDICTED: alpha-1,4 glucan phosphorylase L-2 isozyme, chloroplastic/amyloplastic-like isoform X1 [Glycine max]
Glyma.20G053700	-	-	-	-	-1.5643	0.00027	-	-	2.23282	0.00967	3225	ko00591//Linoleic acid metabolism;ko01100//Metabolic pathways;ko00592//alpha-Linolenic acid metabolism	GO:0009532//plastid stroma;GO:0009526//plastid envelope;GO:0009534//chloroplast thylakoid	GO:0046914//transition metal ion binding;GO:0016702//oxidoreductase activity, acting on single donors with incorporation of molecular oxygen, incorporation of two atoms of oxygen;GO:0032550	GO:0051707//response to other organism;GO:0006633//fatty acid biosynthetic process;GO:0055114//oxidation-reduction process;GO:0001101//response to acid chemical;GO:0006950//response to stress	gi|947040160|gb|KRG89884.1|;gi|356575019|ref|XP_003555640.1|/0;0/hypothetical protein GLYMA_20G053700 [Glycine max];PREDICTED: linoleate 13S-lipoxygenase 2-1, chloroplastic-like [Glycine max]
Glyma.20G079300	-	-	-	-	-	-	-1.0971	0.0003	-	-	2229	ko03018//RNA degradation	GO:0005840//ribosome;GO:0009532//pla	GO:0032550;GO:0046914//transition metal ion binding	GO:0010038//response to metal ion;GO:0006457//protein folding;GO:0006950//respons	gi|734401136|gb|KHN31621.1|/0/Chaperonin CPN60-2, mitochondrial [Glycine soja]

													stid stroma;GO:0009526//plastid envelope;GO:0044437;GO:0044429;GO:0016020//membrane		e to stress	
Glyma.20G080000	-1.6661	0.000972	-	-	-	-	-	-	-3.5159	4.17E-05	1899	ko00380//Tryptophan metabolism	-	GO:0000166//nucleotide binding;GO:0016709//oxidoreductase activity, acting on paired donors, with incorporation or reduction of molecular oxygen, NAD(P)H as one donor, and incorporation of one atom of oxygen	GO:0009683//indoleacetic acid metabolic process;GO:0006950//response to stress	gi|571565271|ref|XP_003555725.2|0/PREDICTED: probable indole-3-pyruvate monooxygenase YUCCA7 [Glycine max]
Glyma.20G083800	-	-	-	-	-	-	-	-	-1.2725	0.00064	1088	-	GO:0031981//nuclear lumen;GO:0009536//plastid;GO:0044437;GO:0000785//chromatin;GO:0005911//cell-cell junction;GO:0016020//membrane	GO:0003676//nucleic acid binding;GO:0046983//protein dimerization activity	GO:0034728//nucleosome organization;GO:0006950//response to stress	gi|475622287|gb|EMT31824.1|1.35134e-52/Histone H4 [Aegilops tauschii]
Glyma.20G122500	-	-	-	-	-	-	-	-	1.76847	1.93E-06	1694	ko00051//Fructose and mannose metabolism;ko00030//Pentose phosphate pathway;ko00010//Glycolysis / Gluconeogenesis;ko01100//Metabolic pathways;ko01110//Biosynthesis of secondary metabolites;ko00710//Carbon fixation in photosynthetic organisms	GO:0031976;GO:0009532//plastid stroma	GO:0016832//aldehyde-lyase activity	GO:0006950//response to stress;GO:0010038//response to metal ion;GO:0006091//generation of precursor metabolites and energy	gi|947041204|gb|KRG90928.1|0/hypothetical protein GLYMA_20G122500 [Glycine max]
Glyma.20G133200	-	-	-1.4898	0.000798	-3.1996	0.00043	-	-	1.3797	0.00326	1580	-	-	-	GO:0009628//response to abiotic stimulus;GO:0006950//response to stress	gi|918463665|gb|ALA09177.1|2.67846e-160/C2H2-Zn transcription factor, partial [Glycine max]
Glyma.20G169200	-	-	-	-	-	-	1.27701	0.00011	-	-	1364	ko01100//Metabolic pathways;ko01110//Biosynthesis of secondary metabolites;ko00360//Phenylalanine metabolism;ko00940//Phenylpropanoid biosynthesis	GO:0005911//cell-cell junction;GO:0005618//cell wall	GO:0003824//catalytic activity;GO:0016209//antioxidant activity;GO:0043169//cation binding;GO:0046906//tetrapyrrole binding	GO:0044710;GO:0006950//response to stress	gi|356576075|ref|XP_003556160.1|0/PREDICTED: peroxidase 12-like [Glycine max]
Glyma.20G197100	-	-	-	-	-	-	-1.0367	0.00127	-	-	2151	ko03018//RNA degradation	GO:0005840//ribosome;GO:0009532//pla	GO:0032550;GO:0046914//transition metal ion binding	GO:0010038//response to metal ion;GO:0006457//protein folding;GO:0006950//respons	gi|356576411|ref|XP_003556325.1|0/PREDICTED: chaperonin CPN60-2, mitochondrial [Glycine max]

20 × 20

													stid stroma;GO:0009526//plastid envelope;GO:0044437;GO:0044429;GO:0016020//membrane		e to stress	
Glyma.20G210800	-	-	-	-	-	-	-	-	2.68797	0.00053	1691	ko04140//Regulation of autophagy	-	GO:0004672//protein kinase activity;GO:0032550	GO:0006796//phosphate-containing compound metabolic process;GO:0006950//response to stress;GO:0006464//cellular protein modification process;GO:0007154//cell communication	gi|356576545|ref|XP_003556391.1|/0/PREDICTED: CBL-interacting serine/threonine-protein kinase 4-like [Glycine max]
Glyma.20G230900	-	-	-	-	-	-	-	-	1.73223	0.00694	2490	-	-	-	GO:0006950//response to stress	gi|947043086|gb|KRG92810.1|;gi|947043085|gb|KRG92809.1|/0;0/hypothetical protein GLYMA_20G230900 [Glycine max];hypothetical protein GLYMA_20G230900 [Glycine max]
Glyma.U033000	-2.5662	9.41E-08	-	-	-	-	-	-	-	-	2316	ko00062//Fatty acid elongation;ko01110//Biosynthesis of secondary metabolites	GO:0043231//intracellular membrane-bounded organelle	GO:0016746//transferase activity, transferring acyl groups	GO:0009314//response to radiation;GO:0006950//response to stress;GO:0006631//fatty acid metabolic process	gi|947038781|gb|KRG88669.1|/0/hypothetical protein GLYMA_U033000 [Glycine max]

20 × 20

Supplementary Table S7: Primers of qPCR

Gene	Size	Sequence(5'-3')
Glyma.11G238800	138 bp	F: GGTGGAAACAACGGCTCAAC
		R: GGACCCAACTCGGATAGCTG
Glyma.14G072200	100 bp	F: GACGCAGCTGACTGGGTTTA
		R: GTATGCGGACCACTTTCCCA
Glyma.16G065700	277 bp	F: AGCAAATGCTTTGTTGAAGTGGAA
		R: AGTGTTGAGATTGGATAAGGAACCA
Glyma.12G210600	159 bp	F: ACCTTCAAGAAGCTTGTGGCT
		R: TGTCAAACAATTCCTTATCCTTCCA
Glyma.08G109300	132 bp	F: AGCACATGACCGAGCTCAAA
		R: ACGAAGGTGCCATGTAAGCA
Glyma.12G232500	84 bp	F: GGAATCGTGTTAGGTTTAATTCCCA
		R: CAACTGATCACCTCGTCGGT
Glyma.16G214900	286 bp	F: TCATCGAAGGCAAGTTTAAAGAGT
		R: TAAGCTTCCTGCAACCAGCA
Glyma.09G073600	204 bp	F: TGGCCTCATCGAGACCTACA
		R: CCACCATTGCATGTGGCAAA
ACT11	213 bp	F: CAACCCAAAGGTCAACAG
		R: CAGCGAGATCCAAACGAA
